# Supplementary material for: Development of a natural product optimization strategy for inhibitors against MraY, a promising antibacterial target
Source: Nat Commun. 2024 Jun 14;15:5085. doi: 10.1038/s41467-024-49484-7 (PMC11178787; doi:10.1038/s41467-024-49484-7)
Supplement: Supplementary file 1 — Supplementary Information [file 41467_2024_49484_MOESM1_ESM.pdf]

Supplementary Information

Development of a Natural Product Optimization Strategy for inhibitors against MraY, a promising antibacterial target

Kazuki Yamamoto,[1,2*] Toyotaka Sato,[3,4,5] Aili Hao,[6] Kenta Asao,[1] Rintaro Kaguchi,[1] Shintaro Kusaka,[1] Radhakrishnam Raju Ruddarraju,[1] Daichi Kazamori,[7] Kiki Seo,[7] Satoshi Takahashi,[8,9] Motohiro Horiuchi,[3,4,5] Shin-ichi Yokota,[10] Seok-Yong Lee,[6] and Satoshi Ichikawa,[1,2,11*]

Contents

1. Supplementary Figures and Tables **2-39**

2. Supplementary Notes

2-1. Detailed discussions about the results of the library of MraY inhibitors **40-41**

2-2. Application of build-up library synthesis for tubulin-binding natural products **42-49**

3. Supplementary methods

3-1. Preparation of compounds **50-88**

3-2. Biological evaluations of library of tubulin-binding natural products **89**

3-3. ^{1}H, ^{13}C NMR spectrum of compounds **90-133**

4. Supplementary References **134-138**

1. Supplementary Figures and Tables

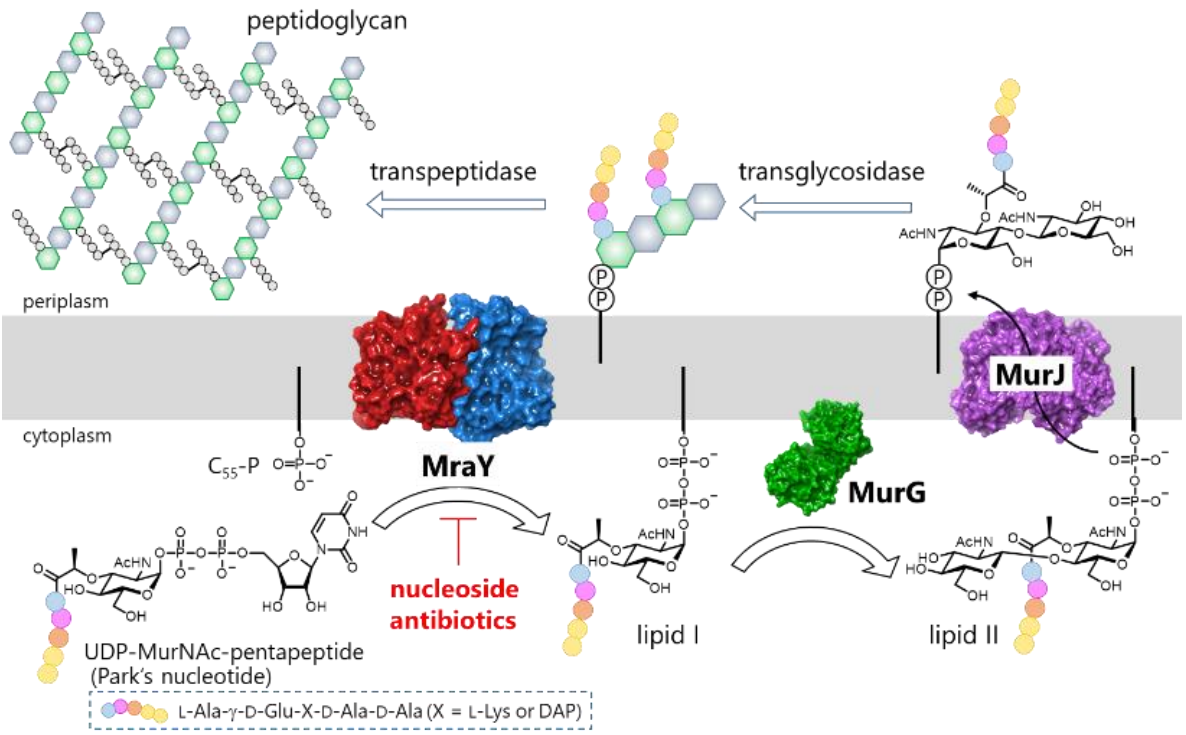

Supplementary Fig. 1. Peptidoglycan biosynthesis pathway. β-Lactams inhibit transpeptidases such as penicillin-binding proteins (PBPs). Vancomycin binds to the D-Ala-D-Ala terminal of lipid II and inhibits transpeptidation and transglycosidation reaction. MraY is an enzyme located upstream of peptidoglycan biosynthesis.

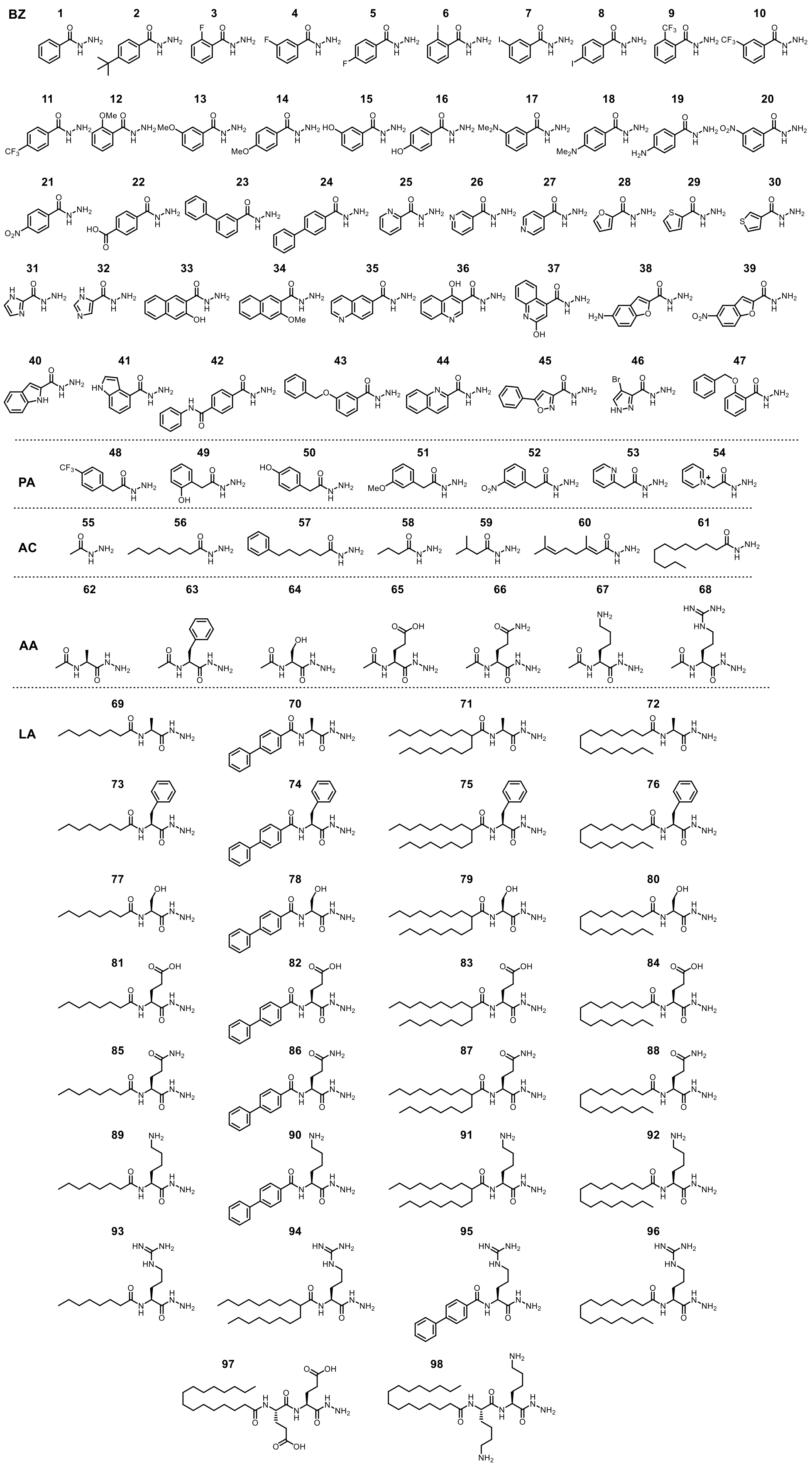

Supplementary Fig. 2. Structures of hydrazine fragments.

a) general scheme

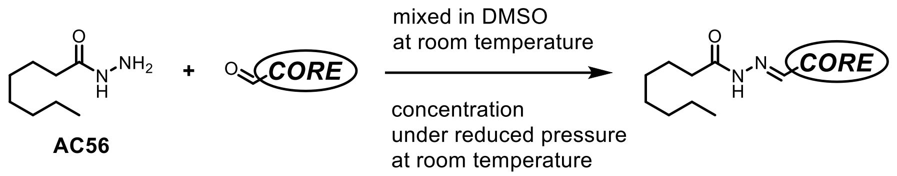

b) buffer only

MraY assay buffer

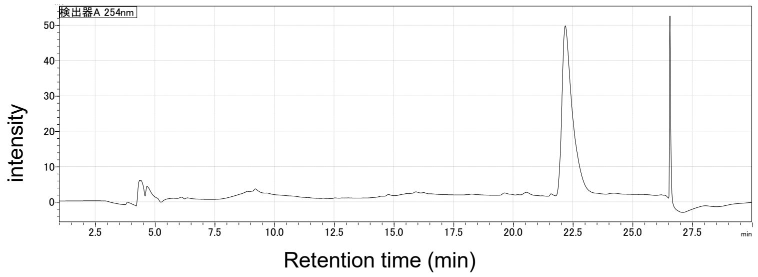

Mueller-Hinton-II broth (MHB)

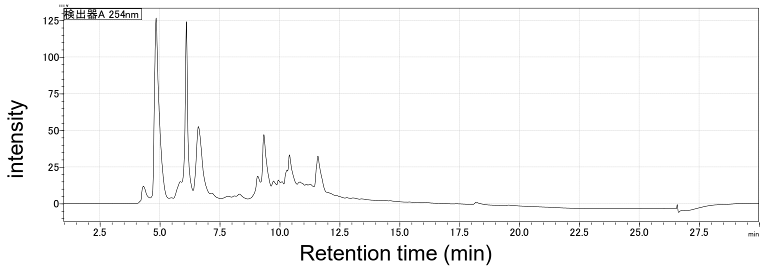

Supplementary Fig. 3. Analyses of hydrazone formation and stability in assay conditions (general scheme and chromatograms of buffer only).

LC-MS conditions; 5% to 90% MeCN in 0.1% *aq.* HCO_2H gradient elution. UV (254 nm) detector was used. Details were shown in the experimental section.

Detailed discussion

We monitored the hydrazone formation using our set of core aldehydes and octanohydrazide (**AC56**) as a model hydrazide to optimize reaction conditions (Supplementary Fig. 3-7). For example, **TUNp-CHO** and **AC56** were mixed in DMSO in a microtube at room temperature for 30 minutes, and the mixture was concentrated under reduced pressure at room temperature overnight, and the progress of the reaction was subsequently analyzed by LC-MS. We observed a peak corresponding to TUNp-CHO completely disappeared while a new peak corresponding to a hydrazone concomitantly appeared, indicating that the desired *N*-octanoyl hydrazone (TUNp-AC56) formation proceeded quantitatively (Supplementary Fig. 4a, b). Next, the stability of the obtained TUNp-AC56 under several assay conditions was investigated. The hydrazone barely decomposed in the MraY assay buffer at 25 °C for 3 hours (Supplementary Fig. 4c); however, in a bacteria culture medium at 37 °C for 18 hours, a small amount of TUNp-CHO was identified, but more than 70% of the hydrazone remained (Supplementary Fig. 4d). These results indicate that the hydrazone is sufficiently stable under the assay conditions to evaluate MraY inhibition and antibacterial activity. We also demonstrated that hydrazone formation with other core aldehydes and AC56 proceeds cleanly (Supplementary Fig. 5b, 6b, 7b).

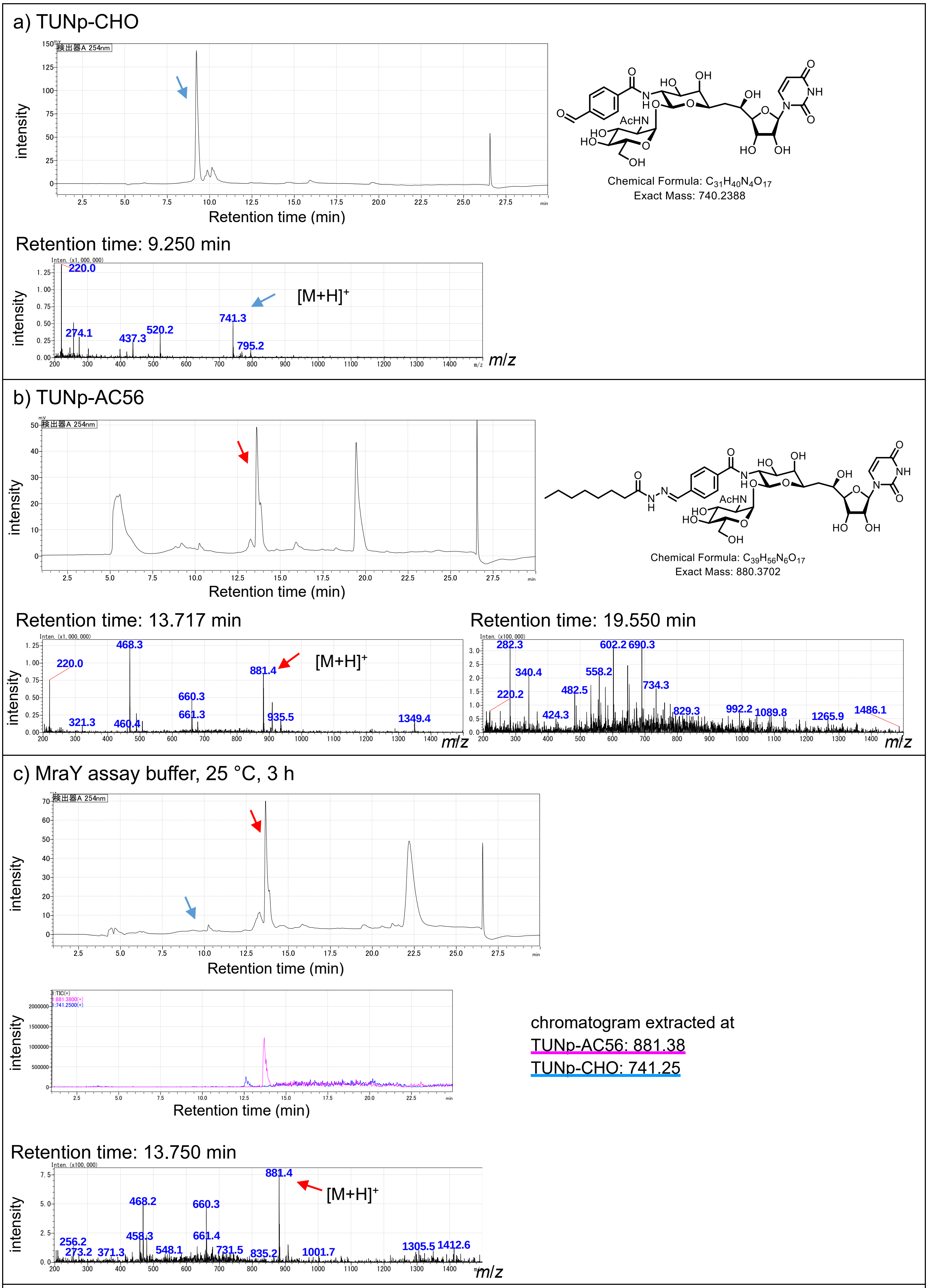

Supplementary Fig. 4. Analyses of hydrazone formation and stability in assay conditions (TUNp-type).

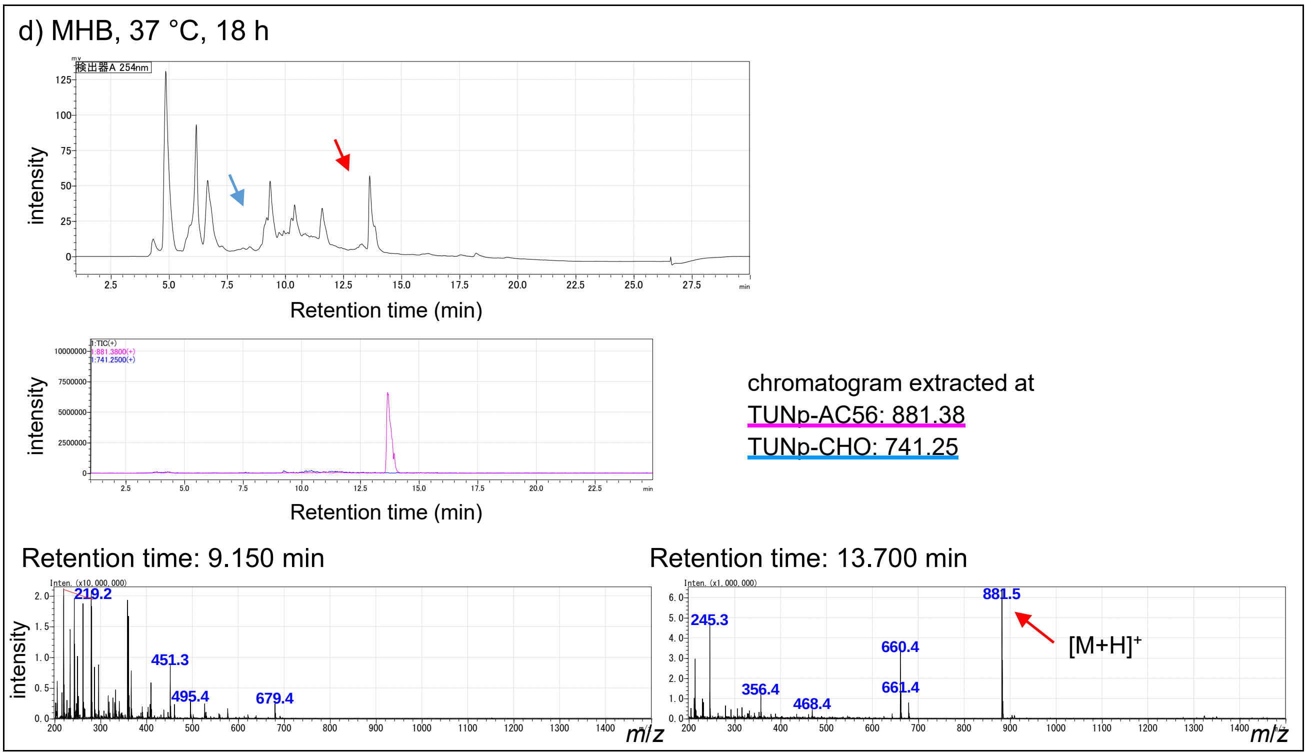

Supplementary Fig. 4. continued.

a) A peak at 9.3 minutes were identical to TUNp-CHO (cyan arrow). b) A peak at 13.7 minutes were identical to TUNp-AC56 (red arrow). Peaks at 5-7 min were results from injecting DMSO solutions, and a peak at 19-20 min were derived from treating microtube with DMSO, in which masses that appear to be polymers were detected. c) TUNp-AC56 were stable in the MraY assay buffer at 25 °C for 3 h. The peaks derived from hydrazone (10-15 min) and Triton X-100 (22.5 min) were detected. d) The TUNp-CHO peak overlapped the peaks derived from the broth. The mass peak of aldehyde oxidized carboxylic acid was not detected.

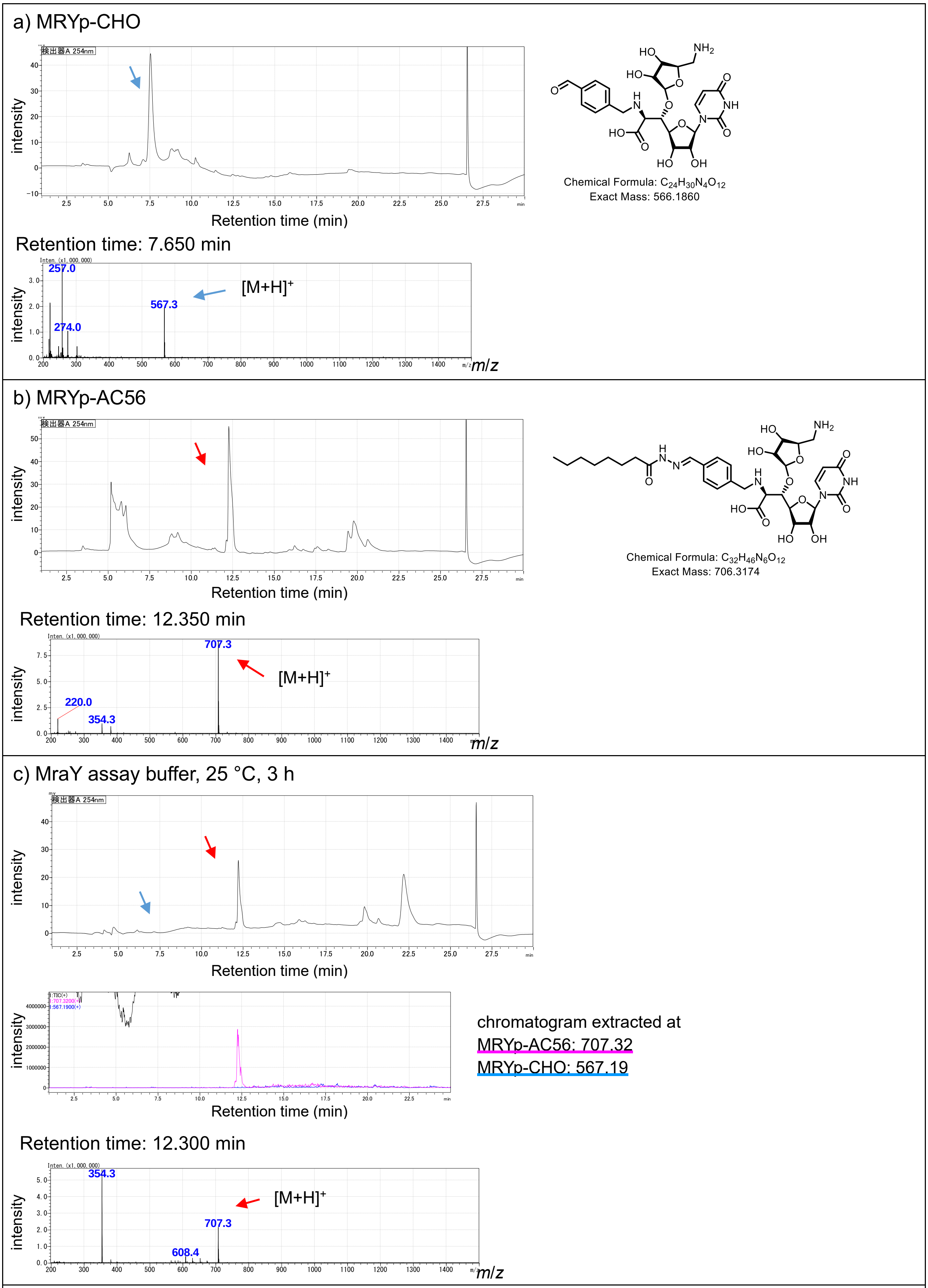

Supplementary Fig. 5. Analyses of hydrazone formation and stability in assay conditions (MRYp-type).

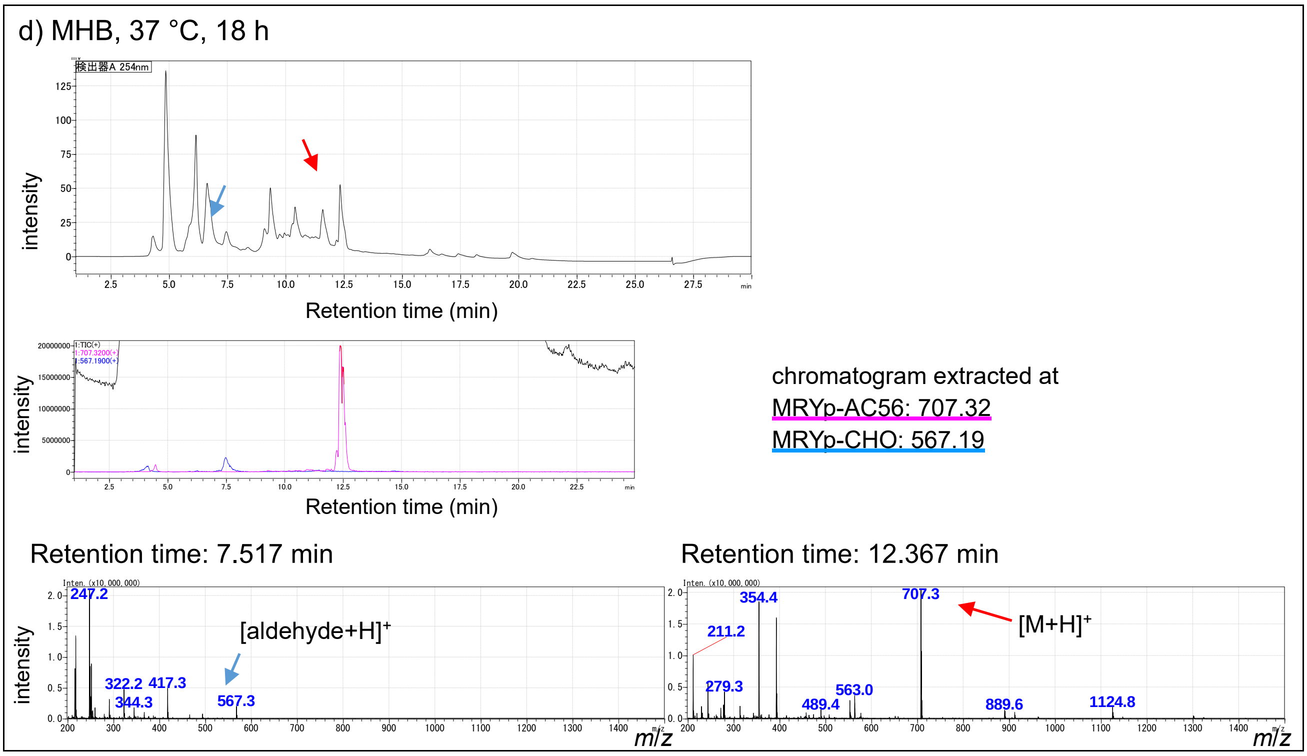

Supplementary Fig. 5. continued.

a) A peak at 7.7 minutes were identical to MRYp-CHO (cyan arrow). b) A peak at 12.4 minutes were identical to MRYp-AC56 (red arrow). Peaks at 5-7 min were results from injecting DMSO solutions, and a peak at 19-20 min were derived from treating microtube with DMSO, in which masses that appear to be polymers were detected. c) MRYp-AC56 were stable in the MraY assay buffer at 25 °C for 3 h. The peaks derived from hydrazone (12.0-12.5 min.) and Triton X-100 (22.5 min.) were detected. d) The MRYp-CHO peak was detected at 7.5 minutes.

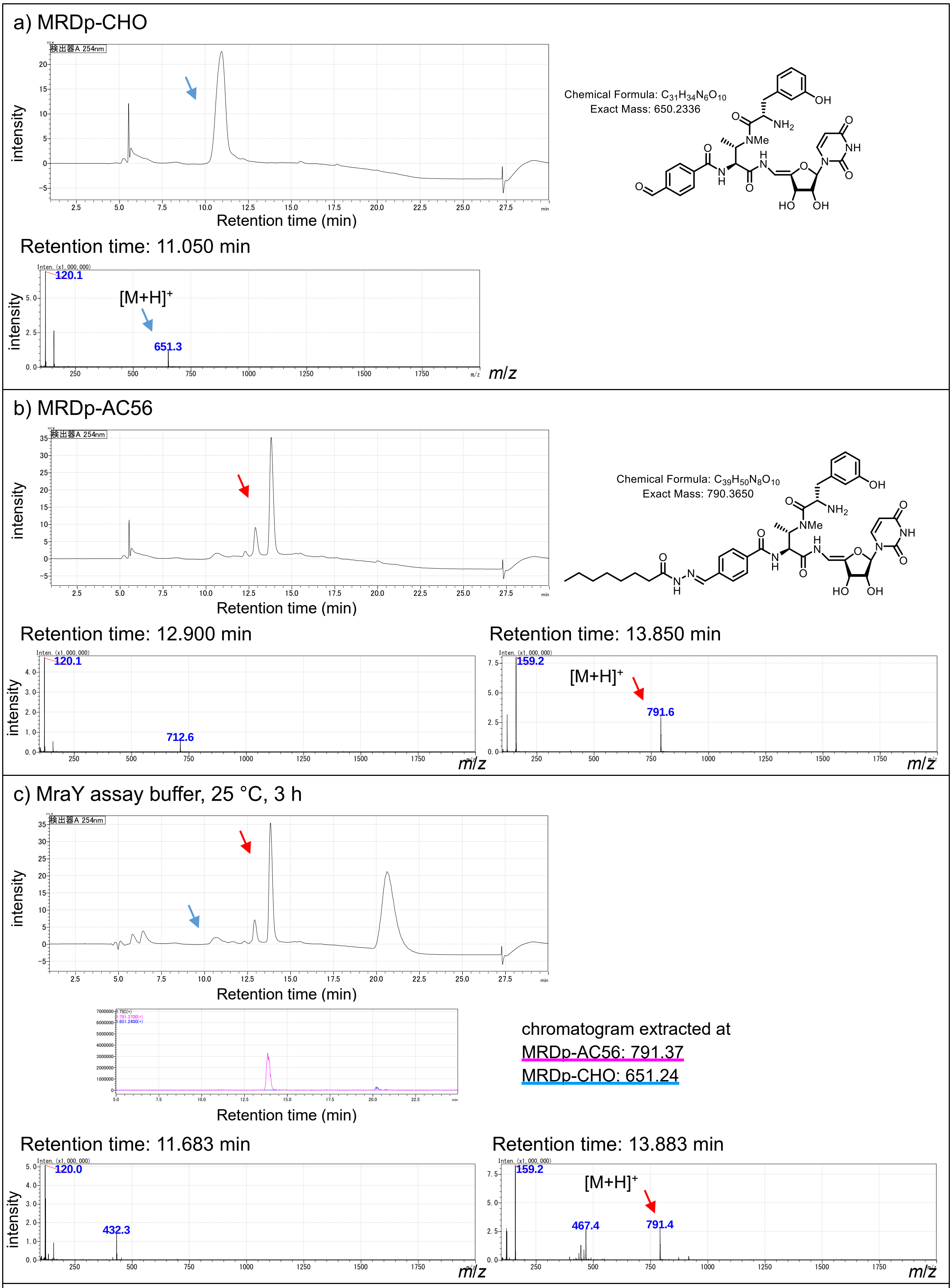

Supplementary Fig. 6. Analyses of hydrazone formation and stability in assay conditions (MRDp-type).

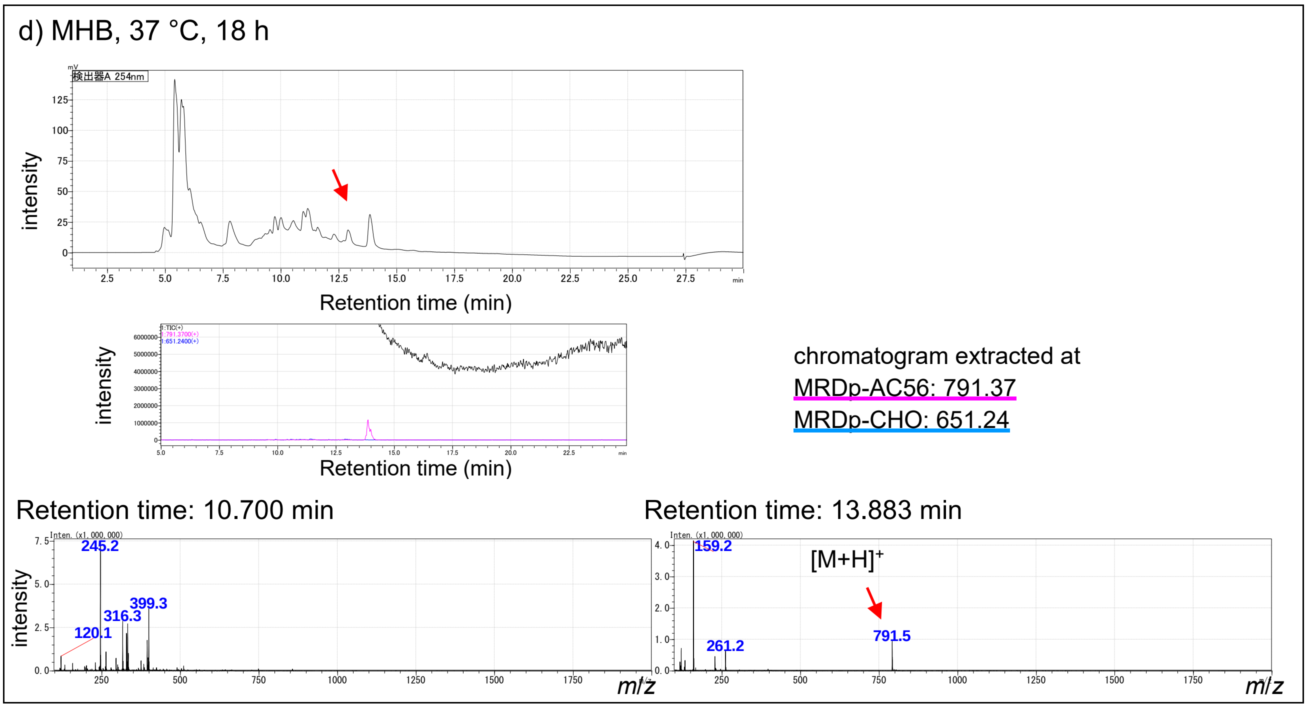

Supplementary Fig. 6. continued.

a) A peak at 11 minutes were identical to MRDp-CHO (cyan arrow). b) A peak at 13.9 minutes were identical to MRDp-AC56 (red arrow). An unknown peak was detected at 12.9 minutes. c) MRDp-AC56 were stable in the MraY assay buffer at 25 °C for 3 h. The peaks derived from hydrazone (13.5-14 min.) and Triton X-100 (22.5 min.) were detected. No mass of MRDp-CHO was detected in the peak with the same retention time as the MRDp-CHO. d) The MRDp-CHO peak overlapped the peaks derived from the broth. The mass peak of aldehyde oxidized carboxylic acid was not detected.

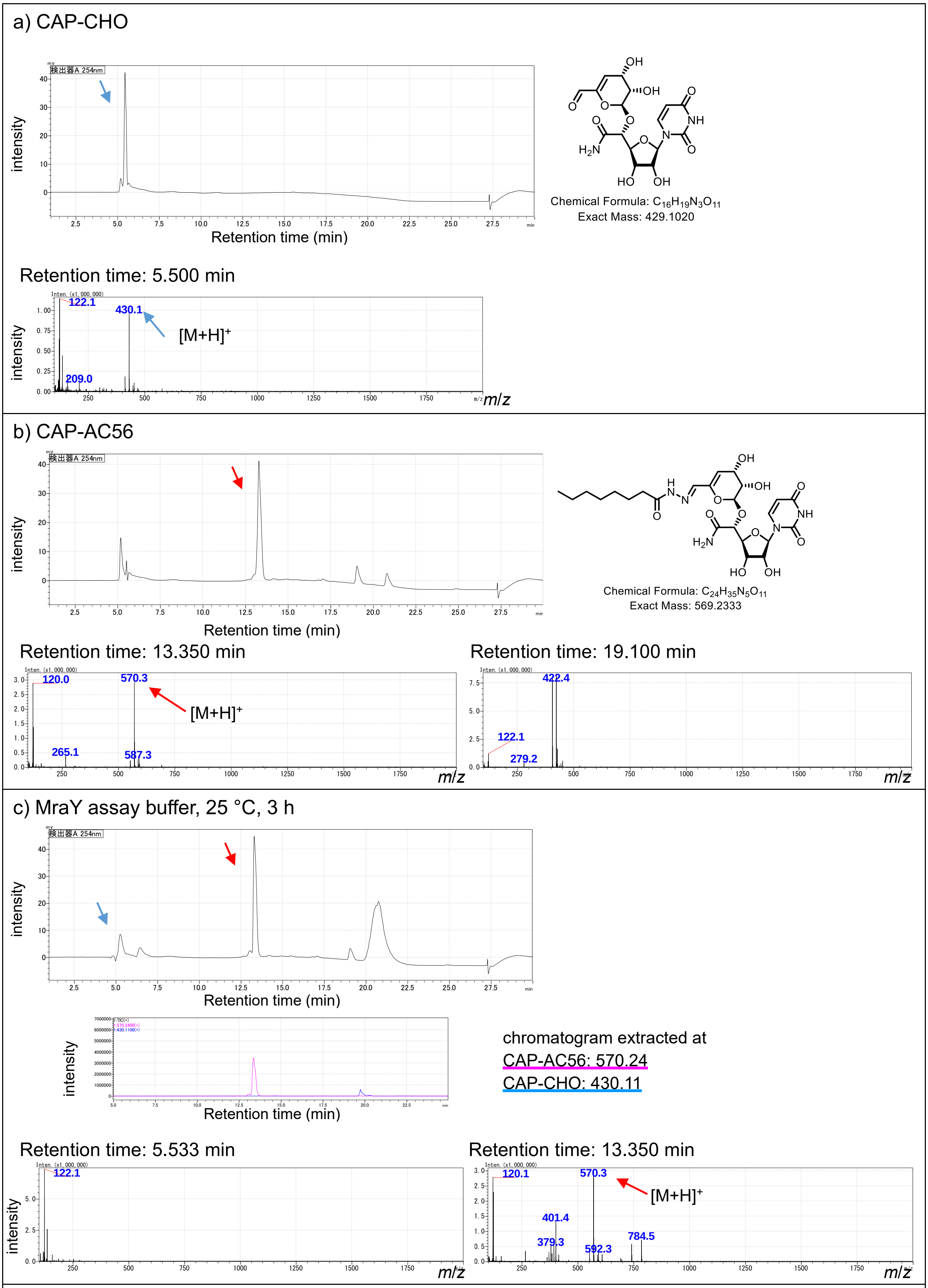

Supplementary Fig. 7. Analyses of hydrazone formation and stability in assay conditions (CAP-type).

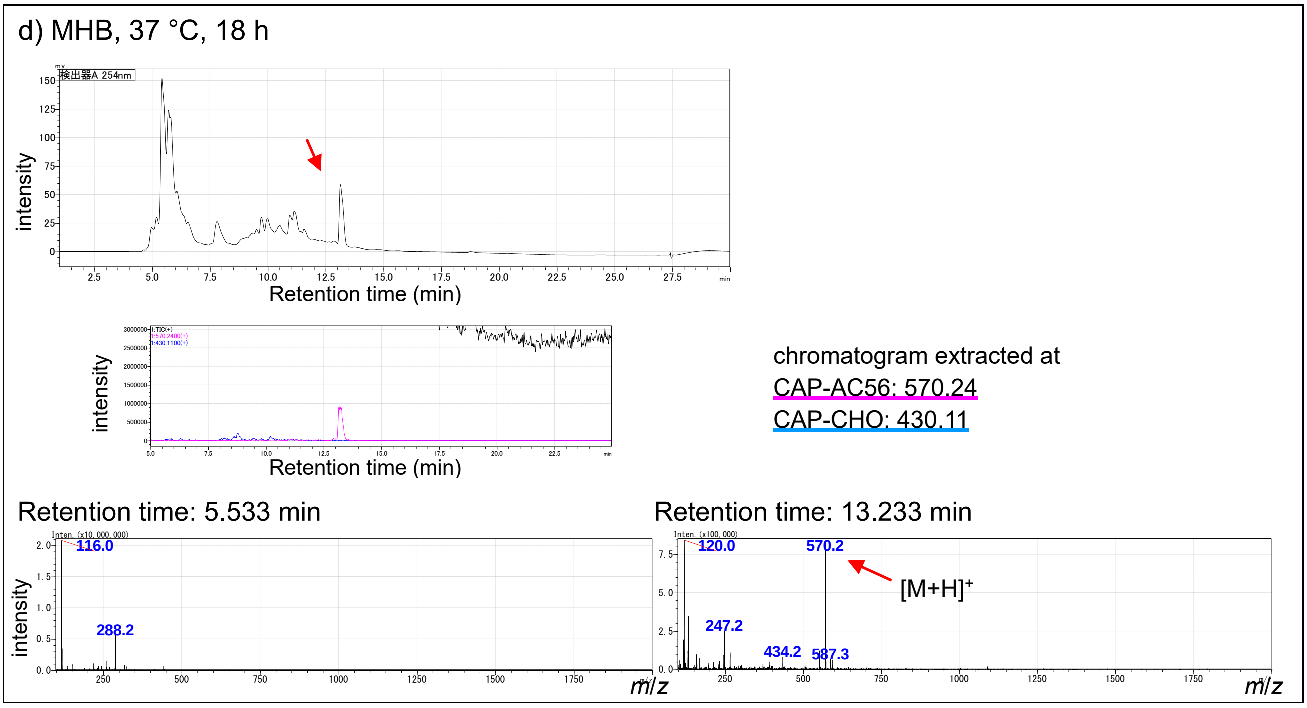

Supplementary Fig. 7. continued.

a) A peak at 5.5 minutes were identical to CAP-CHO (cyan arrow). b) A peak at 13.4 minutes were identical to CAP-AC56 (red arrow). An unknown peak was detected at 19 and 21 minutes. c) MRDp-AC56 were stable in the MraY assay buffer at 25 °C for 3 h. The peaks derived from hydrazone (13.5-14 min.) and Triton X-100 (22.5 min.) were detected. No mass of CAP-CHO was detected in the peak with the same retention time as the CAP-CHO. d) The CAP-CHO peak overlapped the peaks derived from the broth. The mass peak of aldehyde oxidized carboxylic acid was not detected.

MRYp-BZ1

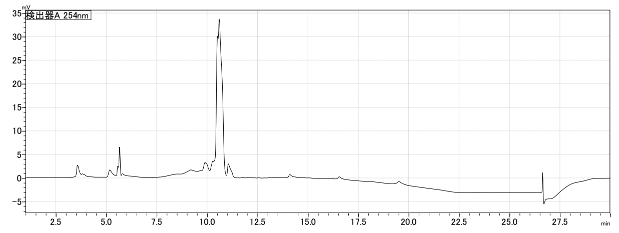

MRYm-BZ2

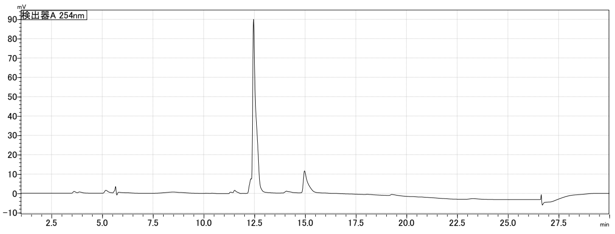

TUNp-BZ3

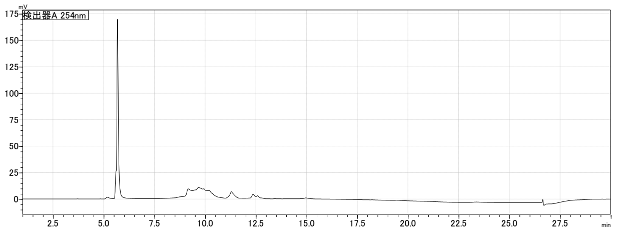

TUNm-BZ4

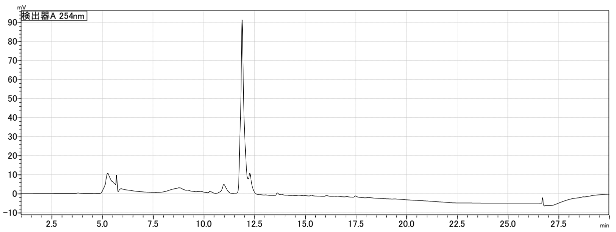

MRDp-BZ5

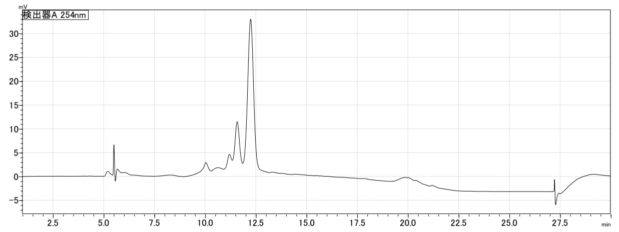

MRDp-BZ6

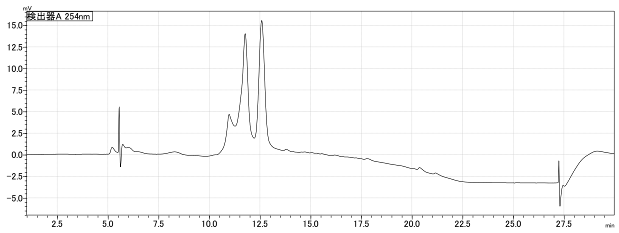

CAP-BZ7

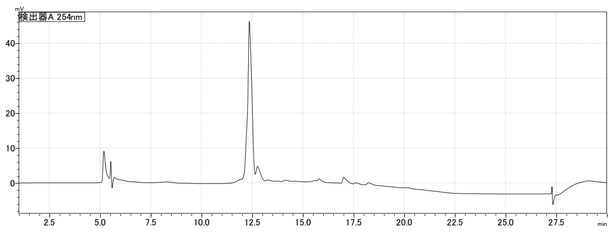

MRYp-BZ8

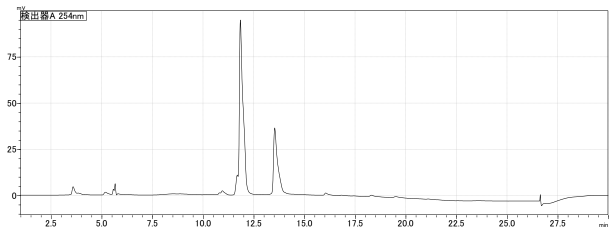

MRYm-BZ9

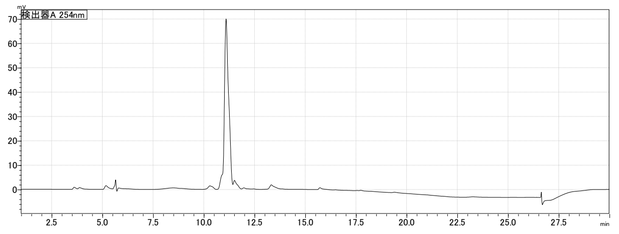

TUNp-BZ10

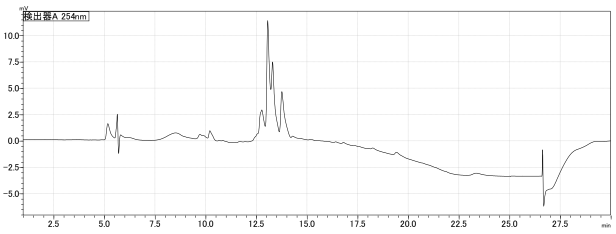

TUNm-BZ11

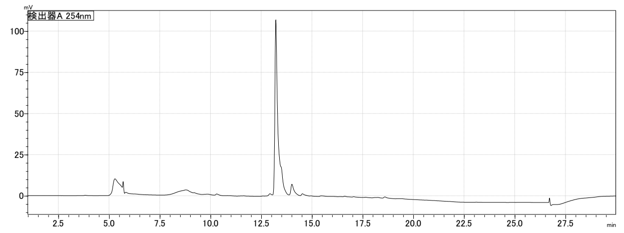

MRDp-BZ12

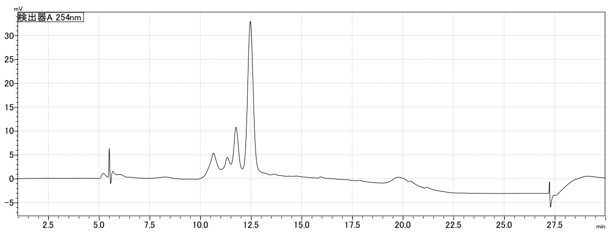

Supplementary Fig. 8. LC-MS analysis of the library (excerpts).

MRDm-BZ13

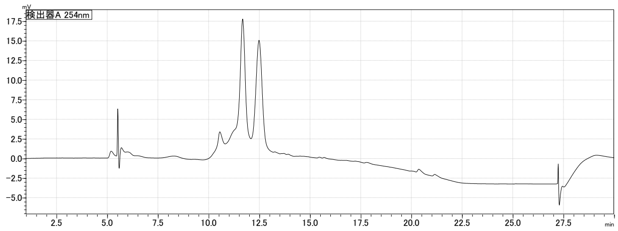

CAP-BZ14

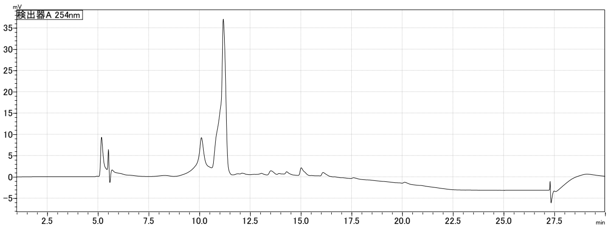

MRYp-BZ15

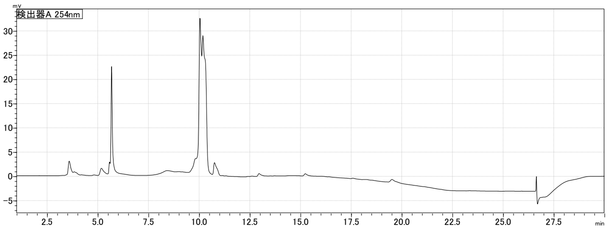

MRYm-BZ16

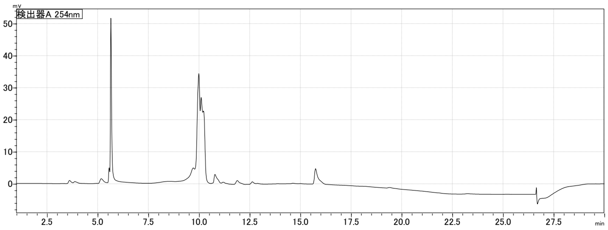

TUNp-BZ17

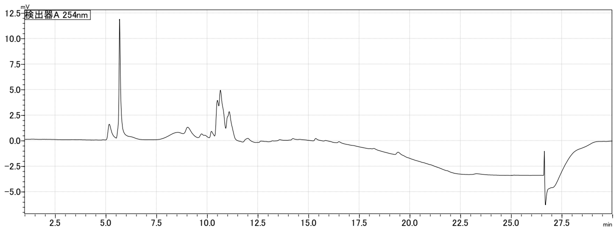

TUNm-BZ18

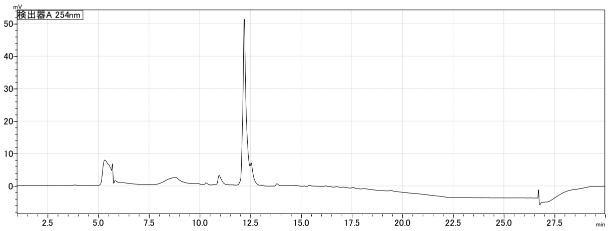

MRDp-BZ19

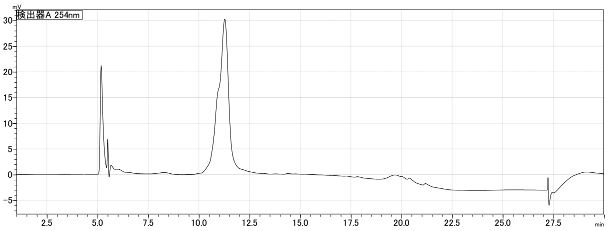

MRDm-BZ20

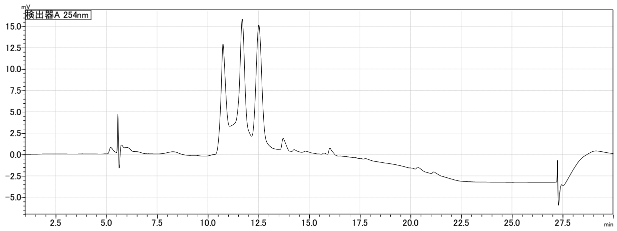

CAP-BZ21

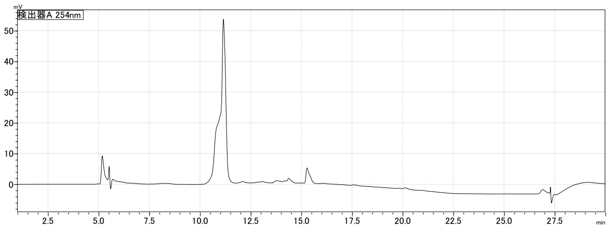

MRYp-BZ22

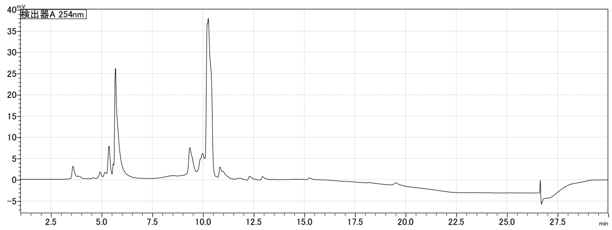

MRYm-BZ23

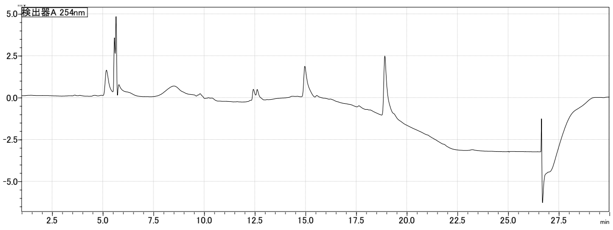

TUNp-BZ24

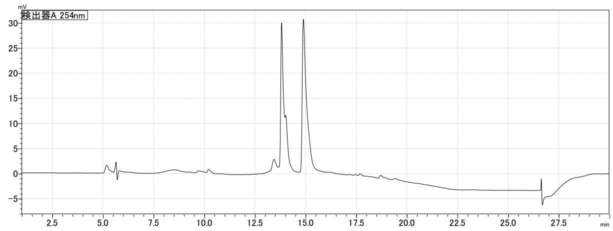

Supplementary Fig. 8. Continued.

TUNm-BZ25

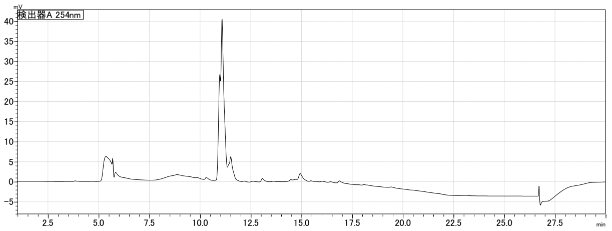

MRDp-BZ26

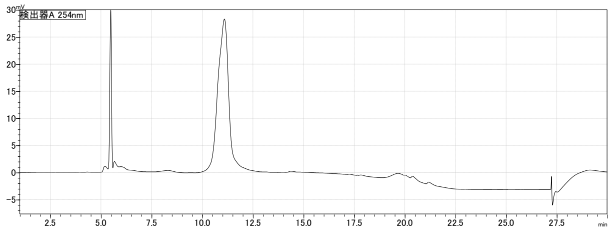

MRDm-BZ27

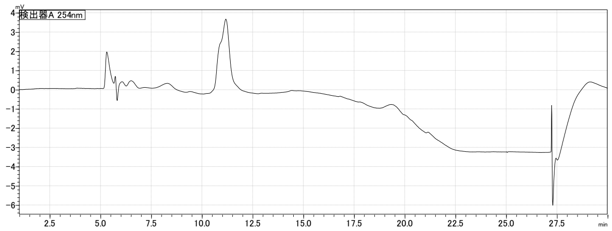

CAP-BZ28

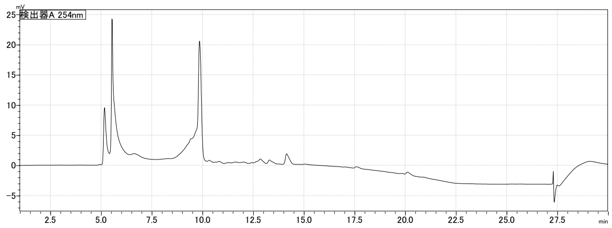

MRYp-BZ29

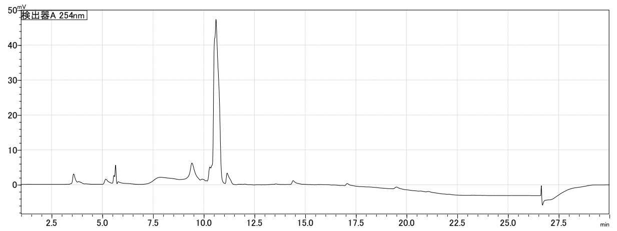

MRYm-BZ30

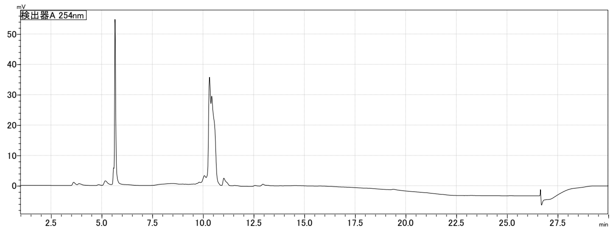

TUNp-BZ31

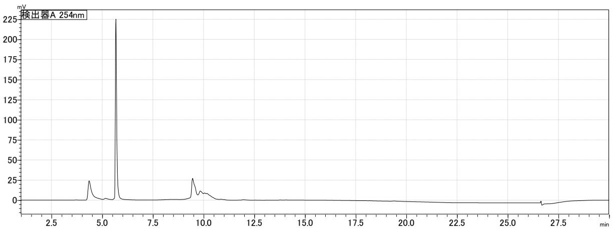

TUNm-BZ32

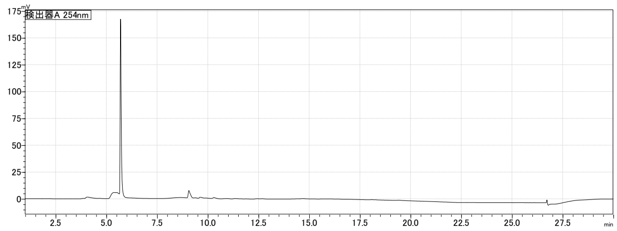

MRDp-BZ33

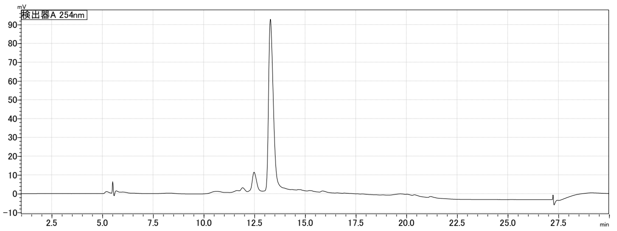

MRDm-BZ34

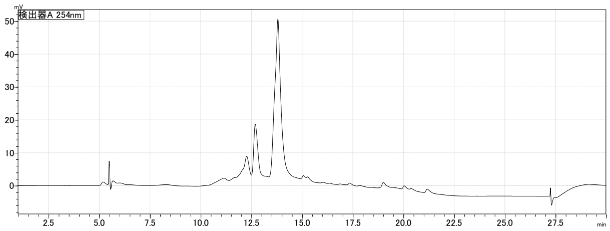

CAP-BZ35

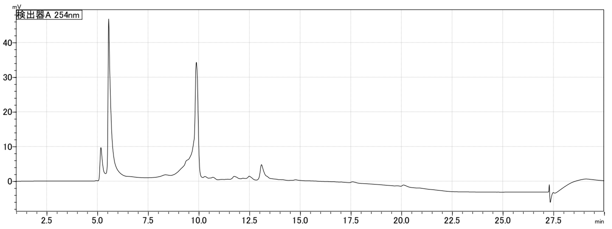

MRYp-BZ36

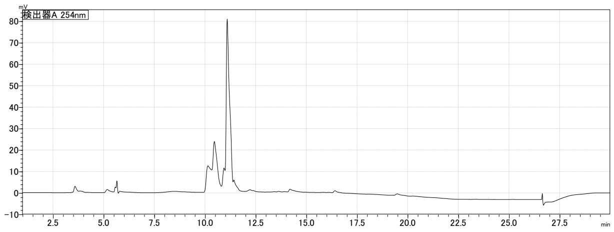

Supplementary Fig. 8. Continued.

MRYm-BZ37

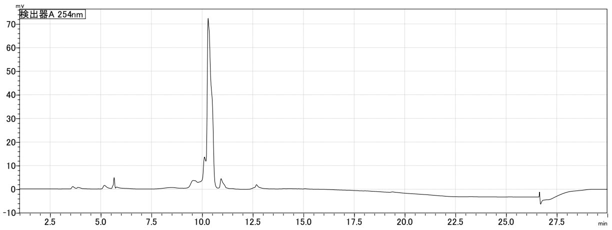

TUNp-BZ38

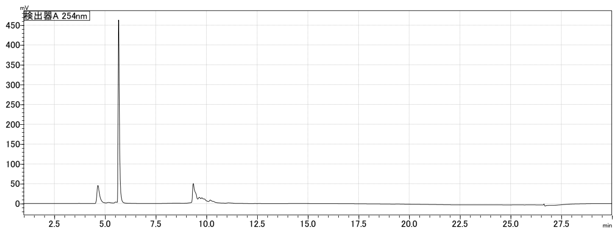

TUNm-BZ39

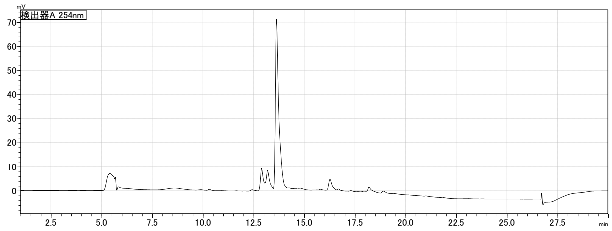

MRDp-BZ40

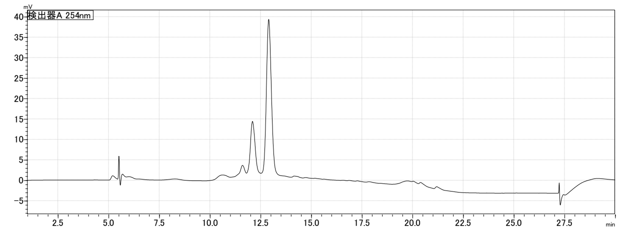

MRDm-BZ41

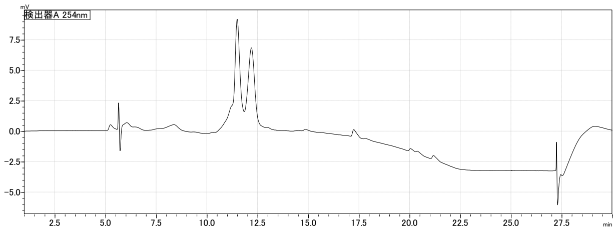

CAP-BZ42

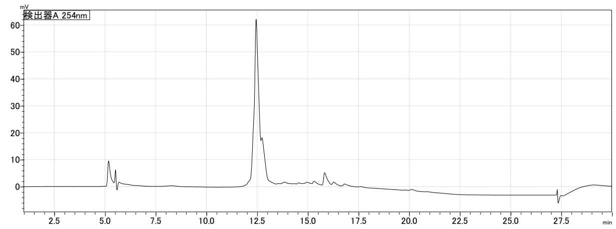

MRYp-BZ43

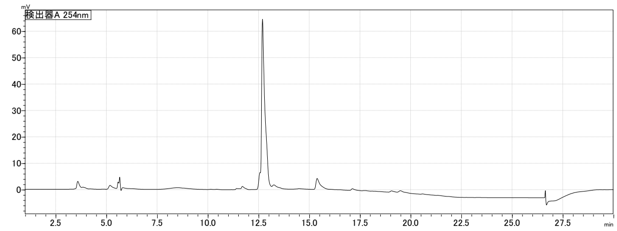

MRYm-BZ44

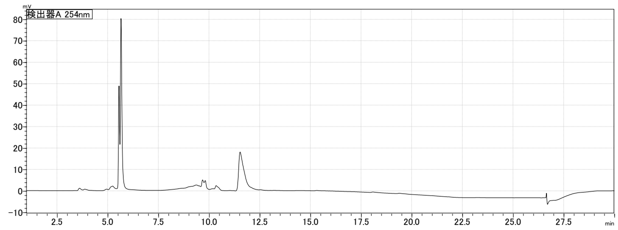

TUNp-BZ45

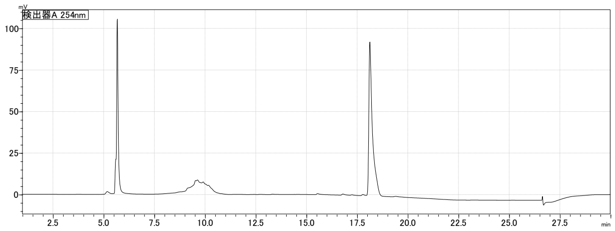

TUNm-BZ46

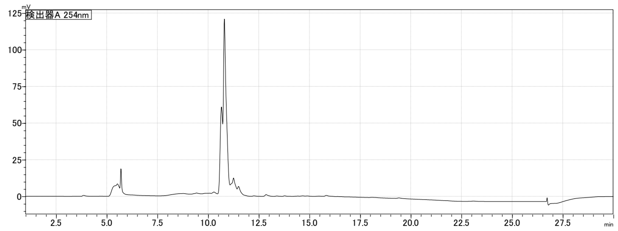

MRDp-BZ47

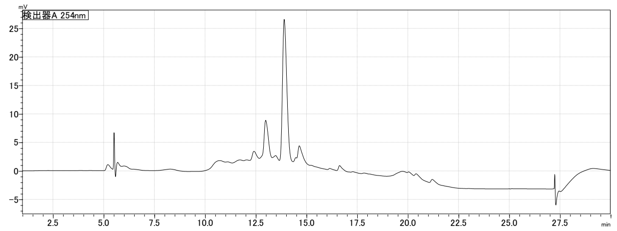

MRDm-PA48

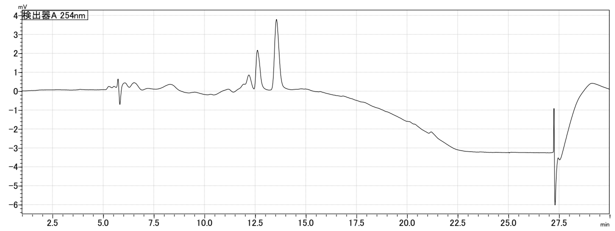

Supplementary Fig. 8. Continued.

CAP-PA49

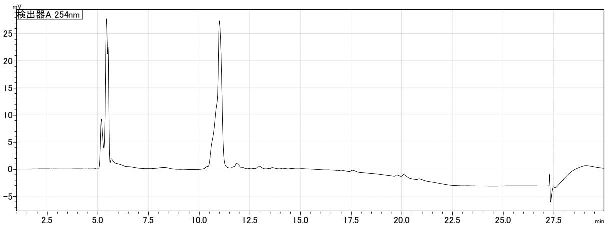

MRYp-PA50

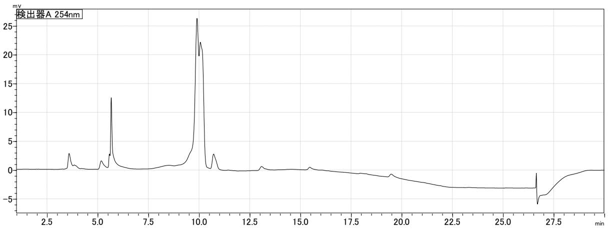

MRYm-PA51

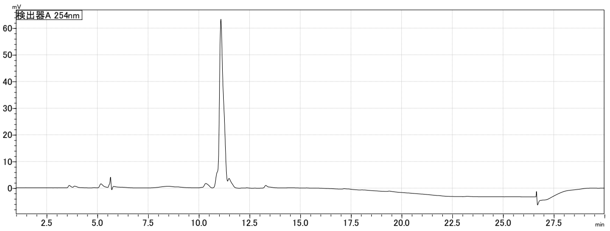

TUNp-PA52

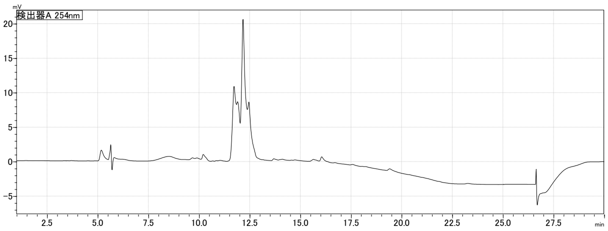

TUNm-PA53

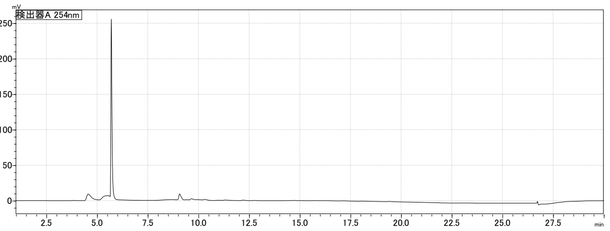

MRDp-PA54

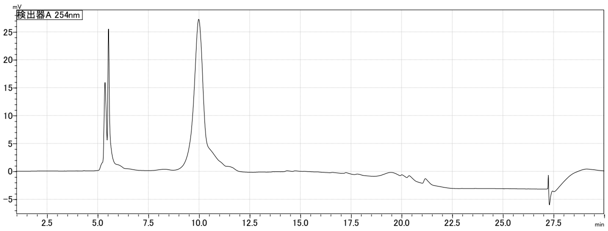

MRDm-AC55

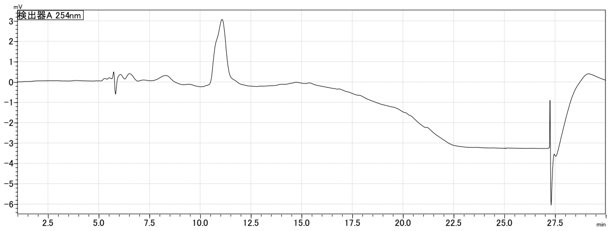

CAP-AC56

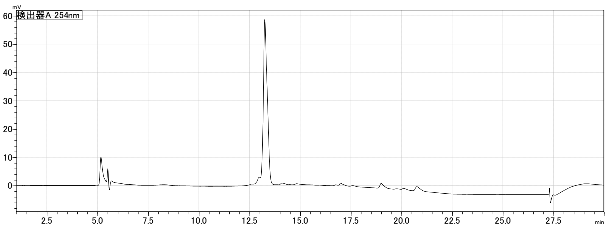

MRYp-AC57

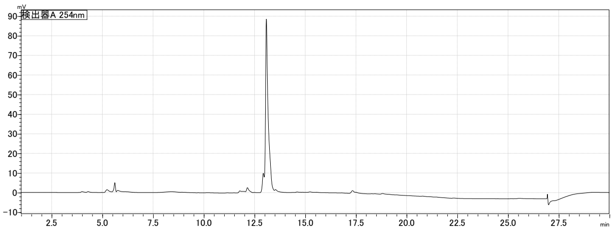

MRYm-AC58

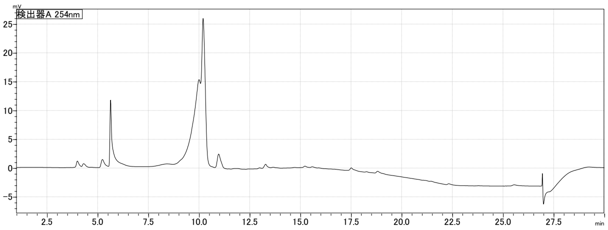

TUNp-AC59

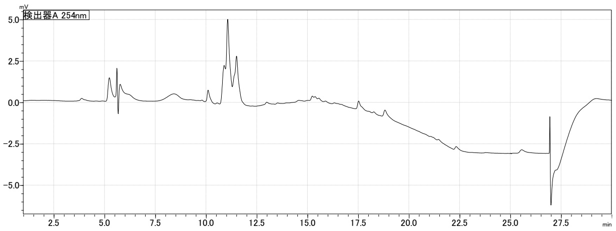

TUNm-AC60

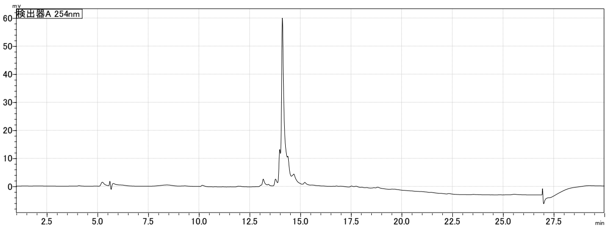

Supplementary Fig. 8. Continued.

MRDp-AC61

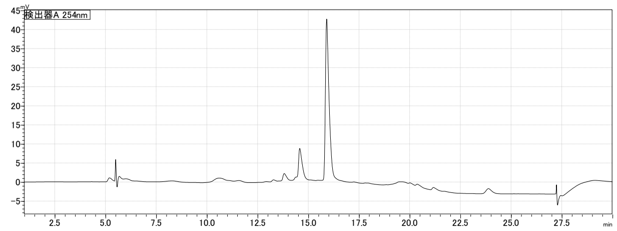

MRDm-AA62

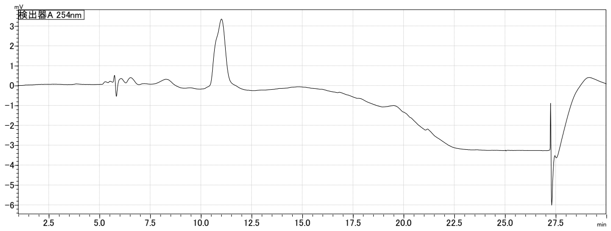

CAP-AA63

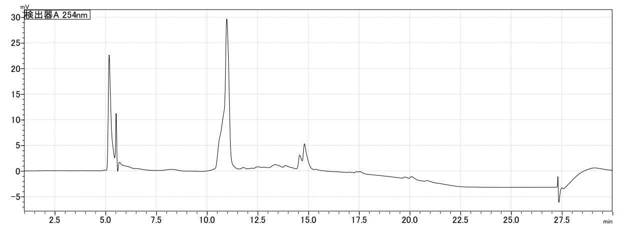

MRYp-AA64

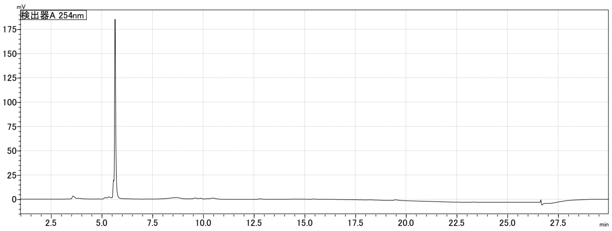

MRYm-AA65

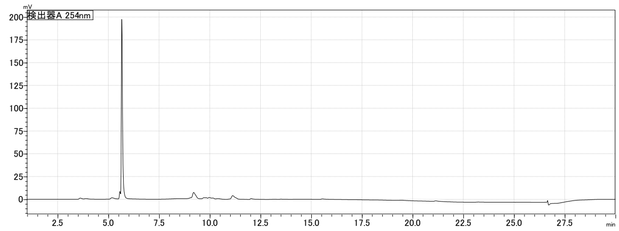

TUNp-AA66

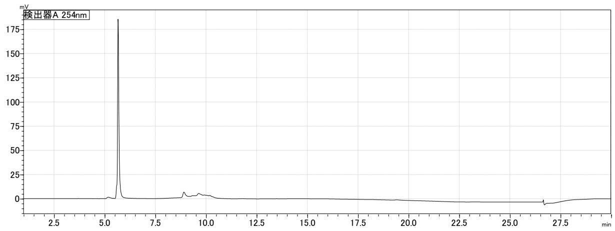

TUNm-AA67

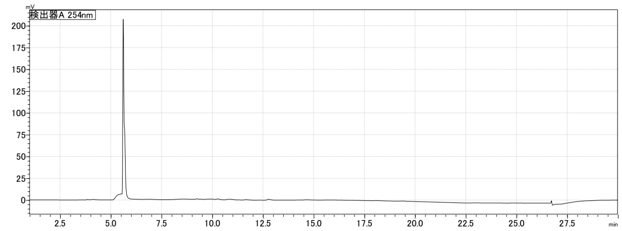

MRDp-AA68

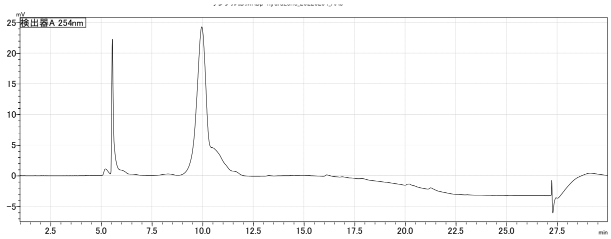

MRDm-LA69

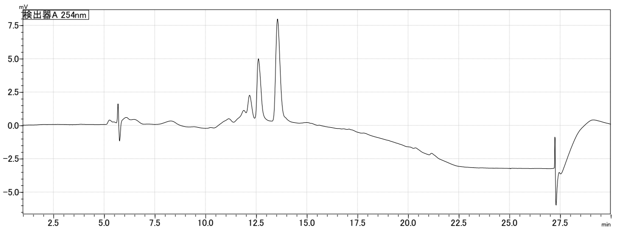

CAP-LA70

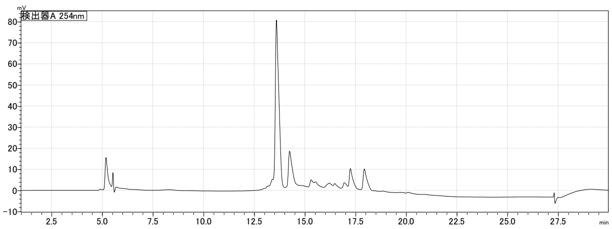

MRYp-LA71

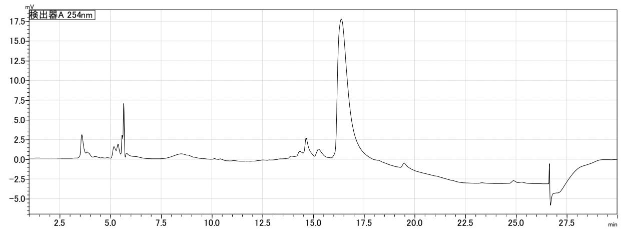

MRYm-LA72

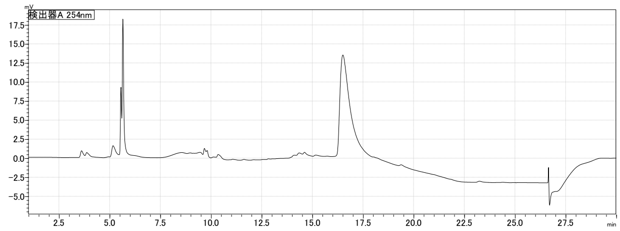

Supplementary Fig. 8. Continued.

TUNp-LA73

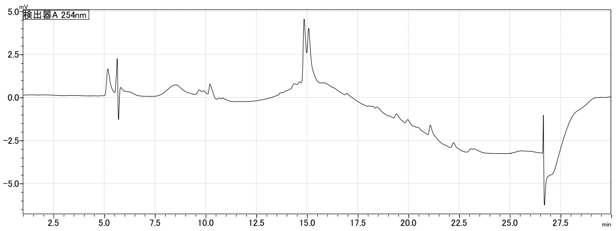

TUNm-LA74

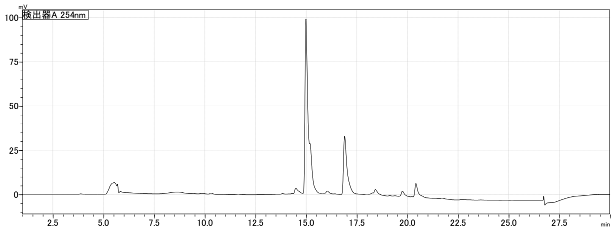

MRDp-LA75

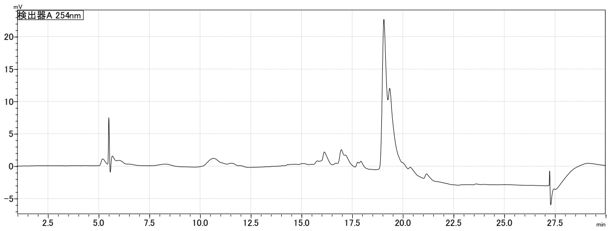

MRDm-LA76

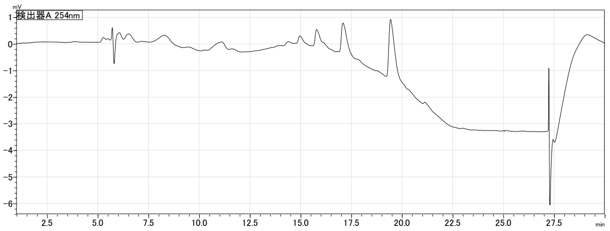

CAP-LA77

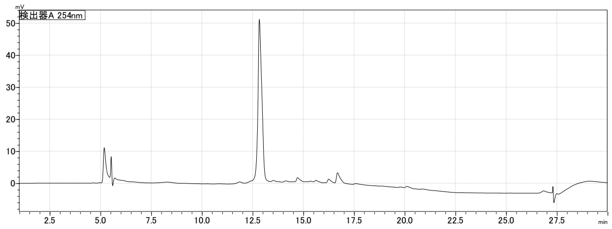

MRYp-LA78

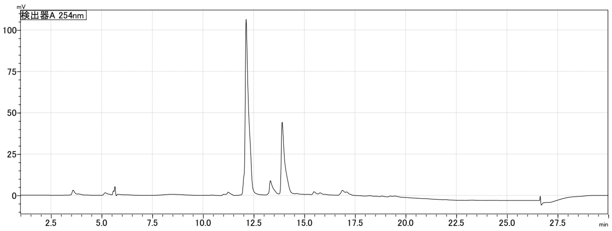

MRYm-LA79

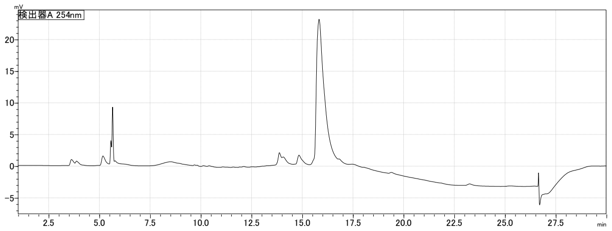

TUNp-LA80

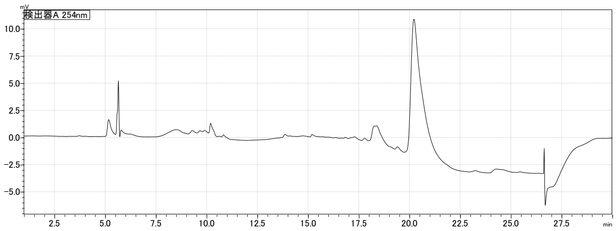

TUNm-LA81

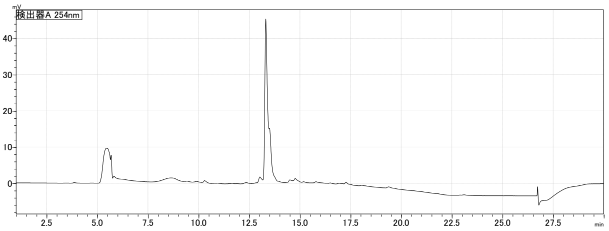

MRDp-LA82

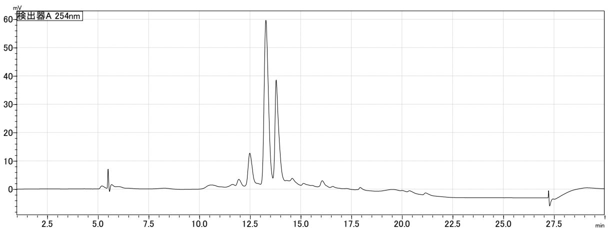

MRDm-LA83

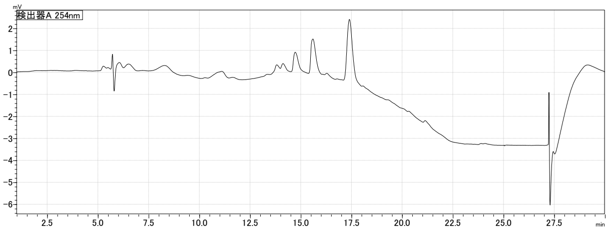

CAP-LA84

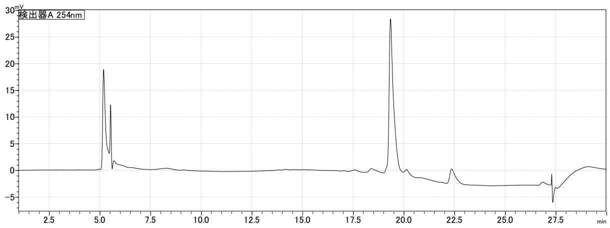

Supplementary Fig. 8. Continued.

MRYp-LA85

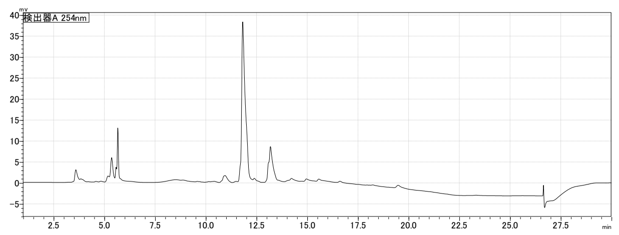

MRYm-LA86

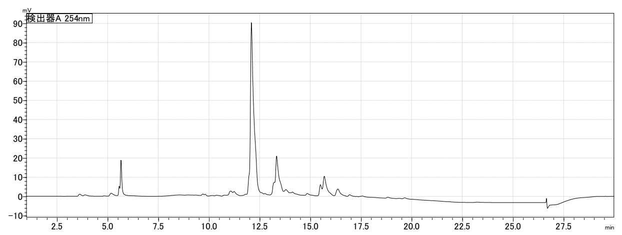

TUNp-LA87

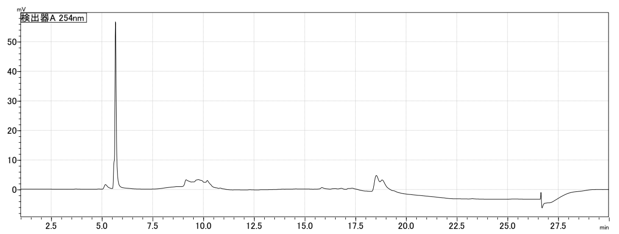

TUNm-LA88

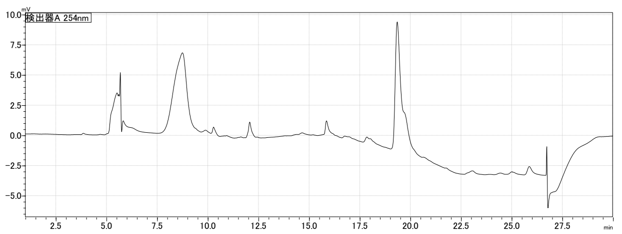

MRDp-LA89

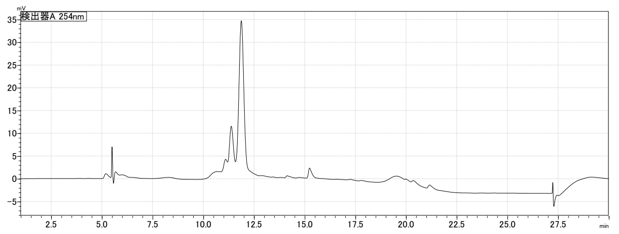

MRDm-LA90

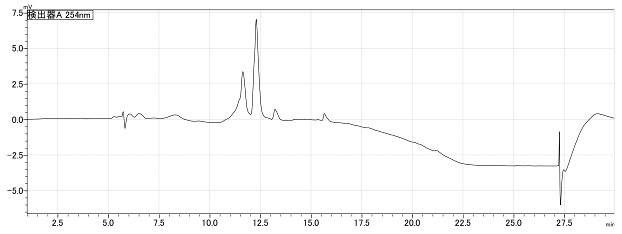

CAP-LA91

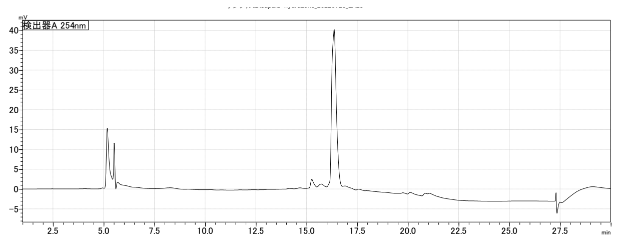

MRYp-LA92

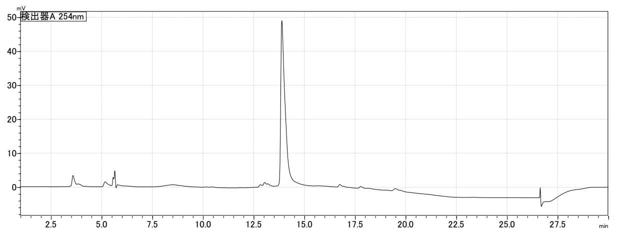

MRYm-LA93

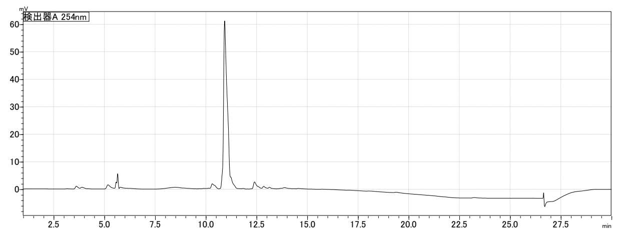

TUNp-LA94

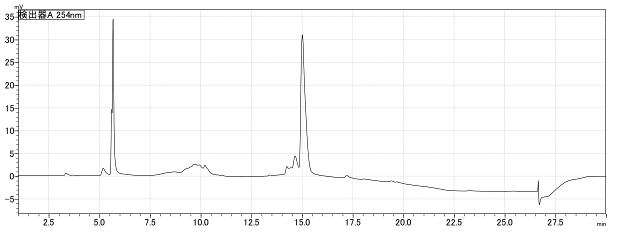

TUNm-LA95

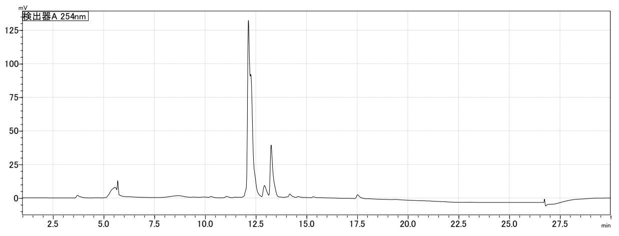

MRDp-LA96

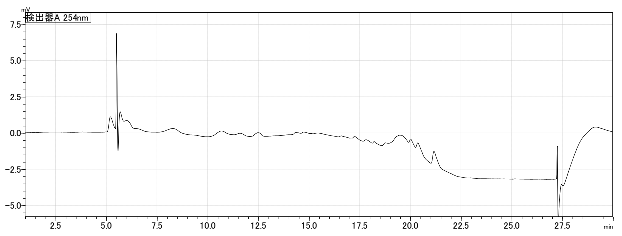

Supplementary Fig. 8. Continued.

MRDm-LA97	CAP-LA98
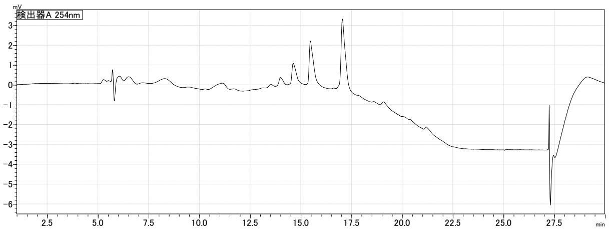	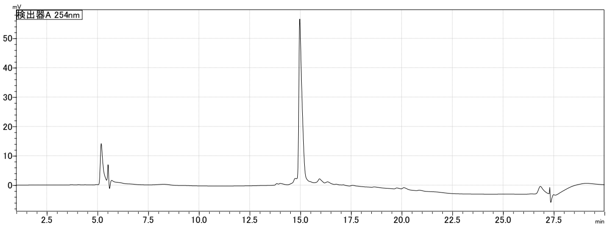

Supplementary Fig. 8. Continued.

conditions: LC-MS; 5% to 90% MeCN in 0.1% *aq.* HCO_2H gradient elusion.

Experimental details are shown in methods section of the main text.

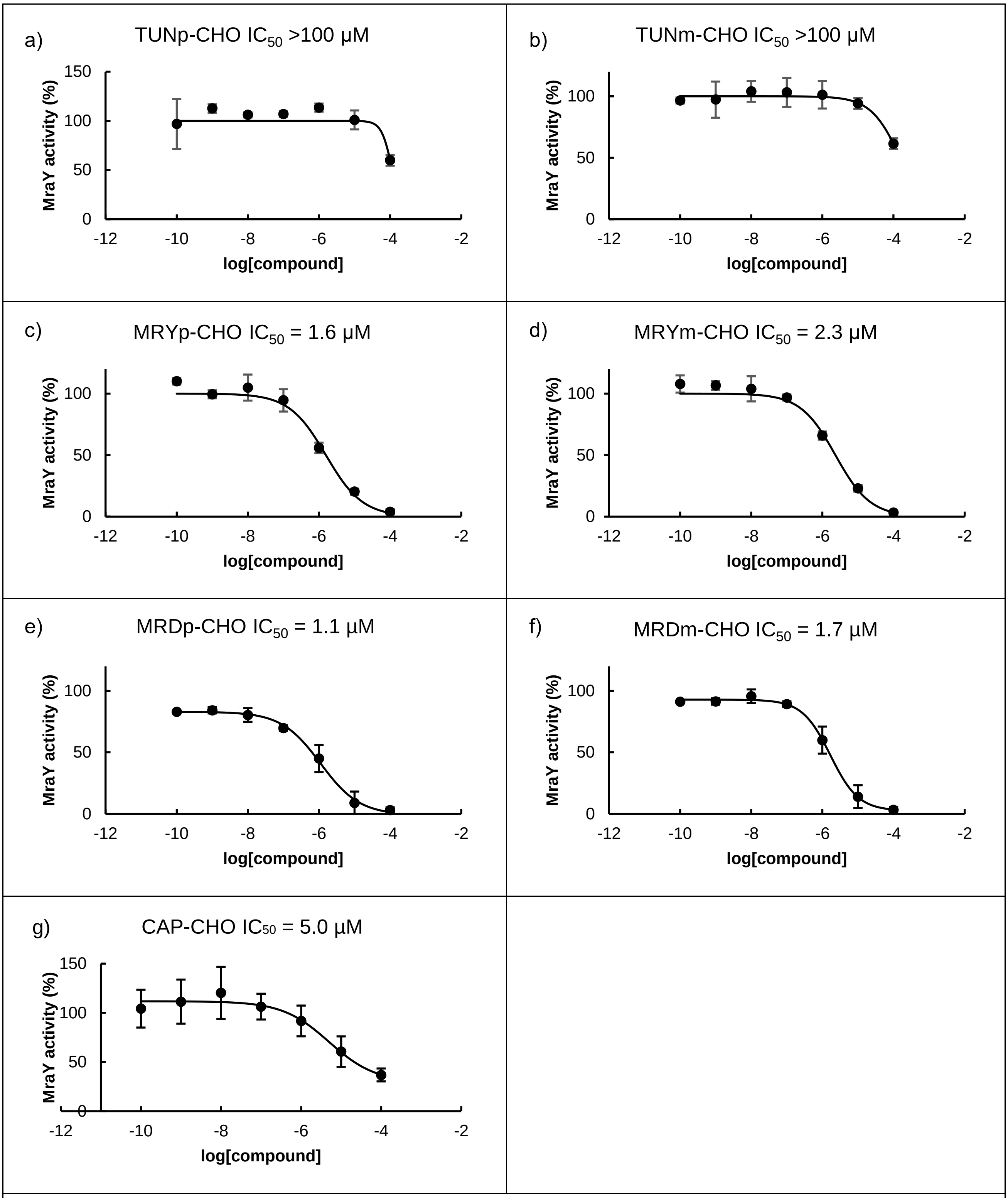

Supplementary Fig. 9. MraY inhibitory activity of core aldehydes.

Conditions: Reactions were carried out in a 384-well microplate. A solution containing 10 μM dansylated - UDP-MurNAc-pentapeptide and 50 μM undecaprenyl phosphate (C_{55}-P) in 20 μL of an assay buffer [50 mM Tris-HCl (pH 7.6), 50 mM KCl, 25 mM $MgCl_2$, 0.2% Triton X-100 and 8% glycerol] was prepered. The reactions were initiated by the addition of *S. aureus* MraY enzyme (5 μL, 11 μg/mL). After 3 h of incubation at room temperature, the formation of dansylated lipid I was monitored by fluorescence enhancement (excitation at

355 nm, emission at 535 nm). The mixtures contained 2% DMSO in order to increase the solubility of the compounds (concentrations; 0.0001, 0.001, 0.01, 0.1, 1, 10, 100 μM). This data was collected from three independent experiments (n=3).

frag	*Ef* ATCC 35667	*Sa* ATCC 25923	*Pa* ATCC 27853	*Kp* ATCC 13883	*Ent* ATCC 13047	*Ab* ATCC 19606	*Ec* ATCC 25922
BZ1	>100	>100	>100	>100	>100	>100	>100
BZ2	>100	>100	>100	>100	>100	>100	>100
BZ3	>100	>100	>100	>100	>100	>100	>100
BZ4	>100	>100	>100	>100	>100	>100	>100
BZ5	>100	>100	>100	>100	>100	>100	>100
BZ6	>100	>100	>100	>100	>100	>100	>100
BZ7	>100	>100	>100	>100	>100	>100	>100
BZ8	>100	>100	>100	>100	>100	>100	>100
BZ9	>100	>100	>100	>100	>100	>100	>100
BZ10	>100	>100	>100	>100	>100	>100	>100
BZ11	>100	>100	>100	>100	>100	>100	>100
BZ12	>100	>100	>100	>100	>100	>100	>100
BZ13	>100	>100	>100	>100	>100	>100	>100
BZ14	>100	>100	>100	>100	>100	>100	>100
BZ15	>100	>100	>100	>100	>100	>100	>100
BZ16	>100	>100	>100	>100	>100	>100	>100
BZ17	>100	>100	>100	>100	>100	>100	>100
BZ18	>100	>100	>100	>100	>100	>100	>100
BZ19	>100	>100	>100	>100	>100	>100	>100
BZ20	>100	>100	>100	>100	>100	>100	>100
BZ21	>100	>100	>100	>100	>100	>100	>100
BZ22	>100	>100	>100	>100	>100	>100	>100
BZ23	>100	>100	>100	>100	>100	>100	>100
BZ24	>100	>100	>100	>100	>100	>100	>100
BZ25	>100	>100	>100	>100	>100	>100	>100
BZ26	>100	>100	>100	>100	>100	>100	>100
BZ27	>100	>100	>100	>100	>100	>100	>100
BZ28	>100	>100	>100	>100	>100	>100	>100
BZ29	>100	>100	>100	>100	>100	>100	>100
BZ30	>100	>100	>100	>100	>100	>100	>100
BZ31	>100	>100	>100	>100	>100	>100	>100
BZ32	>100	>100	>100	>100	>100	>100	>100
BZ33	>100	>100	>100	>100	>100	>100	>100
BZ34	>100	>100	>100	>100	>100	>100	>100
BZ35	>100	>100	>100	>100	>100	>100	>100
BZ36	>100	>100	>100	>100	>100	>100	>100
BZ37	>100	>100	>100	>100	>100	>100	>100
BZ38	>100	>100	>100	>100	>100	>100	>100
BZ39	>100	>100	>100	>100	>100	>100	>100
BZ40	>100	>100	>100	>100	>100	>100	>100
BZ41	>100	>100	>100	>100	>100	>100	>100
BZ42	>100	>100	>100	>100	>100	>100	>100
BZ43	>100	>100	>100	>100	>100	>100	>100
BZ44	>100	>100	>100	>100	>100	>100	>100
BZ45	>100	>100	>100	>100	>100	>100	>100
BZ46	>100	>100	>100	>100	>100	>100	>100
BZ47	>100	>100	>100	>100	>100	>100	>100
PA48	>100	>100	>100	>100	>100	>100	>100
PA49	>100	>100	>100	>100	>100	>100	>100
PA50	>100	>100	>100	>100	>100	>100	>100
PA51	>100	>100	>100	>100	>100	>100	>100
PA52	>100	>100	>100	>100	>100	>100	>100
PA53	>100	>100	>100	>100	>100	>100	>100
PA54	>100	>100	>100	>100	>100	>100	>100
AC55	>100	>100	>100	>100	>100	>100	>100
AC56	>100	>100	>100	>100	>100	>100	>100
AC57	>100	>100	>100	>100	>100	>100	>100
AC58	>100	>100	>100	>100	>100	>100	>100
AC59	>100	>100	>100	>100	>100	>100	>100
AC60	>100	>100	>100	>100	>100	>100	>100
AC61	>100	>100	>100	>100	>100	>100	>100
AA62	>100	>100	>100	>100	>100	>100	>100
AA63	>100	>100	>100	>100	>100	>100	>100
AA64	>100	>100	>100	>100	>100	>100	>100
AA65	>100	>100	>100	>100	>100	>100	>100
AA66	>100	>100	>100	>100	>100	>100	>100
AA67	>100	>100	>100	>100	>100	>100	>100
AA68	>100	>100	>100	>100	>100	>100	>100
LA69	>100	>100	>100	>100	>100	>100	>100
LA70	>100	>100	>100	>100	>100	>100	>100
LA71	>100	>100	>100	>100	>100	>100	>100
LA72	>100	>100	>100	>100	>100	>100	>100
LA73	>100	>100	>100	>100	>100	>100	>100
LA74	>100	>100	>100	>100	>100	>100	>100
LA75	>100	>100	>100	>100	>100	>100	>100
LA76	>100	>100	>100	>100	>100	>100	>100
LA77	>100	>100	>100	>100	>100	>100	>100
LA78	>100	>100	>100	>100	>100	>100	>100
LA79	>100	>100	>100	>100	>100	>100	>100
LA80	>100	>100	>100	>100	>100	>100	>100
LA81	>100	>100	>100	>100	>100	>100	>100
LA82	>100	>100	>100	>100	>100	>100	>100
LA83	50	>100	>100	>100	>100	>100	>100
LA84	>100	>100	>100	>100	>100	>100	>100
LA85	>100	>100	>100	>100	>100	>100	>100
LA86	>100	>100	>100	>100	>100	>100	>100
LA87	>100	>100	>100	>100	>100	>100	>100
LA88	>100	>100	>100	>100	>100	>100	>100
LA89	>100	>100	>100	>100	>100	>100	>100
LA90	>100	>100	>100	>100	>100	>100	>100
LA91	50	50	>100	50	100	50	50
LA92	50	50	>100	>100	>100	>100	>100
LA93	>100	>100	>100	>100	>100	>100	>100
LA94	50	50	>100	>100	>100	>100	>100
LA95	>100	50	>100	>100	>100	>100	>100
LA96	50	50	>100	>100	>100	>100	>100
LA97	>100	>100	>100	>100	>100	>100	>100
LA98	50	50	50	>100	50	>100	50

LA83

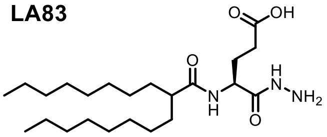

LA91

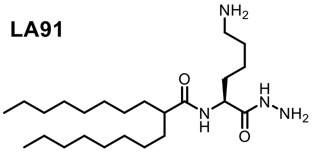

LA92

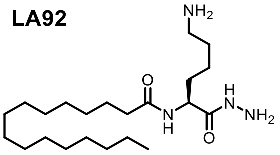

LA94

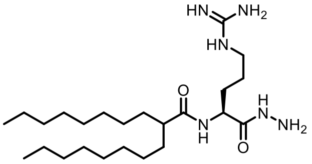

LA95

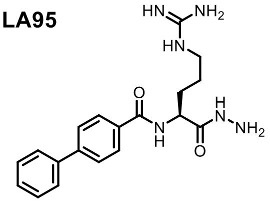

LA96

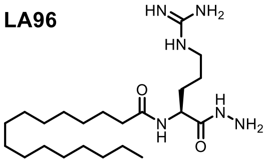

LA98

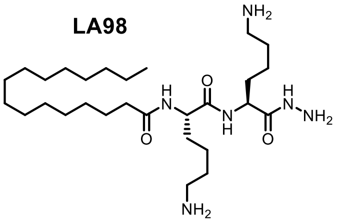

Supplementary Fig. 10. Antibacterial activity of fragments.

E. faecium ATCC 35667, *S. aureus* ATCC 29213, *P. aeruginosa* ATCC 27853, *K. pneumoniae* ATCC 13883, *E. coli* ATCC 25922, *E. cloacae* ATCC 13047, *A. baumannii* ATCC 19606.

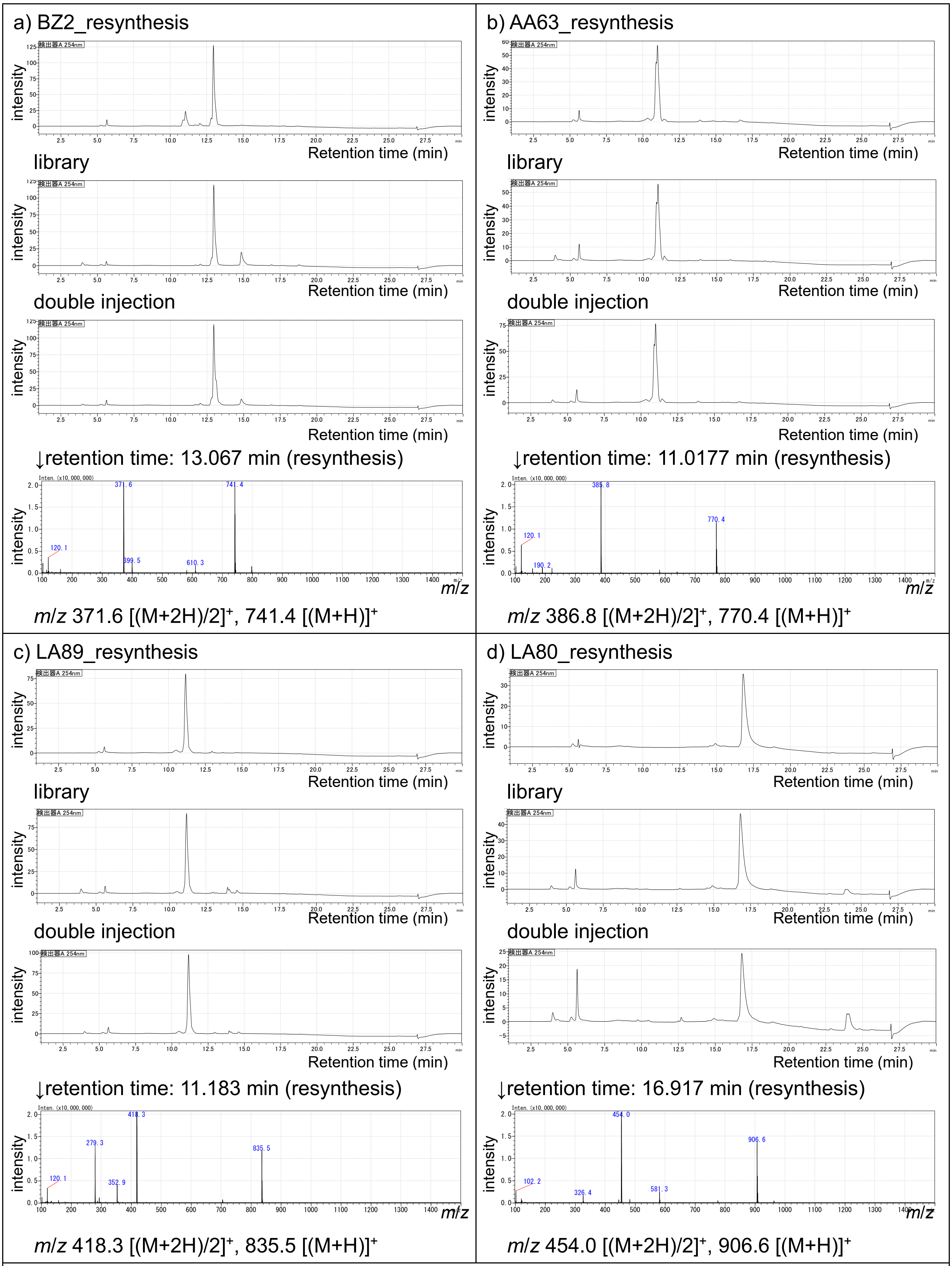

Supplementary Fig. 11. LC-MS analyses of re-synthesized hydrazones.

e) LA92_resynthesis

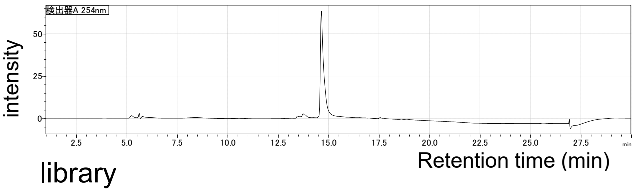

library

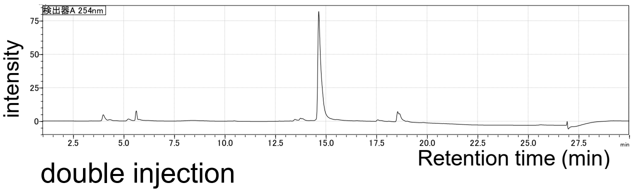

double injection

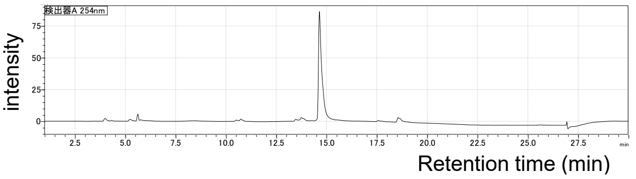

↓retention time: 14.750 min (resynthesis)

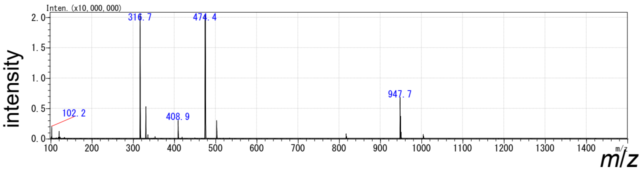

m/z 316.7 [(M+3H)/3]⁺, 474.4 [(M+2H)/2]⁺, 947.7 [(M+H)]⁺

f) LA98_resynthesis

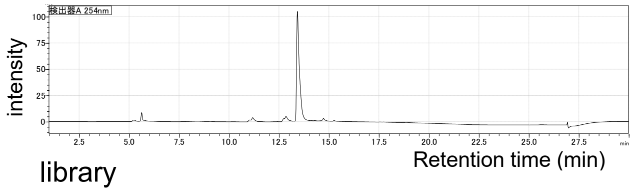

library

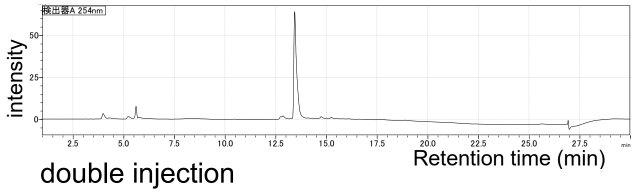

double injection

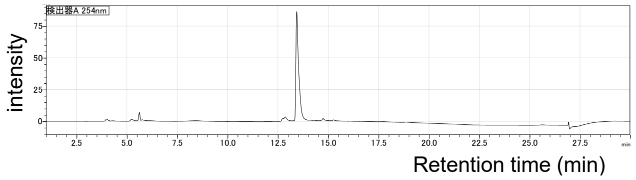

↓retention time: 13.533 min (resynthesis)

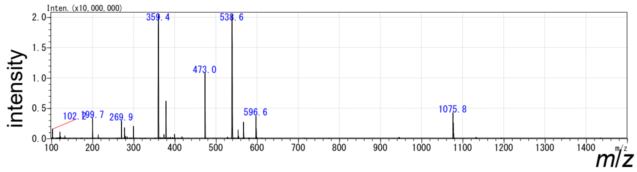

m/z 359.4 [(M+3H)/3]⁺, 538.6 [(M+2H)/2]⁺, 1075.8 [(M+H)]⁺

Supplementary Fig. 11. Continued.

conditions: LC-MS; 5% to 90% MeCN in 0.1% *aq.* HCO_2H gradient elution.

Experimental details are shown in methods section of the main text.

UV chromatograms ‘library’ are results of analysis of the library compounds in the corresponding well. All resynthesized hydrazones were identical to the main compound in the library from UV chromatograms of ‘double injection’.

^{1}H NMR spectra of MRYp-CHO (green) and hydrazone (brown). [CD_3OD, 500 MHz]

a) MRYp-BZ2

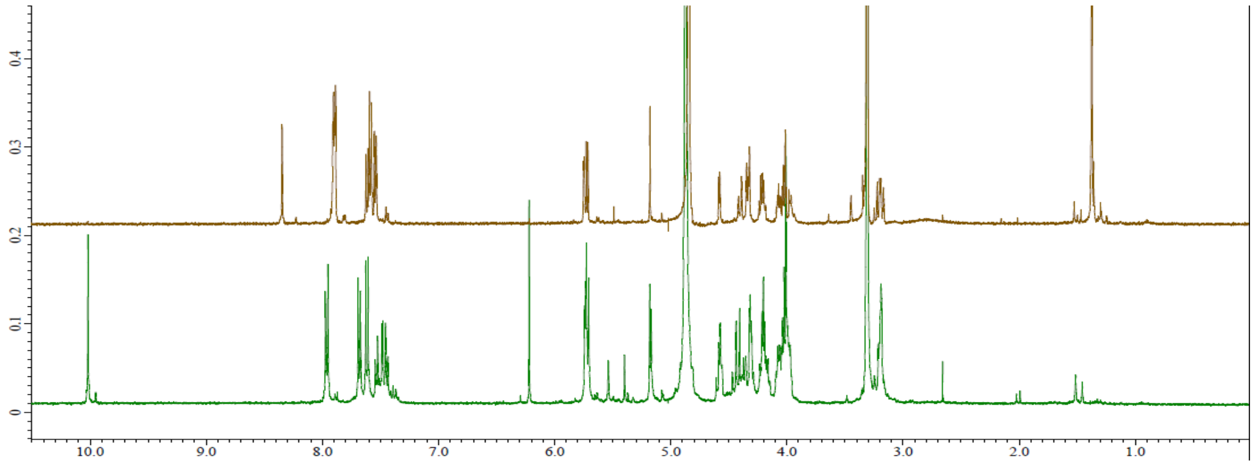

b) MRYp-AA63

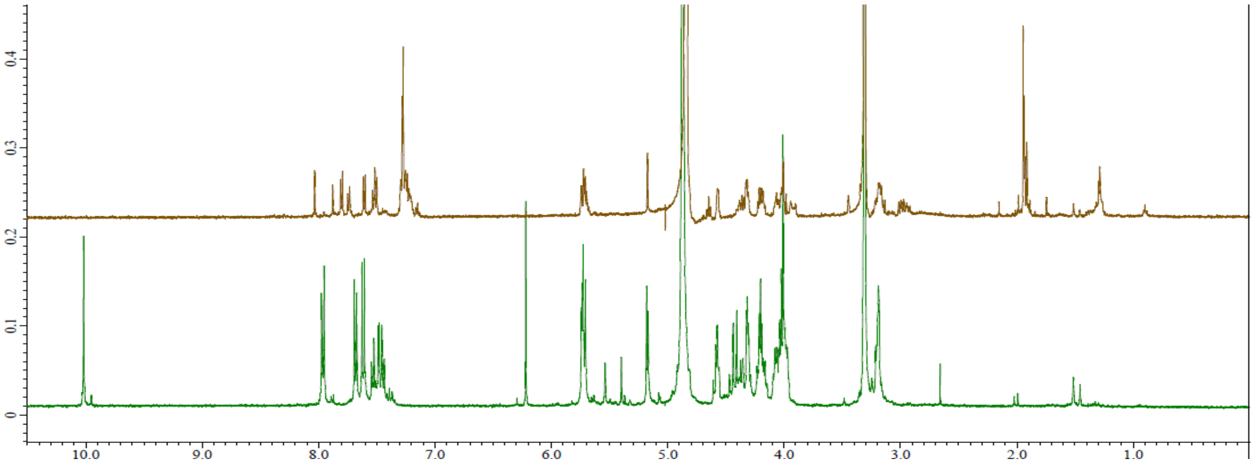

c) MRYp-LA89

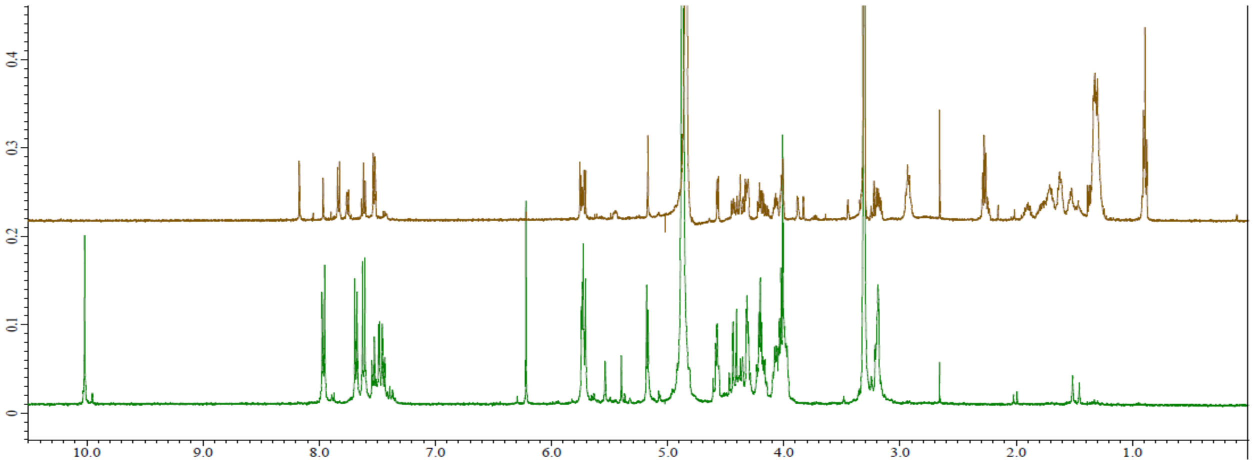

Supplementary Fig. 12. ^{1}H NMR spectra of re-synthesized hydrazones.

d) MRYp-LA80

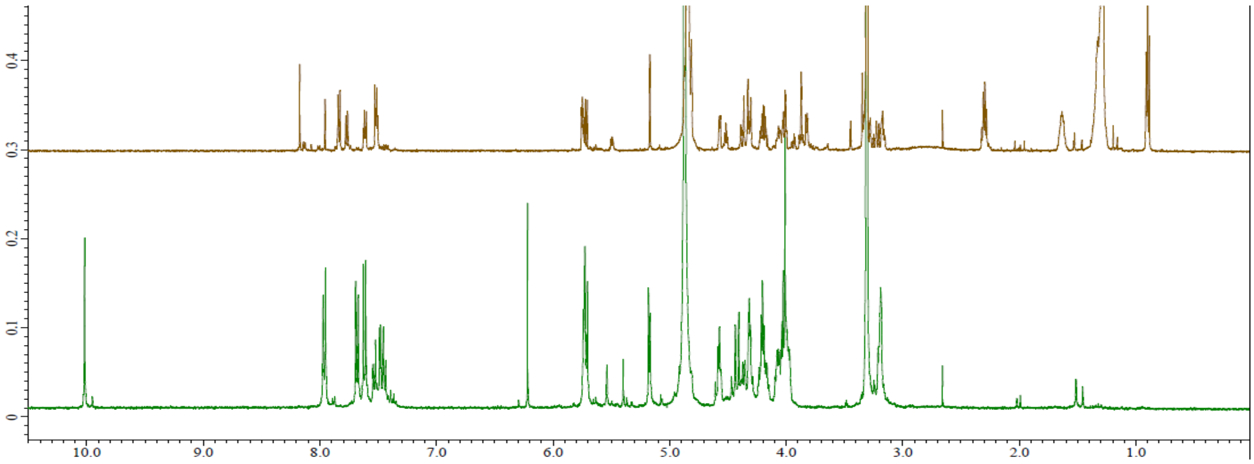

e) MRYp-LA92

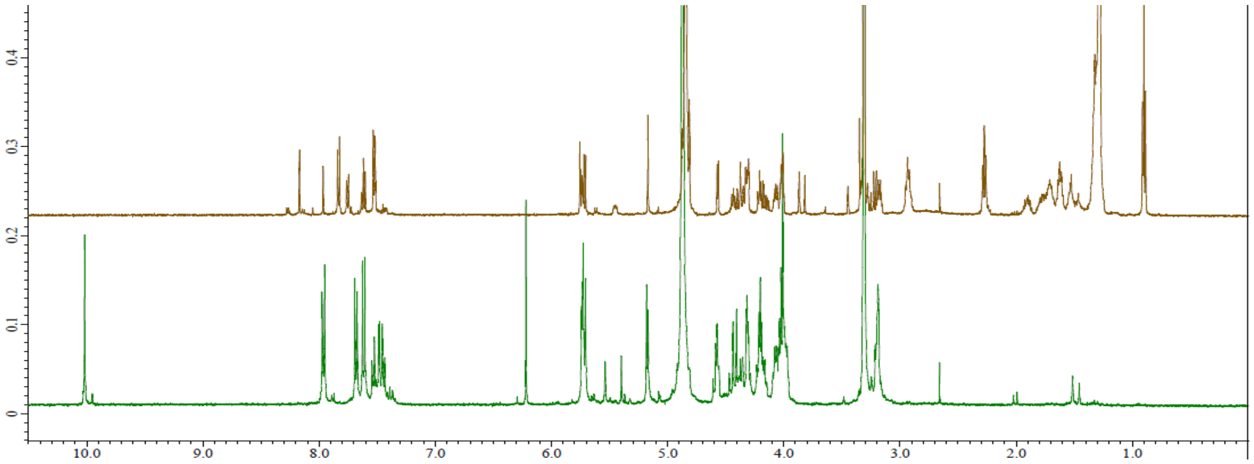

f) MRYp-LA98

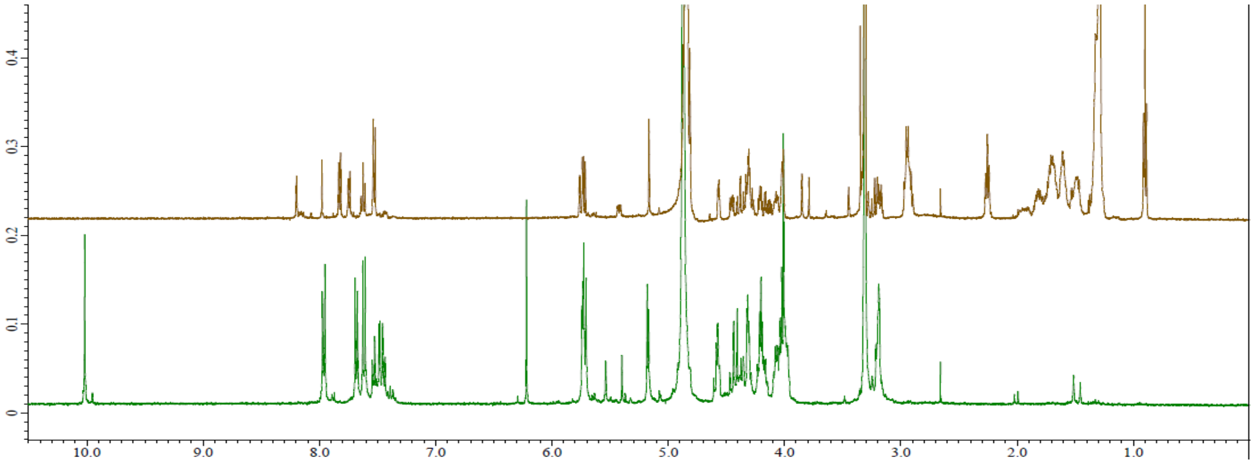

Supplementary Fig. 12. Continued.

The spectrum of aldehyde (green) is shown as a mixture of aldehyde and hydrate/hemiacetal. The peak at 10 ppm is C*H*O and the peak at 6.2 ppm is corresponding C*H*OH(OR). The structure of aldehyde is determined by ^{1}H NMR in D_2O (see preparation of compounds section). a) MRYp-BZ2 appears to exist almost exclusively as a single isomer

(8.3 ppm; C*H*=N-N). b-f) The other hydrazones derived from amino acids appear to be a mixture of *E*/*Z* isomers (8.0 and 8.1-8.2 ppm, ratio 4:6~5:5). The peak of C*H*O of aldehyde disappeared in all hydrazone spectra (brown).

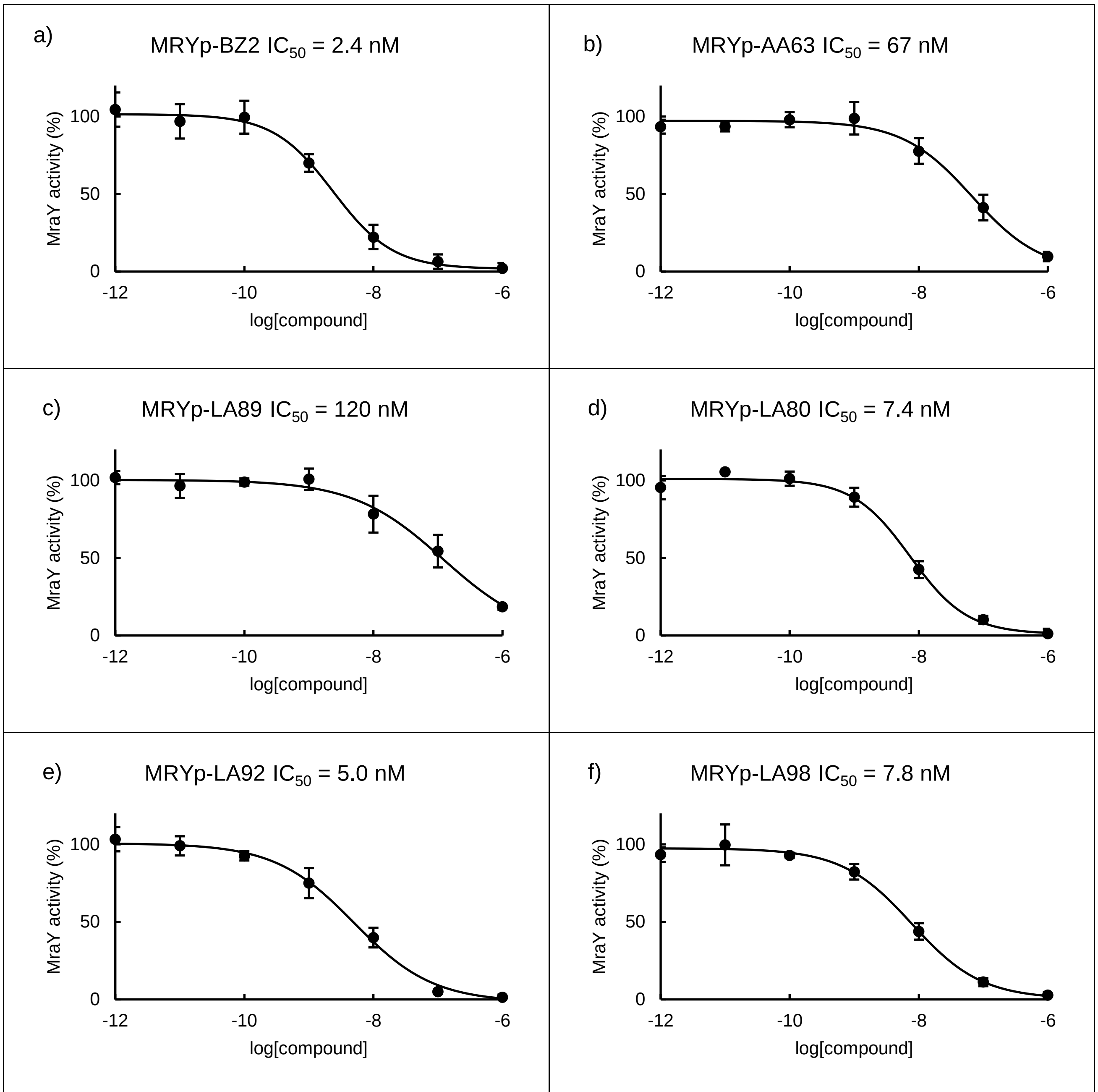

Supplementary Fig. 13. MraY inhibitory activity of re-synthesized hydrazones.

Conditions: Reactions were carried out in a 384-well microplate. A solution containing 10 μM dansylated - UDP-MurNAc-pentapeptide and 50 μM undecaprenyl phosphate (C_{55}-P) in 20 μL of an assay buffer [50 mM Tris-HCl (pH 7.6), 50 mM KCl, 25 mM $MgCl_2$, 0.2% Triton X-100 and 8% glycerol] was prepered. The reactions were initiated by the addition of *S. aureus* MraY enzyme (5 μL, 11 μg/mL). After 3 h of incubation at room temperature, the formation of dansylated lipid I was monitored by fluorescence enhancement (excitation at 355 nm, emission at 535 nm). The mixtures contained 2% DMSO in order to increase the solubility of the compounds (concentrations; 0.001, 0.01, 0.1, 1, 10, 100, 1000 nM). This data was collected from three independent experiments (n=3).

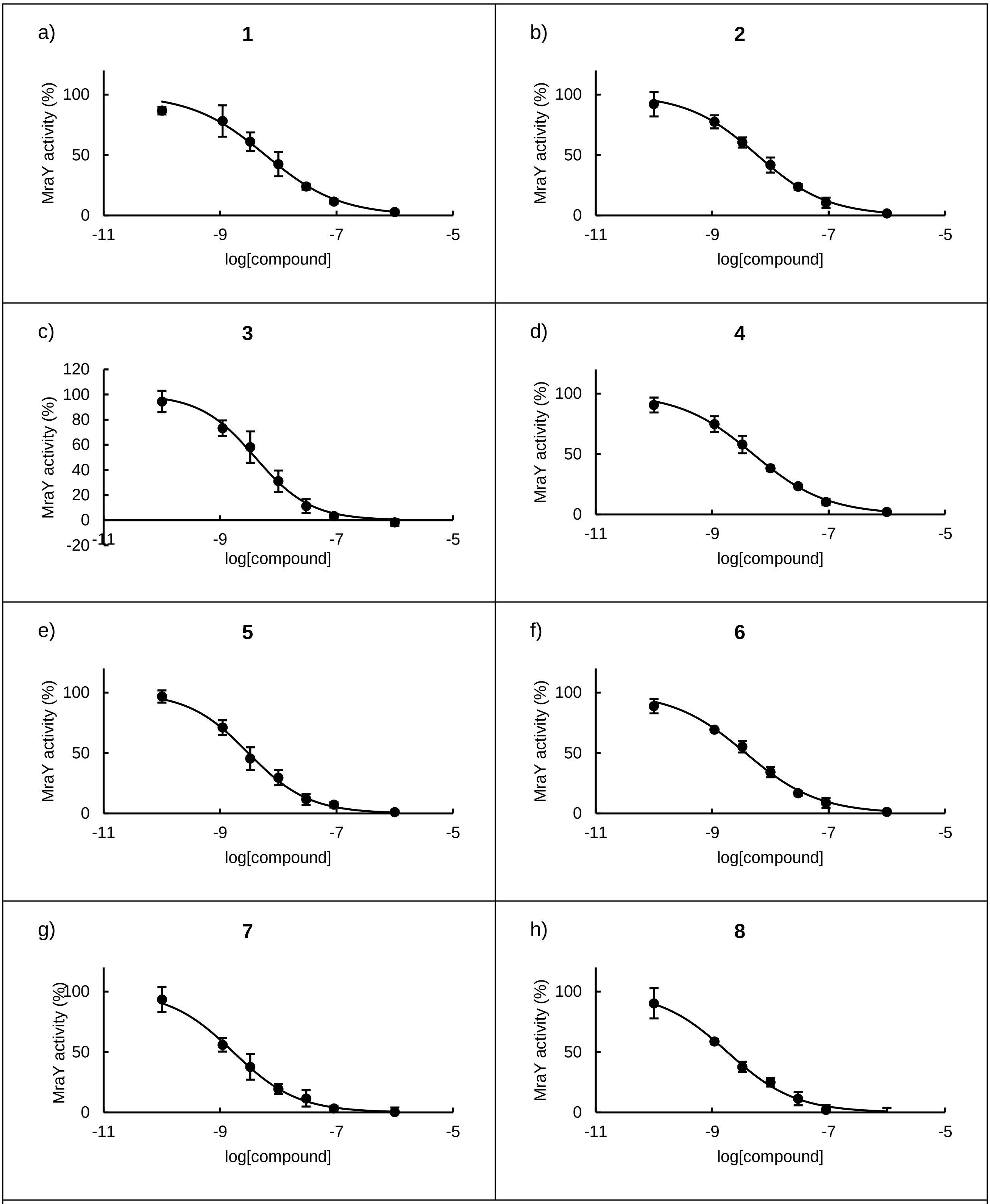

Supplementary Fig. 14. MraY inhibitory activity of the stable analogues 1-8.

Conditions: Reactions were carried out in a 384-well microplate. A solution containing 10 μM dansylated - UDP-MurNAc-pentapeptide and 50 μM undecaprenyl phosphate (C_{55}-P) in 20 μL of an assay buffer [50 mM Tris-HCl (pH 7.6), 50 mM KCl, 25 mM $MgCl_2$, 0.2% Triton X-100 and 8% glycerol] was prepered. The reactions were initiated by the addition of *S. aureus* MraY enzyme (5 μL, 11 μg/mL). After 3 h of incubation at room

temperature, the formation of dansylated lipid I was monitored by fluorescence enhancement (excitation at 355 nm, emission at 535 nm). The mixtures contained 2% DMSO in order to increase the solubility of the compounds (concentrations; 0.1, 1.1, 3.3, 10, 30, 90, 1000 nM). This data was collected from three independent experiments (n=3).

		amide MRY				anilide MRY				ampicillin	vancomycin	levofloxacin
		**1**	**2**	**3**	**4**	**5**	**6**	**7**	**8**			
bacterial spp.	strains	MIC (μg/mL)										
S. aureus	ATCC 29213	0.5	1	1	1	0.5	1	1	1	1	1	0.13
MRSA	JE2	1	1	1	2	1	1	1	2	4	1	8
clinically isolated MRSA	#2934	1	0.5	1	1	0.25	0.5	1	1	16	1	>32
	#2931	0.5	0.5	1	1	0.25	0.5	1	1	32	1	16
	#2334	1	1	1	2	1	1	2	2	16	2	>32
	#2110	2	2	2	4	1	2	2	2	16	2	>32
	#2932	2	1	2	2	1	1	2	2	32	2	>32
	#2933	0.5	0.5	1	1	0.5	0.5	1	1	32	1	>32
	#1958	1	1	2	2	1	1	1	2	32	1	>32
	#2935	1	1	2	2	1	1	1	2	16	2	>32
	#2310	2	2	2	2	1	2	2	2	32	2	>32
	#2071	1	1	1	1	0.5	64	1	64	32	1	0.13
E. faecium	ATCC 35667	0.25	0.5	1	0.5	0.25	0.5	0.5	0.5	1	1	4
E. faecium(VRE)	ATCC 51559	0.5	0.5	0.5	1	0.25	1	0.5	1	128	>128	16
	ATCC 51858	2	2	2	2	1	2	2	2	128	128	4
E. faecalis(VRE)	ATCC 51299	0.5	0.5	0.5	0.25	0.25	0.5	0.5	1	1	128	1
clinically isolated E. faecium	1	1	1	1	1	1	1	1	1	>128	1	>32
	2	1	1	1	2	0.5	1	0.5	2	128	1	>32
	3	1	1	1	1	0.5	1	1	2	>128	1	>32
	4	0.5	0.5	0.5	0.5	0.25	0.5	0.5	1	128	1	>32
	5	1	1	1	1	0.5	1	0.5	1	128	1	32
	7	1	2	1	1	0.5	2	0.5	1	0.5	0.5	0.5
	8	0.5	0.5	0.5	0.5	0.25	0.5	0.5	1	128	1	>32
	9	0.5	0.5	0.5	0.5	0.25	1	0.25	1	>128	1	>32
	10	0.5	1	1	1	0.5	1	1	1	>128	1	>32
	11	2	1	1	1	0.5	1	2	2	128	1	>32
	13	0.5	0.5	0.5	0.5	0.25	0.5	0.5	1	>128	1	>32
	14	0.25	0.5	0.5	0.5	0.25	0.5	0.5	0.5	128	0.5	>32
	15	1	1	1	1	0.5	1	1	2	128	1	>32
clinically isolated E. faecalis	21	0.5	0.13	0.5	0.13	0.5	0.25	0.5	1	1	1	0.5
	22	0.25	0.13	0.13	0.13	0.13	0.5	0.13	1	1	1	1
	23	0.5	0.25	0.13	0.13	0.13	0.5	0.5	0.25	1	1	1
	24	0.5	0.25	0.13	0.13	0.25	0.5	0.13	1	0.5	1	1
	25	0.5	0.25	0.13	0.25	0.25	0.5	0.25	0.5	1	1	0.5
	26	0.25	0.13	0.13	0.25	0.13	0.25	0.13	0.25	1	1	0.5
	27	0.25	0.25	0.25	<0.06	0.25	0.5	0.25	0.5	1	1	1
	28	0.13	<0.06	<0.06	<0.06	<0.06	0.13	<0.06	<0.06	1	1	1
	29	0.25	0.13	0.25	<0.06	<0.06	0.25	<0.06	0.13	1	1	0.5
	30	0.25	0.25	0.25	0.25	0.13	0.5	0.25	0.5	0.5	1	1
	31	0.5	0.25	0.25	0.25	0.25	0.5	0.5	0.5	1	4	1
	32	0.25	0.25	0.13	0.25	0.25	0.5	0.25	0.25	1	1	1
	33	0.25	0.13	0.25	0.13	0.13	0.5	0.13	0.5	1	2	1
	34	0.5	0.25	0.25	0.25	0.25	0.5	0.25	0.25	1	1	1
	35	0.5	0.25	0.25	0.25	0.25	0.5	0.5	1	0.5	1	2

Supplementary Table 1. Antibacterial activity of stable analogues against drug-resistant strains and clinically isolated strains.

Experimental details are shown in Methods of main text. These MIC values were determined in a single trial (n=1). MIC values against *S. aureus* ATCC 29213 and *E. faecium* ATCC 35667 strains were identical to Table 1. Drug breakpoint: *S. aureus*; vancomycin ≥16 μg/mL, levofloxacin ≥4 μg/mL, *enterococci*; ampicillin ≥16 μg/mL, vancomycin ≥32 μg/mL, levofloxacin ≥8 μg/mL [M100 Performance Standards for Antimicrobial Susceptibility Testing, 30th Edition (CLSI)].

	amide MRY				anilide MRY			
	**1**	**2**	**3**	**4**	**5**	**6**	**7**	**8**
IC_{50} (μM)	12	16	18	19	22	16	25	23

Supplementary Table 2. Cytotoxic activity of stable analogues 1-8 against HepG2 cells.

Experimental details are shown in methods section of the main text. HepG2 cells ($1x10^5$ cells/mL, 100 μL) were cultured at 37 °C under 5% CO_2 in air in E-MEM (with L-glutamine, phenol red, sodium pyruvate, non-essential amino acids and 1,500 mg/L sodium bicarbonate, wako) supplemented with 10% fetal bovine serum (FBS). After incubation for 24 h, solutions of test compounds in DMSO were added to the culture, and the 96-well plates were incubated for 24 h under above conditions. After that, the WST-8 cell proliferation assay was used to evaluated cell viability. This data was collected from three independent experiments (n=3).

bacterial spp.	*S. aureus*	*E. faecium*	*P. aeruginosa*	*K. pneumoniae*	*E. cloacae*	*A. baumannii*	*E. coli*
strains	ATCC 29213	ATCC 35667	ATCC 27853	ATCC 13883	ATCC 13047	ATCC 19606	ATCC 25922
				MIC[a] (μM)			
vancomycin	<0.5	<0.5	>50	>50	>50	50	>50
ramoplanin	<0.5	<0.5	>50	>50	>50	50	>50
ampicilin	5	<0.5	>50	>50	>50	50	50
streptomycin	50	50	50	50	>50	>50	50
colistin	>12	>12	5	5	>12	5	5
polimyxin B	>12	>12	<0.5	<0.5	>12	<0.5	<0.5
erythromycin	<0.5	<0.5	>50	>50	>50	50	50
azithromycin	5	<0.5	50	5	50	50	5
daptomycin	<0.5	5	>50	>50	>50	>50	>50
rifampicin	<0.5	5	50	50	50	5	5
tunicamycins	>50	50	>50	>50	>50	>50	>50
muraymycin-C15[ref 16]	<0.5	5	>50	50	>50	>50	>50

Supplementary Table 3. Antibacterial activity against ESKAPE of known drugs in the library screening.

Material source

	supplier	code	Lot.
vancomycin	Aldrich	861987-1G	087K0694
ramoplanin	Aldrich	R1781-250MG	SLBV1752
ampicillin	wako	017-10381	TSR3767
streptomycin	TCI	S0585	R07IM-IC
colistin	TCI	C2930	DTY3C-IB
polymyxin B	TCI	P1923	IJ3PM-LP
erythromycin	nacalai-tesque	14549-94	M5A6030
azithromycin	TCI	A2076	3PY8J-JS
daptomycin	TCI	D4229	NOMJH-AT
rifampicin	TCI	R0079	6K4AF-GH
tunicamycins	wako	202-08241	PTK1898

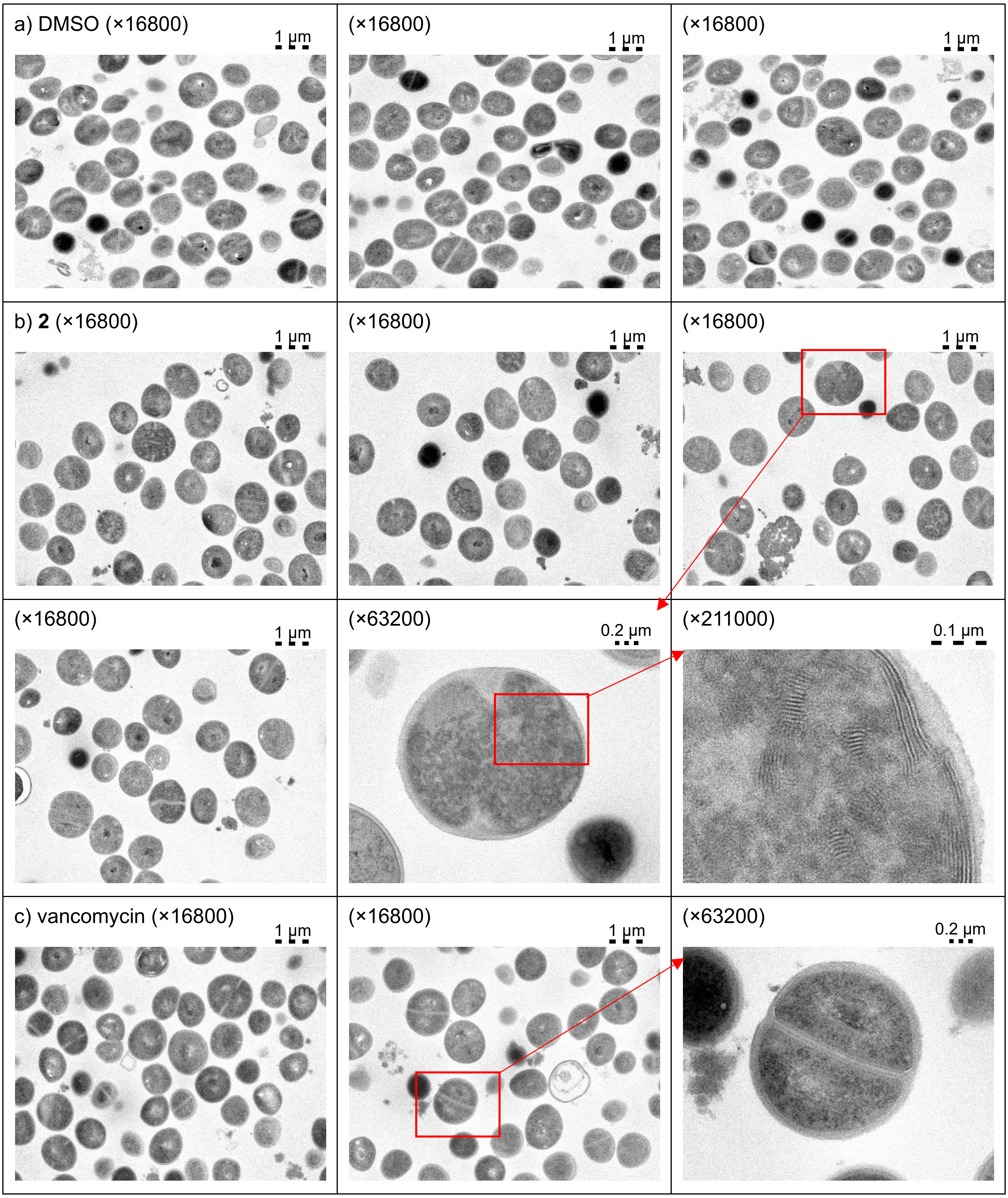

Supplementary Fig. 15. TEM images.

S. aureus (10^9 cfu/mL) was treated with 64 μg/mL of **2** and 32 μg/mL of vancomycin for 3 h. Abnormal membrane-like structures were shown in bacterial cells treated with **2**. These morphological changes were not observed in cells treated with vancomycin. This data was collected from a single trial.

	$MraY_{AA}$-NB7-**2** (EMD-44293) (PDB 9B70)	$MraY_{AA}$-NB7-**3** (EMD-44294) (PDB 9B71)
**Data collection and processing**		
Magnification	81,000	81,000
Voltage (kV)	300	300
Electron exposure (e^{-}/Å^2)	60	60
Defocus range (μm)	-2.0 to -0.8	-1.8 to -0.8
Pixel size (Å)	1.08	1.08
Symmetry imposed	C2	C2
Initial particle images (no.)	1,385,116	1,010,303
Final particle images (no.)	220,165	68,364
Map resolution (Å)	2.88	2.70
FSC threshold	0.143	0.143
Map resolution range (Å)	2.5-4.1	2.4-4.0
**Refinement**		
Initial model used (PDB code)	5CKR	5CKR
Model resolution (Å)		
FSC threshold (0.143)	2.62	2.51
FSC threshold (0.5)	2.96	2.90
Map sharpening *B* factor (Å^2)	-30	-30
Model composition		
Nonhydrogen atoms	7220	7224
Protein residues	930	930
Ligands	2	2
Water	6	6
B factors (Å^2)		
Protein	90.75	66.77
Ligand	90.69	67.03
R.m.s. deviations		
Bond lengths (Å)	0.003	0.004
Bond angles (°)	0.631	0.582
**Validation**		
MolProbity score	1.12	1.08
Clashscore	3.3	2.95
Poor rotamers (%)	0	0.28
Ramachandran plot		
Favored (%)	98.05	98.92
Allowed (%)	1.95	1.08
Disallowed (%)	0	0

Supplementary Table 4. Cryo-EM data collection, refinement, and validation statistics.

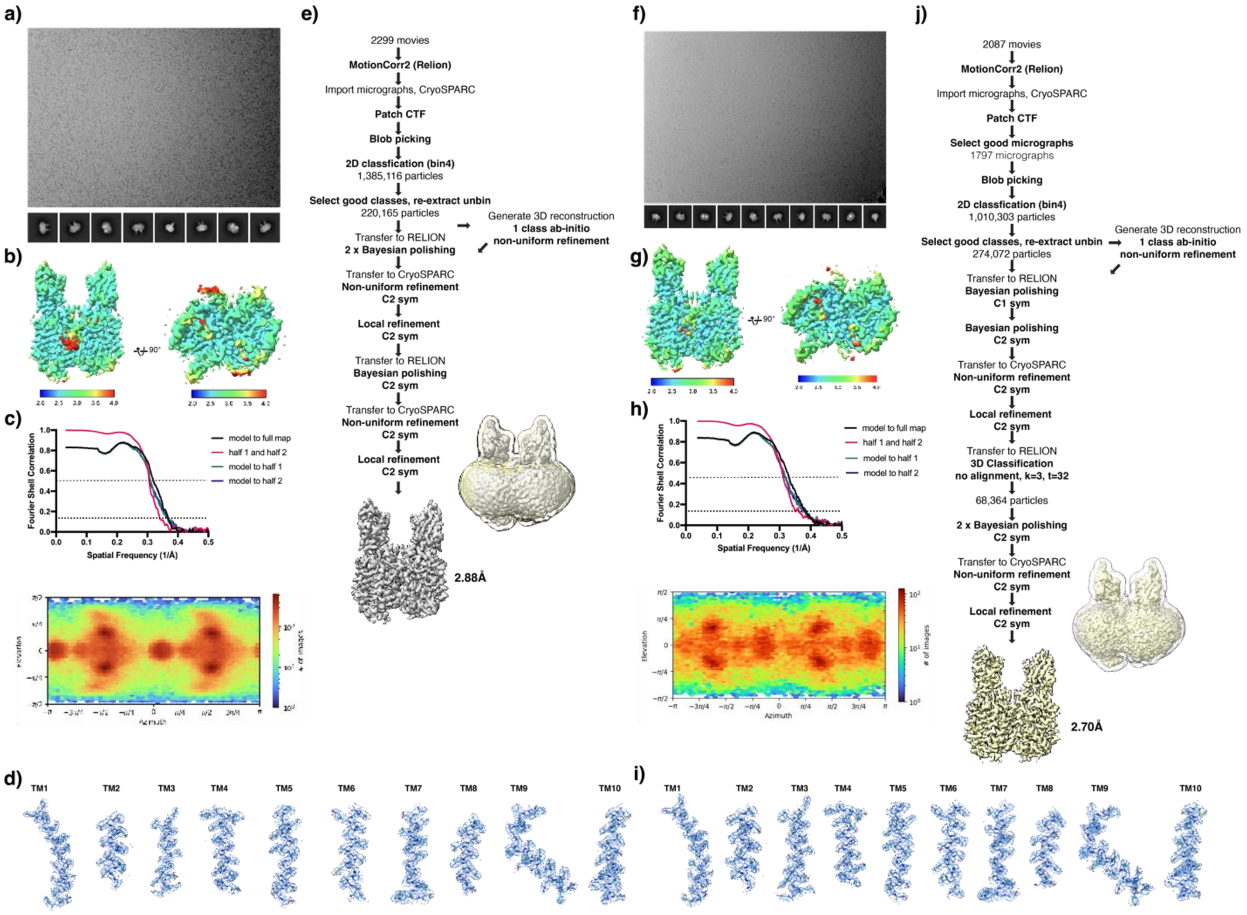

Supplementary Fig. 16. Cryo-EM data processing.

a) representative micrograph and 2D classification for MraY-NB7-**2**.

b) local resolution estimation for MraY-NB7-**2**.

c) phenix reported Fourier shell correlations, and particle angular distribution for the final map of MraY-NB7-**2**. The 0.143 and 0.5 FSC cutoffs are shown as dotted lines.

d) cryo-EM density corresponding to MraY TM1-10 for MraY-NB7-**2** (map threshold = 0.25).

e) processing workflow for MraY-NB7-**2**. The mask used during local refinement is shown.

f) representative micrograph and 2D classification for MraY-NB7-**3**.

g) local resolution estimation for MraY-NB7-**3**.

h) phenix reported Fourier shell correlations, and particle angular distribution for the final map of MraY-NB7-**3**. The 0.143 and 0.5 FSC cutoffs are shown as dotted lines.

i) cryo-EM density corresponding to MraY TM1-10 for MraY-NB7-**3** (map threshold = 0.25).

j) processing workflow for MraY-NB7-**3**. The mask used during local refinement is shown.

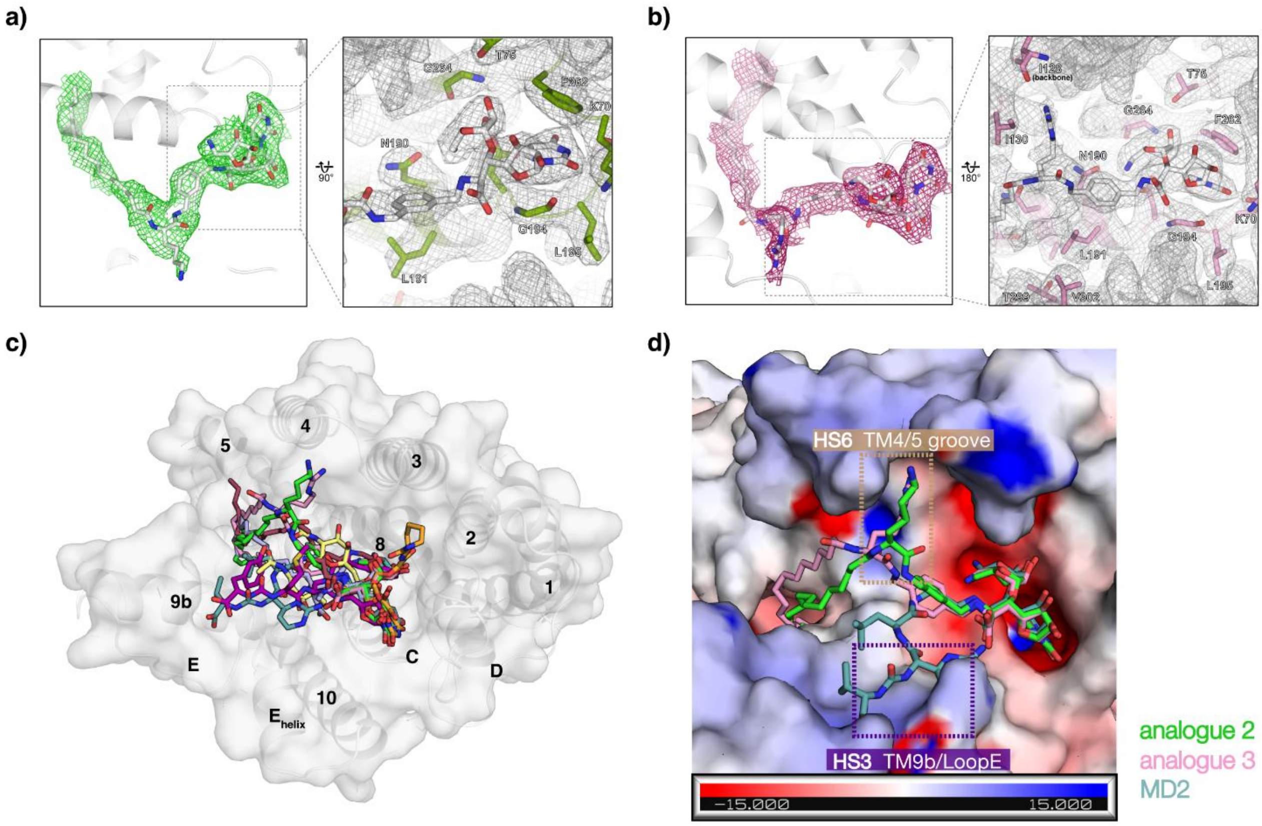

Supplementary Fig. 17. Detailed complex structure of MraY$_{AA}$-2 and MraY$_{AA}$-3.

a) Left shows the cryo-EM density of **2** in green mesh. Right shows the density of **2** and interacting residues from a different viewing angle.

b) Left shows the cryo-EM density of **3** in pink mesh. Right shows the density of **3** and interacting residues from a different viewing angle.

c) Structural overlay of MraY$_{AA}$ bound to **2** (green), **3** (pink), carbacaprazamycin (light blue), capuramycin (orange), 3′-hydroxymureidomycin A (purple), SPM-1 (deep red), and muraymycin D2 (light teal), and MraY$_{CB}$ bound to tunicamycin (yellow) viewed from the cytoplasm.

d) Electrostatic surface representation of the binding site with **2** (green), **3** (pink), and muraymycin D2 (MD2, light teal) overlay. MD2 occupies TM9b/Loop E pocket while analogues **2** and **3** occupies the TM4/5 groove. Electrostatics calculated with APBS[1].

2. Supplementary Notes

2-1. Detailed discussions about the results of the library of MraY inhibitors

A. Elucidation of MraY inhibitory activity of the hydrazone library (Fig. 2)

The library was directly screened using a fluorescent-based MraY assay[2-5] to measure inhibition. The MraY inhibitory activity of each sub-libraries was evaluated at a certain concentration one order of magnitude above the IC_{50} value of the parent natural products (100 nM of TUN and MRD, 10 nM of MRY, 1000 nM of CAP sub-library), where differences tend to be more obvious.

The results of the TUN sub-library are shown in Fig. 2b, c. The core aldehydes TUNp-CHO and TUNm-CHO exhibited almost no inhibition at 10 μM, which represents a nearly 100-fold decrease in activity compared to the parent tunicamycin (Supplementary Fig. 9a, b).[6] The TUNm-library showed a similar trend to TUNp-library. The analogues of BZ-, PA-, AC-, and AA-type hydrazones exhibited almost no inhibitory activity for TUN sub-library. On the other hand, among LA-types having long-chain lipophilic substituents (isostearoyl and palmitoyl group), TUNp-LA71 and TUNp-LA72, which have Ala, TUNp-LA75 and TUNp-LA76, which have Phe, and TUNp-LA79 and TUNp-LA80, which have Ser, exhibits improved MraY inhibition (50-80% inhibition at 100 nM) relative to the parent tunicamycin (~40% inhibition at 100 nM).[6] In addition, TUNp-LA83 and TUNp-LA84, which have Glu, TUNp-LA91 and TUNp-LA92, which have Lys, and TUNp-LA95 and TUNp-LA96, which have Arg, were equipotent to or possessed slightly reduced activity compared to the natural tunicamycin. Taken together, these results suggest that the long-chain lipophilic acyl group substantially contributes to the observed MraY inhibition.

The results of the MRY sub-library are shown in Fig. 2d, e. The core aldehydes MRYp-CHO and MRYm-CHO exhibit only about 10% inhibition at 100 nM (Supplementary Fig. 9c, d). Compared to the TUN sub-library, more hydrazones exhibited improved inhibition relative to the parent MRY-core. In the BZ- and PA-type hydrazones among MRYp-library, the inhibitory potency of hydrazones containing lipophilic substituents at the *para*-position of the aromatic ring of the hydrazine tended to be high, and in particular, MRYp-BZ2 exhibited remarkable inhibition of MraY (90% inhibition at 10 nM). In the AC-, AA-, and LA-type hydrazones, the length of the alkyl group correlated with the inhibition, which was congruent with previous reports.[7,8] In particular, those with LA-type long-chain acyl groups (isostearoyl and palmitoyl group) are preferable although there is no remarkable preference between amino acid residues, and MRYp-LA84, which has Glu, and MRYp-LA92, which has Lys, exhibited particularly potent MraY inhibition (80% inhibition at 10 nM). These results suggest that the hydrazine fragment moiety of the MRY sub-library extends in the direction of the lipophilic groove, and the lipophilic interaction favorably affects MraY inhibition. Although MRYm-library showed roughly a similar trend to MRYp-library, the inhibition potency was greatly reduced in some hydrazones (MRYm-BZ2 and MRYm-BZ47). This difference in potency may be due to the substitution position of the hydrazone bond, which directs hydrophobic substitutions in a different orientation resulting in reduced interactions with MraY.

The results of the MRD-type library are shown in Fig. 2f, g. The core aldehyde MRDp-CHO and MRDm-CHO exhibits ~15% inhibition of MraY at 100 nM (Supplementary Fig. 9e, f).[9] While most hydrazones did not inhibit MraY at 100 nM, some hydrazones did display more potent inhibition than the cores; namely MRDp-BZ6, MRDp-BZ9, MRDp-BZ37, MRDp-BZ38, MRDp-LA97, MRDm-PA54, MRDm-LA69, and MRDm-LA97. Interestingly,

linking to the fragment LA97 resulted in potent inhibition independent of *p*- or *m*-substitution. The MRD sub-library showed a different trend than the other two sub-libraries wherein, the inhibition was not enhanced by the addition of a lipophilic alkyl chain.[9]

The results of the CAP sub-library are shown in Fig. 2h. The CAP-CHO core aldehyde possessed the weakest MraY inhibition of all of the core aldehydes examined with only ~15% inhibition at 1000 nM (Supplementary Fig. 9g). In the CAP sub-library, hydrazones containing amino acid (AA and LA-type) exhibited weak inhibition, whereas BZ-type hydrazones were more potent. This result is the opposite of the MRY and TUN sub-libraries and suggests that aromatic substituents, not lipophilic alkyl chains, enhance the affinity for MraY in the CAP sub-library.

B. Elucidation of antibacterial activity of the hydrazone library (Fig. 2)

Antibacterial activity of vancomycin and colistin were shown in Fig. 2 and antibacterial activity of other antibiotics were shown in Supplementary Table 3.

In the TUN sub-library, BZ-, PA-, AC-, and AA-type hydrazones exhibited no antibacterial activity or MraY inhibition, while analogues possessing LA76 and LA79 exhibited antibacterial activity against gram-positive bacteria equal to or higher than that of natural tunicamycin yielding a minimum inhibitory concentration (MIC) 50 μM against *E. faecium*, which was consistent with their improved MraY inhibition (Fig. 2b, c, Supplementary Table 3). In addition, the antibacterial spectrum of hydrazones possessing fragments LA91, LA92, LA94, LA96, and LA98 containing basic amino acid residues, Lys or Arg, expanded the spectrum to include gram-negative bacteria (MIC 50 μM against *A. baumannii*).

In the MRY sub-library, the LA-type hydrazones exhibited higher antibacterial activity, a trend also observed with the TUN sub-library (Fig. 2d, e). In particular, MRYp-LA71, MRYp-LA72, MRYp-LA79, MRYp-LA80, MRYp-LA87, and MRYp-LA88, which have Ala, Ser, or Gln residue, showed potent antibacterial activity against *S. aureus* with MICs below 0.5 μM. Moreover, the antibacterial spectrum of MRYp-LA91, MRYp-LA92, and MRYp-LA98, which contain a Lys residue expanded the spectrum of activity to include gram-negative bacteria (MIC <0.5 μM against *E. coli* and *K. pneumoniae*). On the other hand, MRYp-BZ2, whose MraY inhibition was as potent as the LA-type, showed no antibacterial activity. These results are consistent with previous reports,[7,8] and suggest that the bacterial membrane permeability is limited by the high polarity of the core structure.

Unlike the TUN and MRY sub-libraries mentioned above, few hydrazones exhibited antibacterial activity for MRD and CAP sub-libraries (Fig. 2f-h). Only weak antibacterial activity with MICs of 50 μM was observed for some analogues. In general, the active hydrazones possess a basic Lys or Arg residue and a long acyl chain, similar to other cationic antimicrobial lipopeptides. Unfortunately, the hydrazones in the MRD and the CAP sub-libraries did not enhance the antibacterial activity compared to the activity of fragments (Supplementary Fig. 10).

2-2. Application of build-up library synthesis for tubulin-binding natural products

Tubulin-binding natural products are well-known compounds and used as antitumor drugs. This series includes epothilone B, paclitaxel, and vinblastine, which have very complex structures and exhibit very strong cytotoxicity, so drug discovery based on tubulin binders have been studied well. However, the analogue synthesis of these natural products are tough tasks because of their complicated and long synthetic route. Therefore, we thought that these natural products are suitable for application for our strategy as feasibility study. We designed six core aldehydes of epothilone B, paclitaxel, and vinblastine according to previous semi-synthetic methods (Supplementary Fig. 18, Scheme S7-9). This allows us to synthesis libraries with different positions of natural product skeleton converted. In the case of epothilone B, the 7-hydroxyl group was modified, which was obtained directly from epothilone B[10]. Additionally, a nitrogen atom in the aziridine analogue was modified by 3-formylbenzyl group, inspired by Nicolaou' reports[11,12]. In the case of paclitaxel, an amino group in the 3-amino-2-hydroxy-3-phenylpropionyl moiety and a hydroxyl group in the taxane scaffold [13,14]. In the case of vinblastine, 5-formyl analogue[15,16] which could be obtained directly formylation of vinblastine, and 7-acyl analogue, which could be obtained from 2-steps transformations, were selected.

The library synthesis with these core aldehydes was conducted similar to synthesis of the library of MraY inhibitors. Two microliters of 10 mM DMSO solution of each aldehyde and two microliters of 10 mM DMSO solution of hydrazine (98 compounds) were applied to 96-well microplate, and the mixture was diluted with 16 μL of DMSO to 1 mM solution. The microplate including reaction solutions were shaken for 30 min. The reaction mixtures were concentrated *in vacuo*, and the residues were dissolved with 20 μL of DMSO of afford 1 mM DMSO solutions of hydrazone.

Firstly, tubulin polymerization assay was conducted with assay kit which detects microtubule polymerization by increasing fluorescence intensity (Supplementary Fig. 19a). Epothilone B and paclitaxel stabilize microtubule, resulting in increasing fluorescence intensity compared to DMSO control (Supplementary Fig. 19c,d). Core aldehydes epo-azi-CHO and pac-O-CHO maintained the activity of the parent natural products, while pac-N-CHO was more active than paclitaxel. However, epo-O-CHO exhibited a significant decrease in activity. Vinblastine inhibits microtubule polymerization, resulting in decreasing fluorescence intensity (Supplementary Fig. 19e). Both vin-ind-CHO and vin-O-CHO maintained the activity of vinblastine. From these results, we next evaluated the microtubule stabilizing/destabilizing activity of epo-azi, pac-N, and vin-O libraries (Supplementary Fig. 20). Because different classes of natural products have different effects on polymerization (Supplementary Fig. 20a-c), we compared the activity of the hydrazone analogues in terms of fluorescence intensity at the time when the difference is most apparent for each. In the epo-azi library, most of the analogues exhibited reduced activity, but there were a few that exhibited activity equal to or better than epo-azi-CHO and comparable to epothilone B (Supplementary Fig. 20d). In the pac-N library, many analogues exhibited improved activity over paclitaxel, with some exhibiting activity comparable to pac-N-CHO (Supplementary Fig. 20e). In the vin-O library, a few of analogues exhibited improved activity over vinblastine and vin-O-CHO (Supplementary Fig. 20f).

Next, cytotoxicity of these libraries against HCT-116 cells was evaluated (Supplementary Fig. 21). Unlike the results of the microtubule polymerization assay, the cell growth inhibitory activities of core aldehydes were lower than that of the parent natural products. Core aldehyde epo-azi-CHO, however, maintained most of the activity of the

epothilone B (Supplementary Fig. 21a). Based on these results, the cell growth inhibitory activity of the libraries was evaluated at two concentrations (Supplementary Fig. 22). With the hydrazones synthesized in this study, we did find analogues with activity equivalent to or higher than that of natural product in epo-azi library (Supplementary Fig. 22a) and improved activity over the core aldehyde in pac-N and vin-O library (Supplementary Fig. 22c,f). Compared with the results of tubulin binding evaluation, we can simultaneously observe analogues that exhibit good activity in both tubulin polymerization promotion/inhibition and cytotoxicity (epo-azi-BZ21, epo-azi-BZ31, epo-azi-PA49, epo-azi-AC55, epo-azi-LA79, pac-N-PA49, pac-N-LA79, vin-O-BZ28), as well as those that exhibit high tubulin polymerization promotion/inhibition activity but do not exhibit cytotoxicity (epo-azi-BZ30, epo-azi-AC57, vin-O-AA series).

The results from the evaluation of tubulin-binding natural products are a clear indication that there does exist a discrepancy between protein-based and cell-based activities in natural product drug discovery. However, our strategy allows us to evaluate protein-based and cell-based activities simultaneously, so we can obtain a lot of information while taking cell-based activity into account. Especially for compounds with high cytotoxicity, such as tubulin-binding natural products, conjugation with various molecules will be considered to achieve selective toxicity. In many cases, the introduction of a linker reduces the activity, but our strategy has the potential to be applied to optimize the linkers available for conjugation. We have completed these experiments, which include synthesis of 6 core aldehydes of natural products, a build-up library synthesis consisting of 588 analogues, and their biological evaluation only 3 months. These efforts demonstrate the generality of our strategy.

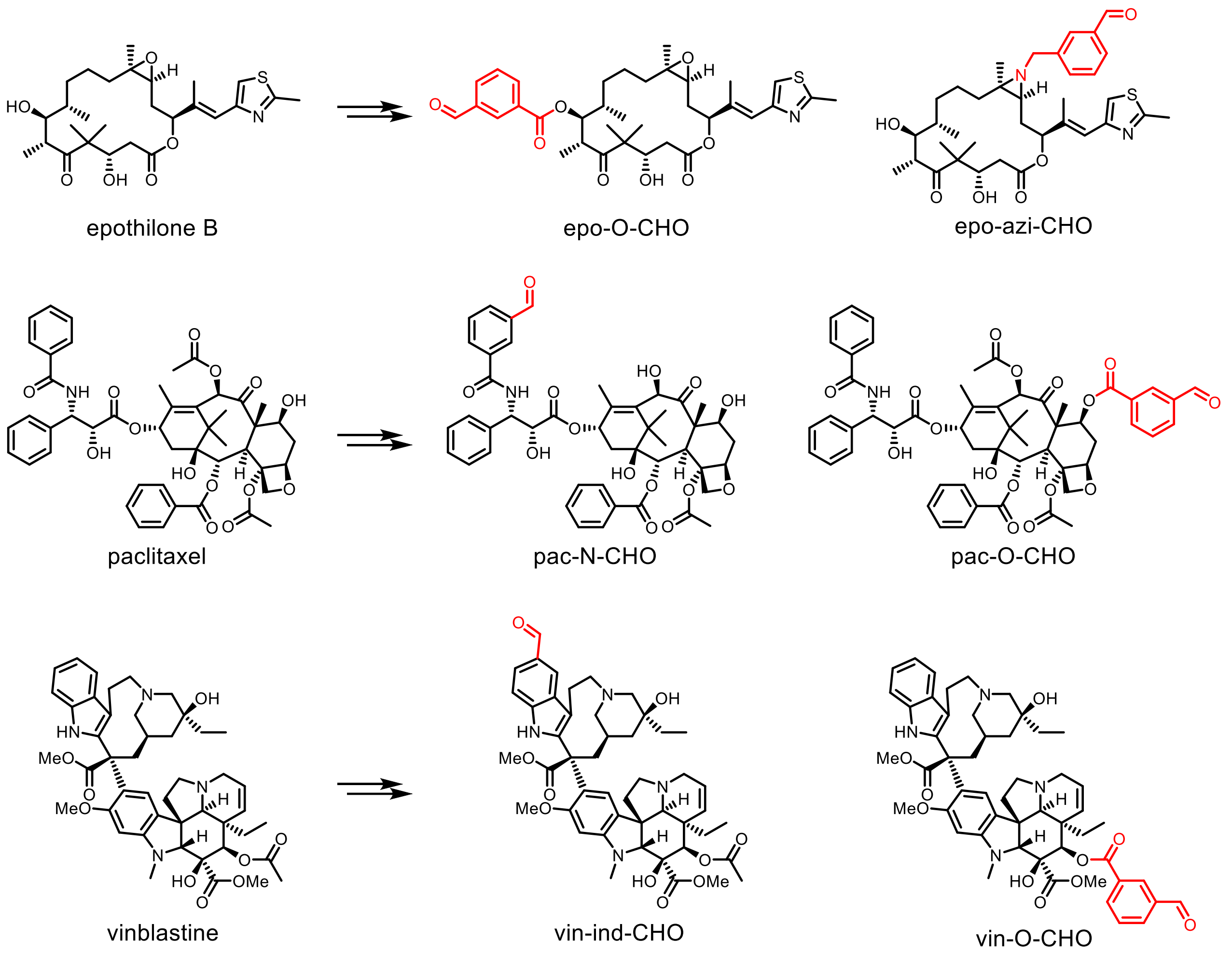

Supplementary Fig. 18. Structures of tubulin-binding natural products (epothilone B, paclitaxel, vinblastine) and designed core aldehydes.

Core aldehydes were named according to the first three letters of natural products and the substitution pattern of the formyl groups (3-formylbenzoyl, 3-formylbenzyl, formyl).

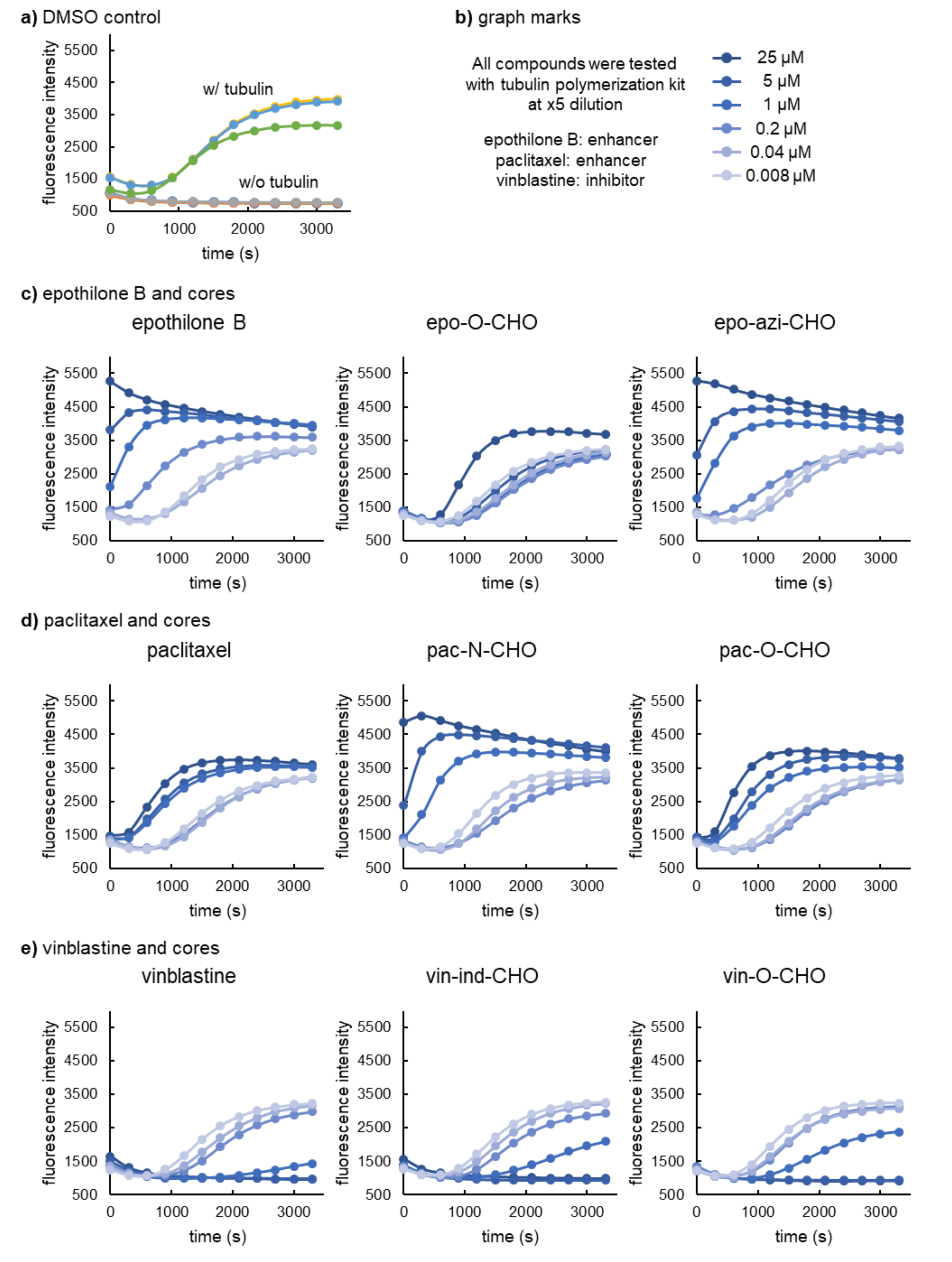

Supplementary Fig. 19. Tubulin polymerization assay with tubulin polymerization kit (Cytoskeleton, Inc.).

a) The fluorescence intensity increases as tubulin polymerizes without compounds. **b**) The correspondence between the marks on the graphs and the concentrations of compounds is shown. **c**) These are graphs of the change in fluorescence intensity of epothilone B and its core aldehydes. Epothilone B stabilizes microtubules, resulting in a rapid increase in fluorescence intensity. **d**) These are graphs of the change in fluorescence intensity of paclitaxel and its core aldehydes. Paclitaxel stabilizes microtubules, resulting in a rapid increase in fluorescence intensity. However, at high concentrations, the increase in fluorescence intensity is small, probably due to low solubility. **e**) These are graphs of the change in fluorescence intensity of vinblastine and its core aldehydes. Vinblastine destabilizes microtubules, resulting in no increase in fluorescence intensity. These data were collected from a single trial (n=1).

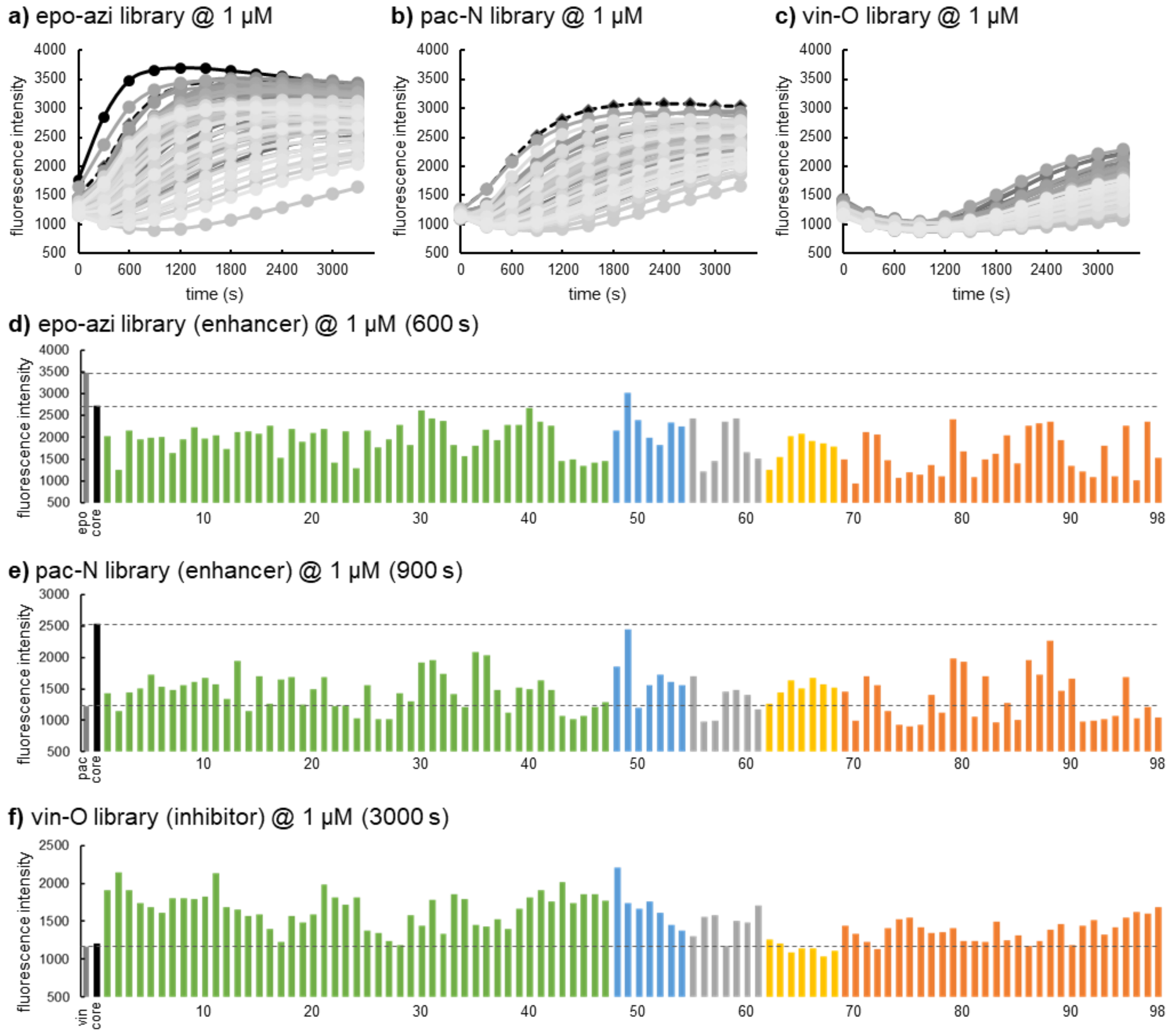

Supplementary Fig. 20. The results of tubulin polymerization assays of the hydrazone library. **a-c**) The fluorescence intensities of all compounds in the library were plotted against time. The results for the original natural product and core aldehyde are represented by solid and dashed black lines, respectively. **d**) The bar graph shows the fluorescence intensities at 600 seconds. Epothilone stabilizes microtubules and inhibits depolymerization, so a higher bar indicates higher activity. **e**) The bar graph shows the fluorescence intensities at 900 seconds. Paclitaxel stabilizes microtubules and inhibits depolymerization, so a higher bar indicates higher activity. **f**) The bar graph shows the fluorescence intensities at 3000 seconds. Vinblastine inhibits microtubule polymerization, so a lower bar indicates higher activity. These data were collected from a single trial (n=1).

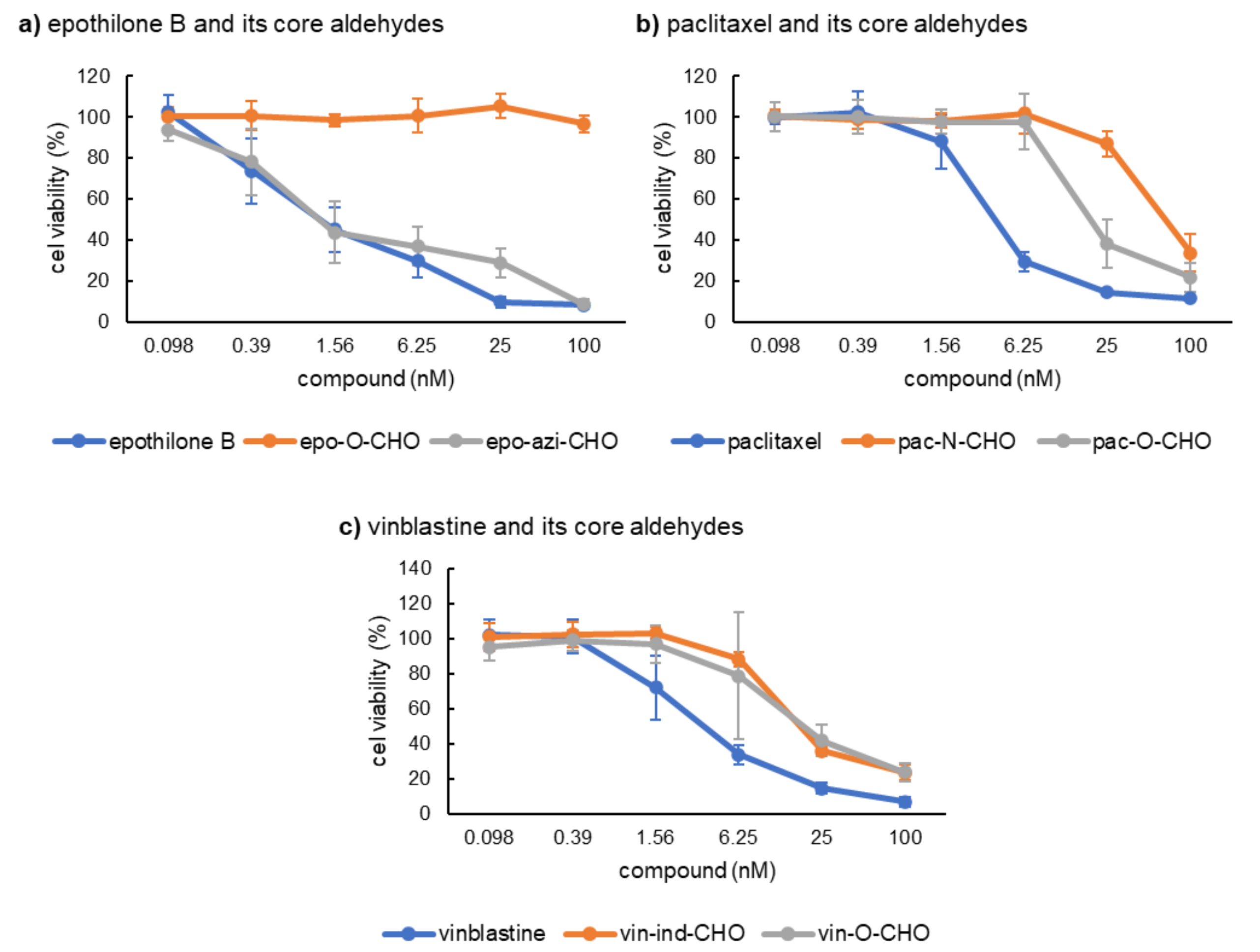

Supplementary Fig. 21. The results for cell growth inhibitory assay of original natural products and core aldehydes.

a) Cell viability plots of epothilone B, epo-O-CHO, and epo-azi-CHO. **b**) Cell viability plots of paclitaxel, pac-N-CHO, and pac-O-CHO. **c**) Cell viability plots of vinblastine, vin-ind-CHO, and vin-O-CHO. HCT-116 cells ($1x10^4$ cells) were cultured at 37 °C under 5% CO_2 in air in McCoy's 5A (1x) (Gibco^{TM}) supplemented with 10% fetal bovine serum (FBS). After incubation for 24 h, solutions of test compounds in DMSO were added to the culture, and the 96-well plates were incubated for 72 h under above conditions. After that, the WST-8 cell proliferation assay was used to evaluated cell viability. These data were collected from three independent experiments (n=3).

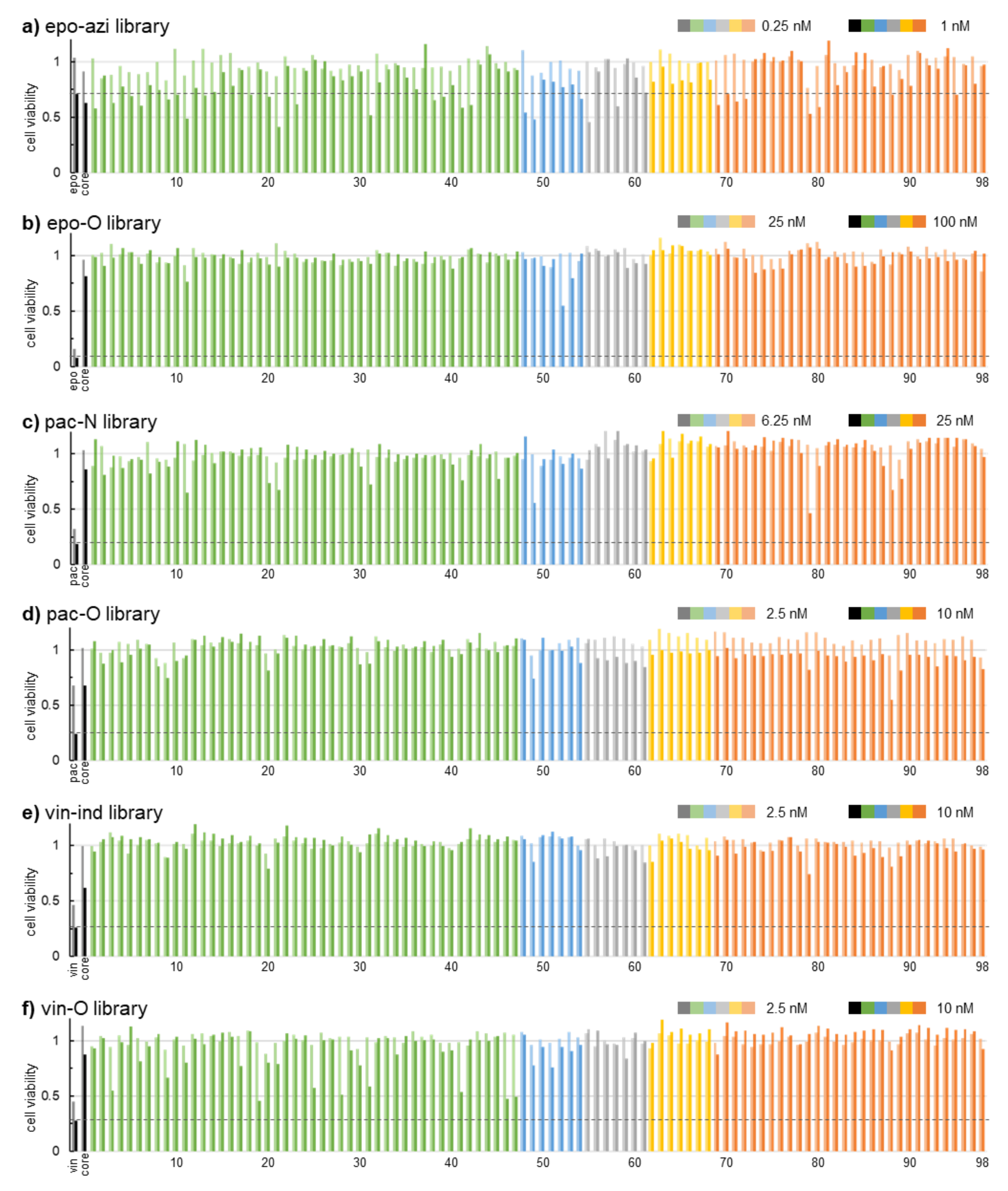

Supplementary Fig. 22. The results for cell growth inhibitory assay (HCT-116, 72 h, WST-8) of the hydrazone library.

The horizonal axis indicates the hydrazone number (green; BZ-type, blue; PA-type, gray; AC-type, yellow; AA-type, orange; LA-type). The left side of each graph shows the results for the original natural products and their core aldehydes (black). The dashed lines show the viability when treated with the higher concentrations of original natural products. These data were collected from a single trial (n=1).

3. Supplementary Methods

3-1. Preparation of compounds

General experimental methods

All reactions except those carried out in the aqueous phase were performed under an argon atmosphere unless otherwise noted. Materials were purchased from commercial suppliers and used without further purification unless otherwise noted. Solvents were distilled according to the standard protocol. Isolated yields were calculated by weighing products. The weight of the starting materials and the products were not calibrated. Analytical thin layer chromatography (TLC) was performed on Merck silica gel $60F_{254}$ plates. Normal-phase column chromatography was performed on Merck silica gel 5715 or Wakogel 60N. Flash column chromatography was performed on Kanto Chemical Silica Gel 60N (spherical, neutral. 40-50 μm). ^{1}H NMR was measured in $CDCl_3$, DMSO-d_6, and methanol-d_4 solution, and reported in parts per million (δ) relative to tetramethylsilane (0.00 ppm) as an internal standard using JEOL ECS400, ECX400, ECZ400, ECA500, unless otherwise noted. ^{13}C NMR was measured in $CDCl_3$ or methanol-d_4 solution, and referenced to residual solvent peaks of $CDCl_3$ (77.16 ppm) or methanol-d_4 (49.00 ppm) using JEOL ECS400, ECX400, ECZ400, ECA500. Coupling constant (*J*) was reported in hertz (Hz). Abbreviations of multiplicity were as follows; s: singlet, d: doublet, t: triplet, q: quartet, m: multiplet, br: broad. Data were presented as follows; chemical shift (multiplicity, integration, coupling constant). The assignment was based on ^{1}H-^{1}H COSY spectra. Mass spectra were obtained on Waters MICRO MASS LCT-premier and the mass analyzer type used for the HRMS measurements was TOF. Optical rotation was measured on a Rudolph Research Analytical Autopol IV automatic polarimeter.

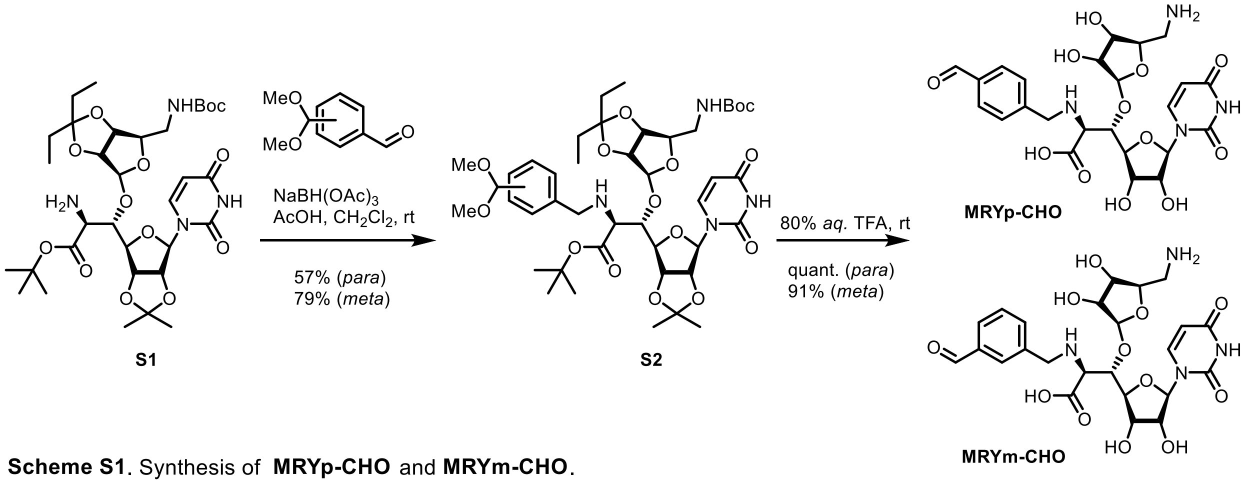

Scheme S1. Synthesis of **MRYp-CHO** and **MRYm-CHO**.

General procedure A

A solution of **S1**[7] (1.0 equiv.) in CH_2Cl_2 (0.5 mL) was treated with aldehyde (1.0~1.5 equiv.), AcOH (3 equiv.), and $NaBH(OAc)_3$ (1.5 equiv.) at room temperature for 1 h. Additional $NaBH(OAc)_3$ (1.5 equiv.) was added to the reaction mixture. After stirring for 1 h, the reaction mixture was quenched with *sat. aq.* $NaHCO_3$ (50 μL), and the mixture was partitioned between EtOAc and *sat. aq.* $NaHCO_3$. The organic layer was washed with brine, dried (Na_2SO_4), filtered and concentrated *in vacuo*. The residue was purified by preparative TLC or silica gel column chromatography to afford products.

tert-Butyl 5-*O*-[5-(*tert*-butyloxycarbonylamino)-5-deoxy-2,3-*O*-(3-pentylidene)-β-D-*ribo*-pentofuranosyl]-6-deoxy-6-[4-(dimethoxymethyl)benzylamino]-2,3-*O*-isopropylidene-1-(uracil-1-yl)-β-D-*glycelo*-L-*taro*-heptofuranuronate (***para*-S2**)

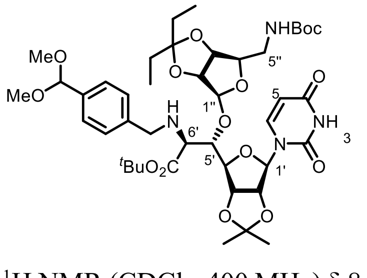

According to general procedure A, **S1** (10.0 mg, 14.0 μmol) and 4-(dimethoxymethyl)benzaldehyde (2.6 mg, 14.4 μmol, 1.03 equiv.) were used to afford ***para*-S2** (7.0 mg, 57%) as a white solid after silica gel column chromatography (preparative TLC MeOH/$CHCl_3$ = 4%).

^{1}H NMR ($CDCl_3$, 400 MHz) δ 8.22 (br s, 1H, N*H*-3), 7.35 (s, 4H, Ar), 7.20 (d, 1H, H-6, $J_{6,5}$ = 8.1 Hz), 5.95 (br t, 1H, BocN*H*, J = 6.1 Hz), 5.66 (d, 1H, H-5, $J_{5,6}$ = 8.1 Hz), 5.52 (d, 1H, H-1', $J_{1',2'}$ = 1.8 Hz), 5.36 (s, 1H, $(MeO)_2CH$), 5.07 (dd, 1H, H-2', $J_{2',3'}$ = 6.7, $J_{2',1'}$ = 1.8 Hz), 5.01 (s, 1H, H-1''), 4.75 (dd, 1H, H-3', $J_{3',2'}$ = 6.7, $J_{3',4'}$ = 4.5 Hz), 4.63 (dd, 1H, H-4', $J_{4',5'}$ = 9.0, $J_{4',3'}$ = 4.5 Hz), 4.56-4.51 (m, 2H, H-2'', H-3''), 4.24 (m, 1H, H-4''), 4.07 (br d, 1H, H-5', J = 9.0 Hz), 3.94 (d, 1H, ArCH_2N, J_{gem} = 13.2 Hz), 3.59 (d, 1H, ArCH_2N, J_{gem} = 13.2 Hz), 3.34-3.32 (m, 1H, N*H*), 3.32 (s, 6H, OMe×2), 3.29 (d, 1H, H-6', J = 2.0 Hz), 3.23-3.16 (m, 1H, H-5''), 3.12-3.04 (m, 1H, H-5''), 1.58 (s, 3H, isopropylidene-CH_3), 1.52 (s, 9H, ^{t}Bu), 1.39 (s, 9H, ^{t}Bu), 1.33 (s, 3H, isopropylidene-CH_3), 1.56-1.40 (m, 4H, pentylidene-CH_2×2), 0.77 (t, 6H, pentylidene-CH_3×2, J = 7.2 Hz); ^{13}C NMR ($CDCl_3$, 100 MHz) δ 192.1, 163.3, 156.3, 150.0, 143.8, 143.6, 129.9, 127.1, 116.3, 116.1, 115.1, 114.7, 112.9, 112.6, 103.0, 102.5, 102.4, 96.5, 95.8, 88.2, 87.1, 87.0, 86.7, 86.5, 84.8, 84.6, 82.2, 82.1, 82.0, 81.7, 79.3, 79.1, 52.9, 52.9, 29.8, 29.4, 28.9, 28.5, 28.5, 28.3, 28.2, 28.0,

27.4, 27.4, 25.7, 25.6, 8.6, 7.4, 7.4; ESIMS-HR *m/z*: $[M+H]^+$ calcd. for $C_{43}H_{65}N_4O_{115}$ 877.4441, found 877.4452; $[\alpha]^{27}_{D}$ +7.12 (*c* 0.70, $CHCl_3$).

tert-Butyl 5-*O*-[5-(*tert*-butyloxycarbonylamino)-5-deoxy-2,3-*O*-(3-pentylidene)-β-D-*ribo*-pentofuranosyl]-6-deoxy-6-[3-(dimethoxymethyl)benzylamino]-2,3-*O*-isopropylidene-1-(uracil-1-yl)-β-D-*glycelo*-L-*taro*-heptofuranuronate (***meta*-S2**)

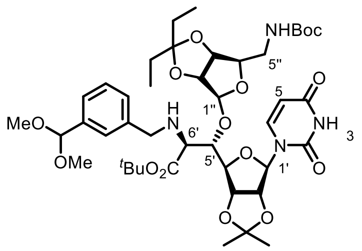

According to general procedure A, **S1** (20.0 mg, 28.1 μmol) and 3-(dimethoxymethyl)benzaldehyde (7.6 mg, 42.1 μmol, 1.5 equiv.) were used to afford ***meta*-S2** (19.4 mg, 79%) as a white solid after silica gel column chromatography (preparative TLC MeOH/$CHCl_3$ = 4%).

^{1}H NMR ($CDCl_3$, 400 MHz) δ 8.32 (br s, 1H, N*H*-3), 7.37-7.27 (m, 4H, Ar), 7.19 (d, 1H, H-6, $J_{6,5}$ = 8.1 Hz), 5.96 (br t, 1H, BocN*H*, *J* = 6.1 Hz), 5.66 (d, 1H, H-5, $J_{5,6}$ = 8.1 Hz), 5.54 (d, 1H, H-1', $J_{1',2'}$ = 1.8 Hz), 5.36 (s, 1H, $(MeO)_2CH$), 5.05 (dd, 1H, H-2', $J_{2',3'}$ = 6.7, $J_{2',1'}$ = 1.8 Hz), 5.02 (s, 1H, H-1''), 4.79 (dd, 1H, H-3', $J_{3',2'}$ = 6.7, $J_{3',4'}$ = 4.5 Hz), 4.59 (dd, 1H, H-4', $J_{4',5'}$ = 9.0, $J_{4',3'}$ = 4.5 Hz), 4.56-4.50 (m, 2H, H-2'', H-3''), 4.25 (m, 1H, H-4''), 4.07 (br d, 1H, H-5', *J* = 8.1 Hz), 3.92 (d, 1H, ArCH_2N, J_{gem} = 13.1 Hz), 3.60 (d, 1H, ArCH_2N, J_{gem} = 13.1 Hz), 3.35-3.30 (m, 2H, H-6', N*H*), 3.32 (s, 3H, OMe), 3.32 (s, 3H, OMe), 3.23-3.17 (m, 1H, H-5''), 3.11-3.03 (m, 1H, H-5''), 1.57 (s, 3H, isopropylidene-CH_3), 1.52 (s, 9H, ^{t}Bu), 1.38 (s, 9H, ^{t}Bu), 1.34 (s, 3H, isopropylidene-CH_3), 1.56-1.39 (m, 4H, pentylidene-CH_2×2), 0.78 (t, 6H, pentylidene-CH_3×2, *J* = 7.2 Hz); ^{13}C NMR ($CDCl_3$, 100 MHz) δ 172.2, 163.3, 156.3, 150.0, 143.3, 140.1, 138.1, 128.7, 128.3, 126.9, 125.5, 116.2, 114.8, 112.8, 103.3, 102.5, 95.5, 87.6, 87.1, 86.7, 84.6, 82.6, 82.2, 81.8, 81.8, 79.0, 61.5, 52.9, 52.8, 52.4, 43.3, 29.5, 28.9, 28.5, 28.4, 27.3, 25.7, 8.6, 7.4; ESIMS-HR *m/z*: $[M+H]^+$ calcd. for $C_{43}H_{65}N_4O_{15}$ 877.4441, found 877.4452; $[\alpha]^{21}_{D}$ +5.81 (*c* 0.97, $CHCl_3$).

MRYp-CHO

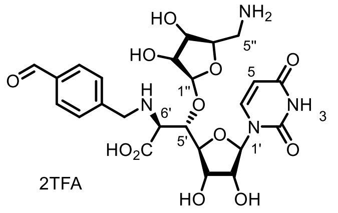

A solution of ***para*-S2** (23.8 mg, 27.1 μmol) in 80% *aq.* TFA (2 mL) was stirred at room temperature for 17 h. The reaction mixture was concentrated *in vacuo*. The resulting solid was washed with CH_2Cl_2 to afford **MRYp-CHO** (22.0 mg, quant.) as a white solid.

^{1}H NMR (D_2O, 500 MHz) δ 9.95 (s, 1H, C*H*O), 7.97 (d, 2H, Ar, *J* = 8.0 Hz), 7.66 (d, 2H, Ar, *J* = 8.0 Hz), 7.58 (d, 1H, H-6, $J_{6,5}$ = 8.0 Hz), 5.82 (d, 1H, H-5, $J_{5,6}$ = 8.0 Hz), 5.66 (d, 1H, H-1', $J_{1',2'}$ = 4.6 Hz), 5.19 (d, 1H, H-1'', $J_{1'',2''}$ = 1.7 Hz), 4.63 (d, 1H, H-5', *J* = 2.9 Hz), 4.58 (d, 1H, Ar-CH_2-N, J_{gem} = 13.2 Hz), 4.47 (t, 1H, H-2', *J* = 4.4 Hz), 4.36 (d, 1H, Ar-CH_2-N, J_{gem} = 13.2 Hz), 4.32-4.27 (m, 2H, H-3', H-4'), 4.18-4.14 (m, 3H, H-2'', H-3'', H-4''), 4.10 (s, 1H, H-6'), 3.34 (dd, 1H, H-5'', J_{gem} = 12.9, $J_{5'',4''}$ = 2.9 Hz), 3.22 (dd, 1H, H-5'', J_{gem} = 12.9, $J_{5'',4''}$ = 10.0 Hz); ^{13}C NMR (D_2O, 1% DMSO-d_6 as an internalized standard, 100 MHz) δ 197.0, 171.8, 167.4, 164.5 (q, J_{CF} = 36.6 Hz), 152.9, 144.9, 139.0, 138.1, 132.5, 132.2, 117.9 (q, J_{CF} = 295.7 Hz), 110.7, 104.1, 93.8, 87.3, 80.8, 77.4, 76.3, 73.3, 73.1, 71.1, 65.1, 53.2, 43.9; ESIMS-HR *m/z*: $[M+H]^+$ calcd. for $C_{24}H_{31}N_4O_{12}$ 567.1933, found 567.1917; $[\alpha]^{20}_{D}$ +18.01 (*c* 0.81, H_2O).

MRYm-CHO

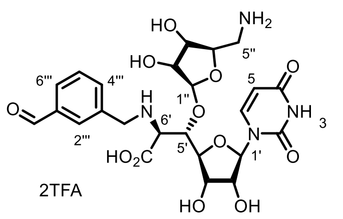

A solution of ***meta*-S2** (26.4 mg, 30.1 μmol) in 80% *aq*. TFA (2 mL) was stirred at room temperature for 17 h. The reaction mixture was concentrated *in vacuo*. The resulting solid was washed with CH_2Cl_2 to afford **MRYm-CHO** (21.8 mg, 91%) as a white solid.

^{1}H NMR (D_2O, 500 MHz) δ 9.95 (s, 1H, C*H*O), 8.02 (d, 1H, H-6''', J = 7.5 Hz), 7.98 (s, 1H, H-2'''), 7.80 (d, 2H, H-4''', J = 8.0 Hz), 7.69 (t, 1H, H-5''', J = 7.5 Hz), 7.60 (d, 1H, H-6, $J_{6,5}$ = 8.0 Hz), 5.82 (d, 1H, H-5, $J_{5,6}$ = 8.0 Hz), 5.67 (d, 1H, H-1', $J_{1',2'}$ = 4.6 Hz), 5.20 (d, 1H, H-1'', $J_{1'',2''}$ = 1.7 Hz), 4.63 (d, 1H, H-5', J = 2.9 Hz), 4.59 (d, 1H, Ar-CH_2-N, J_{gem} = 13.2 Hz), 4.45 (t, 1H, H-2', J = 4.6 Hz), 4.38 (d, 1H, Ar-CH_2-N, J_{gem} = 13.2 Hz), 4.30-4.26 (m, 2H, H-3', H-4'), 4.19-4.13 (m, 3H, H-2'', H-3'', H-4''), 4.09 (s, 1H, H-6'), 3.35 (dd, 1H, H-5'', J_{gem} = 13.4, $J_{5'',4''}$ = 2.9 Hz), 3.22 (dd, 1H, H-5'', J_{gem} = 13.4, $J_{5'',4''}$ = 10.0 Hz); ^{13}C NMR (D_2O, 1% DMSO-d_6 as an internalized standard, 100 MHz) δ 197.0, 172.0, 167.5, 164.5 (q, J_{CF} = 36.6 Hz), 152.8, 144.7, 138.4, 138.0, 133.6, 133.3, 132.3, 131.8, 118.0 (q, J_{CF} = 296.2 Hz), 110.6, 104.0, 93.7, 87.1, 80.8, 77.4, 76.3, 73.4, 73.3, 72.3, 71.0, 65.0, 52.9, 43.9; ESIMS-HR *m/z*: [M+H]$^+$ calcd. for $C_{24}H_{31}N_4O_{12}$ 567.1933, found 567.1917; $[\alpha]^{20}_D$ +9.21 (*c* 0.82, H_2O).

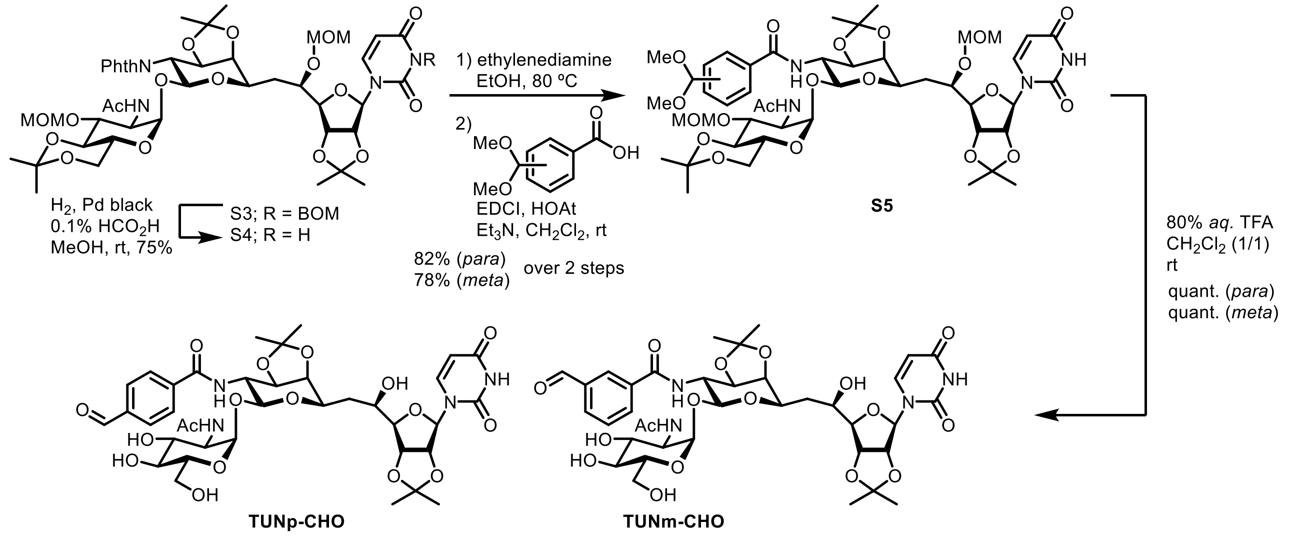

Scheme S2. Synthesis of **TUNp-CHO** and **TUNm-CHO**.

1-{11*S*-6,10-Dideoxy-11-*O*-[2-acetamido-2-deoxy-4,6-*O*-isopropylidene-3-*O*-(methoxymethyl)-α-D-*gluco*-hexopyranosyl]-2,3:8,9-di-*O*-isopropylidene-5-*O*-(methoxymethyl)-10-phthalimido-L-*galacto*-β-D-*allo*-undecodialdo-1,4-furanose-11,7-pyranos-1-yl}uracil (**S4**)

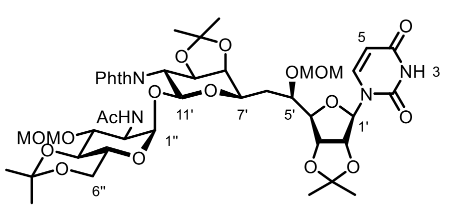

A solution of **S3**[17] (60.0 mg, 56.2 μmol) and Pd black (24 mg) in 0.1% HCO_2H/MeOH (4 mL) was stirred under H_2 atmosphere for 6 h. The insoluble solid was filtered off through a Celite pad and washed with MeOH. The filtrate was concentrated *in vacuo*, and the residue was purified by silica gel column chromatography (ϕ1.1×12 cm; $CHCl_3$/MeOH = 2-5%) to afford **S4** (39.8 mg, 75%) as a white solid and mono-isop-deprotected mixture (12.7 mg, 25%) as a white solid.

^{1}H NMR ($CDCl_3$, 500 MHz) δ 8.92 (br s, 1H, N*H*-3), 7.86 (br s, 2H, Phth), 7.75 (m, 2H, Phth), 7.24 (d, 1H, H-6, $J_{6,5}$ = 8.0 Hz), 5.72 (dd, 1H, H-5, $J_{5,6}$ = 8.0, $J_{5,NH-3}$ = 2.3 Hz), 5.57 (d, 1H, H-1', $J_{1',2'}$ = 1.7 Hz), 5.53 (d, 1H, N*H*-2", J = 8.6 Hz), 5.16 (m, 2H, H-3', H-11'), 4.99 (dd, 1H, H-2', $J_{2',3'}$ = 6.9, $J_{2',1'}$ = 1.7 Hz), 4.94 (d, 1H, H-1", $J_{1'',2''}$ = 3.4 Hz), 4.80 (d, 1H, O-C*H*$_2$-OMe, J_{gem} = 6.3 Hz), 4.75 (dd, 1H, H-9', $J_{9',10'}$ = 9.2, $J_{9',8'}$ = 5.2 Hz), 4.72 (d, 1H, O-C*H*$_2$-OMe, J_{gem} = 6.3 Hz), 4.69 (d, 1H, O-C*H*$_2$-OMe, J_{gem} = 6.3 Hz), 4.54 (d, 1H, O-C*H*$_2$-OMe, J_{gem} = 6.3 Hz), 4.33 (t, 1H, H-10', J = 8.9 Hz), 4.26 (dd, 1H, H-6", J_{gem} = 10.9, $J_{6'',5''}$ = 5.2 Hz), 4.12 (br d, 1H, H-7', J = 10.3 Hz), 4.05 (dd, 1H, H-8', $J_{8',9'}$ = 5.2, $J_{8',7'}$ = 2.3 Hz), 4.00-3.89 (m, 4H, H-4', H-5', H-2", H-5"), 3.71 (t, 1H, H-6", J = 10.6 Hz), 3.66-3.60 (m, 2H, H-3", H-4"), 3.45 (s, 3H, OMe), 3.24 (s, 3H, OMe), 2.29 (m, 1H, H-6'), 1.68 (obscured, H-6'), 1.66, 1.57, 1.50, 1.48, 1.35, 1.31, 1.18 (each s, 3H×7, isopropylidene-C*H*$_3$×6 or Ac); ^{13}C NMR ($CDCl_3$, 100 MHz) δ 170.1, 169.6-167.7 (broad), 163.0, 150.2, 142.4, 134.6, 131.7, 123.8, 114.4, 110.7, 103.0, 100.6, 100.2, 99.3, 98.3, 97.7, 94.7, 90.9, 84.8, 81.6, 75.8, 75.8, 75.3, 74.6, 74.0, 69.3, 64.7, 62.1, 56.3, 55.6, 55.5, 52.8, 35.5, 29.3, 28.1, 27.3, 26.5, 25.4, 22.2, 19.3; ESIMS-HR *m/z*: $[M+H]^+$ calcd. for $C_{44}H_{59}N_4O_{19}$ 947.3768, found 947.3758; $[\alpha]^{22}_{D}$ +70.30 (*c* 10.1, $CHCl_3$).

Re-protection of by-product

A solution of **mono-desisopropylidene** (20.7 mg, 22.8 mg) and 2,2-dimethoxypropane (27.9 μL, 228 μmol, 10 equiv.) in acetone (0.8 mL) was treated with BF_3·OEt_2 (0.286 μL, 2.28 μmol, 0.1 equiv.) at 0 °C for 10 min. The reaction was quenched with *sat. aq.* $NaHCO_3$ (1.5 mL), and the resulting mixture was extracted with EtOAc. The organic layer was washed with brine (1.5 mL), dried (Na_2SO_4), filtered and concentrated *in vacuo*. The residue was purified by silica gel column chromatography (ϕ0.7×8 cm; $CHCl_3$/MeOH = 1-2%) to afford **S4** (17.0 mg, 79%) as a white solid.

General procedure B (acylation)

A solution of **S4** (1.0 equiv.) in EtOH (1 mL) was treated with ethylenediamine (10 equiv.), and the mixture was heated at 80 °C for 5-6 h. The reaction mixture was cooled and concentrated *in vacuo* (co-evaporated with toluene×3). The residue was purified by short silica gel column (treated with 0.1% Et_3N; $CHCl_3$/MeOH = 7%) to afford the crude amine. A solution of the amine in CH_2Cl_2 (0.5 mL) was treated sequentially with Et_3N (3.0 equiv.), carboxylic acid (2.0 equiv.), HOAt (1.0 equiv.) and EDCI (2.2 equiv.) at room temperature for 2-3 h. Methanol (0.5 mL) was added to the reaction mixture, which was stirred for 1-3 h. The mixture was concentrated *in vacuo* and co-evaporated with toluene. The residue was purified by silica gel column chromatography to afford amide products

1-{11*S*-6,10-Dideoxy-11-*O*-[2-acetamido-2-deoxy-4,6-*O*-isopropylidene-3-*O*-(methoxymethyl)-α-D-*gluco*-hexopyranosyl]-2,3:8,9-di-*O*-isopropylidene-5-*O*-(methoxymethyl)-10-(4-dimethoxymethylbenzamido)-L-*galacto*-β-D-*allo*-undecodialdo-1,4-furanose-11,7-pyranos-1-yl}uracil (***para*-S5**)

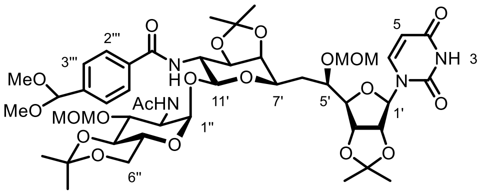

According to general procedure B, **S4** (25.0 mg, 26.4 μmol) and 4-dimethoxymehtylbenzoic acid (8.9 mg, 52.8 μmol) were used to afford ***para*-S5** (21.6 mg, 82% over 2 steps) as a white solid after silica gel column chromatography (ϕ0.7×7.5 cm, hexane/EtOAc = 1/2 → $CHCl_3$/MeOH = 0-2-3%).

^{1}H NMR ($CDCl_3$, 500 MHz) δ 9.05 (br s, 1H, N*H*-3), 7.82 (d, 2H, H-2''', H-6''', *J* = 8.0 Hz), 7.53 (d, 2H, H-3''', H-5''', *J* = 8.0 Hz), 7.25 (d, 1H, H-6, $J_{6,5}$ = 8.0 Hz), 6.64 (d, 1H, N*H*-10', *J* = 6.9 Hz), 5.92 (d, 1H, N*H*-2'', *J* = 9.2 Hz), 5.72 (d, 1H, H-5, $J_{5,6}$ = 8.0 Hz), 5.58 (d, 1H, H-1', $J_{1',2'}$ = 1.7 Hz), 5.43 (s, 1H, C*H*(OMe)$_2$), 5.15 (dd, 1H, H-3', $J_{3',2'}$ = 6.3, $J_{3',4'}$ = 3.4 Hz), 5.03-5.00 (m, 2H, H-11', H-1''), 4.98 (dd, 1H, H-2', $J_{2',3'}$ = 6.3, $J_{2',1'}$ = 1.7 Hz), 4.79 (d, 1H, O-C*H*$_2$-OMe, J_{gem} = 6.8 Hz), 4.76 (d, 1H, O-C*H*$_2$-OMe, J_{gem} = 6.8 Hz), 4.69 (d, 1H, O-C*H*$_2$-OMe, J_{gem} = 6.8 Hz), 4.55 (d, 1H, O-C*H*$_2$-OMe, J_{gem} = 6.8 Hz), 4.49 (dd, 1H, H-9', $J_{9',10'}$ = 8.6, $J_{9',8'}$ = 5.2 Hz), 4.26 (dd, 1H, H-6'', J_{gem} = 10.5, $J_{6'',5''}$ = 5.2 Hz), 4.12 (td, 1H, H-2'', *J* = 9.5, $J_{2'',1''}$ = 3.4 Hz), 4.06-3.93 (m, 5H, H-4', H-5', H-7', H-8', H-5''), 3.75-3.65 (m, 4H, H-10', H-3'', H-4'', H-6''), 3.43 (s, 3H, OMe), 3.29 (s, 3H, OMe), 3.28 (s, 3H, OMe), 3.25 (s, 3H, OMe), 2.26-2.22 (m, 1H, H-6'), 1.66-1.62 (m, 1H, H-6'), 1.60, 1.56, 1.50, 1.49, 1.35, 1.34, 1.21 (each s, 3H×7, Ac and isopropylidene-C*H*$_3$×6); ^{13}C NMR ($CDCl_3$, 100 MHz) δ 170.4, 167.8, 163.5, 150.1, 142.5, 142.3, 133.9, 127.3, 114.6, 110.3, 102.7, 102.0, 101.7, 101.1, 100.1, 98.3, 97.4, 94.2, 90.6, 84.7, 81.2, 75.8, 75.7, 75.7, 75.2, 74.8, 69.1, 64.7, 62.2, 57.4, 56.2, 55.5, 52.6, 52.5, 35.3, 29.3, 28.5, 27.3, 26.4, 25.4, 22.4, 19.3; ESIMS-HR *m*/*z*: $[M+H]^+$ calcd. for $C_{46}H_{67}N_4O_{20}$ 995.4343, found 995.4368; $[\alpha]^{16}_D$ +57.33 (*c* 0.98, $CHCl_3$).

1-{11*S*-6,10-Dideoxy-11-*O*-[2-acetamido-2-deoxy-4,6-*O*-isopropylidene-3-*O*-(methoxymethyl)-α-D-*gluco*-hexopyranosyl]-2,3:8,9-di-*O*-isopropylidene-5-*O*-(methoxymethyl)-10-(3-dimethoxymethylbenzamido)-L-*galacto*-β-D-*allo*-undecodialdo-1,4-furanose-11,7-pyranos-1-yl}uracil (***meta*-S5**)

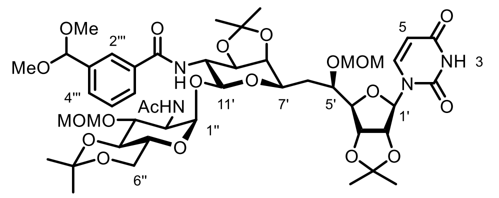

According to general procedure B, **S4** (25.0 mg, 26.4 μmol) and 3-dimethoxymehtylbenzoic acid (8.9 mg, 52.8 μmol) were used to afford ***meta*-S5** (20.5 mg, 78% over 2 steps) as a white solid after silica gel column chromatography (ϕ0.7×7 cm, hexane/EtOAc = 1/2 → $CHCl_3$/MeOH = 0-2-3%).

^{1}H NMR ($CDCl_3$, 500 MHz) δ 9.05 (br s, 1H, N*H*-3), 7.87 (s, 1H, H-2'''), 7.84 (d, 1H, H-6''', *J* = 7.5 Hz), 7.60 (d, 1H, H-4''', *J* = 7.5 Hz), 7.46 (t, 1H, H-5''', *J* = 7.5 Hz), 7.25 (d, 1H, H-6, $J_{6,5}$ = 8.0 Hz), 6.68 (d, 1H, N*H*-10', *J* = 7.5 Hz), 5.92 (d, 1H, N*H*-2'', *J* = 9.2 Hz), 5.72 (d, 1H, H-5, $J_{5,6}$ = 8.0 Hz), 5.58 (d, 1H, H-1', $J_{1',2'}$ = 1.7 Hz), 5.41 (s, 1H, C*H*(OMe)$_2$), 5.16 (dd, 1H, H-3', $J_{3',2'}$ = 6.3, $J_{3',4'}$ = 3.4 Hz), 5.01-5.00 (m, 2H, H-11', H-1''), 4.98 (dd, 1H, H-2', $J_{2',3'}$ = 6.3, $J_{2',1'}$ = 1.7 Hz), 4.79 (d, 1H, O-C*H*$_2$-OMe, J_{gem} = 6.3 Hz), 4.75 (d, 1H, O-C*H*$_2$-OMe, J_{gem} = 6.9 Hz), 4.69 (d, 1H, O-C*H*$_2$-OMe, J_{gem} = 6.3 Hz), 4.55 (d, 1H, O-C*H*$_2$-OMe, J_{gem} = 6.9 Hz), 4.49 (dd, 1H, H-9', $J_{9',10'}$ = 8.6, $J_{9',8'}$ = 5.2 Hz), 4.26 (dd, 1H, H-6'', J_{gem} = 10.9, $J_{6'',5''}$ = 5.2 Hz), 4.12 (td, 1H, H-2'', *J* = 9.5, $J_{2'',1''}$ = 3.4 Hz), 4.06-3.93 (m, 5H, H-4', H-5', H-7', H-8', H-5''), 3.75-3.67 (m, 4H, H-10', H-3'', H-4'', H-6''), 3.44 (s, 3H, OMe), 3.32 (s, 6H, OMe×2), 3.25 (s, 3H, OMe), 2.27-2.22 (m, 1H, H-6'), 1.66-1.62 (m, 1H, H-6'), 1.61, 1.56, 1.50, 1.49, 1.35, 1.34, 1.23 (each s, 3H×7, Ac and isopropylidene-C*H*$_3$×6); ^{13}C NMR ($CDCl_3$, 100 MHz) δ 170.4, 167.9, 163.4, 150.1, 142.4, 139.0, 133.9, 130.6, 128.9, 127.8, 125.3, 114.6, 110.3, 102.8, 102.5, 101.8, 101.1, 100.1, 98.3, 97.4, 94.0, 90.4, 84.6, 81.2, 75.9, 75.7, 75.2, 74.8, 69.1, 64.7, 62.2, 57.3, 56.2, 55.5, 52.9, 52.8, 52.5, 35.3, 29.3, 28.5, 27.3, 26.4, 25.4, 22.4, 19.3; ESIMS-HR *m*/*z*: $[M+H]^+$ calcd. for $C_{46}H_{67}N_4O_{20}$ 995.4343, found 995.4368; $[\alpha]^{17}_D$ +60.16 (*c* 0.91, $CHCl_3$).

General procedure C (deprotection)

A solution of protected analogue (1.0 equiv.) in CH_2Cl_2 (~50 mM) was treated with 80% *aq.* TFA (same volume as CH_2Cl_2) at room temperature for 3 h. The reaction mixture was concentrated *in vacuo*, the resulting solid was washed with hexane and EtOAc (×3) to afford products.

TUNp-CHO

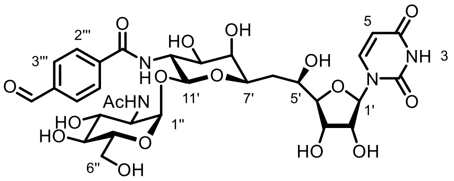

According to general procedure C, ***para*-S5** (20.0 mg, 20.1 μmol) was used to afford **TUNp-CHO** (15.2 mg, quant.) as a white solid.

^{1}H NMR (D_2O, 400 MHz) δ 10.06 (s, 1H, C*H*O), 8.06 (d, 2H, H-3''', H-5''', J = 8.2 Hz), 7.92 (d, 2H, H-2''', H-6''', J = 8.2 Hz), 7.85 (d, 1H, H-6, $J_{6,5}$ = 8.2 Hz), 5.92 (d, 1H, H-5, $J_{5,6}$ = 8.2 Hz), 5.91 (d, 1H, H-1', $J_{1',2'}$ = 5.4 Hz), 5.14 (d, 1H, H-1'', $J_{1'',2''}$ = 3.6 Hz), 4.88 (d, 1H, H-11', $J_{11',10'}$ = 8.2 Hz), 4.34 (t, 1H, H-2', J = 5.4 Hz), 4.31 (dd, 1H, H-3', $J_{3',2'}$ = 5.4, $J_{3',4'}$ = 3.6 Hz), 4.24 (dd, 1H, H-10', $J_{10',9'}$ = 10.0, $J_{10',11'}$ = 8.2 Hz), 4.04-4.01 (m, 2H, H-4', H-5'), 3.97-3.90 (m, 3H, H-7', H-9', H-5''), 3.87-3.74 (m, 5H, H-8', H-2'', H-3'', H-6'', H-6''), 3.51 (dd, 1H, H-4'', J = 10.0, J = 9.1 Hz), 2.07 (m, 1H, H-6'), 1.68 (m, 1H, H-6'), 1.27 (s, 3H, Ac); ^{13}C NMR (D_2O, 100 MHz: 1% DMSO-d_6 as internal standard) δ 197.1, 175.5, 171.8, 167.7, 153.4, 143.5, 140.6, 139.9, 132.0, 129.6, 104.2, 101.8, 100.5, 90.2, 88.9, 75.1, 74.3, 73.1, 72.4, 72.0, 71.7, 71.3, 70.7, 68.7, 62.1, 55.1, 55.0, 35.2, 23.0; ESIMS-HR *m/z*: $[M+H]^+$ calcd. for $C_{31}H_{41}N_4O_{17}$ 741.2461, found 741.2448; $[\alpha]^{21}_D$ +46.90 (c 0.96, H_2O).

TUNm-CHO

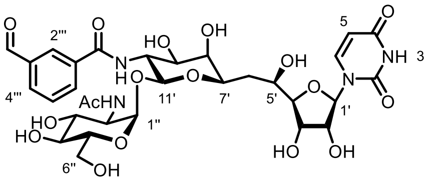

According to general procedure C, ***meta*-S5** (19.5 mg, 19.6 μmol) was used to afford **TUNm-CHO** (15.0 mg, quant., aldehyde form >95%) as a white solid.

^{1}H NMR (D_2O, 400 MHz) δ 10.0 (s, 1H, C*H*O), 8.26 (s, 1H, H-2'''), 8.17 (d, 1H, H-4''', J = 7.7 Hz), 8.06 (d, 1H, H-6''', J = 7.7 Hz), 7.85 (d, 1H, H-6, $J_{6,5}$ = 8.2 Hz), 7.75 (t, 1H, H-5''', J = 7.7 Hz), 5.92 (d, 1H, H-5, $J_{5,6}$ = 8.2 Hz), 5.91 (d, 1H, H-1', $J_{1',2'}$ = 5.4 Hz), 5.14 (d, 1H, H-1'', $J_{1'',2''}$ = 3.2 Hz), 4.88 (d, 1H, H-11', $J_{11',10'}$ = 8.6 Hz), 4.34 (t, 1H, H-2', J = 5.4 Hz), 4.30 (dd, 1H, H-3', $J_{3',2'}$ = 5.4, $J_{3',4'}$ = 3.2 Hz), 4.25 (dd, 1H, H-10', $J_{10', 9'}$ = 10.9, $J_{10',11'}$ = 8.6 Hz), 4.04-4.00 (m, 2H, H-4', H-5'), 3.97-3.91 (m, 3H, H-7', H-9', H-5''), 3.87-3.74 (m, 5H, H-8', H-2'', H-3'', H-6'', H-6''), 3.50 (dd, 1H, H-4'', J = 10.0, J = 9.1 Hz), 2.06 (br t, 1H, H-6', J = 12.5 Hz), 1.68 (br t, 1H, H-6', J = 12.5 Hz), 1.24 (s, 3H, Ac); ^{13}C NMR (D_2O, 100 MHz: 1% DMSO-d_6 as internal standard) δ 196.8, 175.4, 171.6, 167.7, 153.4, 143.5, 137.7, 136.0, 135.4, 135.1, 131.6, 129.9, 104.2, 101.9, 100.5, 90.2, 88.9, 75.1, 74.3, 73.1, 72.5, 71.9, 71.7, 71.3, 70.7, 68.7, 62.1, 55.1, 55.0, 35.2, 22.9; ESIMS-HR *m/z*: $[M+H]^+$ calcd. for $C_{31}H_{41}N_4O_{17}$ 741.2461, found 741.2448; $[\alpha]^{21}_D$ +41.05 (c 0.57, H_2O).

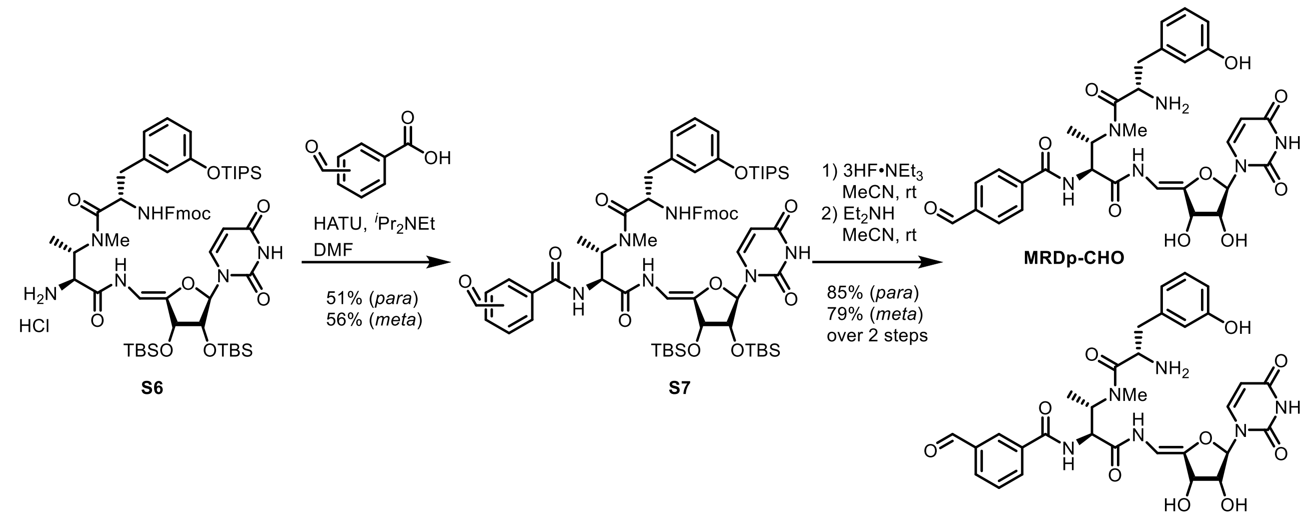

Scheme S3. Synthesis of **MRDp-CHO** and **MRDm-CHO**.

Compound ***para*-S7**

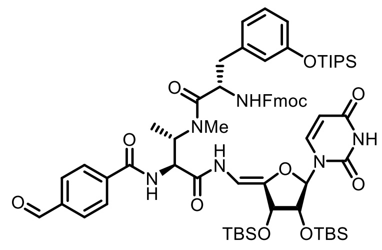

A solution of **S6**[9] (20.0 mg, 16.8 µmol), *p*-formylbenzoic acid (6.3 mg, 41.7 µmol), and ^{i}Pr$_2$NEt (18.7 µL, 110.7 µmol) in DMF (400 µL) was treated with HATU (15.3 mg, 40.3 µmol) at room temperature for 1 h. The mixture was partitioned between EtOAc and 1 M *aq*. HCl. The organic layer was washed with 1 M *aq*. HCl, *sat*. *aq*. NaHCO$_3$ and brine, dried (Na$_2$SO$_4$), filtered and concentrated *in vacuo*. The residue was purified by preparative TLC (MeOH/CHCl$_3$ = 3%) to afford ***para*-S7** (10.8 mg, 8.6 µmol, 51%) as a white solid.

^{1}H NMR (DMSO-d_6, 400 MHz) δ 11.55 (s, 1H, N*H*-3), 10.07 (s, 1H, C*H*O), 9.42 (d, 1H, N*H*-5', $J_{\mathrm{N}H\text{-}5',\,5'}$ = 10.2 Hz), 8.97 (d, 1H, N*H*-α-DABA, $J_{\mathrm{N}H\text{-α-DABA, α-DABA}}$ = 9.2 Hz), 8.07 (d, 2H, H-2", H-6", $J_{2'',\,3''}$ = 8.2 Hz, $J_{6'',\,5''}$ = 8.2 Hz), 7.98 (d, 2H, H-3", H-5", $J_{3'',\,2''}$ = 8.2 Hz, $J_{5'',\,6''}$ = 8.2 Hz), 7.87 (d, 1H, N*H*-*m*Tyr, $J_{\mathrm{N}H\text{-}m\mathrm{Tyr},\,\alpha\text{-}m\mathrm{Tyr}}$ = 8.0 Hz), 7.87-7.24 (m, 9H, Ar-*H* of Fmoc group, H-6), 7.16 (dd, 1H, H-5-*m*Tyr, $J_{5\text{-}m\mathrm{Tyr},\,4\text{-}m\mathrm{Tyr}} = J_{5\text{-}m\mathrm{Tyr},\,6\text{-}m\mathrm{Tyr}}$ = 7.9 Hz), 6.91 (d, 1H, H-4-*m*Tyr, $J_{4\text{-}m\mathrm{Tyr},\,5\text{-}m\mathrm{Tyr}}$ = 7.9 Hz), 6.89 (br s, 1H, H-2-*m*Tyr), 6.69 (dd, 1H, H-6-*m*Tyr, $J_{6\text{-}m\mathrm{Tyr},\,5\text{-}m\mathrm{Tyr}}$ = 7.9 Hz, $J_{6\text{-}m\mathrm{Tyr},\,4\text{-}m\mathrm{Tyr}}$ = 2.2 Hz), 6.19 (d, 1H, H-5', $J_{5',\,\mathrm{N}H\text{-}5'}$ = 10.2 Hz), 6.18 (d, 1H, H-1', $J_{1',\,2'}$ = 7.3 Hz), 5.48 (d, 1H, H-5, $J_{5,\,6}$ = 8.4 Hz), 5.22 (dd, 1H, H-β-DABA, $J_{\text{β-DABA, α-DABA}}$ = 10.4 Hz, $J_{\text{β-DABA, γ-DABA}}$ = 6.9 Hz), 4.76 (dd, 1H, H-α-DABA, $J_{\text{α-DABA, N}H\text{-α-DABA}}$ = 9.2 Hz, $J_{\text{α-DABA, β-DABA}}$ = 10.4 Hz), 4.52-4.48 (m, 1H, H-α-*m*Tyr), 4.48 (d, 1H, H-3', $J_{3',\,2'}$ = 4.2 Hz), 4.34 (dd, 1H, H-2', $J_{2',\,1'}$ = 7.3 Hz, $J_{3',\,2'}$ = 4.2 Hz), 4.14-4.09 (m, 3H, fluorenyl-*H*, fluorenyl-C*H*$_2$O), 3.07 (s, 3H, NC*H*$_3$), 2.78-2.65 (m, 1H, H-β-*m*Tyr), 1.24-1.14 (m, 6H, Si[C*H*(CH$_3$)$_2$]$_3$, H-γ-DABA), 1.05-1.00 (m, 18H, Si[CH(C*H*$_3$)$_2$]$_3$), 0.85 (s, 9H, Me$_2$SiC(C*H*$_3$)$_3$), 0.77 (s, 9H, Me$_2$SiC(C*H*$_3$)$_3$), 0.05 (s, 3H, ^{t}BuSi(C*H*$_3$)$_2$), 0.02 (s, 3H, ^{t}BuSi(C*H*$_3$)$_2$), −0.03 (s, 3H, ^{t}BuSi(C*H*$_3$)$_2$), −0.14 (s, 3H, ^{t}BuSi(C*H*$_3$)$_2$); ^{13}C NMR (DMSO-d_6, 100 MHz) δ 192.8, 172.4, 167.3, 165.5, 162.6, 156.0, 155.3, 150.8, 143.9, 143.6, 140.8, 140.3, 140.0, 138.6, 138.1, 129.4, 128.2, 127.6, 127.1, 125.5, 125.3, 121.9, 120.7, 120.1, 117.6, 102.7, 100.4, 87.1, 79.2, 73.7, 70.8, 65.6, 56.8, 53.2, 48.6, 46.6, 36.3, 29.3, 25.7, 25.5, 17.8, 17.5, 14.8, 12.0, −4.2, −4.7, −4.8, −5.4; ESIMS-LR *m*/*z* 1279.2 [(M+Na)$^+$]; ESIMS-HR calcd. for $C_{67}H_{93}N_6O_{12}Si_3$ 1257.6154, found 1257.6155; $[\alpha]^{18}_{\mathrm{D}}$ 68.50 (*c* 1.08, CHCl$_3$).

Compound ***meta*-S7**

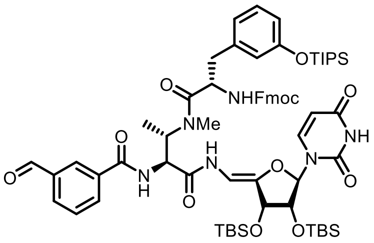

A solution of **S6**[9] (40.0 mg, 33.6 µmol), *m*-formylbenzoic acid (12.6 mg, 83.9 µmol) and $^{i}Pr_2NEt$ (37.4 µL, 221.5 µmol) in DMF (400 µL) was treated with HATU (29.3 mg, 77.2 µmol) at room temperature for 1 h. The mixture was partitioned between EtOAc and 1 M *aq*. HCl. The organic layer was washed with 1 M *aq*. HCl, *sat. aq*. $NaHCO_3$ and brine, dried (Na_2SO_4), filtered and concentrated *in vacuo*. The residue was purified by preparative TLC ($MeOH/CHCl_3$ = 3%) to afford ***meta*-S7** (23.7 mg, 18.8 µmol, 56%) as a white solid.

^{1}H NMR (DMSO-d_6, 400 MHz) δ 11.54 (s, 1H, N*H*-3), 10.06 (s, 1H, C*H*O), 9.45 (d, 1H, N*H*-5', $J_{NH\text{-}5', 5'}$ = 10.3 Hz), 9.01 (d, 1H, N*H*-α-DABA, $J_{NH\text{-}\alpha\text{-DABA}, \alpha\text{-DABA}}$ = 9.2 Hz), 8.45 (s, 1H, H-2''), 8.20 (d, 1H, H-4'', $J_{4'', 5''}$ = 7.6 Hz), 8.10 (d, 1H, H-6'', $J_{6'', 5''}$ = 7.6 Hz), 7.86 (d, 1H, N*H*-*m*Tyr, $J_{NH\text{-}m\text{Tyr}, \alpha\text{-}m\text{Tyr}}$ = 7.6 Hz), 7.71 (dd, 1H, H-5'', $J_{5'', 4''}$ = 7.6 Hz, $J_{5'', 6''}$ = 7.6 Hz), 7.86-7.24 (m, 9H, Ar-*H* of Fmoc group, H-6), 7.16 (dd, 1H, H-5-*m*Tyr, $J_{5\text{-}m\text{Tyr}, 4\text{-}m\text{Tyr}}$ = $J_{5\text{-}m\text{Tyr}, 6\text{-}m\text{Tyr}}$ = 7.9 Hz), 6.92 (d, 1H, H-4-*m*Tyr, $J_{4\text{-}m\text{Tyr}, 5\text{-}m\text{Tyr}}$ = 7.9 Hz), 6.89 (br s, 1H, H-2-*m*Tyr), 6.69 (dd, 1H, H-6-*m*Tyr, $J_{6\text{-}m\text{Tyr}, 5\text{-}m\text{Tyr}}$ = 7.9 Hz, $J_{6\text{-}m\text{Tyr}, 4\text{-}m\text{Tyr}}$ = 2.5 Hz), 6.20 (d, 1H, H-5', $J_{5', NH\text{-}5'}$ = 10.3 Hz), 6.20 (d, 1H, H-1', $J_{1', 2'}$ = 7.6 Hz), 5.41 (d, 1H, H-5, $J_{5, 6}$ = 8.1 Hz), 5.24 (dd, 1H, H-β-DABA, $J_{\beta\text{-DABA}, \alpha\text{-DABA}}$ = 10.3 Hz, $J_{\beta\text{-DABA}, \gamma\text{-DABA}}$ = 6.7 Hz), 4.78 (dd, 1H, H-α-DABA, $J_{\alpha\text{-DABA}, \beta\text{-DABA}}$ = 10.3 Hz, $J_{\alpha\text{-DABA}, NH\text{-}\alpha\text{-DABA}}$ = 9.2 Hz), 4.52-4.50 (m, 1H, H-α-*m*Tyr), 4.48 (d, 1H, H-3', $J_{3', 2'}$ = 4.2 Hz), 4.32 (dd, 1H, H-2', $J_{2', 1'}$ = 7.3 Hz, $J_{3', 2'}$ = 4.2 Hz), 4.14-4.09 (m, 3H, fluorenyl-*H*, fluorenyl-CH_2O), 3.08 (s, 3H, NCH_3), 2.78-2.66 (m, 1H, H-β-*m*Tyr), 1.26-1.13 (m, 6H, Si[C*H*(CH_3)$_2$]$_3$, H-γ-DABA), 1.03-1.02 (m, 18H, Si[CH(CH_3)$_2$]$_3$), 0.84 (s, 9H, $Me_2SiC(CH_3)_3$), 0.77 (s, 9H, $Me_2SiC(CH_3)_3$), 0.05 (s, 3H, $^{t}BuSi(CH_3)_2$), 0.02 (s, 3H, $^{t}BuSi(CH_3)_2$), −0.04 (s, 3H, $^{t}BuSi(CH_3)_2$), −0.15 (s, 3H, $^{t}BuSi(CH_3)_2$); ^{13}C NMR (DMSO-d_6, 100 MHz) δ 192.7, 172.4, 167.4, 165.1, 162.5, 156.0, 155.3, 150.8, 143.8, 143.6, 140.7, 140.2, 140.0, 136.3, 134.4, 133.3, 133.0, 129.4, 129.3, 127.6, 127.1, 125.5, 125.3, 121.9, 120.7, 120.1, 117.6, 102.7, 100.4, 86.9, 79.2, 73.8, 70.8, 65.6, 56.8, 53.2, 48.6, 46.6, 36.4, 29.3, 25.7, 25.5, 17.8, 17.5, 14.8, 12.0, −4.2, −4.7, −4.8, −5.4; ESIMS-LR *m/z* 1279.2 [$(M+Na)^+$]; ESIMS-HR calcd. for $C_{67}H_{93}N_6O_{12}Si_3$ 1257.6154, found 1257.6155; $[\alpha]^{17}_{D}$ 82.05 (*c* 1.10, $CHCl_3$).

MRDp-CHO

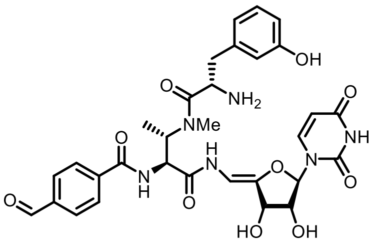

A solution of ***para*-S7** (10.8 mg, 8.6 µmol) in MeCN (400 µL) was treated with $3HF\cdot NEt_3$ (55.9 µL, 343.4 µmol) at room temperature for 3 days. Trimethylsilanol (89.0 µL, 796.7 µmol) was added to the reaction mixture, which was further stirred for 1 h. The mixture was concentrated *in vacuo*, and the residue was treated with 20% Et_2NH/MeCN (2.0 mL) at room temperature for 1 h. The mixture was concentrated *in vacuo*, and the residue was triturated with Et_2O. The solvent was removed by decantation, and the resulting precipitate was purified by ODS column chromatography (MeCN/H_2O = 0-30%, containing 0.1% TFA) to afford **MRDp-CHO** (5.6 mg, 7.3 µmol, 85%) as a white solid.

^{1}H NMR (DMSO-d_6, 400 MHz, 1:1 mixture of rotamers, selected data for the major rotamer) δ 11.51 (s, 1H, N*H*-3), 10.07 (s, 1H, C*H*O), 9.69 (d, 1H, N*H*-5', $J_{NH\text{-}5', 5'}$ = 10.0 Hz), 9.60 (s, 1H, O*H*-*m*Tyr), 9.08 (d, 1H, N*H*-α-DABA, $J_{NH\text{-}\alpha\text{-DABA}, \alpha\text{-DABA}}$ = 8.7 Hz), 8.05-7.97 (m, 6H, H-2'', H-3'', H-5'', H-6'', NH_2-*m*Tyr), 7.18 (dd, 1H, H-5-*m*Tyr, $J_{5\text{-}m\text{Tyr}, 4\text{-}m\text{Tyr}}$ = $J_{5\text{-}m\text{Tyr}, 6\text{-}m\text{Tyr}}$ = 7.9 Hz), 6.85 (d, 1H, H-6, $J_{6, 5}$ = 8.2 Hz), 6.74-6.63 (m, 3H, H-2-*m*Tyr, H-4-*m*Tyr, H-6-*m*Tyr), 6.10

(d, 1H, H-1', $J_{1',2'}$ = 6.6 Hz), 6.08 (d, 1H, H-5', $J_{5',NH-5'}$ = 10.0 Hz), 5.66 (d, 1H, O*H*-2', $J_{OH-2',2'}$ = 6.2 Hz), 5.49 (d, 1H, O*H*-3', $J_{OH-3',3'}$ = 4.9 Hz), 5.41 (d, 1H, H-5, $J_{5,6}$ = 8.2 Hz), 5.21 (dd, 1H, H-β-DABA, $J_{\beta\text{-DABA},\alpha\text{-DABA}}$ = 10.5 Hz, $J_{\beta\text{-DABA},\gamma\text{-DABA}}$ = 7.0 Hz), 4.81 (dd, 1H, H-α-DABA, $J_{\alpha\text{-DABA},\beta\text{-DABA}}$ = 10.5 Hz, $J_{\alpha\text{-DABA},NH\text{-}\alpha\text{-DABA}}$ = 8.7 Hz), 4.47 (d, 1H, H-α-*m*Tyr, $J_{\alpha\text{-}m\text{Tyr},\beta\text{-}m\text{Tyr}}$ = 9.8 Hz), 4.36-4.33 (m, 1H, H-3'), 3.90 (ddd, 1H, H-2', $J_{2',1'}$ = 6.6 Hz, $J_{2',OH-2'}$ = 6.2 Hz, $J_{2',3'}$ = 5.9 Hz), 3.06 (s, 3H, NCH_3), 2.97-2.46 (m, 2H, H-β-*m*Tyr), 1.19 (d, 3H, H-γ-DABA, $J_{\gamma\text{-DABA},\beta\text{-DABA}}$ = 6.8Hz); ^{13}C NMR (D_2O, 100 MHz) δ 195.9, 170.6, 169.7, 168.9, 166.0, 164.0, 163.7, 157.5, 151.2, 144.3, 141.8, 137.0, 136.2, 134.4, 132.1, 131.0, 128.7, 121.9, 118.9, 116.7, 116.4, 115.9, 103.5, 100.1, 73.0, 70.6, 69.2, 57.0, 53.3, 37.0, 30.5, 14.7; ESIMS-LR *m*/*z* 651.7 [$(M+H)^+$]; ESIMS-HR calcd. for $C_{31}H_{35}N_6O_{10}$ 651.2409, found 651.2386; $[\alpha]^{19}_{D}$ 30.10 (*c* 0.80, MeOH).

MRDm-CHO

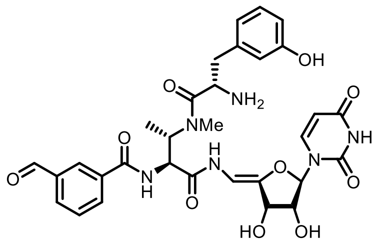

A solution of ***meta*-S7** (34.7 mg, 27.6 µmol) in MeCN (300 µL) was treated with 3HF·NEt_3 (180.0 µL, 1.1 mmol) at room temperature for 3 days. Trimethylsilanol (583.0 µL, 5.3 mmol) was added to the reaction mixture, which was further stirred for 4 h. The mixture was concentrated *in vacuo,* and the residue was treated with 20% Et_2NH/MeCN (2.0 mL) at room temperature for 1 h. The mixture was concentrated *in vacuo,* and the residue was triturated with Et_2O. The solvent was removed by decantation, and the resulting precipitate was purified by ODS column chromatography (MeCN/H_2O = 0-30%, containing 0.1% TFA) to afford **MRDm-CHO** (16.7 mg, 21.8 µmol, 79%) as a white solid. ^{1}H NMR (DMSO-d_6, 400 MHz, 6:4 mixture of rotamers, selected data for the major rotamer) δ 11.51 (s, 1H, N*H*-3), 10.07 (s, 1H, C*H*O), 9.69 (d, 1H, N*H*-5', $J_{NH-5',5'}$ = 9.4 Hz), 9.60 (s, 1H, O*H*-*m*Tyr), 9.13 (d, 1H, N*H*-α-DABA, $J_{NH\text{-}\alpha\text{-DABA},\alpha\text{-DABA}}$ = 9.4 Hz), 8.43 (s, 1H, H-2''), 8.21-8.08 (m, 4H, H-4'', H-6'', NH_2-*m*Tyr), 7.18 (dd, 1H, H-5-*m*Tyr, $J_{5\text{-}m\text{Tyr},4\text{-}m\text{Tyr}}$ = $J_{5\text{-}m\text{Tyr},6\text{-}m\text{Tyr}}$ = 7.9 Hz), 6.85 (d, 1H, H-6, $J_{6,5}$ = 8.1 Hz), 6.77-6.65 (m, 3H, H-2-*m*Tyr, H-4-*m*Tyr, H-6-*m*Tyr), 6.11 (d, 1H, H-1', $J_{1',2'}$ = 7.6 Hz), 6.08 (d, 1H, H-5', $J_{5',NH-5'}$ = 9.9 Hz), 5.70 (br s, 1H, O*H*-2'), 5.53 (br s, 1H, O*H*-3'), 5.40 (d, 1H, H-5, $J_{5,6}$ = 8.1 Hz), 5.21 (dd, 1H, H-β-DABA, $J_{\beta\text{-DABA},\alpha\text{-DABA}}$ = 10.3 Hz, $J_{\beta\text{-DABA},\gamma\text{-DABA}}$ = 6.8 Hz), 4.83 (dd, 1H, H-α-DABA, $J_{\alpha\text{-DABA},\beta\text{-DABA}}$ = 10.3 Hz, $J_{\alpha\text{-DABA},NH\text{-}\alpha\text{-DABA}}$ = 9.4 Hz), 4.45 (d, 1H, H-α-*m*Tyr, $J_{\alpha\text{-}m\text{Tyr},\beta\text{-}m\text{Tyr}}$ = 8.5 Hz), 4.36-4.33 (m, 1H, H-3'), 3.90 (ddd, 1H, H-2', $J_{2',OH-2'}$ = 8.0 Hz, $J_{2',1'}$ = 7.6 Hz, $J_{2',3'}$ = 5.8 Hz), 3.04 (s, 3H, NCH_3), 2.97-2.50 (m, 2H, H-β-*m*Tyr), 1.17 (d, 3H, H-γ-DABA, $J_{\gamma\text{-DABA},\beta\text{-DABA}}$ = 6.8Hz); ^{13}C NMR (D_2O, 100 MHz) δ 195.5, 170.6, 169.7, 168.9, 166.0, 164.0, 163.7, 157.5, 151.2, 144.3, 141.8, 137.0, 136.2, 134.4, 132.1, 131.0, 128.7, 121.9, 118.9, 116.7, 116.4, 115.9, 103.5, 100.1, 73.1, 70.6, 69.0, 57.3, 53.1, 50.8, 49.9, 37.0, 30.6, 14.7; ESIMS-LR *m*/*z* 651.7 [$(M+H)^+$]; ESIMS-HR calcd. for $C_{31}H_{35}N_6O_{10}$ 651.2409, found 651.2386; $[\alpha]^{20}_{D}$ 27.44 (*c* 0.83, MeOH).

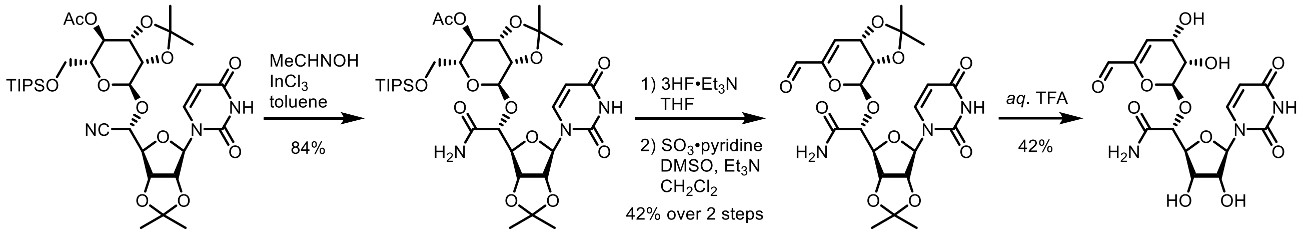

S8 **S9** **S10** **CAP-CHO**

Scheme S4. Synthesis of **CAP-CHO**.

Compound **S9**

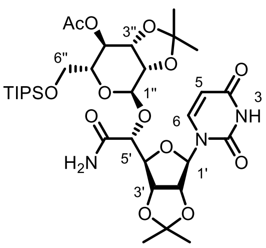

A mixture of **S8**[18] (2.32 g, 3.27 mmol), acetaldoxime (3.86 g, 65.4 mmol) and indium (III) chloride anhydrous (717 mg, 3.27 mmol) in toluene (5 mL) was stirred at room temperature for 18 h. The mixture was concentrated *in vacuo*. The residue was purified by silica gel chromatography (50 g, MeOH/$CHCl_3$ = 3%) to afford **S9** (1.99 g, 2.73 mmol, 84%) as a white amorphous solid.

^{1}H NMR ($CDCl_3$, 400 MHz) δ 8.08 (br s, 1H, N*H*-3), 7.68 (d, 1H, H-6, $J_{6,5}$ = 8.0 Hz), 6.67 (br s, 1H, CONH_2-5'), 5.96 (d, 1H, H-1', $J_{1',2'}$ = 3.2 Hz), 5.71 (dd, 1H, H-5, $J_{5,6}$ = 8.0 Hz), 5.61 (br s, 1H, CONH_2-5'), 5.19 (t, 1H, H-4'', J = 6.2 Hz), 4.99 (d, 1H, H-1'', $J_{1'',2''}$ = 5.0 Hz), 4.95 (dd, 1H, H-3', $J_{3',2'}$ = 6.4, $J_{3',4'}$ = 3.0 Hz), 4.72 (dd, 1H, H-2', $J_{2',3'}$ = 6.4, $J_{2',1'}$ = 3.2 Hz), 4.64 (t, 1H, H-4', J = 3.0 Hz), 4.46 (d, 1H, H-5', $J_{5',4'}$ = 3.0 Hz), 4.32 (t, 1H, H-3'', J = 6.2 Hz), 4.08 (dd, 1H, H-2'', $J_{2'',3''}$ = 6.2, $J_{2'',1''}$ = 5.0 Hz), 3.88-3.78 (m, 3H, H-5'', H-6''), 2.11 (s, 3H, OAc), 1.59 (s, 3H, Me), 1.52 (s, 3H, Me), 1.37 (s, 3H, Me), 1.36 (s, 3H, Me), 1.11-1.05 (m, 21H, Si(*i*-Pr)$_3$); ^{13}C NMR ($CDCl_3$, 100 MHz) δ 169.7, 163.0, 150.1, 140.6, 115.7, 115.6, 110.5, 103.3, 96.1, 92.1, 83.9, 83.8, 80.6, 75.8, 75.0, 71.3, 69.1, 64.4, 62.5, 27.5, 27.2, 26.4, 25.4, 21.0, 18.0, 12.0; ESIMS-HR *m/z*: $[M+Na]^+$ calcd for $C_{33}H_{53}N_3O_{13}SiNa$ 750.3240, found 750.3264; $[\alpha]^{20}_D$ +29.17 (*c* 0.13, $CHCl_3$).

Compound **S10**

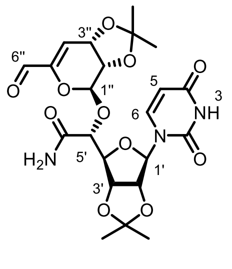

A mixture of **S9** (1.76 g, 2.42 mmol) and triethylamine trihydrofluoride (7.59 mL, 46.6 mmol) in THF (50 mL) was stirred at 80 °C for 3 h. After cooled to 0 °C, the reaction was quenched with silica gel (10 g). The mixture was concentrated *in vacuo*. The residue was purified by silica gel chromatography (4 g, MeOH/$CHCl_3$ = 4%) to afford the crude primary alcohol (2.32 g) as white amorphous. A mixture of the crude primary alcohol (190 mg), dimethyl sulfoxide (141 μL, 1.98 mmol) and triethylamine (0.5 mL) in CH_2Cl_2 (2.5 mL) was treated with sulfur trioxide-pyridine complex (315 mg, 1.98 mmol) at room temperature for 2 h. After the reaction was quenched with water (0.3 mL), the mixture was concentrated *in vacuo*. The residue was purified by silica gel chromatography (8 g, MeOH/$CHCl_3$ = 3%) to afford **S10** (42.1 mg, 0.0826 mmol, calcd. 42% over 2 steps) as a white amorphous solid.

^{1}H NMR ($CDCl_3$, 400 MHz) δ 9.32 (s, 1H, C*H*O-5''), 8.13 (br s, 1H, N*H*-3), 7.60 (d, 1H, H-6, $J_{6,5}$ = 8.1 Hz), 7.11 (br s, 1H, CONH_2-5'), 6.19 (d, 1H, H-4'', $J_{4'',3''}$ = 4.3 Hz), 5.94 (d, 1H, H-1', $J_{1',2'}$ = 3.1 Hz), 5.73 (d, 1H, H-5, $J_{5,6}$ = 8.1 Hz), 5.65 (br s, 1H, CONH_2-5'), 5.16 (dd, 1H, H-3', $J_{3',2'}$ = 6.3, $J_{3',4'}$ = 2.8 Hz), 4.83 (dd, 1H, H-3'', $J_{3'',2''}$ = 6.3, $J_{3'',4''}$ = 4.3 Hz), 4.80 (t, 1H, H-4', J = 2.8 Hz), 4.77 (dd, 1H, H-2', $J_{2',3'}$ = 6.3, $J_{2',1'}$ = 3.1 Hz), 4.60-4.58 (m, 2H, H-5', H-1''),

4.06 (dd, 1H, H-2", $J_{2'',1''}$ = 8.1, $J_{2'',3''}$ = 6.3 Hz), 1.61 (s, 3H, Me), 1.51 (s, 3H, Me), 1.46 (s, 3H, Me), 1.41 (s, 3H, Me); ^{13}C NMR ($CDCl_3$, 100 MHz) δ 185.2, 170.8, 163.7, 150.5, 150.3, 140.9, 116.7, 114.6, 110.8, 102.5, 100.1, 92.5, 85.5, 84.6, 81.0, 79.8, 73.6, 69.0, 27.7, 27.3, 25.5, 25.3; ESIMS-HR *m/z*: $[M+H]^+$ calcd for $C_{22}H_{28}N_3O_{11}$ 510.1718, found 510.1726; $[\alpha]^{20}_D$ +75.08 (*c* 0.17, $CHCl_3$).

CAP-CHO

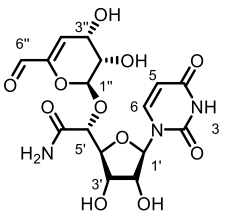

A mixture of **S10** (28.0 mg, 0.0550 mmol) and 80% *aq.* TFA (1 mL) was stirred at room temperature for 30 min. The mixture was concentrated *in vacuo*. The residue was purified by Hi-Flash reverse phase silica gel column chromatography (Sep-Pak C18, H_2O) to afford **CAP-CHO** as white amorphous solid (10.0 mg, 0.0233 mmol, 42%).

^{1}H NMR (DMSO-d_6, 400 MHz) δ 11.31 (br s, 1H, N*H*-3), 9.18 (s, 1H, C*H*O-5"), 7.70 (d, 1H, H-6, $J_{6,5}$ = 8.2 Hz), 7.61 (br s, 1H, CONH_2-5'), 7.51 (br s, 1H, CONH_2-5'), 6.01-6.00 (m, 1H, H-4"), 5.72 (d, 1H, H-1', $J_{1',2'}$ = 5.5 Hz), 5.65 (d, H-5, $J_{5,6}$ = 8.2 Hz), 5.06 (d, 1H, H-1", $J_{1'',2''}$ = 3.2 Hz), 4.36-4.34 (m, 1H, H-3"), 4.28 (d, 1H, H-5', $J_{5',4'}$ = 1.4 Hz), 4.25-4.22 (m, 1H, H-4'), 4.06-4.04 (m, 1H, H-2"), 3.80-3.78 (m, 1H, H-2'), 3.72-3.70 (m, 1H, H-3'); ^{13}C NMR (DMSO-d_6, 100 MHz) δ 187.3, 170.1, 163.1, 150.6, 147.6, 140.0, 125.4, 101.9, 99.1, 87.3, 84.3, 76.2, 73.9, 70.4, 65.2, 61.9; ESIMS-HR *m/z*: $[M+H]^+$ calcd for $C_{16}H_{20}N_3O_{11}$ 430.1092, found 430.1104; $[\alpha]^{20}_D$ +165.48 (*c* 0.13, MeOH).

General procedure for the synthesis of BZ and PA hydrazides

A mixture of ester (1.0 mmol) in EtOH (1.0 mL) was treated with $NH_2NH_2 \cdot H_2O$ (122 μL, 2.5 mmol) at 100 °C for 12 h in a sealed tube. After the completion of the reaction, the reaction mixture was concentrated *in vacuo*. The precipitate was filtered and dried to afford **BZ2-22**, **24-36**, **38-47**, and **PA48-54**.

Hydrazide **BZ2**[19)], **BZ3**[20)], **BZ4**[21)], **BZ5**[22)], **BZ6**[23)], **BZ7**[23)], **BZ8**[23)], **BZ9**[24)], **BZ10**[25)], **BZ11**[26)], **BZ12**[27)], **BZ13**[28)], **BZ14**[29)], **BZ15**[30)], **BZ16**[30)], **BZ17**[31)], **BZ18**[32)], **BZ19**[33)], **BZ20**[28)], **BZ21**[34)], **BZ22**[35)], **BZ25**[36)], **BZ26**[37)], **BZ27**[38)], **BZ28**[39)], **BZ29**[40)], **BZ30**[41)], **BZ32**[42)], **BZ33**[43)], **BZ34**[44)], **BZ35**[45)], **BZ36**[46)], **BZ37**[47)], **BZ40**[48)], **BZ41**[49)], **BZ42**[50)], **BZ43**[51)], **BZ44**[52)], **BZ45**[53)], **BZ46**[54)], **BZ47**[55)], **PA48**[56)], **PA49**[57)], **PA50**[58)], **PA51**[59)], **PA52**[60)], and **PA53**[61)] are known compound. Hydrazides **BZ1**, **BZ23**, **BZ24** and **PA54** are commercially available.

1*H*-Imidazole-2-carbohydrazide (**BZ31**)

Compound **BZ31** (111.6 mg, 0.92 mmol, 92% as a brown solid) was prepared from ethyl 2-imidazolecarboxylate (140 mg, 1.0 mmol) and $NH_2NH_2 \cdot H_2O$ (122 μL) in EtOH (1.0 mL) as described above.

^{1}H NMR (DMSO-d_6, 400 MHz) δ 9.56 (s, 1H, NH), 7.24 (s, 1H, Ph), 7.01 (s, 1H, Ph), 4.43 (s, 2H, NH_2); ^{13}C NMR (DMSO-d_6, 100 MHz) δ 158.1, 140.3; ESIMS-LR *m/z* 127.1 [$(M+H)^+$].

ss

5-Aminobenzofuran-2-carbohydrazide (**BZ38**)

Compound **BZ39** (135.8 mg, 0.71 mmol, 71% as a brown solid) was prepared from ethyl 5-aminobenzofuran-2-carboxylate (205 mg, 1.0 mmol) and $NH_2NH_2 \cdot H_2O$ (122 μL) in EtOH (1.0 mL) as described above.

^1H NMR (DMSO-d_6, 400 MHz) δ 9.84 (s, 1H, NH), 7.27 (d, 1H, H-7, $J_{7,6}$ = 8.8 Hz), 7.25 (d, 1H, H-3, J = 0.8 Hz), 6.75 (d, 1H, H-4, $J_{4,6}$ = 2.2 Hz), 6.72 (dd, 1H, H-6, $J_{6,7}$ = 8.8, $J_{6,4}$ = 2.2 Hz), 4.97 (s, 2H, NH_2), 4.51 (s, 2H, NH_2); ^{13}C NMR (DMSO-d_6, 400 MHz) δ 158.3, 148.2, 147.6, 145.3, 127.8, 115.5, 111.7, 108.5, 104.2; ESIMS-LR *m/z* 192.1 [$(M+H)^+$].

5-Nitrobenzofuran-2-carbohydrazide (**BZ39**)

Compound **BZ39** (197.8 mg, 0.89 mmol, 89% as a brown solid) was prepared from ethyl 5-nitrobenzofuran-2-carboxylate (235 mg, 1.0 mmol) and $NH_2NH_2·H_2O$ (122 μL) in EtOH (1.0 mL) as described above.
^1H NMR (DMSO-d_6, 400 MHz) δ 10.3 (s, 1H, NH), 8.77 (d, 1H, H-4, $J_{4,6}$ = 2.6 Hz), 8.31 (dd, 1H, H-6, $J_{6.4}$ = 2.6, $J_{6,7}$ = 8.8 Hz), 7.89 (d, 1H, H-7, $J_{7,6}$ = 8.8 Hz), 7.71 (s, 1H, H-3), 4.66 (s, 2H, NH_2); ^{13}C NMR (DMSO-d_6, 400 MHz) δ137.0, 156.9, 151.2, 144.1, 127.7, 122.0, 119.4, 112.8, 109.5; ESIMSLR *m/z* 222.1 [$(M+H)^+$].

General procedure for the synthesis of AC hydrazides

A solution of 3-methylbutanoyl chloride or butanoic anhydride (1 mmol) in THF (5 mL) was treated with $NH_2NH_2·H_2O$ (243 μL, 5 mmol) at 0 °C for 2.5 h. The reaction mixture was concentrated *in vacuo*. The residue was purified by Hi-Flash silica gel column chromatography to afford **AC58** and **AC59**.

A solution of geranic acid and lauric acid (1 mmol) in CH_2Cl_2 (5 mL) was treated with $(COCl)_2$ (85.8 μL, 1 mmol) at 0 °C for 30 min. The mixture was warmed to room temperature and stirred for 2 h. The reaction mixture was cooled at 0 °C and treated with $NH_2NH_2·H_2O$ (243 μL, 5 mmol) for 2 h. The resulting mixture was partitioned between Et_2O and H_2O. The organic layer was washed with brine, dried (Na_2SO_4), filtered and concentrated *in vacuo*. The residue was purified by Hi-Flash silica gel column chromatography to afford **AC60** and **AC61**.

Hydrazides **AC58**[62)] and **AC61**[63)] are known compounds. Compounds **AC55**, **AC56**, and **AC57** are commercially available.

3-Methylbutanohydrazide (**AC59**)
^1H NMR (DMSO-d_6, 400 MHz) δ 8.89 (s, 1H, N*H*), 4.16 (br s, 2H, NH_2), 1.95 (m, 1H, C*H*-β), 1.88 (d, 2H, CH_2-α, J = 6.4 Hz), 0.85 (d, 6H, CH_3-γ, J = 6.3 Hz); ESIMSLR *m/z* 117.1 [$(M+H)^+$].

(*E*)-3,7-Dimethyloctan-2,7-dienohydrazide (**AC60**)
^1H NMR (DMSO-d_6, 400 MHz) δ 8.94 (s, 1H, N*H*), 5.55 (s, 1H, C*H*-α), 5.07 (t, 1H, C=C*H*, J = 6.2 Hz), 4.20 (br s, 2H, NH_2), 2.09-2.00 (m, 7H, CH_2×2, CH_3), 1.64 (s, 3H, CH_3), 1.57 (s, 3H, CH_3); ESIMSLR *m/z* 205.1 [$(M+Na)^+$].

General procedure for the synthesis of AA and LA hydrazides

Each 2-chlorotrityl chloride (2-CTC) resin (300×2 mg, 0.51×2 mmol) was placed in two 10 mL polypropylene syringes fitted with a polyethylene filter disc. Each resin was agitated with CH_2Cl_2 (4 mL, 1h). After the removal of CH_2Cl_2, a solution of $NH_2NH_2·H_2O$, $^iPr_2NEt$, DMF, and MeOH (v/v 0.25/0.88/2.4/0.8, 4 mL) was added. After agitation for 1.5 h at room temperature, solvent and soluble reagents were removed by suction. All the resins were subjected to the following washing treatment with $^iPr_2NEt$/MeOH/DMF (1/2/17×3), DMF (×3), and CH_2Cl_2 (×3). A

solution of Fmoc-AA-OH (1.5×2 mmol), HBTU (1.45×2 mmol), and $^{i}Pr_2NEt$ (4.12×2 mmol) in DMF (1.8×2 mL) was added to the resins, which were agitated for 1.5 h. All the resins were washed with DMF (×3) and CH_2Cl_2 (×3).

Amino acids bound resins (100 mg) were placed in a 5 mL polypropylene syringe fitted with a polyethylene filter disc. Each resin was agitated with CH_2Cl_2 (4 mL, 30 min). The resins were treated with piperidine/DMF (1:4. 5 min, then 1:9, 15 min) to remove the Fmoc group, and the resins were washed with DMF (×3) and CH_2Cl_2 (×3). A solution of $^{i}Pr_2NEt$ (0.65 mL) in DMF (3 mL) was added to the resins, and acyl chloride (0.68 mmol) was added. In the case of carboxylic acid instead of acyl chloride, a solution of carboxylic acid (0.4 mmol), HBTU (0.4 mmol), and $^{i}Pr_2NEt$ (1 mmol) in DMF (2 mL) was added to the resins. After agitation for 2 h, all the resins were washed with DMF (×3) and CH_2Cl_2 (×3). The resins were treated with 50% TFA/CH_2Cl_2, and the supernatant was concentrated *in vacuo*. In the case of amino acids possessing Trt and Pbf groups, the crude compound was treated with a solution of TFA/$^{i}Pr_3SiH$/H_2O (95/2.5/2.5) for 15 min, and the resulting mixture was concentrated *in vacuo*. The residue was washed with Et_2O (or hexane) to afford **AA** and **LA** hydrazides as TFA salts.

N-Acetyl-L-alanine hydrazide (**AA62**)
^{1}H NMR (DMSO-d_6, 400 MHz) δ 10.55 (s, 1H, N*H*), 8.25 (d, 1H, Ala-α-N*H*, *J* = 6.8 Hz), 4.25 (quin, 1H, Ala-α-C*H*, *J* = 7.2 Hz), 1.84 (s, 3H, Ac), 1.22 (d, 3H, Ala-β-CH_3, *J* = 7.2 Hz); ESIMSLR *m/z* 168.0 [$(M+Na)^+$].

N-Acetyl-L-phenylalanine hydrazide (**AA63**)
^{1}H NMR (DMSO-d_6, 400 MHz) δ 10.63 (s, 1H, N*H*), 8.34 (d, 1H, Phe-α-N*H*, *J* = 8.0 Hz), 7.30-7.19 (m, 5H, Ph), 4.52-4.46 (m, 1H, Phe-α-C*H*), 2.95 (dd, 1H, Phe-β-CH_2, J_{gem} = 13.6, $J_{β,α}$ = 4.8 Hz), 2.80 (dd, 1H, Phe-β-CH_2, J_{gem} = 13.6, $J_{β,α}$ = 9.6 Hz), 1.77 (s, 3H, Ac); ESIMSLR *m/z* 243.9 [$(M+Na)^+$].

N-Acetyl-L-serine hydrazide (**AA64**)
^{1}H NMR (DMSO-d_6, 400 MHz) δ 10.75 (s, 1H, N*H*), 8.15 (d, 1H, Ser-α-N*H*, *J* = 7.6 Hz), 4.31 (dd, 1H, Ser-α-C*H*, *J* = 13.6, *J* = 6.0 Hz), 3.59 (d, 2H, Ser-β-CH_2, *J* = 6.0 Hz), 1.87 (s, 3H, Ac); ESIMSLR *m/z* 161.9, [$(M+H)^+$].

N-Acetyl-L-glutamic acid hydrazide (**AA65**)
^{1}H NMR (DMSO-d_6, 400 MHz) δ 10.74 (s, 1H, N*H*), 8.24 (d, 1H, Glu-α-N*H*, *J* = 8.0 Hz), 4.27-4.22 (m, 1H, Glu-α-C*H*), 2.26 (t, 2H, Glu-γ-CH_2, *J* = 7.6 Hz), 1.95-1.70 (m, 2H, Glu-β-CH_2), 1.85 (s, 3H, Ac); ESIMSLR *m/z* 225.3 [$(M+Na)^+$].

N-Acetyl-L-glutamine hydrazide (**AA66**)
^{1}H NMR (DMSO-d_6, 400 MHz) δ 10.84 (s, 1H, N*H*), 8.27 (d, 1H, Gln-α-N*H*, *J* = 7.6 Hz), 7.31 (s, 1H, Gln-δ-NH_2), 6.81 (s, 1H, Gln-δ-NH_2), 4.24-4.18 (m, 1H, Gln-α-C*H*), 2.20-2.05 (m, 2H, Gln-γ-CH_2), 1.95-1.81 (m, 1H, Glu-β-CH_2), 1.86 (s, 3H, Ac), 1.77-1.71 (m, 1H, Glu-β-CH_2); ESIMSLR *m/z* 224.9 [$(M+Na)^+$].

N-Acetyl-L-lysine hydrazide (**AA67**)
^{1}H NMR (DMSO-d_6, 400 MHz) δ 10.60 (s, 1H, N*H*), 8.21 (d, 1H, Lys-α-N*H*, *J* = 8.0 Hz), 7.67 (br s, 3H, Lys-ε-

NH_3), 4.25-4.19 (m, 1H, Lys-α-CH), 2.80-2.72 (m, 2H, Lys-ε-CH_2), 1.86 (s, 3H, Ac), 1.66-1.45 (m, 4H, Lys-β-CH_2, Lys-δ-CH_2), 1.39-1.28 (m, 2H, Lys-γ-CH_2); ESIMSLR *m/z* 203.0 [$(M+H)^+$].

N-Acetyl-L-arginine hydrazide (**AA68**)

^{1}H NMR (DMSO-d_6, 400 MHz) δ 10.28 (s, 1H, NH), 8.20 (d, 1H, Arg-α-NH, J = 8.4 Hz), 7.58 (br s, 1H, Arg-δ-NH), 7.48-6.69 (br s, 4H, Arg-δ-NH), 4.27-4.21 (m, 1H, Arg-α-CH), 3.12-3.07 (m, 2H, Arg-δ-CH_2), 1.85 (s, 3H, Ac), 1.69-1.60 (m, 1H, Arg-β-CH_2 or Arg-γ-CH_2), 1.57-1.37 (m, 3H, Arg-β-CH_2, Arg-γ-CH_2); ESIMSLR *m/z* 231.0 [$(M+H)^+$].

N-Octanoyl-L-alanine hydrazide (**LA69**)

^{1}H NMR (DMSO-d_6, 400 MHz) δ 10.73 (s, 1H, NH), 8.18 (d, 1H, Ala-α-NH, J = 7.2 Hz), 4.26 (quin, 1H, Ala-α-CH, J = 7.2 Hz), 2.10 (t, 2H, acyl-α-CH_2, J = 7.6 Hz), 1.49-1.43 (m, 2H, acyl-β-CH_2), 1.31-1.17 (m, 11H, Ala-β-CH_3, acyl-CH_2×4), 0.86 (t, 3H, acyl-CH_3, J = 6.8 Hz); ESIMSLR *m/z* 252.1 [$(M+Na)^+$].

N-(4-Phenylbenzoyl)-L-alanine hydrazide (**LA70**)

^{1}H NMR (DMSO-d_6, 400 MHz) δ 9.71 (s, 1H, NH), 8.61 (d, 1H, Ala-α-NH, J = 7.2 Hz), 8.00 (d, 2H, Ar, J = 8.4 Hz), 7.78 (d, 2H, Ar, J = 8.4 Hz), 7.74 (d, 2H, Ar, J = 8.4 Hz), 7.50 (t, 2H, Ar, J = 7.8 Hz), 7.41 (t, 1H, Ar, J = 7.2 Hz), 4.49 (quin, 1H, Ala-α-CH, J = 7.2 Hz), 1.36 (d, 3H, Ala-β-CH_3, $J_{β,α}$ = 7.2 Hz); ESIMSLR *m/z* 284.1 [$(M+H)^+$].

N-Isostearoyl-L-alanine hydrazide (**LA71**)

^{1}H NMR (DMSO-d_6, 400 MHz) δ 10.55 (s, 1H, NH), 8.14 (d, 1H, Ala-α-NH, J = 6.8 Hz), 4.26 (quin, 1H, Ala-α-CH, J = 6.8 Hz), 2.21-2.16 (m, 1H, acyl-α-CH), 1.46-1.33 (m, 2H, acyl-β-CH_2), 1.31-1.09 (m, 29H, Ala-β-CH_3, acyl-CH_2×13), 0.85 (t, 6H, acyl-CH_3×2, J = 7.0 Hz); ESIMSLR *m/z* 370.0 [$(M+H)^+$].

N-Palmitoyl-L-alanine hydrazide (**LA72**)

^{1}H NMR (DMSO-d_6, 400 MHz) δ 10.48 (s, 1H, NH), 8.14 (d, 1H, Ala-α-NH, J = 6.8 Hz), 4.25 (quin, 1H, Ala-α-CH, J = 7.2 Hz), 2.09 (t, 2H, acyl-α-CH_2, J = 7.6 Hz), 1.48-1.42 (m, 2H, acyl-β-CH_2), 1.30-1.16 (m, 27H, Ala-β-CH_3, acyl-CH_2×12), 0.85 (t, 3H, acyl-CH_3, J = 7.0 Hz); ESIMSLR *m/z* 342.2 [$(M+H)^+$].

N-Octanoyl-L-phenylalanine hydrazide (**LA73**)

^{1}H NMR (DMSO-d_6, 400 MHz) δ 10.73 (s, 1H, NH), 8.18 (d, 1H, Phe-α-NH, J = 8.4 Hz), 7.28-7.18 (m, 5H, Ph), 4.52-4.46 (m, 1H, Phe-α-CH), 2.94 (dd, 1H, Phe-β-CH_2, J_{gem} = 13.2, $J_{β,α}$ = 4.9 Hz), 2.77 (dd, 1H, Phe-β-CH_2, J_{gem} = 13.2, $J_{β,α}$ = 10.1 Hz), 2.01 (t, 2H, acyl-α-CH_2, J = 7.6 Hz), 1.37-1.01 (m, 12H, acyl-CH_2×6), 0.85 (t, 3H, acyl-CH_3, J = 7.4 Hz); ESIMSLR *m/z* 306.1 [$(M+H)^+$].

N-(4-Phenylbenzoyl)-L-phenylalanine hydrazide (**LA74**)

^{1}H NMR (DMSO-d_6, 400 MHz) δ 10.31 (s, 1H, NH), 8.80 (d, 1H, Phe-α-NH, J = 8.4 Hz), 7.91 (d, 2H, Ar, J = 8.8 Hz), 7.76 (d, 2H, Ar, J = 8.8 Hz), 7.72 (d, 2H, Ar, J = 8.4 Hz), 7.49 (t, 2H, Ar, J = 7.6 Hz), 7.43-7.35 (m, 3H, Ar),

7.28 (t, 2H, Ar, *J* = 7.6 Hz), 7.20-7.16 (m, 1H, Ar), 4.73 (q, 1H, Phe-α-C*H*, *J* = 8.0 Hz), 3.08 (d, 2H, Phe-β-CH_2, *J* = 7.6 Hz); ESIMSLR *m/z* 360.1 [$(M+H)^+$].

N-Isostearoyl-L-phenylalanine hydrazide (**LA75**)

^{1}H NMR (DMSO-d_6, 400 MHz) δ 10.55 (s, 1H, N*H*), 8.18 (d, 1H, Phe-α-N*H*, *J* = 8.8 Hz), 7.28-7.14 (m, 5H, Ph), 4.58-4.52 (m, 1H, Phe-α-C*H*), 2.93 (dd, 1H, Phe-β-CH_2, J_{gem} = 13.5, $J_{β,α}$ = 4.5 Hz), 2.81 (dd, 1H, Phe-β-CH_2, J_{gem} = 13.5, $J_{β,α}$ = 10.8 Hz), 2.13-2.07 (m, 1H, acyl-α-C*H*), 1.37-1.00 (m, 28H, acyl-CH_2×14), 0.89-0.84 (m, 6H, acyl-CH_3×2); ESIMSLR *m/z* 446.1 [$(M+H)^+$].

N-Palmitoyl-L-phenylalanine hydrazide (**LA76**)

^{1}H NMR (DMSO-d_6, 400 MHz) δ 10.49 (s, 1H, N*H*), 8.21 (d, 1H, Phe-α-N*H*, *J* = 8.0 Hz), 7.28-7.14 (m, 5H, Ph), 4.54-4.48 (m, 1H, Phe-α-C*H*), 2.95 (dd, 1H, Phe-β-CH_2, J_{gem} = 13.5, $J_{β,α}$ = 4.9 Hz), 2.79 (dd, 1H, Phe-β-CH_2, J_{gem} = 13.5, $J_{β,α}$ = 10.1 Hz), 2.02 (t, 2H, acyl-α-CH_2, *J* = 7.4 Hz), 1.37-1.01 (m, 26H, acyl-CH_2×13), 0.85 (t, 3H, acyl-CH_3, *J* = 7.0 Hz); ESIMSLR *m/z* 418.2 [$(M+H)^+$].

N-Octanoyl-L-serine hydrazide (**LA77**)

^{1}H NMR (DMSO-d_6, 400 MHz) δ 10.99 (s, 1H, N*H*), 8.07 (d, 1H, Ser-α-N*H*, *J* = 8.0 Hz), 4.32 (q, 1H, Ser-α-C*H*, *J* = 6.5 Hz), 3.60 (d, 2H, Ser-β-CH_2, *J* = 6.0 Hz), 2.14 (t, 2H, acyl-α-CH_2, *J* = 7.2 Hz), 1.50-1.42 (m, 2H, acyl-β-CH_2), 1.29-1.20 (m, 8H, acyl-CH_2×4), 0.85 (t, 3H, acyl-CH_3, *J* = 7.0 Hz); ESIMSLR *m/z* 268.0 [$(M+Na)^+$].

N-(4-Phenylbenzoyl)-L-serine hydrazide (**LA78**)

^{1}H NMR (DMSO-d_6, 400 MHz) δ 9.90 (s, 1H, N*H*), 8.42 (d, 1H, Ser-α-N*H*, *J* = 7.2 Hz), 8.01 (d, 2H, Ar, *J* = 8.0 Hz), 7.80 (d, 2H, Ar, *J* = 6.8 Hz), 7.75 (d, 2H, Ar, *J* = 8.0 Hz), 7.50 (t, 2H, Ar, *J* = 7.2 Hz), 7.42 (t, 1H, Ar, *J* = 8.4 Hz), 4.53 (q, 1H, Ser-α-C*H*, *J* = 6.0 Hz), 3.74 (d, 2H, Ser-β-CH_2, *J* = 5.2 Hz); ESIMSLR *m/z* 300.1 [$(M+H)^+$].

N-Isostearoyl-L-serine hydrazide (**LA79**)

^{1}H NMR (DMSO-d_6, 400 MHz) δ 10.54 (s, 1H, N*H*), 8.00 (d, 1H, Ser-α-N*H*, *J* = 7.6 Hz), 4.31 (q, 1H, Ser-α-C*H*, *J* = 6.4 Hz), 3.53 (obscured, 2H, Ser-β-CH_2), 2.25-2.18 (m, 1H, acyl-α-C*H*), 1.47-1.10 (m, 28H, acyl-CH_2×14), 0.85 (m, 6H, acyl-CH_3×2); ESIMSLR *m/z* 386.0 [$(M+H)^+$].

N-Palmitoyl-L-serine hydrazide (**LA80**)

^{1}H NMR (DMSO-d_6, 400 MHz) δ 10.38 (s, 1H, N*H*), 7.98 (d, 1H, Ser-α-N*H*, *J* = 8.0 Hz), 4.30 (q, 1H, Ser-α-C*H*, *J* = 6.4 Hz), 3.56 (d, 2H, Ser-β-CH_2, *J* = 5.6 Hz), 2.13 (t, 2H, acyl-α-CH_2, *J* = 7.2 Hz), 1.50-1.43 (m, 2H, acyl-β-CH_2), 1.31-1.17 (m, 24H, acyl-CH_2×12), 0.85 (t, 3H, acyl-CH_3, *J* = 6.6 Hz); ESIMSLR *m/z* 358.2 [$(M+H)^+$].

N-Octanoyl-L-glutamic acid hydrazide (**LA81**)

^{1}H NMR (DMSO-d_6, 400 MHz) δ 9.56 (br s, 1H, N*H*), 8.98 (d, 1H, Glu-α-N*H*, *J* = 8.0 Hz), 4.22 (q, 1H, Glu-α-C*H*, *J* = 7.3 Hz), 2.23-2.18 (m, 2H, Glu-γ-CH_2), 2.15-2.09 (m, 2H, acyl-α-CH_2), 1.92-1.68 (m, 2H, Glu-β-CH_2), 1.51-

1.43 (m, 2H, acyl-β-C*H*$_2$), 1.30-1.19 (m, 8H, acyl-C*H*$_2$×4), 0.85 (t, 3H, acyl-C*H*$_3$, *J* = 7.0 Hz); ESIMSLR *m/z* 310.0 [(M+Na)$^+$], 285.9 [(M-H)$^-$].

N-(4-Phenylbenzoyl)-L-glutamic acid hydrazide (**LA82**)

^{1}H NMR (DMSO-*d*$_6$, 400 MHz) δ 9.73 (s, 1H, N*H*), 8.57 (d, 1H, Glu-α-N*H*, *J* = 8.0 Hz), 8.00 (d, 2H, Ar, *J* = 8.8 Hz), 7.78 (d, 2H, Ar, *J* = 8.8 Hz), 7.74 (d, 2H, Ar, *J* = 8.8 Hz), 7.50 (t, 2H, Ar, *J* = 7.8 Hz), 7.41 (t, 1H, Ar, *J* = 7.6 Hz), 4.45 (m, 1H, Glu-α-C*H*), 2.40-2.27 (m, 2H, Glu-γ-C*H*$_2$), 2.08-1.91 (m, 2H, Glu-β-C*H*$_2$); ESIMSLR *m/z* 342.1 [(M+H)$^+$].

N-Isostearoyl-L-glutamic acid hydrazide (**LA83**)

^{1}H NMR (DMSO-*d*$_6$, 400 MHz) δ 10.61 (s, 1H, N*H*), 8.15 (d, 1H, Glu-α-N*H*, *J* = 7.6 Hz), 4.26 (m, 1H, Glu-α-C*H*), 2.31-2.20 (m, 3H, Glu-γ-C*H*$_2$, acyl-α-C*H*), 1.90-1.75 (m, 2H, Glu-β-C*H*$_2$), 1.46-1.12 (m, 28H, acyl-C*H*$_2$×14), 0.85 (t, 6H, acyl-C*H*$_3$×2, *J* = 6.4 Hz); ESIMSLR *m/z* 428.0 [(M+H)$^+$].

N-Palmitoyl-L-glutamic acid hydrazide (**LA84**)

^{1}H NMR (DMSO-*d*$_6$, 400 MHz) δ 10.16 (s, 1H, N*H*), 8.06 (d, 1H, Glu-α-N*H*, *J* = 8.0 Hz), 4.23 (m, 1H, Glu-α-C*H*), 2.26-2.20 (m, 2H, Glu-γ-C*H*$_2$), 2.17-2.06 (m, 2H, acyl-α-C*H*$_2$), 1.91-1.70 (m, 2H, Glu-β-C*H*$_2$), 1.50-1.43 (m, 2H, acyl-β-C*H*$_2$), 1.30-1.18 (m, 24H, acyl-C*H*$_2$×12), 0.85 (t, 3H, acyl-C*H*$_3$, *J* = 7.0 Hz); ESIMSLR *m/z* 400.1 [(M+H)$^+$], 398.1 [(M−H)$^−$].

N-Octanoyl-L-glutamine hydrazide (**LA85**)

^{1}H NMR (DMSO-*d*$_6$, 400 MHz) δ 10.06 (s, 1H, N*H*), 8.06 (d, 1H, Gln-α-N*H*, *J* = 6.8 Hz), 7.28 (s, 1H, Gln-δ-N*H*$_2$), 6.78 (s, 1H, Gln-δ-N*H*$_2$), 4.19 (m, 1H, Gln-α-C*H*), 2.15-2.05 (m, 4H, Gln-γ-C*H*$_2$, acyl-α-C*H*$_2$), 1.90-1.68 (m, 2H, Gln-β-C*H*$_2$), 1.51-1.44 (m, 2H, acyl-β-C*H*$_2$), 1.30-1.19 (m, 8H, acyl-C*H*$_2$×4), 0.86 (t, 3H, acyl-C*H*$_3$, *J* = 6.8 Hz); ESIMSLR *m/z* 287.0 [(M+H)$^+$].

N-(4-Phenylbenzoyl)-L-glutamine hydrazide (**LA86**)

^{1}H NMR (DMSO-*d*$_6$, 400 MHz) δ 10.80 (s, 1H, N*H*), 8.90 (d, 1H, Gln-α-N*H*, *J* = 7.2 Hz), 8.00 (d, 2H, Ar, *J* = 8.8 Hz), 7.80 (d, 2H, Ar, *J* = 8.8 Hz), 7.74 (d, 2H, Ar, *J* = 7.2 Hz), 7.51 (t, 2H, Ar, *J* = 7.6 Hz), 7.42 (t, 1H, Ar, *J* = 7.6 Hz), 7.39 (s, 1H, Gln-δ-N*H*$_2$), 6.90 (s, 1H, Gln-δ-N*H*$_2$), 4.43 (m, 1H, Gln-α-C*H*), 2.32-2.19 (m, 2H, Gln-γ-C*H*$_2$), 2.10-1.93 (m, 2H, Gln-β-C*H*$_2$); ESIMSLR *m/z* 363.1 [(M+Na)$^+$].

N-Isostearoyl-L-glutamine hydrazide (**LA87**)

^{1}H NMR (DMSO-*d*$_6$, 400 MHz) δ 8.10 (br s, 1H, Gln-α-N*H*), 7.27 (br s, 1H, Gln-δ-N*H*$_2$), 6.77 (br s, 1H, Gln-δ-N*H*$_2$), 4.28-4.20 (m, 1H, Gln-α-C*H*), 2.25-2.06 (m, 3H, Gln-γ-C*H*$_2$, acyl-α-C*H*), 1.90-1.72 (m, 2H, Gln-β-C*H*$_2$), 1.47-1.13 (m, 28H, acyl-C*H*$_2$×14), 0.85 (br s, 6H, acyl-C*H*$_3$×2); ESIMSLR *m/z* 427.3 [(M+H)$^+$].

N-Palmitoyl-L-glutamine hydrazide (**LA88**)

^{1}H NMR (DMSO-d_6, 400 MHz) δ 10.38 (s, 1H, N*H*), 8.11 (d, 1H, Gln-α-N*H*, *J* = 7.2 Hz), 7.29 (s, 1H, Gln-δ-NH_2), 6.79 (s, 1H, Gln-δ-NH_2), 4.20 (m, 1H, Gln-α-C*H*), 2.17-2.06 (m, 4H, Gln-γ-CH_2, acyl-α-CH_2), 1.90-1.69 (m, 2H, Gln-β-CH_2), 1.51-1.44 (m, 2H, acyl-β-CH_2), 1.30-1.19 (m, 24H, acyl-CH_2×12), 0.85 (t, 3H, acyl-CH_3, *J* = 7.0 Hz); ESIMSLR *m/z* 399.3 [(M+H)$^+$].

N-Octanoyl-L-lysine hydrazide (**LA89**)

^{1}H NMR (DMSO-d_6, 400 MHz) δ 9.82 (s, 1H, N*H*), 8.00 (d, 1H, Lys-α-N*H*, *J* = 7.2 Hz), 7.65 (br s, 3H, Lys-ε-NH_3), 4.21 (m, 1H, Lys-α-C*H*), 2.79-2.70 (m, 2H, Lys-ε-CH_2), 2.11 (t, 2H, acyl-α-CH_2, *J* = 7.2 Hz), 1.62-1.42 (m, 6H, Lys-β-CH_2, Lys-δ-CH_2, acyl-β-CH_2), 1.38-1.19 (m, 10H, Lys-γ-CH_2, acyl-CH_2×4), 0.86 (t, 3H, acyl-CH_3, *J* = 7.0 Hz); ESIMSLR *m/z* 287.0 [(M+H)$^+$].

N-(4-Phenylbenzoyl)-L-lysine hydrazide (**LA90**)

^{1}H NMR (DMSO-d_6, 400 MHz) δ 8.71 (d, 1H, Lys-α-N*H*, *J* = 7.4 Hz), 8.01 (d, 2H, Ar, *J* = 8.8 Hz), 7.80 (d, 2H, Ar, *J* = 8.0 Hz), 7.74 (d, 2H, Ar, *J* = 7.2 Hz), 7.65 (br s, 3H, Lys-ε-NH_3), 7.51 (t, 2H, Ar, *J* = 7.8 Hz), 7.42 (t, 1H, Ar, *J* = 7.4 Hz), 4.48 (m, 1H, Lys-α-C*H*), 2.83-2.73 (m, 2H, Lys-ε-CH_2), 1.88-1.31 (m, 6H, Lys-β-CH_2, Lys-γ-CH_2, Lys-δ-CH_2); ESIMSLR *m/z* 341.2 [(M+H)$^+$].

N-Isostearoyl-L-lysine hydrazide (**LA91**)

^{1}H NMR (DMSO-d_6, 400 MHz) δ 10.49 (s, 1H, N*H*), 8.11 (d, 1H, Lys-α-N*H*, *J* = 7.2 Hz), 7.70 (s, 3H, Lys-ε-NH_3), 4.24 (q, 1H, Lys-α-C*H*, *J* = 7.5 Hz), 2.78-2.69 (m, 2H, Lys-ε-CH_2), 2.23-2.18 (m, 1H, acyl-α-C*H*), 1.60-1.10 (m, 34H, Lys-β-CH_2, Lys-γ-CH_2, Lys-δ-CH_2, acyl-CH_2×14), 0.86 (t, 6H, acyl-CH_3×2, *J* = 6.4 Hz); ESIMSLR *m/z* 427.1 [(M+H)$^+$].

N-Palmitoyl-L-lysine hydrazide (**LA92**)

^{1}H NMR (DMSO-d_6, 400 MHz) δ 7.99 (d, 1H, Lys-α-N*H*, *J* = 8.4 Hz), 7.64 (s, 3H, Lys-ε-NH_3), 4.20 (m, 1H, Lys-α-C*H*), 2.78-2.70 (m, 2H, Lys-ε-CH_2), 2.10 (t, 2H, acyl-α-CH_2, *J* = 7.6 Hz), 1.64-1.41 (m, 6H, Lys-β-CH_2, Lys-δ-CH_2, acyl-β-CH_2), 1.34-1.15 (m, 26H, Lys-γ-CH_2, acyl-CH_2×12), 0.85 (t, 3H, acyl-CH_3, *J* = 6.8 Hz); ESIMSLR *m/z* 399.2 [(M+H)$^+$].

N-Octanoyl-L-arginine hydrazide (**LA93**)

^{1}H NMR (DMSO-d_6, 400 MHz) δ 8.04 (d, 1H, Arg-α-N*H*, *J* = 7.6 Hz), 7.48 (br s, 1H, Arg-δ-N*H*), 7.47-6.58 (br s, 4H, Arg-δ-N*H*), 4.23 (q, 1H, Arg-α-C*H*, *J* = 7.2 Hz), 3.08 (q, 2H, Arg-δ-CH_2, *J* = 6.2 Hz), 2.11 (t, 2H, acyl-α-CH_2, *J* = 7.4 Hz), 1.67-1.34 (m, 6H, Arg-β-CH_2, Arg-γ-CH_2, acyl-β-CH_2), 1.30-1.14 (m, 8H, acyl-CH_2×4), 0.86 (t, 3H, acyl-CH_3, *J* = 7.2 Hz); ESIMSLR *m/z* 315.1 [(M+H)$^+$].

N-Isostearoyl-L-arginine hydrazide (**LA94**)

^{1}H NMR (DMSO-d_6, 400 MHz) δ 10.37 (s, 1H, N*H*), 8.08 (d, 1H, Arg-α-N*H*, *J* = 7.6 Hz), 7.50 (br s, 1H, Arg-δ-N*H*), 7.39-6.62 (br s, 4H, Arg-δ-N*H*), 4.24 (m, 1H, Lys-α-C*H*), 3.13-3.08 (m, 2H, Arg-δ-CH_2), 2.27-2.19 (m, 1H, acyl-α-

C*H*), 1.68-1.10 (m, 32H, Arg-β-CH_2, Arg-γ-CH_2,, acyl-CH_2×14), 0.87-0.83 (m, 6H, acyl-CH_3×2); ESIMSLR *m/z* 455.3 [$(M+H)^+$].

N-(4-Phenylbenzoyl)-L-arginine hydrazide (**LA95**)

^{1}H NMR (DMSO-d_6, 400 MHz) δ 9.39 (s, 1H, N*H*), 8.54 (d, 1H, Arg-α-N*H*, *J* = 8.0 Hz), 8.00 (d, 2H, Ar, *J* = 8.0 Hz), 7.80-7.72 (m, 4H, Ar), 7.52-7.47 (m, 3H, Arg-δ-N*H*, Ar), 7.42 (t, 1H, Ar, *J* = 7.2 Hz), 7.42-6.67 (br s, 4H, Arg-δ-N*H*), 4.46 (m, 1H, Arg-α-C*H*), 3.16-3.11 (m, 2H, Arg-δ-CH_2), 1.91-1.42 (m, 4H, Arg-β-CH_2, Arg-γ-CH_2); ESIMSLR *m/z* 369.1 [$(M+H)^+$].

N-Palmitoyl-L-arginine hydrazide (**LA96**)

^{1}H NMR (DMSO-d_6, 400 MHz) δ 8.01 (d, 1H, Arg-α-N*H*, *J* = 8.4 Hz), 7.46 (br s, 1H, Arg-δ-N*H*), 7.47-6.64 (br s, 4H, Arg-δ-N*H*), 4.23 (q, 1H, Arg-α-C*H*, *J* = 7.2 Hz), 3.10-3.06 (m, 2H, Arg-δ-CH_2), 2.11 (t, 2H, acyl-α-CH_2, *J* = 7.4 Hz), 1.66-1.12 (m, 30H, Arg-β-CH_2, Arg-γ-CH_2, acyl-CH_2×13), 0.85 (t, 3H, acyl-CH_3, *J* = 6.6 Hz); ESIMSLR *m/z* 427.3 [$(M+H)^+$].

N-Palmitoyl-L-glutamic acid-L-glutamic acid hydrazide (**LA97**)

^{1}H NMR (DMSO-d_6, 400 MHz) δ 10.47 (s, 1H, N*H*), 8.18 (d, 1H, Glu-α-N*H*, *J* = 7.2 Hz), 7.96 (d, 1H, Glu-α-N*H*, *J* = 8.0 Hz), 4.28-4.21 (m, 2H, Glu-α-C*H*×2), 2.30-2.04 (m, 6H, Glu-γ-CH_2×2, acyl-α-CH_2), 1.93-1.64 (m, 4H, Glu-β-CH_2×2), 1.50-1.41 (m, 2H, acyl-β-CH_2), 1.30-1.16 (m, 24H, acyl-CH_2×12), 0.85 (t, 3H, acyl-CH_3, *J* = 7.0 Hz); ESIMSLR *m/z* 527.1 [$(M+H)^+$], 527.1 [$(M-H)^-$].

N-Palmitoyl-L-lysine-L-lysine hydrazide (**LA98**)

^{1}H NMR (DMSO-d_6, 400 MHz) δ 8.11 (d, 1H, Lys-α-N*H*, *J* = 8.4 Hz), 7.94 (d, 1H, Lys-α-N*H*, *J* = 8.4 Hz), 7.72 (br s, 6H, Lys-ε-NH_3×2), 4.25-4.19 (m, 2H, Lys-α-C*H*×2), 2.78-2.70 (m, 4H, Lys-ε-CH_2×2), 2.16-2.02 (m, 2H, acyl-α-CH_2), 1.66-1.42 (m, 10H, Lys-β-CH_2×2, Lys-δ-CH_2×2, acyl-β-CH_2), 1.40-1.18 (m, 28H, Lys-γ-CH_2×2, acyl-CH_2×12) 0.85 (t, 3H, acyl-CH_3, *J* = 7.0 Hz); ESIMSLR *m/z* 527.2 [$(M+H)^+$].

Synthesis of palmitoyl amino acid fragments

Each 2-chlorotrityl chloride (2-CTC) resin (1.6 mmol/g, 1 equiv.) was placed in a 5 mL polypropylene syringe fitted with a polyethylene filter disc. Each resin was agitated with CH_2Cl_2 for 30 min. After the removal of CH_2Cl_2, a solution of Fmoc-AA-OH (3 equiv.) and $^iPr_2NEt$ (8 equiv.) in CH_2Cl_2 (1 mL) was added. After agitation for 2 h at room temperature, solvent and soluble reagents were removed by suction. All the resins were subjected to the following washing treatment with $^iPr_2NEt$/MeOH/DMF (1/2/17 ×3), DMF (×3), and CH_2Cl_2 (×3). The resins were treated with piperidine/DMF (1:4. 5 min, then 1:9, 15 min) to remove the Fmoc group, and the resins were washed with DMF (×3) and CH_2Cl_2 (×3). A solution of $^iPr_2NEt$ (8 equiv.) in DMF (0.7 mL) was added to the resins, and palmitoyl chloride (4 equiv.) was added. After agitation for 1.5 h, all the resins were washed with DMF (×3) and CH_2Cl_2 (×3). The resins were treated with 1% TFA/CH_2Cl_2, and the supernatant was concentrated *in vacuo* (toluene co-evaporation ×3). The resulting solid was used for the next step without further purification.

N_{α}-Pamitoyl N_{ε}-Boc lysine

2-CTC resin (150 mg) was used to afford the title compound (80.0 mg) as a white solid.

^{1}H NMR (DMSO-d_6, 400 MHz) δ 7.97 (d, 1H, Lys-α-N*H*, J = 8.2 Hz), 6.75 (br s, 1H, Lys-ε-N*H*), 4.15-4.09 (m, 1H, Lys-α-C*H*), 2.90-2.85 (m, 2H, Lys-ε-CH_2), 2.13-2.05 (m, 2H, acyl-α-CH_2), 1.70-1.62 (m, 1H, Lys-β-CH_2), 1.59-1.43 (m, 3H, Lys-β-CH_2, acyl-β-CH_2), 1.40-1.16 (m, 37H, Lys-γ-CH_2, Lys-δ-CH_2, ^{t}Bu, acyl-CH_2×12), 0.85 (t, 3H, acyl-CH_3, J = 6.8 Hz); ESIMSLR *m/z* 507.6 [(M+Na)$^{+}$].

N_{α}-Palmitoyl N_{ω}-Pbf arginine

2-CTC resin (150 mg) was used to afford the title compound (140 mg) as a yellow solid.

^{1}H NMR (DMSO-d_6, 400 MHz) δ 8.01 (d, 1H, Arg-α-N*H*, J = 7.8 Hz), 4.15-4.10 (m, 1H, Arg-α-C*H*), 3.02 (q, 2H, Arg-δ-CH_2, J = 6.3 Hz), 2.96 (s, 2H, Pbf-CH_2), 2.47 (s, 3H, Pbf-CH_3), 2.42 (s, 3H, Pbf-CH_3), 2.09 (t, 2H, acyl-α-CH_2, J = 7.3 Hz), 2.00 (s, 3H, Pbf-CH_3), 1.72-1.62 (m, 1H, Arg-β-CH_2), 1.57-1.33 (m, 11H, Arg-β-CH_2, Arg-γ-CH_2, acyl-β-CH_2, Pbf-CH_3×2), 1.30-1.18 (m, 24H, acyl-CH_2×12), 0.85 (t, 3H, acyl-CH_3, J = 6.9 Hz); ESIMSLR *m/z* 665.7 [(M+H)$^{+}$], 663.6 [(M−H)$^{-}$].

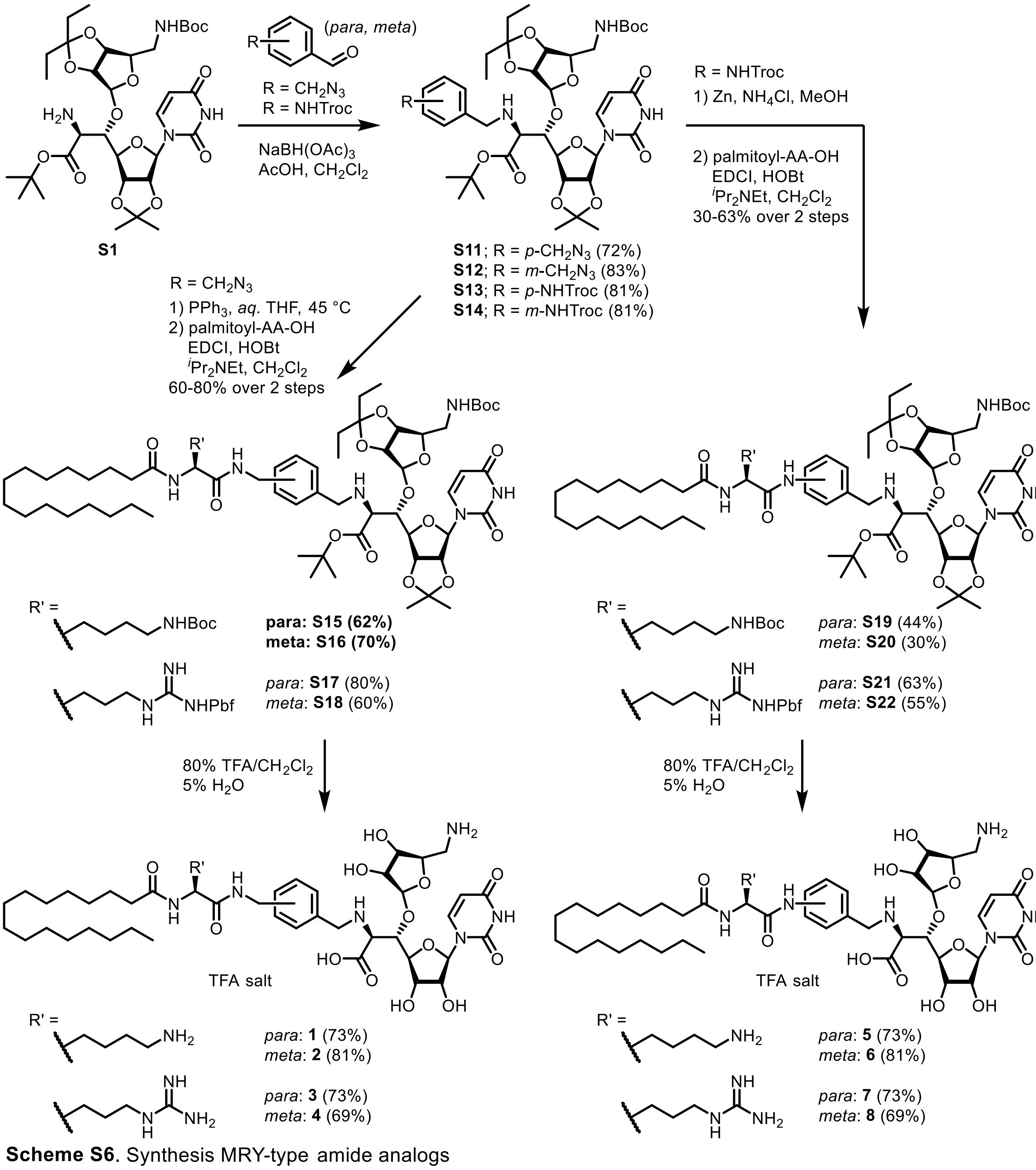

Scheme S6. Synthesis MRY-type amide analogs

tert-Butyl 5-*O*-[5-(*tert*-butyloxycarbonylamino)-5-deoxy-2,3-*O*-(3-pentylidene)-β-D-*ribo*-pentofuranosyl]-6-[4-(azidomethyl)benzylamino]-6-deoxy-2,3-*O*-isopropylidene-1-(uracil-1-yl)-β-D-*glycelo*-L-*taro*-heptofuranuronate (**S11**)

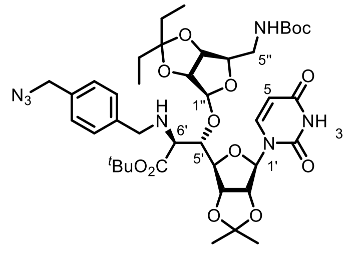

According to general procedure A, **S1** (50.0 mg, 70.1 μmol) and 4-azidomethylbenzaldehyde (16.9 mg, 105 μmol, 1.5 equiv.) were used to afford **S11** (43.1 mg, 72%) as a white solid after Hi-Flash silica gel column chromatography (S size, hexane/EtOAc = 3/2 → 2/3 gradient elution).

^{1}H NMR ($CDCl_3$, 400 MHz) δ 8.19 (br s, 1H, N*H*-3), 7.37 (d, 2H, Ar, *J* = 8.2 Hz), 7.23 (d, 2H, Ar, *J* = 8.2 Hz), 7.22 (d, 1H, H-6, $J_{6,5}$ = 8.2 Hz), 5.93 (br t, 1H, BocN*H*, *J* = 8.1 Hz), 5.67 (dd, 1H, H-5, $J_{5,6}$ = 8.1, $J_{5,3}$ = 1.8 Hz), 5.51 (d,

1H, H-1', $J_{1',2'}$ = 1.8 Hz), 5.09 (dd, 1H, H-2', $J_{2',3'}$ = 6.3, $J_{2',1'}$ = 1.8 Hz), 5.01 (s, 1H, H-1"), 4.75 (dd, 1H, H-3', $J_{3',2'}$ = 6.3, $J_{3',4'}$ = 4.5 Hz), 4.64 (dd, 1H, H-4', $J_{4',5'}$ = 9.1, $J_{4',3'}$ = 4.5 Hz), 4.56-4.51 (m, 2H, H-2", H-3"), 4.31 (s, 2H, ArC*H*₂), 4.26-4.23 (m, 1H, H-4"), 4.09 (br d, 1H, H-5', *J* = 8.6 Hz), 3.94 (d, 1H, ArC*H*₂, J_{gem} = 13.4 Hz), 3.60 (d, 1H, ArC*H*₂, J_{gem} = 13.4 Hz), 3.30 (d, 1H, H-6', $J_{6',5'}$ = 1.8 Hz), 3.24-3.16 (m, 1H, H-5"), 3.14-3.06 (m, 1H, H-5"), 1.58 (s, 3H, isopropylidene-CH_3), 1.52 (s, 9H, ^{t}Bu), 1.39 (s, 9H, ^{t}Bu), 1.34 (s, 3H, isopropylidene-CH_3), 1.61-1.35 (m, 4H, pentylidene-CH_2×2), 0.77 (t, 6H, pentylidene-CH_3×2, *J* = 7.5 Hz); ^{13}C NMR ($CDCl_3$, 100 MHz) δ 172.2, 163.3, 156.3, 150.0, 143.6, 140.4, 134.1, 129.0, 128.3, 116.1, 114.7, 112.9, 102.4, 96.1, 88.0, 87.1, 86.8, 84.8, 82.6, 82.2, 82.1, 81.9, 79.0, 61.5, 54.7, 51.9, 43.3, 29.5, 28.9, 28.5, 28.4, 27.4, 25.6, 8.6, 7.4; ESIMS-HR *m*/*z*: $[M+H]^+$ calcd. for $C_{41}H_{60}N_7O_{13}$ 858.4244, found 858.4262; $[\alpha]^{23}_D$ +8.30 (*c* 0.43, $CHCl_3$).

tert-Butyl 5-*O*-[5-(*tert*-butyloxycarbonylamino)-5-deoxy-2,3-*O*-(3-pentylidene)-β-D-*ribo*-pentofuranosyl]-6-[3-(azidomethyl)benzylamino]-6-deoxy-2,3-*O*-isopropylidene-1-(uracil-1-yl)-β-D-*glycelo*-L-*taro*-heptofuranuronate (**S12**)

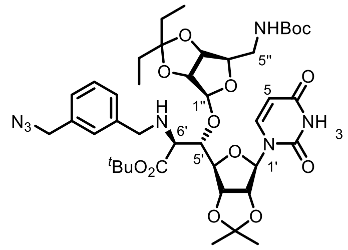

According to general procedure A, **S1** (50.0 mg, 70.1 μmol) and 3-azidomethylbenzaldehyde (16.9 mg, 105 μmol, 1.5 equiv.) were used to afford **S12** (50.0 mg, 83%) as a white solid after silica gel column chromatography (ϕ1.1×7 cm, MeOH/$CHCl_3$ = 0-1%).

^{1}H NMR ($CDCl_3$, 400 MHz) δ 8.32 (br s, 1H, N*H*-3), 7.39-7.18 (m, 5H, Ar, H-6), 5.91 (br s, 1H, BocN*H*), 5.66 (d, 1H, H-5, $J_{5,6}$ = 7.7 Hz), 5.52 (br s, 1H, H-1'), 5.09 (br d, 1H, H-2', *J* = 6.3 Hz), 5.02 (br s, 1H, H-1"), 4.77 (t, 1H, H-3', *J* = 5.4 Hz), 4.62 (dd, 1H, H-4', $J_{4',5'}$ = 9.1, $J_{4',3'}$ = 4.5 Hz), 4.57-4.51 (m, 2H, H-2", H-3"), 4.32 (s, 2H, ArC*H*₂), 4.26-4.22 (m, 1H, H-4"), 4.09 (br d, 1H, H-5', *J* = 8.6 Hz), 3.94 (d, 1H, ArC*H*₂, J_{gem} = 13.1 Hz), 3.61 (d, 1H, ArC*H*₂, J_{gem} = 13.1 Hz), 3.31 (s, 1H, H-6'), 3.25-3.17 (m, 1H, H-5"), 3.13-3.04 (m, 1H, H-5"), 1.68-1.32 (m, 28H, ^{t}Bu×2, pentylidene-CH_2×2, isopropylidene-CH_3×2), 0.78 (t, 6H, pentylidene-CH_3×2, *J* = 7.3 Hz); ^{13}C NMR ($CDCl_3$, 100 MHz) δ 172.2, 163.7, 156.3, 150.1, 143.5, 140.8, 135.4, 128.8, 128.5, 128.3, 126.9, 116.1, 114.7, 112.8, 102.4, 95.8, 87.9, 87.0, 86.7, 84.7, 82.6, 82.2, 82.0, 81.9, 79.0, 61.6, 54.8, 52.1, 43.3, 29.5, 28.9, 28.5, 28.3, 27.3, 25.6, 8.5, 7.4; ESIMS-HR *m*/*z*: $[M+H]^+$ calcd. for $C_{41}H_{60}N_7O_{13}$ 858.4244, found 858.4262; $[\alpha]^{22}_D$ +6.88 (*c* 0.58, $CHCl_3$).

2,2,2-trichloroethyl *N*-(4-formylphenyl)carbamate

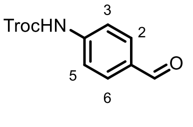

A solution of 4-aminobenzyl alcohol (246 mg, 2.00 mmol) and pyridine (323 μL, 4.00 mmol, 2.0 equiv.) in CH_2Cl_2 was treated with TrocCl (241 μL, 1.80 mmol, 0.9 equiv.) at 0 °C for 2 h. Sodium hydroxide (0.5 M in H_2O, 2 mL) was added to the reaction mixture and the mixture was stirred for 6 h. The resulting mixture was partitioned between EtOAc and 1 M *aq.* HCl, and the organic layer was washed with brine, dried (Na_2SO_4), filtered and concentrated *in vacuo*. A solution of the crude in CH_2Cl_2 was treated with Dess-Martin periodinane (580 mg, 1.37 mmol, 0.68 equiv.) at 0 °C for 10 min. The reaction was quenched with *sat. aq.* $Na_2S_2O_3$/*sat. aq.* $NaHCO_3$ (v/v 1/1), and the resulting mixture was extracted with EtOAc. The organic layer was washed with brine, dried (Na_2SO_4), filtered and concentrated *in vacuo*. The residue was purified by Hi-Flash

silica gel column chromatography (L size, hexane/EtOAc = 95/5 to 75/25 gradient elution) to afford the title compound (247 mg, 42% over 2 steps) as a yellow oil.
^{1}H NMR ($CDCl_3$, 400 MHz) δ 9.94 (s, 1H, C*H*O), 7.88 (d, 2H, H-2, H-6, *J* = 8.1 Hz), 7.61 (d, 2H, H-3, H-5, *J* = 8.1 Hz), 7.12 (br s, 1H, N*H*), 4.85 (s, 2H, O-CH_2CCl$_3$); ^{13}C NMR ($CDCl_3$, 100 MHz) δ 191.3, 151.2, 143.0, 132.3, 131.5, 118.5, 95.0, 74.8; ESIMSLR *m/z* 382.0 [(M+2MeOH+Na)$^+$].

2,2,2-trichloroethyl *N*-(3-formylphenyl)carbamate

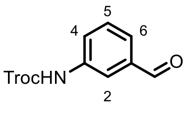

A solution of 3-aminobenzyl alcohol (246 mg, 2.00 mmol) and pyridine (323 µL, 4.00 mmol, 2.0 equiv.) in CH_2Cl_2 was treated with TrocCl (241 µL, 1.80 mmol, 0.9 equiv.) at 0 °C for 2 h. Sodium hydroxide (0.5 M in H_2O, 2 mL) was added to the reaction mixture and the mixture was stirred for 6 h. The resulting mixture was partitioned between EtOAc and 1 M *aq.* HCl, and the organic layer was washed with brine, dried (Na_2SO_4), filtered and concentrated *in vacuo*. A solution of the crude in CH_2Cl_2 was treated with Dess-Martin periodinane (580 mg, 1.37 mmol, 0.68 equiv.) at 0 °C for 10 min. The reaction was quenched with *sat. aq.* $Na_2S_2O_3$/*sat. aq.* $NaHCO_3$ (v/v 1/1), and the resulting mixture was extracted with EtOAc. The organic layer was washed with brine, dried (Na_2SO_4), filtered and concentrated *in vacuo*. The residue was purified by Hi-Flash silica gel column chromatography (L size, hexane/EtOAc = 95/5 to 75/25 gradient elution) to afford the title compound (211 mg, 36% over 2 steps) as a yellow oil.
^{1}H NMR ($CDCl_3$, 400 MHz) δ 10.01 (s, 1H, C*H*O), 7.96 (s, 1H, H-2), 7.73 (br d, 1H, H-6, *J* = 7.2 Hz), 7.65 (d, 1H, H-4, *J* = 7.6 Hz), 7.53 (t, 1H, H-5, *J* = 7.9 Hz), 7.03 (br s, 1H, N*H*), 4.85 (s, 2H, O-CH_2CCl$_3$); ^{13}C NMR ($CDCl_3$, 100 MHz) δ 192.1, 151.6, 138.2, 137.4, 130.1, 125.6, 124.7, 119.6, 95.2, 74.7; ESIMSLR *m/z* 350.0 [(M+MeOH+Na)$^+$]

tert-Butyl 5-*O*-[5-(*tert*-butyloxycarbonylamino)-5-deoxy-2,3-*O*-(3-pentylidene)-β-D-*ribo*-pentofuranosyl]-6-deoxy-2,3-*O*-isopropylidene-6-[4-(2,2,2-trichloroethylcarbonylamino)benzylamino]-1-(uracil-1-yl)-β-D-*glycelo*-L-*taro*-heptofuranuronate (**S13**)

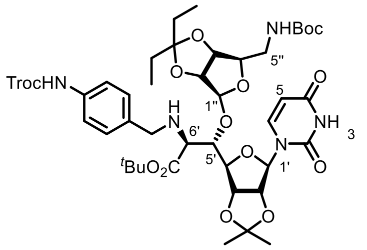

According to general procedure A, **S1** (50.0 mg, 70.1 µmol) and 2,2,2-trichloroethyl *N*-(4-formylphenyl)carbamate (31.1 mg, 105 µmol, 1.5 equiv.) were used to afford **S13** (56.5 mg, 81%) as a white solid after silica gel column chromatography (ϕ1.1×7 cm, MeOH/$CHCl_3$ = 0-1%).

^{1}H NMR ($CDCl_3$, 400 MHz) δ 8.21 (br s, 1H, N*H*-3), 7.35-7.28 (m, 4H, Ar), 7.20 (d, 1H, H-6, $J_{6,5}$ = 8.2Hz), 6.87 (br s, 1H, TrocN*H*), 5.93 (br s, 1H, BocN*H*), 5.66 (dd, 1H, H-5, $J_{5,6}$ = 8.2, $J_{5,3}$ = 1.8 Hz), 5.51 (d, 1H, H-1', $J_{1',2'}$ = 1.8 Hz), 5.07 (dd, 1H, H-2', $J_{2',3'}$ = 6.3, $J_{2',1'}$ = 1.8 Hz), 5.01 (br s, 1H, H-1''), 4.82 (s, 2H, OCH_2CCl$_3$), 4.74 (dd, 1H, H-3', $J_{3',2'}$ = 6.3, $J_{3',4'}$ = 4.5 Hz), 4.61 (dd, 1H, H-4', $J_{4',5'}$ = 8.6, $J_{4',3'}$ = 4.5 Hz), 4.56-4.51 (m, 2H, H-2'', H-3''), 4.26-4.23 (m, 1H, H-4''), 4.08 (br d, 1H, H-5', *J* = 9.1 Hz), 3.90 (d, 1H, ArCH_2, J_{gem} = 13.1 Hz), 3.57 (d, 1H, ArCH_2, J_{gem} = 13.1 Hz), 3.28 (d, 1H, H-6', *J* = 1.8 Hz), 3.24-3.17 (m, 1H, H-5''), 3.13-3.05 (m, 1H, H-5''), 1.62-1.34 (m, 28H, ^{t}Bu×2, pentylidene-CH_2×2, isopropylidene-CH_3×2), 0.78 (t, 6H, pentylidene-CH_3×2, *J* = 7.5 Hz); ^{13}C NMR ($CDCl_3$, 100 MHz) δ 172.2, 163.7, 156.3, 151.7, 150.0, 143.4, 136.2, 135.7, 129.3, 118.8, 116.1, 114.7, 112.8, 102.4, 95.7, 95.4,

87.7, 87.0, 86.7, 84.6, 82.6, 82.2, 81.9, 81.7, 79.0, 74.5, 61.2, 51.6, 43.3, 29.4, 28.8, 28.5, 28.3, 27.3, 25.6, 8.5, 7.4; ESIMS-HR *m/z*: $[M+H]^+$ calcd. for $C_{43}H_{61}Cl_3N_5O_{15}$ 992.3224, found 992.3250; $[\alpha]^{22}_D$ +3.70 (*c* 0.64, $CHCl_3$).

tert-Butyl 5-*O*-[5-(*tert*-butyloxycarbonylamino)-5-deoxy-2,3-*O*-(3-pentylidene)-β-D-*ribo*-pentofuranosyl]-6-deoxy-2,3-*O*-isopropylidene-6-[3-(2,2,2-trichloroethylcarbonylamino)benzylamino]-1-(uracil-1-yl)-β-D-*glycelo*-L-*taro*-heptofuranuronate (**S14**)

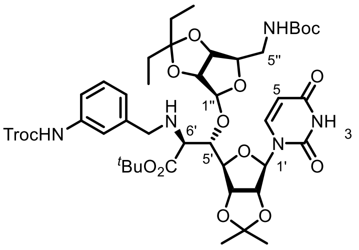

According to general procedure A, **S1** (50.0 mg, 70.1 μmol) and 2,2,2-trichloroethyl *N*-(3-formylphenyl)carbamate (31.1 mg, 105 μmol, 1.5 equiv.) was used to afford **S14** (56.1 mg, 81%) as a white solid after silica gel column chromatography (ϕ1.1×7 cm, MeOH/$CHCl_3$ = 0-1%).

^1H NMR ($CDCl_3$, 400 MHz) δ 8.19 (br s, 1H, N*H*-3), 7.49-7.45 (m, 1H, Ar), 7.32-7.19 (m, 3H, Ar, H-6), 7.08-7.02 (m, 2H, Ar, TrocN*H*), 5.92 (br s, 1H, BocN*H*), 5.65 (d, 1H, H-5, $J_{5,6}$ = 7.7 Hz), 5.53 (br s, 1H, H-1'), 5.12 (br d, 1H, H-2', J = 6.3 Hz), 4.99 (br s, 1H, H-1"), 4.83 (s, 2H, OCH_2CCl_3), 4.80 (dd, 1H, H-3', $J_{3',2'}$ = 6.3, $J_{3',4'}$ = 4.5 Hz), 4.63-4.52 (m, 3H, H-4', H-2", H-3"), 4.23 (dd, 1H, H-4", J = 6.3, J = 4.1 Hz), 4.11 (br d, 1H, H-5', J = 8.2 Hz), 3.94 (d, 1H, ArCH_2, J_{gem} = 13.1 Hz), 3.62 (d, 1H, ArCH_2, J_{gem} = 13.1 Hz), 3.29 (br s, 1H, H-6'), 3.26-3.18 (m, 1H, H-5"), 3.15-3.07 (m, 1H, H-5"), 1.62-1.36 (m, 28H, ^{t}Bu×2, pentylidene-CH_2×2, isopropylidene-CH_3×2), 0.78 (t, 6H, pentylidene-CH_3×2, J = 7.3 Hz); ^{13}C NMR ($CDCl_3$, 100 MHz) δ 172.1, 163.6, 156.4, 151.6, 150.1, 143.5, 141.1, 137.4, 129.2, 124.1, 118.6, 117.6, 116.1, 114.7, 112.8, 102.5, 95.7, 95.4, 87.8, 87.0, 86.7, 84.5, 82.6, 82.2, 81.7, 79.1, 74.5, 61.1, 51.7, 43.4, 29.4 28.8, 28.5, 18.3, 27.3, 25.6, 8.6, 7.4; ESIMS-HR *m/z*: $[M+H]^+$ calcd. for $C_{43}H_{61}Cl_3N_5O_{15}$ 992.3224, found 992.3250; $[\alpha]^{23}_D$ -0.51 (*c* 0.71, $CHCl_3$).

General procedure D (Staudinger reaction, and amide formation)

A solution of **S11** or **S12** (1.0 equiv.) in THF/H_2O (0.7 mL, 5/2 v/v) was treated with PPh_3 (1.2 equiv.) at 45 °C for 2.5~5 h. The reaction mixture was cooled and partitioned between CH_2Cl_2 and 0.1 M *aq.* HCl. The aqueous layer was washed with CH_2Cl_2. The organic layer was extracted with 0.1 M *aq.* HCl. The combined aqueous layer was neutralized with $NaHCO_3$, and the resulting mixture was extracted with EtOAc. The organic layer was washed with brine, dried (Na_2SO_4), filtered and concentrated *in vacuo*. A solution of the crude amine and carboxylic acid (1.5 equiv.) in CH_2Cl_2 (1 mL) was treated with HOBt·H_2O (1.5 equiv.), EDCI (1.4 equiv.) and $^i Pr_2NEt$ (1.5 equiv.) at room temperature for 30 min. The reaction was quenched with *sat. aq.* $NaHCO_3$, and the resulting mixture was extracted with EtOAc. The organic layer was washed with brine, dried (Na_2SO_4), filtered and concentrated *in vacuo*. The residue was purified by silica gel column chromatograph to afford products.

General procedure E (removal of Troc, and amide formation)

A solution of **S13** or **S14** (1.0 equiv.) in MeOH (1 mL) was treated with NH_4Cl (30 equiv.) and Zn powder (activated with HCl, 16 equiv.) at rt for 2.5~5 h. The reaction mixture was filtered off through a Celite pad, and the filtrate was concentrated *in vacuo*. The residue was partitioned between $CHCl_3$ and *sat. aq.* $NaHCO_3$/brine (1/1), and the aqueous layer was re-extracted with $CHCl_3$. The combined organic layer was dried (Na_2SO_4), filtered and concentrated *in*

vacuo. A solution of the crude amine and carboxylic acid (1.5 equiv.) in CH_2Cl_2 (1 mL) was treated with HOBt·H_2O (1.5 equiv.), EDCI (1.4 equiv.) and ${}^{i}Pr_2NEt$ (1.5 equiv.) at room temperature for 5 h. Additional carboxylic acid, EDCI, and HOBt·H_2O were added to the reaction mixture. The reaction was quenched with *sat. aq.* $NaHCO_3$, and the resulting mixture was extracted with EtOAc. The organic layer was washed with brine, dried (Na_2SO_4), filtered and concentrated *in vacuo*. The residue was purified by silica gel column chromatography to afford products.

Protected *p*Lys amide (**S15**)

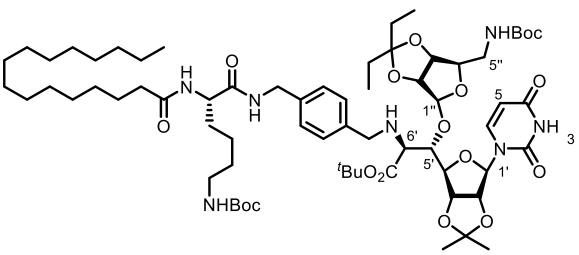

According to general procedure D, **S11** (11.5 mg, 13.4 μmol) and palmitoyl-Lys(Boc)-OH (9.7 mg, 20.1 μmol) were used to afford **S15** (10.8 mg, 62% over 2 steps) as a white solid after silica gel column chromatography (ϕ0.8×8 cm, MeOH/$CHCl_3$ = 1-2%).

^{1}H NMR (DMSO-d_6, 400 MHz) δ 11.39 (s, 1H, N*H*-3), 8.31 (t, 1H, amide-N*H*, J = 5.9 Hz), 7.86 (d, 1H, Lys-α-N*H*, J = 8.2 Hz), 7.78 (d, 1H, H-6, $J_{6,5}$ = 7.7 Hz), 7.23 (d, 2H, Ar, J = 8.2 Hz), 7.14 (d, 2H, Ar, J = 8.2 Hz), 6.72 (t, 1H, Lys-ε-N*H*, J = 5.4 Hz), 6.66 (t, 1H, N*H*-5'', J = 6.1 Hz), 5.77 (d, 1H, H-1', $J_{1',2'}$ = 1.8 Hz), 5.59 (d, 1H, H-5, $J_{5,6}$ = 7.7 Hz), 5.11 (dd, 1H, H-2', $J_{2',3'}$ = 6.4, $J_{2',1'}$ = 1.8 Hz), 4.94 (s, 1H, H-1''), 4.69 (dd, 1H, H-3', $J_{3',2'}$ = 6.4, $J_{3',4'}$ = 4.5 Hz), 4.54-4.49 (m, 2H, H-2'', H-3''), 4.40 (dd, 1H, H-4', $J_{4',5'}$ = 8.6, $J_{4',3'}$ = 4.5 Hz), 4.29-4.17 (m, 3H, Lys-α-C*H*, ArC*H*$_2$), 4.11 (br d, 1H, H-5', J = 8.6 Hz), 3.92 (dd, 1H, H-4'', J = 8.2, J = 5.9 Hz), 3.81 (dd, 1H, ArC*H*$_2$, J_{gem} = 12.4, J = 2.9 Hz), 3.50 (dd, 1H, ArC*H*$_2$, J_{gem} = 12.4, J = 7.0 Hz), 3.20 (d, 1H, H-6', J = 12.2 Hz), 3.03-2.92 (m, 1H, H-5''), 2.90-2.82 (m, 3H, H-5'', Lys-ε-C*H*$_2$), 2.15-1.99 (m, 3H, NH-6', acyl-α-C*H*$_2$), 1.66-1.14 (m, 69H, Lys-β-C*H*$_2$, Lys-γ-C*H*$_2$, Lys-δ-C*H*$_2$, isopropylidene-C*H*$_3$×2, pentylidene-C*H*$_2$×2, ^{t}Bu, acyl C*H*$_2$), 0.85 (t, 3H, C*H*$_3$, J = 6.8 Hz), 0.72 (t, 3H, C*H*$_3$, J = 7.3 Hz), 0.68 (t, 3H, C*H*$_3$, J = 7.3 Hz); ^{13}C NMR (DMSO-d_6, 100 MHz) δ 172.2, 171.9, 171.3, 163.4, 155.5, 155.5, 150.4, 143.9, 138.4, 137.9, 127.9, 126.9, 115.0, 113.3, 111.4, 101.6, 93.3, 86.7, 85.6, 85.3, 83.9, 81.6, 81.5, 81.2, 77.9, 77.3, 52.4, 51.2, 42.7, 41.7, 35.2, 31.7, 31.3, 29.2, 29.0, 29.0, 29.0, 28.8, 28.7, 28.6, 28.3, 28.1, 28.0, 27.8, 27.6, 27.1, 25.3, 22.8, 22.1, 13.9, 8.2, 7.1; ESIMS-HR *m/z*: $[M+H]^+$ calcd. for $C_{68}H_{112}N_7O_{17}$ 1298.8109, found 1298.8118; $[\alpha]^{25}_D$ -3.27 (*c* 0.50, $CHCl_3$).

Protected *p*Arg amide (**S17**)

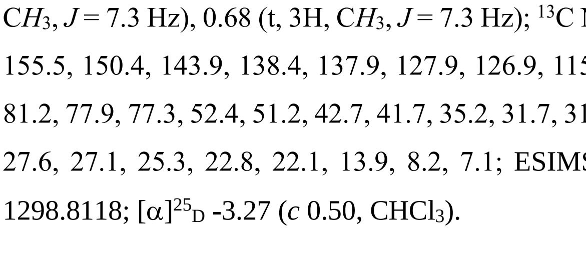

According to general procedure D, **S11** (20.0 mg, 23.3 μmol) and palmitoyl-Arg(Pbf)-OH (23.3 mg, 35.0 μmol) were used to afford **S17** (27.5 mg, 80% over 2 steps) as a white solid after silica gel column chromatography (ϕ0.8×8 cm, MeOH/$CHCl_3$ = 1-2%).

^{1}H NMR (DMSO-d_6, 500 MHz) δ 11.39 (d, 1H, N*H*-3, $J_{NH-3,5}$ = 2.3 Hz), 8.32 (t, 1H, amide-N*H*, J = 5.7 Hz), 7.89 (d, 1H, Arg-α-N*H*, J = 8.0 Hz), 7.78 (d, 1H, H-6, $J_{6,5}$ = 8.0 Hz), 7.22 (d, 2H, Ar, J = 8.0 Hz), 7.14 (d, 2H, Ar, J = 8.0 Hz), 6.65 (t, 1H, N*H*-5'', J = 6.0 Hz), 6.40 (br s, 1H, Arg-N*H*), 5.77 (d, 1H, H-1', $J_{1',2'}$ = 1.7 Hz), 5.59 (dd, 1H, H-5,

$J_{5,6}$ = 8.0, $J_{6,NH-3}$ = 2.3 Hz), 5.10 (dd, 1H, H-2', $J_{2',3'}$ = 6.3, $J_{2',1'}$ = 1.7 Hz), 4.94 (s, 1H, H-1''), 4.69 (t, 1H, H-3', J = 5.2 Hz), 4.53-4.49 (m, 2H, H-2'', H-3''), 4.40 (dd, 1H, H-4', $J_{4',5'}$ = 9.2, $J_{4',3'}$ = 4.6 Hz), 4.31-4.14 (m, 3H, Arg-α-C*H*, ArC*H*2), 4.11 (br d, 1H, H-5', J = 10.3 Hz), 3.92 (dd, 1H, H-4'', J = 8.6, J = 5.7 Hz), 3.80 (br d, 1H, ArC*H*2, J = 11.5 Hz), 3.50 (dd, 1H, ArC*H*2, J_{gem} = 13.8, J = 6.3 Hz), 3.21 (d, 1H, H-6', J = 10.9 Hz), 3.05-2.94 (m, 5H, H-5'', Arg-δ-C*H*2, Pbf), 2.99-2.83 (m, 1H, H-5''), 2.48 (s, 3H, Pbf), 2.42 (s, 3H, Pbf), 2.10 (t, 2H, acyl-α-C*H*2, J = 7.2 Hz), 2.09-1.98 (br s, 1H, N*H*-6'), 2.00 (s, 3H, Pbf), 1.68-1.18 (m, 64H, Arg-β-C*H*2, Arg-γ-C*H*2, Pbf CH3, isopropylidene-C*H*3×2, pentylidene-C*H*2×2, *t*Bu, acyl C*H*2), 0.85 (t, 3H, C*H*3, J = 6.9 Hz), 0.72 (t, 3H, C*H*3, J = 7.5 Hz), 0.69 (t, 3H, C*H*3, J = 7.5 Hz); ^{13}C NMR (DMSO-d_6, 100 MHz) δ 172.3, 171.7, 171.4, 165.2, 163.4, 157.5, 156.1, 155.5, 150.5, 144.0, 138.5, 137.9, 137.3, 134.2, 131.4, 127.9, 127.0, 124.3, 116.3, 115.0, 113.4, 111.4, 101.7, 93.4, 86.8, 86.3, 85.7, 85.4, 83.9, 81.6, 81.5, 81.2, 79.2, 77.9, 67.0, 60.9, 52.2, 51.3, 42.7, 42.5, 41.8, 35.2, 31.3, 29.5, 29.1, 29.1, 29.0, 28.9, 28.8, 28.7, 28.3, 28.1, 27.8, 27.6, 27.1, 25.3, 22.1, 19.0, 17.6, 14.0, 12.3, 8.3, 7.1; ESIMS-HR *m*/*z*: $[M+H]^+$ calcd. for $C_{76}H_{120}N_9O_{18}S$ 1478.8467, found 1478.8527; $[\alpha]^{22}_D$ -11.44 (*c* 0.59, $CHCl_3$).

Protected *m*Lys amide (**S16**)

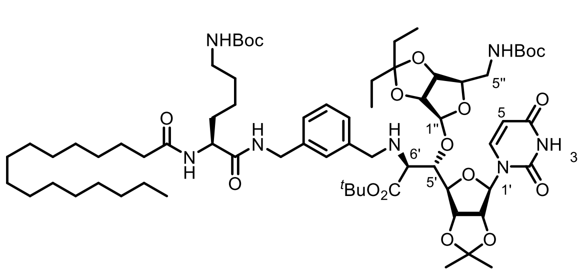

According to general procedure D, **S12** (22.0 mg, 25.6 μmol) and palmitoyl-Lys(Boc)-OH (18.6 mg, 38.4 μmol) were used to afford **S16** (23.2 mg, 70% over 2 steps) as a white solid after silica gel column chromatography (ϕ1.1×7 cm, MeOH/$CHCl_3$ = 1-2%).

^{1}H NMR (DMSO-d_6, 500 MHz) δ 11.38 (s, 1H, N*H*-3), 8.30 (t, 1H, amide-N*H*, J = 6.0 Hz), 7.85 (d, 1H, Lys-α-N*H*, J = 8.0 Hz), 7.77 (d, 1H, H-6, $J_{6,5}$ = 8.0 Hz), 7.25-7.20 (m, 2H, Ar), 7.12 (s, 1H, Ar), 7.09 (d, 1H, Ar, J = 6.9 Hz), 6.70 (t, 1H, Lys-ε-N*H*, J = 5.2 Hz), 6.66 (t, 1H, N*H*-5'', J = 6.0 Hz), 5.77 (d, 1H, H-1', $J_{1',2'}$ = 1.7 Hz), 5.59 (d, 1H, H-5, $J_{5,6}$ = 8.0 Hz), 5.11 (dd, 1H, H-2', $J_{2',3'}$ = 6.3, $J_{2',1'}$ = 1.7 Hz), 4.95 (s, 1H, H-1''), 4.71 (dd, 1H, H-3', $J_{3',2'}$ = 6.3, $J_{3',4'}$ = 4.6 Hz), 4.54-4.51 (m, 2H, H-2'', H-3''), 4.40 (dd, 1H, H-4', $J_{4',5'}$ = 8.9, $J_{4',3'}$ = 4.6 Hz), 4.26-4.20 (m, 3H, Lys-α-C*H*, ArC*H*2), 4.12 (dd, 1H, H-5', $J_{5',4'}$ = 8.9, $J_{5',6'}$ = 1.4 Hz), 3.92 (dd, 1H, H-4'', J = 8.6, J = 5.7 Hz), 3.80 (br d, 1H, ArC*H*2, J = 10.3 Hz), 3.51 (dd, 1H, ArC*H*2, J_{gem} = 13.5, J = 7.2 Hz), 3.23 (br d, 1H, H-6', J = 10.9 Hz), 3.00-2.95 (m, 1H, H-5''), 2.89-2.83 (m, 3H, H-5'', Lys-ε-C*H*2), 2.15-2.07 (m, 2H, acyl-α-C*H*2), 2.06-2.00 (m, 1H, N*H*-6'), 1.65-1.17 (m, 69H, Lys-β-C*H*2, Lys-γ-C*H*2, Lys-δ-C*H*2, isopropylidene-C*H*3×2, pentylidene-C*H*2×2, *t*Bu, acyl C*H*2), 0.85 (t, 3H, C*H*3, J = 6.9 Hz), 0.72 (t, 3H, C*H*3, J = 7.5 Hz), 0.68 (t, 3H, C*H*3, J = 7.5 Hz); ^{13}C NMR (DMSO-d_6, 100 MHz) δ 172.2, 171.9, 171.4, 163.4, 155.6, 150.5, 144.0, 140.0, 139.3, 128.2, 126.9, 126.4, 125.5, 115.0, 113.3, 111.4, 101.7, 93.4, 86.7, 85.7, 85.4, 83.9, 81.6, 81.5, 81.3, 77.9, 77.3, 61.1, 52.5, 51.6, 42.7, 42.0, 35.2, 31.8, 31.3, 29.2, 29.1, 28.9, 28.8, 28.7, 28.3, 28.1, 27.8, 27.1, 25.3, 22.8, 22.1, 14.0, 8.3, 7.1; ESIMS-HR *m*/*z*: $[M+H]^+$ calcd. for $C_{68}H_{112}N_7O_{17}$ 1298.8109, found 1298.8191; $[\alpha]^{22}_D$ -6.07 (*c* 0.51, $CHCl_3$).

Protected *m*Arg amide (**S18**)

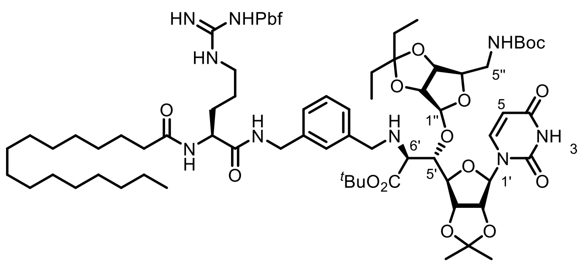

According to general procedure D, **S12** (22.0 mg, 25.6 μmol) and palmitoyl-Arg(Pbf)-OH (25.5 mg, 38.4 μmol) were used to afford **S18** (22.7 mg, 60% over 2 steps) as a white solid after silica gel column chromatography (ϕ1.1×7 cm, MeOH/$CHCl_3$ = 1-2%).

^{1}H NMR (DMSO-d_6, 500 MHz) δ 11.39 (s, 1H, N*H*-3), 8.31 (t, 1H, amide-N*H*, *J* = 5.4 Hz), 7.89 (d, 1H, Arg-α-N*H*, *J* = 8.6 Hz), 7.77 (d, 1H, H-6, $J_{6,5}$ = 8.0 Hz), 7.24-7.20 (m, 2H, Ar), 7.12 (s, 1H, Ar), 7.08 (d, 1H, Ar, *J* = 6.3 Hz), 6.66 (t, 1H, N*H*-5", *J* = 6.0 Hz), 6.38 (br s, 1H, Arg-N*H*), 5.77 (d, 1H, H-1', $J_{1',2'}$ = 1.7 Hz), 5.60 (dd, 1H, H-5, $J_{5,6}$ = 8.0, $J_{5,3}$ = 1.7 Hz), 5.12 (dd, 1H, H-2', $J_{2',3'}$ = 6.3, $J_{2',1'}$ = 1.7 Hz), 4.95 (s, 1H, H-1"), 4.72 (dd, 1H, H-3', $J_{3',2'}$ = 6.3, $J_{3',4'}$ = 4.6 Hz), 4.54-4.51 (m, 2H, H-2", H-3"), 4.41 (dd, 1H, H-4', $J_{4',5'}$ = 8.6, $J_{4',3'}$ = 4.6 Hz), 4.30-4.17 (m, 3H, Arg-α-C*H*, ArCH_2), 4.12 (br d, 1H, H-5', *J* = 8.6 Hz), 3.92 (dd, 1H, H-4", *J* = 8.6, *J* = 5.7 Hz), 3.81 (dd, 1H, ArCH_2, J_{gem} = 13.2, *J* = 3.4 Hz), 3.51 (dd, 1H, ArCH_2, J_{gem} = 13.2, *J* = 7.5 Hz), 3.23 (br d, 1H, H-6', *J* = 12.0 Hz), 3.04-2.95 (m, 5H, H-5", Arg-δ-CH_3, Pbf-CH_2), 2.89-2.83 (m, 1H, H-5"), 2.48 (s, 3H, Pbf-CH_3), 2.42 (s, 3H, Pbf-CH_3), 2.10 (t, 2H, acyl-α-CH_2, *J* = 7.2 Hz), 2.07-1.99 (m, 1H, N*H*-6'), 1.66-1.17 (m, 55H, Arg-β-CH_2, Arg-γ-CH_2, Pbf-CH_3, isopropylidene-CH_3×2, pentylidene-CH_2×2, ^{t}Bu, acyl-CH_2), 0.84 (t, 3H, CH_3, *J* = 6.9 Hz), 0.72 (t, 3H, CH_3, *J* = 7.5 Hz), 0.68 (t, 3H, CH_3, *J* = 7.5 Hz); ^{13}C NMR (DMSO-d_6, 100 MHz) δ 172.2, 171.6, 171.3, 163.3, 157.4, 156.0, 155.5, 150.4, 143.9, 140.0, 139.1, 137.3, 134.2, 131.4, 128.2, 127.0, 126.4, 125.5, 124.3, 116.2, 115.0, 113.3, 111.4, 101.7, 93.3, 86.7, 86.2, 85.7, 85.3, 83.8, 81.6, 81.5, 81.2, 77.9, 61.1, 52.1, 51.6, 42.7, 42.5, 42.0, 35.2, 31.3, 29.0, 29.0, 28.8, 28.7, 28.3, 28.1, 27.8, 27.1, 25.3, 22.1, 18.9, 17.6, 13.9, 12.2, 8.2, 7.1; ESIMS-HR *m/z*: $[M+H]^+$ calcd. for $C_{76}H_{120}N_9O_{18}S$ 1478.8467, found 1478.8527; $[\alpha]^{22}_D$ -8.46 (*c* 0.50, $CHCl_3$).

Protected *p*Lys anilide (**S19**)

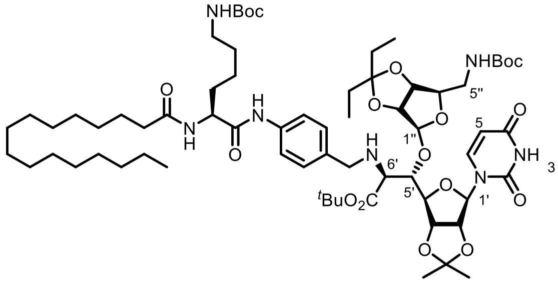

According to general procedure E, **S13** (26.0 mg, 26.2 μmol) and palmitoyl-Lys(Boc)-OH (19.0×2 mg, 39.3×2 μmol) were used to afford **S19** (14.9 mg, 44% over 2 steps) as a white solid after silica gel column chromatography (ϕ1.1×7 cm, MeOH/$CHCl_3$ = 0-1-2%).

^{1}H NMR (DMSO-d_6, 500 MHz) δ 11.39 (s, 1H, N*H*-3), 9.94 (s, 1H, amide-N*H*), 7.99 (d, 1H, Lys-α-N*H*, *J* = 7.5 Hz), 7.77 (d, 1H, H-6, $J_{6,5}$ = 8.0 Hz), 7.52 (d, 2H, Ar, *J* = 8.6 Hz), 7.21 (d, 2H, Ar, *J* = 8.6 Hz), 6.73 (t, 1H, Lys-ε-N*H*, *J* = 5.7 Hz), 6.64 (t, 1H, N*H*-5", *J* = 6.3 Hz), 5.76 (d, 1H, H-1', $J_{1',2'}$ = 1.7 Hz), 5.59 (dd, 1H, H-5, $J_{5,6}$ = 8.0, $J_{5,3}$ = 2.3 Hz), 5.11 (dd, 1H, H-2', $J_{2',3'}$ = 6.3, $J_{2',1'}$ = 1.7 Hz), 4.93 (s, 1H, H-1"), 4.64 (dd, 1H, H-3', $J_{3',2'}$ = 6.3, $J_{3',4'}$ = 4.6 Hz), 4.54-4.49 (m, 2H, H-2", H-3"), 4.40-4.33 (m, 2H, H-4', Lys-α-C*H*), 4.10 (br d, 1H, H-5', $J_{5',4'}$ = 9.7 Hz), 3.92 (dd, 1H, H-4", *J* = 7.7, *J* = 6.0 Hz), 3.80 (br d, 1H, ArCH_2, *J* = 12.6 Hz), 3.47 (br d, 1H, ArCH_2, J_{gem} = 12.6 Hz), 3.16 (br d, 1H, H-6', *J* = 10.3 Hz), 3.01-2.96 (m, 1H, H-5"), 2.91-2.84 (m, 3H, H-5", Lys-ε-CH_2), 2.15-2.04 (m, 3H, N*H*-6', acyl-α-CH_2), 1.68-1.15 (m, 69H, Lys-β-CH_2, Lys-γ-CH_2, Lys-δ-CH_2, isopropylidene-CH_3×2, pentylidene-CH_2×2, ^{t}Bu, acyl

CH_2), 0.85 (t, 3H, CH_3, J = 6.9 Hz), 0.71 (t, 3H, CH_3, J = 7.7 Hz), 0.68 (t, 3H, CH_3, J = 7.7 Hz); ^{13}C NMR (DMSO-d_6, 100 MHz) δ 172.3, 171.4, 170.9, 163.4, 155.6, 155.5, 150.5, 144.0, 137.8, 134.6, 128.4, 119.0, 115.0, 113.3, 111.4, 101.6, 93.5, 86.8, 85.7, 85.4, 83.9, 81.6, 81.2, 77.9, 77.3, 60.4, 53.2, 50.9, 42.7, 35.1, 31.9, 31.3, 29.3, 29.1, 29.0, 29.0, 28.8, 28.8, 28.6, 28.3, 28.1, 27.8, 27.6, 27.0, 25.3, 25.3, 22.8, 22.1, 14.0, 8.3, 7.1; ESIMS-HR *m/z*: [M+H]$^+$ calcd. for $C_{67}H_{110}N_7O_{17}$ 1284.7953, found 1284.7983; $[\alpha]^{25}_D$ -5.01 (*c* 0.39, $CHCl_3$).

Protected *p*Arg anilide (**S21**)

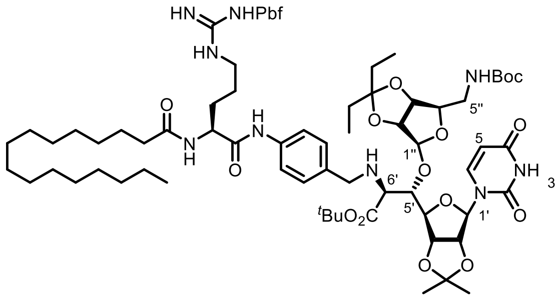

According to general procedure E, **S13** (26.0 mg, 26.2 μmol) and palmitoyl-Arg(Pbf)-OH (26.1×2 mg, 39.3×2 μmol) were used to afford **S21** (24.0 mg, 63% over 2 steps) as a white solid after silica gel column chromatography (ϕ1.1×8 cm, MeOH/$CHCl_3$ = 1-2%).

^{1}H NMR (DMSO-d_6, 500 MHz) δ 11.39 (s, 1H, NH-3), 9.97 (s, 1H, amide-NH), 8.03 (d, 1H, Arg-α-NH, J = 8.0 Hz), 7.78 (d, 1H, H-6, $J_{6,5}$ = 8.0 Hz), 7.53 (d, 2H, Ar, J = 8.0 Hz), 7.21 (d, 2H, Ar, J = 8.0 Hz), 6.64 (t, 1H, NH-5'', J = 5.7 Hz), 6.44-6.32 (br s, 1H, Arg-NH), 5.76 (d, 1H, H-1', $J_{1',2'}$ = 1.7 Hz), 5.59 (dd, 1H, H-5, $J_{5,6}$ = 8.0, $J_{5,3}$ = 1.7 Hz), 5.11 (dd, 1H, H-2', $J_{2',3'}$ = 6.3, $J_{2',1'}$ = 1.7 Hz), 4.94 (s, 1H, H-1''), 4.65 (dd, 1H, H-3', $J_{3',2'}$ = 6.3, $J_{3',4'}$ = 4.6 Hz), 4.54-4.49 (m, 2H, H-2'', H-3''), 4.41-4.36 (m, 2H, H-4', Arg-α-CH), 4.10 (br d, 1H, H-5', $J_{5',4'}$ = 9.2 Hz), 3.92 (dd, 1H, H-4'', J = 8.0, J = 5.7 Hz), 3.80 (br d, 1H, ArCH_2, J = 10.3 Hz), 3.51-3.45 (m, 1H, ArCH_2), 3.17 (br d, 1H, H-6', J = 11.5 Hz), 3.07-2.83 (m, 6H, H-5'', Arg-δ-CH_2, Pbf-CH_2), 2.46 (s, 3H, Pbf-CH_3), 2.41 (s, 3H, Pbf-CH_3), 2.12 (t, 2H, acyl-α-CH_2, J = 7.2 Hz), 2.13-2.03 (m, 1H, NH-6'), 1.98 (s, 3H, Pbf-CH_3), 1.69-1.15 (m, 64H, Arg-β-CH_2, Arg-γ-CH_2, isopropylidene-CH_3×2, pentylidene-CH_2×2, ^{t}Bu, acyl CH_2, Pbf-CH_3), 0.85 (t, 3H, CH_3, J = 6.9 Hz), 0.71 (t, 3H, CH_3, J = 7.5 Hz), 0.68 (t, 3H, CH_3, J = 7.5 Hz); ^{13}C NMR (DMSO-d_6, 100 MHz) δ 172.4, 171.4, 170.6, 163.4, 157.5, 156.1, 155.5, 150.5, 144.0, 137.7, 137.3, 134.7, 134.2, 131.5, 128.4, 124.3, 119.0, 116.3, 115.0, 113.3, 111.4, 101.7, 93.5, 86.8, 86.3, 85.7, 85.4, 83.9, 81.6, 81.2, 77.9, 60.5, 52.9, 50.9, 42.7, 42.5, 35.1, 31.3, 29.6, 29.1, 29.1, 28.9, 28.8, 28.7, 28.3, 28.2, 27.9, 27.0, 25.3, 25.3, 22.1, 19.0, 17.6, 13.3, 12.3, 8.3, 7.1; ESIMS-HR *m/z*: [M+H]$^+$ calcd. for $C_{75}H_{118}N_9O_{18}S$ 1486.8130, found 1464.8394; $[\alpha]^{24}_D$ -11.15 (*c* 0.67, $CHCl_3$).

Protected *m*Lys anilide (**S20**)

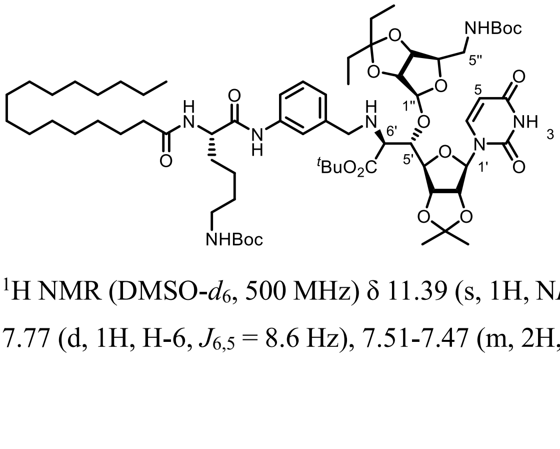

According to general procedure E, **S14** (26.0 mg, 26.2 μmol) and palmitoyl-Lys(Boc)-OH (19.0×2 mg, 39.3×2 μmol) were used to afford **S20** (10.1 mg, 30% over 2 steps) as a white solid after silica gel column chromatography (ϕ1.1×7 cm, MeOH/$CHCl_3$ = 0-1-2%, and preparative TLC MeOH/$CHCl_3$ = 4%).

^{1}H NMR (DMSO-d_6, 500 MHz) δ 11.39 (s, 1H, NH-3), 9.94 (s, 1H, amide-NH), 7.98 (d, 1H, Lys-α-NH, J = 8.0 Hz), 7.77 (d, 1H, H-6, $J_{6,5}$ = 8.6 Hz), 7.51-7.47 (m, 2H, Ar), 7.21 (t, 1H, Ar, J = 7.7 Hz), 7.01 (d, 1H, Ar, J = 7.5 Hz), 6.74

(t, 1H, Lys-α-N*H*, J = 5.5 Hz), 6.64 (t, 1H, N*H*-5'', J = 5.7 Hz), 5.77 (br s, 1H, H-1'), 5.59 (d, 1H, H-5, $J_{5,6}$ = 8.6 Hz), 5.09 (br d, 1H, H-2', J = 6.3 Hz), 4.96 (s, 1H, H-1''), 4.69 (br t, 1H, H-3', J = 5.2 Hz), 4.55-4.49 (m, 2H, H-2'', H-3''), 4.40-4.33 (m, 2H, H-4', Lys-α-C*H*), 4.10 (br d, 1H, H-5', $J_{5',4'}$ = 9.2 Hz), 3.92 (dd, 1H, H-4'', J = 8.3, J = 6.0 Hz), 3.76 (br d, 1H, ArCH_2, J = 12.0 Hz), 3.51-3.44 (m, 1H, ArCH_2), 3.20 (br d, 1H, H-6', J = 10.3 Hz), 3.02-2.96 (m, 1H, H-5''), 2.91-2.81 (m, 3H, H-5'', Lys-ε-CH_2), 2.18-2.07 (m, 2H, acyl-α-CH_2), 2.07-2.00 (m, 1H, N*H*-6'), 1.68-1.15 (m, 69H, Lys-β-CH_2, Lys-γ-CH_2, Lys-δ-CH_2, isopropylidene-CH_3×2, pentylidene-CH_2×2, ^{t}Bu, acyl CH_2), 0.85 (t, 3H, CH_3, J = 6.8 Hz), 0.71 (t, 3H, CH_3, J = 7.5 Hz), 0.68 (t, 3H, CH_3, J = 7.5 Hz); ^{13}C NMR (DMSO-d_6, 100 MHz) δ 172.3, 171.4, 170.9, 163.3, 155.5, 155.5, 150.4, 143.8, 140.5, 138.9, 128.5, 123.1, 119.0, 117.8, 115.0, 113.4, 111.3, 101.7, 93.1, 86.5, 85.6, 85.3, 83.8, 81.6, 81.4, 81.2, 81.1, 77.9, 77.3, 60.9, 53.2, 51.9, 42.7, 35.1, 31.8, 31.3, 29.3, 29.0, 29.0, 28.8, 28.7, 28.6, 28.2, 28.1, 27.8, 27.6, 27.0, 25.3, 22.8, 22.1, 13.9, 8.2, 7.1; ESIMS-HR *m*/*z*: $[M+H]^+$ calcd. for $C_{67}H_{110}N_7O_{17}$ 1284.7953, found 1284.8058; $[\alpha]^{24}_D$ +0.62 (*c* 0.60, $CHCl_3$).

Protected *m*Arg anilide (**S21**)

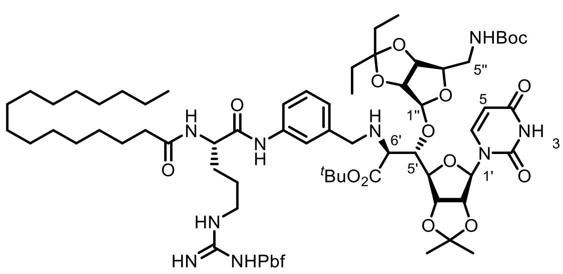

According to general procedure E, **S14** (26.0 mg, 26.2 μmol) and palmitoyl-Arg(Pbf)-OH (26.1×2 mg, 39.3×2 μmol) were used to afford **S21** (21.3 mg, 55% over 2 steps) as a white solid after silica gel column chromatography (ϕ1.1×8 cm, MeOH/$CHCl_3$ = 1-2-3%).

^{1}H NMR (DMSO-d_6, 500 MHz) δ 11.39 (d, 1H, N*H*-3, J = 1.7 Hz), 9.97 (s, 1H, amide-N*H*), 8.01 (d, 1H, Arg-α-N*H*, J = 7.5 Hz), 7.77 (d, 1H, H-6, $J_{6,5}$ = 8.0 Hz), 7.52-7.48 (m, 2H, Ar), 7.22 (t, 1H, Ar, J = 7.5 Hz), 7.02 (d, 1H, Ar, J = 7.5 Hz), 6.64 (t, 1H, N*H*-5'', J = 5.6 Hz), 6.43-6.31 (br s, 1H, Arg-N*H*), 5.77 (br s, 1H, H-1'), 5.59 (dd, 1H, H-5, $J_{5,6}$ = 8.0, $J_{5,3}$ = 1.7 Hz), 5.09 (br d, 1H, H-2', J = 6.3 Hz), 4.96 (s, 1H, H-1''), 4.69 (br t, 1H, H-3', J = 4.8 Hz), 4.54-4.49 (m, 2H, H-2'', H-3''), 4.42-4.37 (m, 2H, H-4', Arg-α-C*H*), 4.11 (br d, 1H, H-5', $J_{5',4'}$ = 8.6 Hz), 3.92 (dd, 1H, H-4'', J = 8.3, J = 5.4 Hz), 3.77 (br d, 1H, ArCH_2, J = 11.5 Hz), 3.51-3.45 (m, 1H, ArCH_2), 3.20 (br d, 1H, H-6', J = 11.5 Hz), 3.07-2.91 (m, 5H, H-5'', Arg-δ-CH_2, Pbf-CH_2), 2.88-2.81 (m, 1H, H-5''), 2.46 (s, 3H, Pbf-CH_3), 2.41 (s, 3H, Pbf-CH_3), 2.12 (t, 2H, acyl-α-CH_2, J = 7.5 Hz), 2.08-1.96 (m, 1H, N*H*-6'), 1.98 (s, 3H, Pbf-CH_3), 1.70-1.17 (m, 64H, Arg-β-CH_2, Arg-γ-CH_2, isopropylidene-CH_3×2, pentylidene-CH_2×2, ^{t}Bu, acyl CH_2, Pbf-CH_3), 0.85 (t, 3H, CH_3, J = 6.9 Hz), 0.71 (t, 3H, CH_3, J = 7.2 Hz), 0.69 (t, 3H, CH_3, J = 7.2 Hz); ^{13}C NMR (DMSO-d_6, 100 MHz) δ 172.4, 171.4, 170.7, 163.4, 157.5, 156.1, 155.5, 150.5, 143.9, 140.6, 138.9, 137.3, 134.2, 131.4, 128.5, 124.3, 123.1, 119.0, 117.8, 116.3, 115.0, 113.4, 111.3, 101.7, 93.1, 93.0, 86.5, 86.3, 85.6, 85.3, 83.9, 81.6, 81.3, 77.9, 60.9, 52.9, 51.9, 42.7, 42.5, 35.1, 31.3, 29.6, 29.1, 29.0, 28.8, 28.7, 28.7, 28.3, 28.1, 27.8, 27.1, 25.3, 22.1, 19.0, 17.6, 14.0, 12.3, 8.3, 7.1; ESIMS-HR *m*/*z*: $[M+H]^+$ calcd. for $C_{75}H_{118}N_9O_{18}S$ 1464.8310, found 1464.8394; $[\alpha]^{24}_D$ -1.16 (*c* 0.43, $CHCl_3$).

General procedure F (deprotection)

A solution of protected analogues was treated with 80% TFA/CH_2Cl_2 (1 mL) and H_2O (50 μL) at room temperature for 3-5 h. The reaction mixture was concentrated *in vacuo*. The resulting solid was purified by Hi-Flash reverse-phase

silica gel column chromatography (ODS-AQ 30 mm 120A; MeCN/H_2O 30% to 70% gradient elution containing 0.1% TFA) to afford products.

Palmitoyl-*p*Lys-amide (**1**)

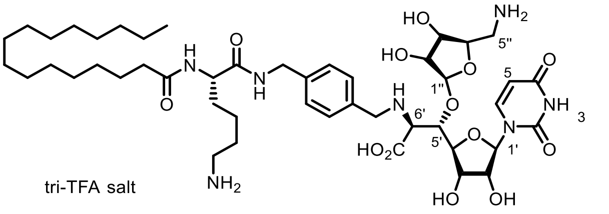

According to general procedure F, **S15** (10.0 mg, 7.70 μmol) was used to afford **1** (7.9 mg, 80%) as a white solid.

^{1}H NMR (CD_3OD, 400 MHz) δ 7.61 (d, 1H, H-6, $J_{6,5}$ = 8.2 Hz), 7.40 (d, 2H, Ar, J = 8.2 Hz), 7.33 (d, 2H, Ar, J = 8.2 Hz), 5.73 (d, 1H, H-5, $J_{5,6}$ = 8.2 Hz), 5.73 (d, 1H, H-1', $J_{1',2'}$ = 3.2 Hz), 5.15 (s, 1H, H-1''), 4.56 (d, 1H, H-5', $J_{5',4'}$ = 4.6 Hz), 4.47-4.31 (m, 5H, H-2', ArCH_2, Lys-α-C*H*), 4.25 (d, 1H, ArCH_2, J_{gem} = 12.8 Hz), 4.21 (t, 1H, H-3', J = 6.2 Hz), 4.15 (dd, 1H, H-4', $J_{4',3'}$ = 6.9, $J_{4',5'}$ = 4.6 Hz), 4.11-4.00 (m, 3H, H-2'', H-3'', H-4''), 3.94-3.90 (br s, 1H, H-6'), 3.24-3.16 (m, 2H, H-5''), 2.96-2.86 (m, 2H, Lys-ε-CH_2), 2.27 (t, 2H, acyl-α-CH_2, J = 7.3 Hz), 1.91-1.82 (m, 1H, Lys-β-CH_2), 1.76-1.58 (m, 5H, Lys-β-CH_2, Lys-δ-CH_2, acyl-β-CH_2), 1.55-1.39 (m, 2H, Lys-γ-CH_2), 1.38-1.25 (m, 24H, acyl-CH_2×12), 0.90 (t, 3H, acyl-CH_3, J = 6.9 Hz); ^{13}C NMR (CD_3OD, 100 MHz) δ 176.7, 174.5, 171.2, 165.9, 162.6 (q, $^2J_{C,F}$ = 36.6 Hz), 152.1, 143.6, 141.8, 131.5, 131.0, 129.1, 118.0 (q, $^1J_{C,F}$ = 291.9 Hz), 110.4, 103.4, 94.0, 86.6, 80.5, 77.3, 76.4, 73.7, 71.1, 64.3, 54.6, 52.5, 43.9, 43.6, 40.5, 36.9, 33.1, 32.4, 30.8, 30.7, 30.7, 30.5, 30.5, 30.4, 28.1, 26.9, 23.9, 23.7, 14.4; ESIMS-HR *m*/*z*: $[M+H]^+$ calcd. for $C_{46}H_{76}N_7O_{13}$ 934.5496, found 934.5477; $[\alpha]^{24}_D$ -1.45 (*c* 0.76, MeOH).

Palmitoyl-*m*Lys-amide (**2**)

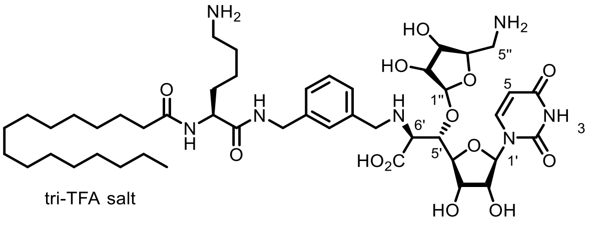

According to general procedure F, **S16** (22.2 mg, 17.1 μmol) was used to afford **2** (18.8 mg, 86%) as a white solid.

^{1}H NMR (CD_3OD, 400 MHz) δ 7.64 (d, 1H, H-6, $J_{6,5}$ = 8.1 Hz), 7.41-7.30 (m, 4H, Ar), 5.77 (d, 1H, H-1', $J_{1',2'}$ = 3.1 Hz), 5.72 (d, 1H, H-5, $J_{5,6}$ = 8.1 Hz), 5.16 (s, 1H, H-1''), 4.56 (br d, 1H, H-5', $J_{5',4'}$ = 4.5 Hz), 4.46-4.26 (m, 6H, H-2', ArCH_2×2, Lys-α-C*H*), 4.20 (dd, 1H, H-3', $J_{3',4'}$ = 7.4, $J_{3',2'}$ = 5.4 Hz), 4.16 (dd, 1H, H-4', $J_{4',3'}$ = 7.4, $J_{4',5'}$ = 4.9 Hz), 4.10-4.00 (m, 3H, H-2'', H-3'', H-4''), 3.92 (br s, 1H, H-6'), 3.25-3.16 (m, 2H, H-5''), 2.96-2.87 (m, 2H, Lys-ε-CH_2), 2.27 (t, 2H, acyl-α-CH_2, J = 7.6 Hz), 1.91-1.81 (m, 1H, Lys-β-CH_2), 1.77-1.55 (m, 5H, Lys-β-CH_2, Lys-δ-CH_2, acyl-β-CH_2), 1.55-1.38 (m, 2H, Lys-γ-CH_2), 1.37-1.23 (m, 24H, acyl-CH_2×12), 0.90 (t, 3H, acyl-CH_3, J = 6.7 Hz); ^{13}C NMR (CD_3OD, 100 MHz) δ 176.7, 174.6, 171.2, 165.9, 162.6 (q, $^2J_{C,F}$ = 35.7 Hz), 152.1, 143.4, 141.1, 132.4, 130.4, 130.3, 130.1, 129.6, 118.0 (q, $^1J_{C,F}$ = 291.3 Hz), 110.3, 103.2, 94.0, 86.0, 80.4, 77.5, 76.4, 74.1, 73.8, 71.1, 64.0, 54.7, 52.6, 43.9, 43.7, 40.4, 36.9, 33.1, 32.3, 30.8, 30.8, 30.7, 30.5, 30.5, 30.4, 28.1, 27.0, 23.9, 23.7, 14.5; ESIMS-HR *m*/*z*: $[M+H]^+$ calcd. for $C_{46}H_{76}N_7O_{13}$ 934.5496, found 934.5477; $[\alpha]^{24}_D$ -2.91 (*c* 0.69, MeOH).

Palmitoyl-*p*Arg-amide (**3**)

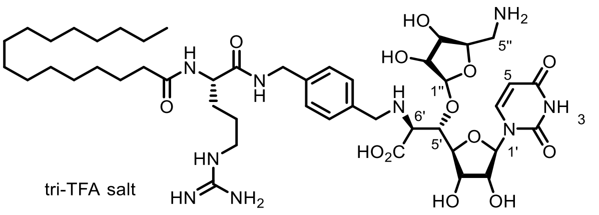

According to general procedure F, **S**17 (26.5 mg, 17.9 μmol) was used to afford **3** (19.4 mg, 83%) as a white solid.

^{1}H NMR (CD_3OD, 400 MHz) δ 7.61 (d, 1H, H-6, $J_{6,5}$ = 8.2 Hz), 7.40 (d, 2H, Ar, J = 8.2 Hz), 7.33 (d, 2H, Ar, J = 8.2 Hz), 5.73 (d, 1H, H-5, $J_{5,6}$ = 8.2 Hz), 5.72 (d, 1H, H-1', $J_{1',2'}$ = 3.2 Hz), 5.15 (s, 1H, H-1''), 4.56 (d, 1H, H-5', $J_{5',4'}$ = 5.0 Hz), 4.45-4.31 (m, 5H, H-2', ArCH_2, Arg-α-CH), 4.25 (d, 1H, ArCH_2, J_{gem} = 12.8 Hz), 4.21 (t, 1H, H-3', J = 6.2 Hz), 4.14 (dd, 1H, H-4', $J_{4',3'}$ = 6.9, $J_{4',5'}$ = 4.6 Hz), 4.11-3.99 (m, 3H, H-2'', H-3'', H-4''), 3.93 (br s, 1H, H-6'), 3.25-3.15 (m, 4H, H-5'', Arg-δ-CH_2), 2.28 (t, 2H, acyl-α-CH_2, J = 7.6 Hz), 1.93-1.85 (m, 1H, Arg-β-CH_2), 1.75-1.57 (m, 5H, Arg-β-CH_2, Arg-γ-CH_2, acyl-β-CH_2), 1.38-1.25 (m, 24H, acyl-CH_2×12), 0.90 (t, 3H, acyl-CH_3, J = 6.6 Hz); ^{13}C NMR (CD_3OD, 100 MHz) δ 176.7, 174.3, 171.2, 165.9, 162.5 (q, $^2J_{C,F}$ = 35.6 Hz), 158.7, 152.1, 143.6, 141.8, 131.5, 131.0, 129.1, 117.9 (q, $^1J_{C,F}$ = 289.0 Hz), 110.4, 103.3, 94.0, 86.5, 80.5, 77.3, 76.4, 73.7, 73.7, 71.1, 64.1, 54.4, 52.5, 43.9, 43.6, 41.9, 36.9, 33.1, 30.8, 30.7, 30.6, 30.4, 30.4, 30.2, 26.9, 26.5, 23.7, 14.4; ESIMS-HR *m*/*z*: $[M+H]^+$ calcd. for $C_{46}H_{76}N_9O_{13}$ 962.5557, found 962.5575; $[\alpha]^{24}_D$ -3.97 (*c* 0.73, MeOH).

Palmitoyl-*m*Arg-amide (**4**)

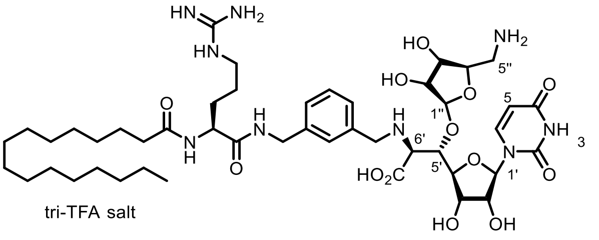

According to general procedure F, **S18** (21.7 mg, 14.7 μmol) was used to afford **4** (17.3 mg, 90%) as a white solid.

^{1}H NMR (CD_3OD, 400 MHz) δ 7.64 (d, 1H, H-6, $J_{6,5}$ = 8.1 Hz), 7.40-7.32 (m, 4H, Ar), 5.76 (d, 1H, H-1', $J_{1',2'}$ = 2.7 Hz), 5.72 (d, 1H, H-5, $J_{5,6}$ = 8.1 Hz), 5.17 (s, 1H, H-1''), 4.56 (dd, 1H, H-5', $J_{5',4'}$ = 4.9, $J_{5',6'}$ = 0.9 Hz), 4.44-4.25 (m, 6H, H-2', ArCH_2×2, Arg-α-CH), 4.21 (dd, 1H, H-3', $J_{3',4'}$ = 7.2, $J_{3',2'}$ = 5.8 Hz), 4.16 (dd, 1H, H-4', $J_{4',3'}$ = 7.2, $J_{4',5'}$ = 4.9 Hz), 4.10-4.00 (m, 3H, H-2'', H-3'', H-4''), 3.92-3.90 (br s, 1H, H-6'), 3.25-3.17 (m, 4H, H-5'', Arg-δ-CH_2), 2.28 (t, 2H, acyl-α-CH_2, J = 7.6 Hz), 1.94-1.83 (m, 1H, Arg-β-CH_2), 1.77-1.56 (m, 5H, Arg-β-CH_2, Arg-γ-CH_2, acyl-β-CH_2), 1.36-1.25 (m, 24H, acyl-CH_2×12), 0.90 (t, 3H, acyl-CH_3, J = 7.0 Hz); ^{13}C NMR (CD_3OD, 100 MHz) δ 176.7, 174.4, 171.3, 165.9, 162.7 (q, $^2J_{C,F}$ = 34.7 Hz), 158.6, 152.1, 143.4, 141.1, 132.4, 130.4, 130.3, 130.1, 129.7, 117.9 (q, $^1J_{C,F}$ = 286.1 Hz), 110.3, 103.2, 94.1, 86.1, 80.4, 77.5, 76.4, 74.1, 73.8, 71.1, 64.0, 54.5, 52.6, 43.9, 43.7, 41.9, 36.9, 33.1, 30.8, 30.7, 30.6, 30.5, 30.5, 30.4, 30.2, 27.0, 26.5, 23.7, 14.4; ESIMS-HR *m*/*z*: $[M+H]^+$ calcd. for $C_{46}H_{76}N_9O_{13}$ 962.5557, found 962.5575; $[\alpha]^{24}_D$ -5.73 (*c* 0.70, MeOH).

Palmitoyl-*p*Lys-anilide (**5**)

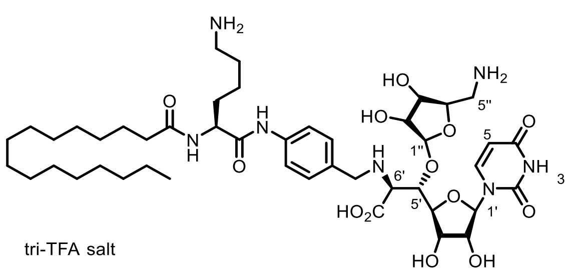

According to general procedure F, **S19** (21.0 mg, 16.3 μmol) was used to afford **5** (15.0 mg, 73%) as a white solid.

^{1}H NMR (CD_3OD, 400 MHz) δ 7.64 (d, 2H, Ar, *J* = 8.5 Hz), 7.62 (d, 1H, H-6, $J_{6,5}$ = 8.1 Hz), 7.42 (d, 2H, Ar, *J* = 8.5 Hz), 5.72 (d, 1H, H-5, $J_{5,6}$ = 8.1 Hz), 5.71 (d, 1H, H-1', $J_{1',2'}$ = 3.1 Hz), 5.16 (s, 1H, H-1''), 4.56 (d, 1H, H-5', $J_{5',4'}$ = 4.9 Hz), 4.46 (dd, 1H, Lys-α-C*H*, *J* = 8.8, *J* = 5.6 Hz), 4.35-4.29 (m, 2H, H-2', ArC*H*2), 4.25 (d, 1H, ArC*H*2, J_{gem} = 13.0 Hz), 4.21 (dd, 1H, H-3', $J_{3',4'}$ = 7.2, $J_{3',2'}$ = 5.8 Hz), 4.16 (dd, 1H, H-4', $J_{4',3'}$ = 7.2, $J_{4',5'}$ = 4.9 Hz), 4.10-3.99 (m, 3H, H-2'', H-3'', H-4''), 3.95 (br s, 1H, H-6'), 3.24-3.16 (m, 2H, H-5''), 2.94 (t, 2H, Lys-ε-C*H*2, *J* = 7.6 Hz), 2.28 (t, 2H, acyl-α-C*H*2, *J* = 7.6 Hz), 1.95-1.85 (m, 1H, Lys-β-C*H*2), 1.82-1.42 (m, 7H, Lys-β-C*H*2, Lys-γ-C*H*2, Lys-δ-C*H*2, acyl-β-C*H*2), 1.38-1.24 (m, 24H, acyl-C*H*2×12), 0.90 (t, 3H, acyl-C*H*3, *J* = 6.7 Hz); ^{13}C NMR (CD_3OD, 100 MHz) δ 176.6, 172.8, 171.1, 165.8, 162.4 (q, $^2J_{C,F}$ = 37.6 Hz), 152.1, 143.7, 141.0, 132.1, 127.6, 121.5, 110.3, 103.2, 94.4, 86.2, 80.4, 77.5, 76.3, 73.8, 73.8, 71.1, 63.8, 55.2, 52.4, 43.9, 40.5, 36.8, 33.1, 32.6, 30.8, 30.7, 30.6, 30.5, 30.4, 28.2, 26.9, 23.9, 23.7, 14.4; ESIMS-HR *m/z*: [M+H]$^+$ calcd. for $C_{45}H_{74}N_7O_{13}$ 920.5339, found 920.5359; $[\alpha]^{21}_D$ 0.52 (*c* 0.66, MeOH).

Palmitoyl-*m*Lys-anilide (**6**)

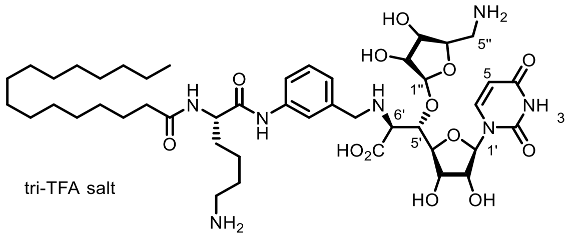

According to general procedure F, **S20** (22.4 mg, 17.4 μmol) was used to afford **6** (17.7 mg, 81%) as a white solid.

^{1}H NMR (CD_3OD, 400 MHz, containing conformers, attributed the main peak) δ 7.86 (s, 1H, Ar), 7.66 (d, 1H, H-6, $J_{6,5}$ = 8.1 Hz), 7.47 (br d, 1H, Ar, *J* = 8.1 Hz), 7.39 (t, 1H, Ar, *J* = 7.9 Hz), 7.20 (br d, 1H, Ar, *J* = 7.6 Hz), 5.77 (d, 1H, H-1', $J_{1',2'}$ = 2.7 Hz), 5.72 (d, 1H, H-5, $J_{5,6}$ = 8.1 Hz), 5.16 (s, 1H, H-1''), 4.57 (br d, 1H, H-5', *J* = 4.5 Hz), 4.47 (dd, 1H, Lys-α-C*H*, *J* = 9.0, *J* = 5.4 Hz), 4.39 (d, 1H, ArC*H*2, J_{gem} = 13.0 Hz), 4.30-4.19 (m, 3H, H-2', H-3', ArC*H*2), 4.15 (dd, 1H, H-4', $J_{4',3'}$ = 7.2, $J_{4',5'}$ = 4.5 Hz), 4.12-4.01 (m, 3H, H-2'', H-3'', H-4''), 3.95-3.92 (m, 1H, H-6'), 3.26-3.13 (m, 2H, H-5''), 2.94 (t, 2H, Lys-ε-C*H*2, *J* = 7.0 Hz), 2.31-2.27 (m, 2H, acyl-α-C*H*2), 1.97-1.86 (m, 1H, Lys-β-C*H*2), 1.84-1.68 (m, 3H, Lys-β-C*H*2, Lys-δ-C*H*2), 1.68-1.58 (m, 2H, acyl-β-C*H*2), 1.58-1.42 (m, 2H, Lys-γ-C*H*2), 1.39-1.24 (m, 24H, acyl-C*H*2×12), 0.90 (t, 3H, acyl-C*H*3, *J* = 6.7 Hz); ^{13}C NMR (CD_3OD, 100 MHz) δ 176.6, 172.9, 171.0, 165.9, 162.2 (q, $^2J_{C,F}$ = 35.6 Hz), 152.1, 143.5, 140.3, 132.9, 130.8, 127.1, 122.9, 122.5, 117.8 (q, $^1J_{C,F}$ = 294.8 Hz), 110.5, 103.2, 94.3, 86.2, 80.5, 77.3, 76.4, 74.0, 73.7, 71.0, 64.2, 55.2, 52.7, 43.9, 40.5, 36.8, 33.1, 32.6, 30.8, 30.7, 30.6, 30.5, 30.4, 28.2, 26.9, 24.0, 23.7, 14.4;ESIMS-HR *m/z*: [M+H]$^+$ calcd. for $C_{45}H_{73}N_7O_{13}$ 920.5339, found 920.5359; $[\alpha]^{22}_D$ -4.84 (*c* 0.28, MeOH).

Palmitoyl-*p*Arg-anilide (**7**)

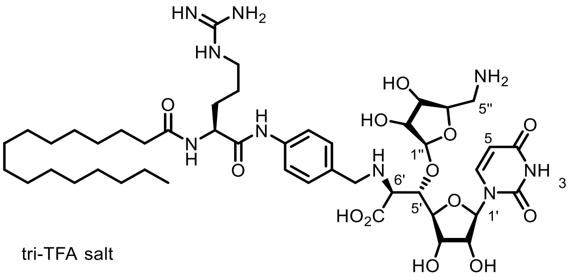

According to general procedure F, **S21** (23.1 mg, 15.8 μmol) was used to afford **7** (14.9 mg, 73%) as a white solid.

^{1}H NMR (CD_3OD, 400 MHz) δ 7.65 (d, 2H, Ar, J = 8.5 Hz), 7.62 (d, 1H, H-6, $J_{6,5}$ = 8.1 Hz), 7.42 (d, 2H, Ar, J = 8.5 Hz), 5.73 (d, 1H, H-5, $J_{5,6}$ = 8.1 Hz), 5.71 (d, 1H, H-1', $J_{1',2'}$ = 4.0 Hz), 5.16 (s, 1H, H-1''), 4.56 (br d, 1H, H-5', J = 4.5 Hz), 4.49 (dd, 1H, Arg-α-C*H*, J = 8.3, J = 5.6 Hz), 4.35-4.30 (m, 2H, H-2', ArCH_2), 4.25 (d, 1H, ArCH_2, J_{gem} = 13.0 Hz), 4.21 (dd, 1H, H-3', $J_{3',4'}$ = 7.2, $J_{3',2'}$ = 5.8 Hz), 4.16 (dd, 1H, H-4', $J_{4',3'}$ = 7.2, $J_{4',5'}$ = 4.9 Hz), 4.09-4.00 (m, 3H, H-2'', H-3'', H-4''), 3.95 (br s, 1H, H-6'), 3.26-3.18 (m, 4H, H-5'', Arg-δ-CH_2), 2.28 (t, 2H, acyl-α-CH_2, J = 7.6 Hz), 1.96-1.87 (m, 1H, Arg-β-CH_2), 1.82-1.59 (m, 5H, Arg-β-CH_2, Arg-γ-CH_2, acyl-β-CH_2), 1.36-1.24 (m, 24H, acyl-CH_2×12), 0.90 (t, 3H, acyl-CH_3, J = 6.7 Hz); ^{13}C NMR (CD_3OD, 100 MHz) δ 176.6, 172.6, 171.1, 165.9, 162.4 (q, $^2J_{C,F}$ = 37.6 Hz), 158.6, 152.1, 143.7, 140.9, 132.2, 127.6, 121.5, 117.8 (q, $^1J_{C,F}$ = 291.9 Hz), 110.3, 103.2, 94.4, 86.2, 80.4, 77.5, 76.3, 73.8, 73.7, 71.1, 63.8, 55.0, 52.4, 43.9, 42.0, 36.8, 33.1, 30.8, 30.6, 30.5, 30.4, 28.3, 26.9, 26.4, 23.7, 14.4; ESIMS-HR *m*/*z*: $[M+H]^+$ calcd. for $C_{45}H_{74}N_9O_{13}$ 948.5401, found 948.5388; $[\alpha]^{21}_D$ -0.43 (*c* 0.69, MeOH).

Palmitoyl-*m*Arg-anilide (**8**)

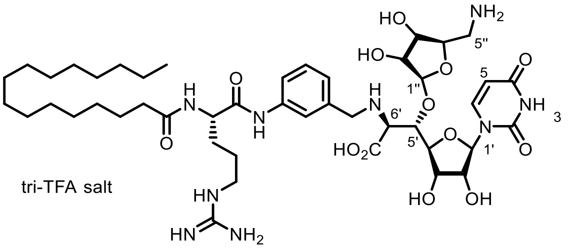

According to general procedure F, **S22** (22.1 mg, 15.1 μmol) was used to afford **8** (13.5 mg, 69%) as a white solid.

^{1}H NMR (CD_3OD, 400 MHz, containing conformers, attributed the main peak) δ 7.88 (br s, 1H, Ar), 7.66 (d, 1H, H-6, $J_{6,5}$ = 8.1 Hz), 7.46 (br d, 1H, Ar, J = 9.0 Hz), 7.39 (t, 1H, Ar, J = 7.6 Hz), 7.20 (br d, 1H, Ar, J = 7.6 Hz), 5.76 (d, 1H, H-1', $J_{1',2'}$ = 2.7 Hz), 5.72 (d, 1H, H-5, $J_{5,6}$ = 8.1 Hz), 5.16 (s, 1H, H-1''), 4.58 (br d, 1H, H-5', J = 4.5 Hz), 4.50 (dd, 1H, Arg-α-C*H*, J = 8.1, J = 5.8 Hz), 4.40 (d, 1H, ArCH_2, J_{gem} = 13.0 Hz), 4.31-4.20 (m, 3H, H-2', H-3', ArCH_2), 4.15 (dd, 1H, H-4', $J_{4',3'}$ = 7.6, $J_{4',5'}$ = 4.5 Hz), 4.12-4.00 (m, 4H, H-6', H-2'', H-3'', H-4''), 3.28-3.19 (m, 4H, H-5'', Arg-δ-CH_2), 2.33-2.28 (m, 2H, acyl-α-CH_2), 1.98-1.87 (m, 1H, Arg-β-CH_2), 1.85-1.58 (m, 5H, Arg-β-CH_2, Arg-δ-CH_2, acyl-β-CH_2), 1.38-1.24 (m, 24H, acyl-CH_2×12), 0.90 (t, 3H, acyl-CH_3, J = 7.0 Hz); ^{13}C NMR (CD_3OD, 100 MHz) δ 176.7, 172.6, 171.0, 165.9, 162.2 (q, $^2J_{C,F}$ = 36.6 Hz), 158.6, 152.1, 143.4, 140.3, 132.9, 130.8, 127.1, 122.9, 122.5, 117.7 (q, $^1J_{C,F}$ = 290.0 Hz), 110.5, 103.2, 94.3, 86.2, 80.5, 77.2, 76.4, 74.1, 73.7, 71.0, 64.3, 55.0, 52.7, 43.9, 42.0, 36.8, 33.1, 30.8, 30.8, 30.6, 30.5, 30.4, 26.9, 26.5, 23.7, 14.4; ESIMS-HR *m*/*z*: $[M+H]^+$ calcd. for $C_{45}H_{74}N_9O_{13}$ 948.5401, found 948.5388; $[\alpha]^{22}_D$ -5.84 (*c* 0.24, MeOH).

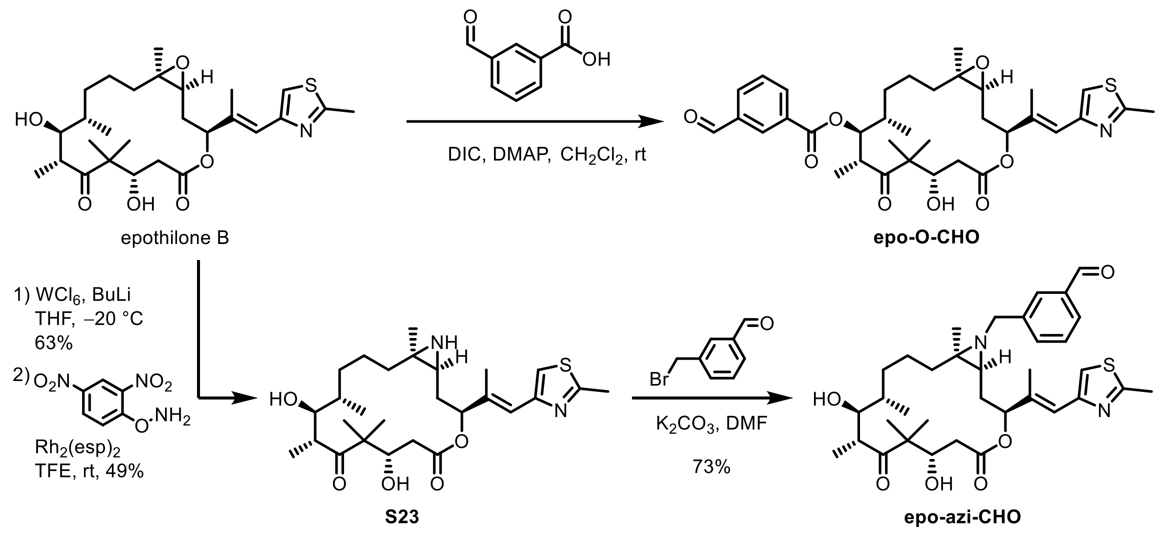

Scheme S7. Synthesis of **epo-O-CHO** and **epo-azi-CHO**.

7-*O*-(3-formylbenzoyl)-epothilone B (**epo-O-CHO**)

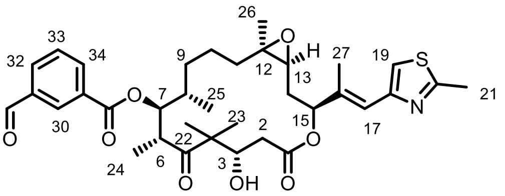

A solution of epothilone B (29.4 mg, 57.9 µmol, 1.0 equiv.) in CH_2Cl_2 (1 mL) was sequentially treated with DMAP (1.4 mg, 11.6 µmol, 0.2 equiv.), 3-formylbenzoic acid (8.7 mg, 57.9 µmol, 1.0 equiv.), and DIC (13.4 µL, 86.9 µmol, 1.5 equiv.) at room temperature for 25 h. The reaction mixture was concentrated *in vacuo*, and the residue was purified by preparative TLC (4% MeOH/$CHCl_3$) to afford the desired 7-ester **epo-O-CHO** (11.5 mg, 31%) as a white solid, diester (2.8 mg, 6%) as a white solid, 3-ester (2.4 mg, 6%) as a white solid, and recovered epothilone B (15.8 mg, 54%) as a white solid.

^{1}H NMR (CD_3OD, 400 MHz) δ 10.10 (s, 1H, C*H*O), 8.54 (dd, 1H, H-30, $J_{30,32} = J_{30,34} = 1.4$ Hz), 8.33 (ddd, 1H, H-32, $J_{32,33} = 7.6$, $J_{32,30} = J_{32,34} = 1.4$ Hz), 8.10 (ddd, 1H, H-34, $J_{34,33} = 7.6$, $J_{34,30} = J_{34,32} = 1.4$ Hz), 7.66 (dd, 1H, H-33, $J_{33,32} = J_{33,34} = 7.6$ Hz), 7.01 (s, 1H, H-19), 6.66 (br s, 1H, H-17), 5.64 (dd, 1H, H-7, $J_{7,6} = 8.8$, $J_{7,8} = 1.8$ Hz), 5.52 (dd, 1H, H-15, $J_{15,14} = J_{15,14} = 4.5$ Hz), 4.35 (br d, 1H, 3-OH, $J = 6.3$ Hz), 4.17-4.12 (m, 1H, H-3), 3.66 (dq, 1H, H-6, $J_{6,7} = 8.8$, $J_{6,24} = 6.7$ Hz), 2.89 (dd, 1H, H-13, $J_{13,14} = J_{13,14} = 6.3$ Hz), 2.71 (s, 3H, H-21), 2.62 (dd, 1H, H-2, $J_{gem} = 13.9$, $J_{2,3} = 9.9$ Hz), 2.54 (dd, 1H, H-2, $J_{gem} = 13.9$, $J_{2,3} = 4.0$ Hz), 2.14 (s, 3H, H-27), 2.10-1.96 (m, 2H, H-14), 1.87-1.46 (m, 5H, H-8, H-10, H-11), 1.40 (s, 3H, H-22 or 23), 1.31 (s, 3H, H-22 or 23), 1.31-1.22 (m, 2H, H-9), 1.13 (d, 3H, H-24, $J_{24,6} = 6.7$ Hz), 1.11 (s, 3H, H-26), 0.99 (d, 3H, H-25, $J_{25,8} = 6.7$ Hz); ^{13}C NMR (CD_3OD, 100 MHz) δ 217.0, 191.6, 170.8, 165.2, 165.1, 152.2, 136.7, 136.5, 135.5, 133.4, 131.6, 131.3, 129.5, 119.8, 116.5, 80.1, 76.0, 73.9, 60.9, 60.8, 52.5, 44.0, 38.7, 35.2, 31.6, 31.0, 29.6, 23.3, 23.1, 22.1, 20.2, 19.3, 180, 16.1, 15.8; ESIMS-HR *m*/*z*: $[M+H]^+$ calcd. for $C_{35}H_{46}NO_8S$ 640.2939, found 640.2941; $[\alpha]^{20}_D$ +12.30 (*c* 0.26, $CHCl_3$).

N-(3-formylbenzyl)-12,13-aziridinyl-epothilone B (**epo-azi-CHO**)

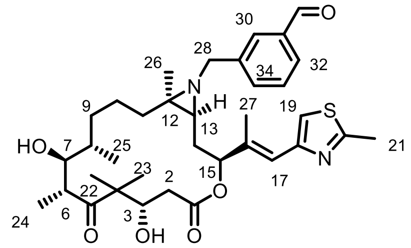

A solution of 12,13-aziridinyl-epothilone synthesized from epothilone B (**S23**)[11,12] (6.3 mg, 14.3 μmol, 1.0 equiv.) in DMF (0.5 mL) was treated with 3-bromomethylbenzaldehyde (14.2 mg, 71.5 μmol, 5.0 equiv.) and K_2CO_3 (11.9 mg, 85.8 μmol, 6 equiv.) at room temperature, and the reaction mixture was heated to 50 °C. After stirring for 20 min, the reaction mixture was cooled and quenched with *sat. aq.* NH_4Cl (1.5 mL). The resulting mixture was extracted with EtOAc. The organic layer was washed with brine, dried (Na_2SO_4), filtered and concentrated *in vacuo* (toluene co-evap×3). The residue was purified by flash silica gel column chromatography (ϕ0.8×6 cm; MeOH/$CHCl_3$ = 0-1-2-5%) to afford **epo-azi-CHO** (5.2 mg, 8.32 μmol, 73%) as a pale yellow solid.

^{1}H NMR (CD_3OD, 400 MHz) δ 10.01 (s, 1H, C*H*O), 7.88 (s, 1H, H-30), 7.76 (d, 1H, H-32, $J_{32,33}$ = 7.7 Hz), 7.62 (d, 1H, H-34, $J_{34,33}$ = 7.7 Hz), 7.49 (dd, 1H, H-33, $J_{33,32}$ = $J_{33,34}$ = 7.7 Hz), 6.96 (s, 1H, H-19), 6.55 (s, 1H, H-17), 5.36 (dd, 1H, H-15, $J_{15,14}$ = 8.2, $J_{15,14}$ = 3.6 Hz), 4.34-4.28 (br d, 1H, OH-3, J = 5.0 Hz), 4.16 (m, 1H, H-3), 3.81-3.76 (m, 1H, H-7), 3.81 (d, 1H, H-28, J_{gem} = 14.5 Hz), 3.68 (d, 1H, H-28, J_{gem} = 14.5 Hz), 3.22 (qd, 1H, H-6, $J_{6,24}$ = 6.9, $J_{6,7}$ = 4.0 Hz), 2.70 (s, 3H, H-21), 2.65 (br s, 1H, OH-7), 2.49 (dd, 1H, H-2, J_{gem} = 14.2, $J_{2,3}$ = 9.5 Hz), 2.38 (dd, 1H, H-2, J_{gem} = 14.2, $J_{2,3}$ = 2.9 Hz), 2.09-2.02 (m, 1H, H-14), 2.05 (d, 3H, H-27, J = 0.9 Hz), 1.81 (ddd, 1H, H-14, J_{gem} = 15.0, $J_{14,15}$ = $J_{14,13}$ = 8.6 Hz), 1.76-1.68 (m, 1H, H-8), 1.68-1.39 (m, 5H, H-10, H-11, H-13), 1.38-1.23 (m, 2H, H-9), 1.33 (s, 3H, H-22 or 23 or 26), 1.22 (s, 3H, H-22 or 23 or 26), 1.14 (d, 3H, H-24, $J_{24,6}$ = 6.9 Hz), 1.10 (s, 3H, H-22 or 23 or 26), 0.96 (d, 3H, H-25, $J_{25,8}$ = 7.3 Hz); ^{13}C NMR (CD_3OD, 100 MHz) δ 221.1, 192.6, 171.1, 165.3, 151.9, 141.5, 138.1, 136.6, 134.2, 129.1, 128.5, 120.0, 116.2, 78.7, 73.4, 56.1, 53.0, 48.9, 44.8, 42.8, 39.6, 36.4, 35.9, 32.8, 31.1, 21.8, 21.7, 19.8, 19.2, 17.3, 16.1, 15.6, 13.1; ESIMS-HR *m/z*: $[M+H]^+$ calcd. for $C_{35}H_{49}N_2O_6S$ 625.3306, found 625.3311; $[\alpha]^{20}_D$ -48.27 (*c* 0.26, $CHCl_3$).

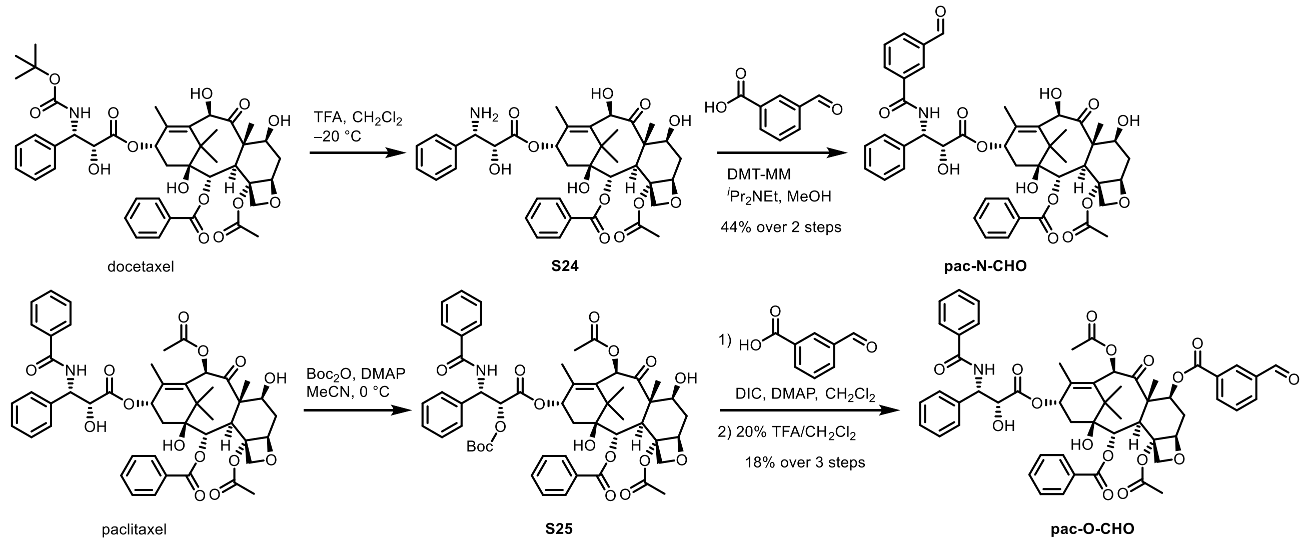

Scheme S8. Synthesis of **pac-O-CHO** and **pac-N-CHO**.

10-deacetyl-3′′′-formylpaclitaxel (**pac-N-CHO**)

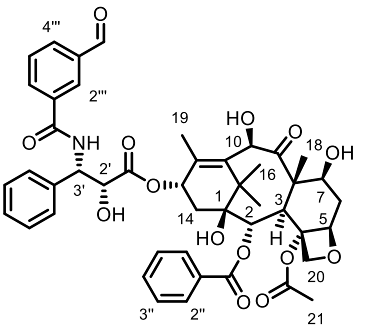

A solution of docetaxel (300 mg, 371 μmol) in CH_2Cl_2 (3 mL) was treated with TFA (750 μL) at −10 °C for 4 h. The reaction was quenched with *sat. aq.* $NaHCO_3$, and the whole mixture was partitioned between CH_2Cl_2/MeOH (9/1) and *sat. aq.* $NaHCO_3$. The organic phase was washed with *sat. aq.* $NaHCO_3$, dried (Na_2SO_4), filtered, and concentrated *in vacuo*. The residue was purified by silica gel column chromatography (φ 2.6 × 10.0 cm, 0-4-10% MeOH/$CHCl_3$) to afford **S24** (229 mg, 324 μmol, 87%) as a white solid. A solution of **S24** (30.0 mg, 42.4 μmol), *m*-formylbenzoic acid (12.7 mg, 84.8 μmol) and ^{i}Pr_2NEt (14.3 μL, 84.8 μmol) in MeOH (2 mL) was treated with DMT-MM (23.5 mg, 84.8 μmol) at room temperature for 1.5 h. The mixture was concentrated *in vacuo* and the residue was cludely purified by silica gel column chromatography (φ 2.0 × 6.5 cm, 0-1-2-5% MeOH/$CHCl_3$). The clude compound was partitioned between CH_2Cl_2/MeOH (9/1) and 1 M *aq*. HCl. The organic phase was washed with 1 M *aq*. HCl, *sat. aq*. $NaHCO_3$, dried (Na_2SO_4), filtered, and concentrated *in vacuo*. The residue was purified by silica gel column chromatography (φ 1.6 × 6.0 cm, 0-1-2-5% MeOH/$CHCl_3$) to afford **pac-N-CHO** (18.3 mg, 21.8 μmol, 51%) as a white solid.

^{1}H NMR ($CDCl_3$, 400 MHz) δ 9.92 (s, 1H, C*H*O), 8.23 (s, 1H, H-2'''), 8.12 (d, 2H, H-2'', H-6'', $J_{2'',3''}$ = 7.6 Hz, $J_{6'',5''}$ = 7.6 Hz), 8.04 (d, 1H, H-4''', $J_{4''',5'''}$ = 7.6 Hz), 7.97 (d, 1H, H-6''', $J_{6''',5'''}$ = 7.6 Hz), 7.61 (t, 1H, H-4'', $J_{4'',3''}$ = $J_{4'',5''}$ = 7.6 Hz), 7.56 (dd, 1H, H-5''', $J_{5''',4'''}$ = $J_{5''',6'''}$ = 7.6 Hz), 7.50 (t, 2H, H-3'', H-5'', $J_{3'',2''}$ = $J_{3'',4''}$ = 7.6 Hz, $J_{5'',4''}$ = $J_{5'',6''}$ = 7.6 Hz), 7.49-7.31 (m, 6H, Ar-*H* of C3' phenyl, N*H*-3'), 6.19 (t, 1H, H-13, $J_{13,14}$ = 8.5 Hz), 5.79 (dd, 1H, H-3', $J_{3',NH\text{-}3'}$ = 8.8 Hz, $J_{3',2'}$ = 2.5 Hz), 5.65 (d, 1H, H-2, $J_{2,3}$ = 7.0 Hz), 5.20 (s, 1H, H-10), 4.92 (d, 1H, H-5, $J_{5,6}$ = 8.5 Hz), 4.80 (s, 1H, H-2'), 4.33-4.17 (m, 4H, H-7, H-20, O*H*-10), 4.03 (br s, 1H, O*H*-2'), 3.87 (d, 1H, H-3, $J_{3,2}$ = 7.0 Hz), 2.58-2.50 (m, 1H, H-6), 2.37 (s, 3H, H-21), 2.31-2.18 (m, 2H, H-14), 1.95-1.73 (m, 7H, H-6, H-18, H-19), 1.17 (s, 3H, H-16), 1.09 (s, 3H, H-17); ^{13}C NMR ($CDCl_3$, 100 MHz) δ 213.3, 193.9, 174.3, 172.7, 169.1, 168.0, 140.3, 139.9, 138.6, 138.2, 136.8, 136.0, 135.3, 135.1, 132.4, 131.7, 131.3, 131.2, 130.9, 130.6, 130.1, 129.2, 86.4, 83.3, 80.9, 79.4, 76.9, 76.6, 75.3, 74.4, 74.1, 59.8, 57.4, 48.6, 45.2, 39.1, 37.9, 28.7, 24.7, 22.7, 16.6, 12.0; ESIMS-HR calcd. for $C_{46}H_{50}NO_{14}$ 840.3226, found 840.3218; $[\alpha]^{21}_D$ −26.25 (*c* 0.44, $CHCl_3$).

7-(3-formylbenzoyl)paclitaxel (**pac-O-CHO**)

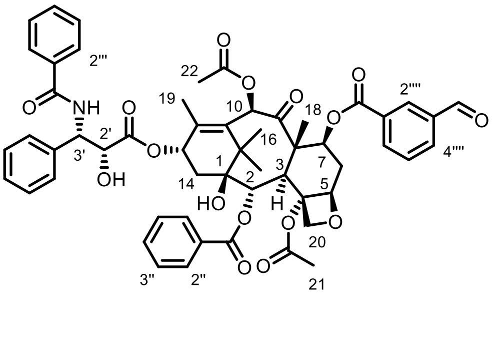

A solution of paclitaxel (34.2 mg, 40.1 μmol), Boc_2O (8.7 mg, 40.1 μmol) in MeCN (400 μL) was treated with DMAP (2.0 mg, 16.0 μmol) at 0 °C for 1 h. Trimethylsilanol (89.0 μL, 797 μmol) was added to the reaction mixture, which was further stirred for 1 h. When the reaction was not completed after 1 h, Boc_2O was added as needed. After completion of the reaction, the mixture was concentrated *in vacuo*, and the residue was partitioned between AcOEt and 0.1 M *aq*. HCl. The organic phase was washed with 1 M *aq*. HCl, *sat. aq*. $NaHCO_3$ and brine, dried (Na_2SO_4), filtered, and concentrated *in vacuo* to afford Boc-paclitaxel **S25** (37.2 mg, 39.0 μmol, 98%) as a white solid. A solution of Boc-paclitaxel **S25** (34.1 mg, 35.7 μmol), *m*-formylbenzoic acid (21.4 mg, 143 μmol)

and DMAP (10.6 mg, 86.9 μmol) in DMF (1.5 mL) was treated with DIC (22.0 μL, 143 μmol) at room temperature for 2 d. The mixture was partitioned between AcOEt and 1 M *aq*. HCl. The organic phase was washed with 1 M *aq*. HCl, *sat. aq*. $NaHCO_3$ and brine, dried (Na_2SO_4), filtered, and concentrated *in vacuo*. A solution of the residue, *m*-formylbenzoic acid (214 mg, 1.43 mmol) and DMAP (50.9 mg, 417 μmol) in DMF (1.5 mL) was treated with DIC (220 μL, 1.43 mmol) at room temperature for 1 d. The mixture was partitioned between AcOEt and 1 M *aq*. HCl. The organic phase was washed with 1 M *aq*. HCl, *sat. aq*. $NaHCO_3$ and brine, dried (Na_2SO_4), filtered, and concentrated *in vacuo*. The residue was purified by silica gel column chromatography (φ 2.0 × 6.5 cm, 30-40% AcOEt/Hexane), and the purified compound was treated with 20% TFA/CH_2Cl_2 (1 mL) at 0 °C for 3 h. The mixture was partitioned between AcOEt and *sat. aq*. $NaHCO_3$. The organic phase was washed with *sat. aq*. $NaHCO_3$ and brine, dried (Na_2SO_4), filtered, and concentrated *in vacuo*. The residue was purified by silica gel column chromatography (φ 2.0 × 6.5 cm, 45-55% AcOEt/Hexane) to afford **pac-O-CHO** (7.3 mg, 7.4 μmol, 21%) as a white solid.

^{1}H NMR ($CDCl_3$, 400 MHz) δ 10.08 (s, 1H, C*H*O), 8.39 (s, 1H, H-2''''), 8.17-8.13 (m, 3H, H-2'', H-6'', H-4'''), 8.09 (d, 1H, H-4'''', $J_{4'''', 5''''}$ = 7.6 Hz), 7.77 (d, 2H, H-2''', H-6''', $J_{2''', 3'''}$ = 7.6 Hz, $J_{6''', 5'''}$ = 7.6 Hz), 7.66-7.34 (m, 12H, H-5'''', H-6'''', H-3'', H-5'', H-4'', H-3''', H-5''', Ar-*H* of C3' phenyl), 7.07 (d, 1H, N*H*-3', $J_{NH\text{-}3', 3'}$ = 8.5 Hz), 6.34 (s, 1H, H-10), 6.20 (t, 1H, H-13, $J_{13, 14}$ = 8.5 Hz), 5.83-5.74 (m, 3H, H-3', H-2, H-7), 5.00 (d, 1H, H-5, $J_{5, 6}$ = 9.0 Hz), 4.80 (s, 1H, H-2'), 4.37 (d, 1H, H-20), 4.25 (d, 1H, H-20), 4.03 (d, 1H, H-3, $J_{3, 2}$ = 6.7 Hz), 3.63 (br s, 1H, O*H*-2'), 2.84-2.76 (m, 1H, H-6), 2.42 (s, 3H, H-21), 2.36 (d, 2H, H-14, $J_{14, 13}$ = 8.5 Hz), 2.03-1.96 (m, 7H, H-6, H-18, H-19), 1.87 (s, 3H, H-22), 1.21 (s, 3H, H-16), 1.19 (s, 3H, H-17); ^{13}C NMR ($CDCl_3$, 125 MHz) δ 204.5, 193.7, 174.7, 172.6, 170.7, 169.2, 169.1, 166.5, 142.7, 140.1, 138.5, 137.6, 136.0, 135.8, 135.3, 134.5, 134.4, 134.1, 133.4, 132.3, 131.2, 130.9, 130.9, 130.5, 129.2, 129.2, 86.0, 83.1, 80.8, 78.6, 77.1, 76.5, 75.3, 74.8, 74.3, 58.5, 57.1, 48.9, 45.4, 37.8, 35.6, 31.8, 29.8, 28.6, 24.7, 23.1, 22.6, 16.8, 13.2; ESIMS-HR calcd. for $C_{55}H_{56}NO_{16}$ 986.3594, found 986.3609; $[\alpha]^{20}_D$ −18.31 (*c* 0.73, $CHCl_3$).

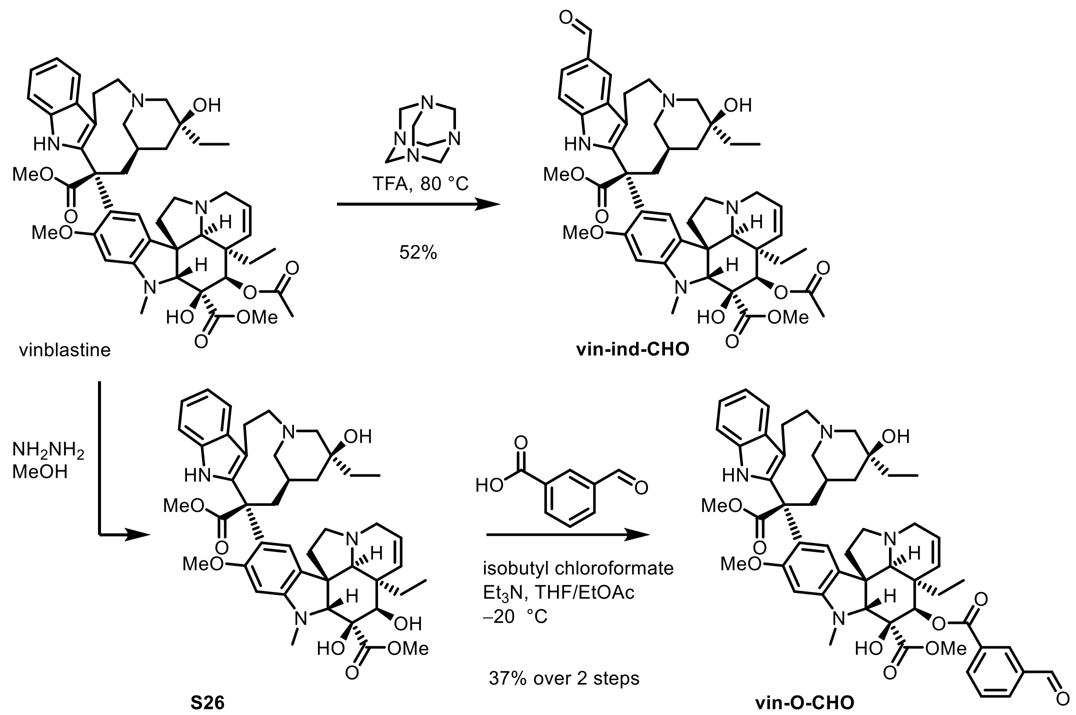

Scheme S9. Synthesis of **vin-ind-CHO** and **vin-O-CHO**.

12′-formylvinblastine bis-trifluoroacetate (**vin-ind-CHO**)

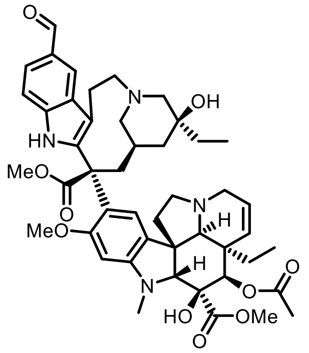

Vinblastine sulfate (20.0 mg, 22.0 μmol, 1.0 equiv.) and hexamethylenetetramine (37.0 mg, 264 μmol, 12 equiv.) were dissolved in TFA (4 mL) at room temperature, and the mixture was heated to 75 °C. After stirring for 20 min, the reaction mixture was cooled to room temperature and concentrated *in vacuo*. The residue was dissolved in *sat. aq.* $NaHCO_3$ (2 mL). The mixture was neutralized with solid $NaHCO_3$ and extracted with CH_2Cl_2×3. The combined organic layer was washed with brine, dried (Na_2SO_4), filtered and concentrated *in vacuo*. The residue was purified by flash silica gel column chromatography (ϕ1.1×8 cm; MeOH/$CHCl_3$ = 0-2-4%) and Sep-Pak ODS (MeCN/H_2O = 10-30-50-70%, containing 0.1% TFA) to afford **vin-ind-CHO** bis-trifluoroacetate (12.3 mg, 11.5 μmol, 52%) as a white solid. This is a known compounds in WO 2005/055939[15].

^{1}H NMR (CD_3OD, 400 MHz) δ 9.72 (br s, 1H), 7.59 (s, 1H), 7.29 (d, 1H, *J* = 8.6 Hz), 7.21 (dd, 1H, *J* = 8.6, *J* = 1.4 Hz), 6.73 (s, 1H), 6.43 (s, 1H), 5.94 (dd, 1H, *J* = 10.2, *J* = 4.3 Hz), 5.67 (br d, 1H, *J* = 10.4 Hz), 5.44 (s, 1H), 5.36 (s, 1H), 4.70-4.60 (m, 2H), 3.99-3.90 (m, 3H), 3.88-3.86 (m, 1H), 3.87 (s, 3H), 3.82 (s, 3H), 3.79 (s, 1H), 3.77-3.58 (m, 5H), 3.69 (s, 3H), 3.49 (br d, 1H, *J* = 15.4 Hz), 3.37 (dd, 1H, *J* = 10.6, *J* = 6.3 Hz), 3.27-3.18 (m, 3H), 2.90 (dd, 1H, *J* = 8.2, *J* = 6.1 Hz), 2.78 (S, 3H), 2.47 (dd, 1H, *J* = 16.1, *J* = 4.3 Hz), 2.38-2.31 (m, 1H), 2.08 (s, 3H), 2.06-1.99 (m, 1H), 1.78-1.71 (m, 1H), 1.67 (m, 2H), 1.61-1.49 (m, 3H), 1.43-1.35 (br s, 1H), 0.98 (t, 3H, *J* = 7.5 Hz), 0.81 (t, 3H, *J* = 7.3 Hz); ^{13}C NMR (CD_3OD, 100 MHz) δ 176.1, 172.1, 171.7, 161.9, 161.5, 160.5, 154.3, 137.5, 137.4, 133.1, 132.8, 130.7, 124.8, 122.6, 122.2, 122.2, 121.1, 118.9, 117.2, 116.0, 115.1, 112.4, 105.7, 101.3, 95.6, 81.4, 81.2, 76.0, 68.3, 67.4, 62.2, 58.1, 56.8, 56.6, 53.9, 53.5, 53.0, 50.6, 50.0, 46.4, 44.4, 44.2, 38.5, 37.7, 36.5, 35.5, 32.0, 27.9, 21.9,

20.8, 8.3, 7.0; ESIMS-HR *m/z*: $[M+H]^+$ calcd. for $C_{47}H_{59}N_4O_{10}$ 839.4226, found 839.4230; $[\alpha]^{21}_{D}$ -33.11 (*c* 0.18, MeOH).

Deacetyl-(3-formylbenzoyl)vinblastine (**vin-O-CHO**)

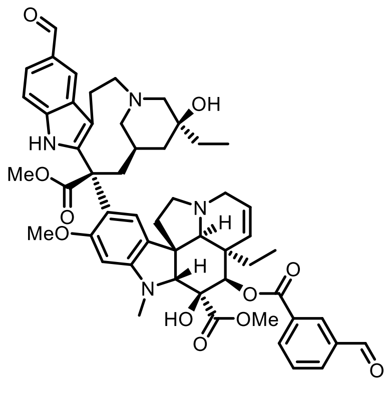

A solution of vinblastine sulfate (36.4 mg, 40.0 μmol) in MeOH (2 mL) was treated with $NH_2NH_2 \cdot H_2O$ (920 μL, 18.9 mmol) at room temperature for 1 d. The mixture was partitioned between CH_2Cl_2 and *sat. aq*. $NaHCO_3$. The organic phase was washed with *sat. aq*. $NaHCO_3$, H_2O and brine, dried (Na_2SO_4), filtered, and concentrated *in vacuo*. A solution of the crude compound **S26**, *m*-formylbenzoic acid (46.2 mg, 308 μmol) and NEt_3 (43.0 μL, 308 μmol) in THF/AcOEt (7:3) (2.5 mL) was treated with isobutyl chloroformate (40.5 μL, 308 μmol) at −20 °C for 20 min. The mixture was warmed to room temperature for 17 h. When the reaction was not completed after 17 h, *m*-formylbenzoic acid, NEt_3 and isobutyl chloroformate were added as needed. After completion of the reaction, the mixture was partitioned between AcOEt and *sat. aq*. $NaHCO_3$. The organic phase was washed with *sat. aq*. $NaHCO_3$, H_2O and brine, dried (Na_2SO_4), filtered, and concentrated *in vacuo*. The residue purified by ODS column chromatography (φ 2.3 × 12.3 cm, 0-20-50% MeCN/H_2O, containing 0.1% TFA) to afford **vin-O-CHO** (16.9 mg, 15.0 μmol, 37%) as a yellow solid.

^{1}H NMR ($CDCl_3$, 400 MHz) δ 12.34 (br s, 1H), 10.47 (br s, 1H), 10.03 (s, 1H, C*H*O), 8.62 (s, 1H, H-2'''), 8.45 (d, 1H, H-4''', $J_{4''',5'''}$ = 7.9 Hz), 8.15 (s, 1H), 8.10 (d, 1H, H-6''', $J_{6''',5'''}$ = 7.9 Hz), 7.63 (dd, 1H, H-5''', $J_{5''',4'''}$ = $J_{5''',6'''}$ = 7.9 Hz), 7.45 (d, 1H, *J* = 8.1 Hz), 7.22-7.09 (m, 3H), 6.58 (s, 1H), 6.19 (s, 1H), 5.85 (dd, 1H, *J* = 10.5 Hz, *J* = 5.2 Hz), 5.77 (s, 1H), 5.44 (d, 1H, *J* = 11.0 Hz), 4.57 (dd, 1H, *J* = 16.8 Hz, *J* = 11.9 Hz), 4.12 (dd, 1H, *J* = 15.5 Hz, *J* = 4.0 Hz), 3.97-3.51 (m, 16H), 3.37-2.82 (m, 7H), 2.73 (s, 1H), 2.60-2.47 (m, 2H), 2.26-2.19 (m, 1H), 2.07-2.01 (m, 1H), 1.80-1.38 (m, 7H), 0.97 (t, 3H, *J* = 7.2 Hz), 0.77 (t, 3H, *J* = 7.2 Hz); ^{13}C NMR ($CDCl_3$, 100 MHz) δ 193.9, 173.1, 167.5, 160.9, 155.2, 138.8, 138.1, 137.2, 135.1, 134.7, 132.6, 132.0, 131.8, 130.6, 125.8, 125.3, 123.7, 123.4, 122.3, 120.2, 119.5, 116.5, 112.5, 99.6, 82.8, 81.3, 78.0, 70.4, 70.1, 63.1, 58.7, 58.1, 57.4, 55.2, 55.1, 54.2, 53.0, 52.0, 47.2, 45.1, 40.2, 39.1, 37.0, 36.4, 33.2, 28.4, 23.1, 9.7, 8.8; ESIMS-LR *m/z* 451.6 $[(M+2H)^{2+}]$; ESIMS-HR calcd. for $C_{51}H_{59}N_4O_{10}$ 901.4382 found 901.4387; $[\alpha]^{20}_{D}$ 16.47 (*c* 0.83, $CHCl_3$).

3-2. Biological evaluations of library of tubulin-binding natural products

Library synthesis of tubulin-binding natural products on the 96 well microplate

10 mM DMSO stock solutions of aldehyde and hydrazine were prepared. Two microlitters of hydrazine solutions were dispensed into 96-well V-bottom plates [3363, Corning], and 2 μL of aldehyde solutions and 16 μL of DMSO were added. After shaking the mixtures for 30 min, DMSO was removed under reduced pressure with a plate centrifuge. When the concentration was completed, 20 μL of DMSO was added to each well and the solids were dissolved by shaking to make 1 mM hydrazone solutions.

Evaluation of microtubule stabilizing/destabilizing effects of tubulin-binding natural products and their analogues

Tubulin polymerization assay was performed according to manufacturer protocol [Cat. # BK011P; Cytoskeleton, Inc]. Test compounds were prepared as DMSO solutions (200x). Tubulin polymerization solutions were prepared on ice [2 mg/mL tubulin in 80 mM PIPES pH 6.9, 2.0 mM $MgCl_2$, 0.5 mM EGTA, 1.0 mM GTP, and 15% glycerol]. One microliter of 200x DMSO solutions were added to 19 μL of deionized water (sterile), and 5 μL of the resulting test solutions (10x) were dispensed into 96 well microplate [flat-black, middle binding, non-binding, #655900, Greiner]. The microplate was warmed at 37 °C (<1 min), and 50 μL of the tubulin solution was added quickly. The fluorescence was detected with TECAN plate reader every 300 seconds [conditions: excitation; 360 nm, emission; 450 nm, gain; 80, number of flashes 25].

Evaluation of cytotoxicity of tubulin-binding natural products and their analogues

HCT-116 cells (ATCC CCL-247) were cultured at 37 °C under 5% CO_2 in air in McCoy's 5A (1x) (Gibco™) supplemented with 10% fetal bovine serum (FBS). Cells were regularly passed to maintain exponential growth. HCT-116 cells were sowed by 1.0×10^4 cells for each well in 96-well plate and cultured at 37 °C under 5% CO_2 in air for 24 h. The cells were treated with a solution of test compounds in DMSO at various concentration and incubated at 37 °C under 5% CO_2 in air for 72 h. After that, the WST-8 (Dojindo) cell proliferation assay was used. Cell viability was indicated as a percentage of the negative control (cells treated with DMSO only) and that value was fixed at 100%.

3-3. ^{1}H, ^{13}C NMR spectrum of compounds

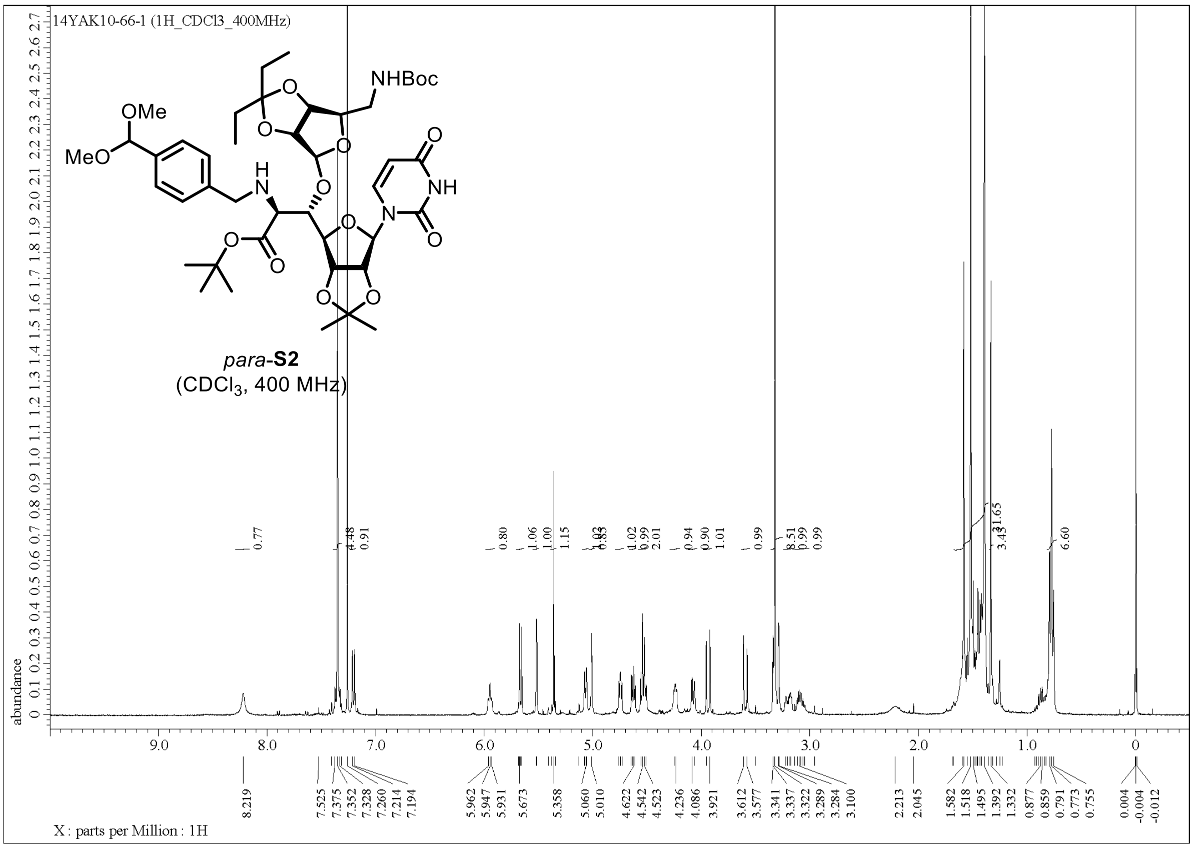

Supplementary Fig. 23. ^{1}H NMR spectrum of compound *para*-**S2**.

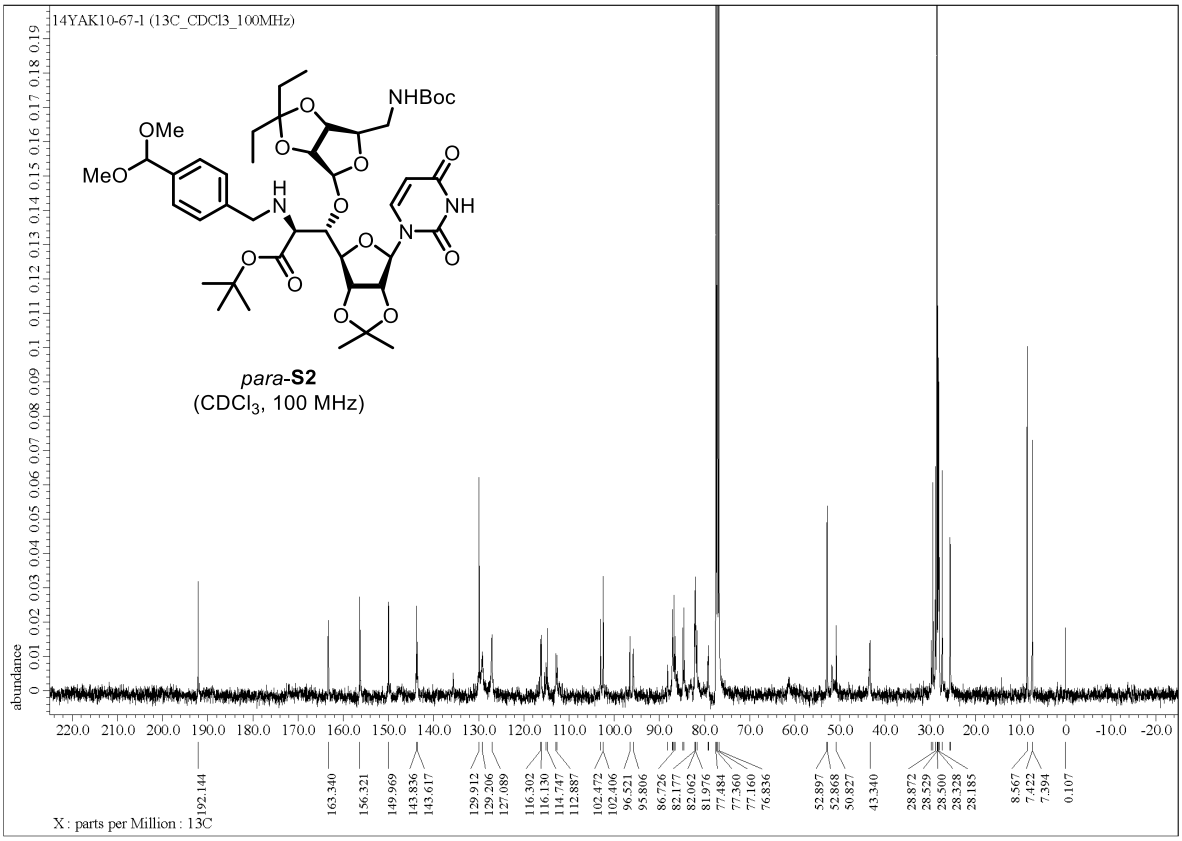

Supplementary Fig. 24. ^{13}C NMR spectrum of compound *para*-**S2**.

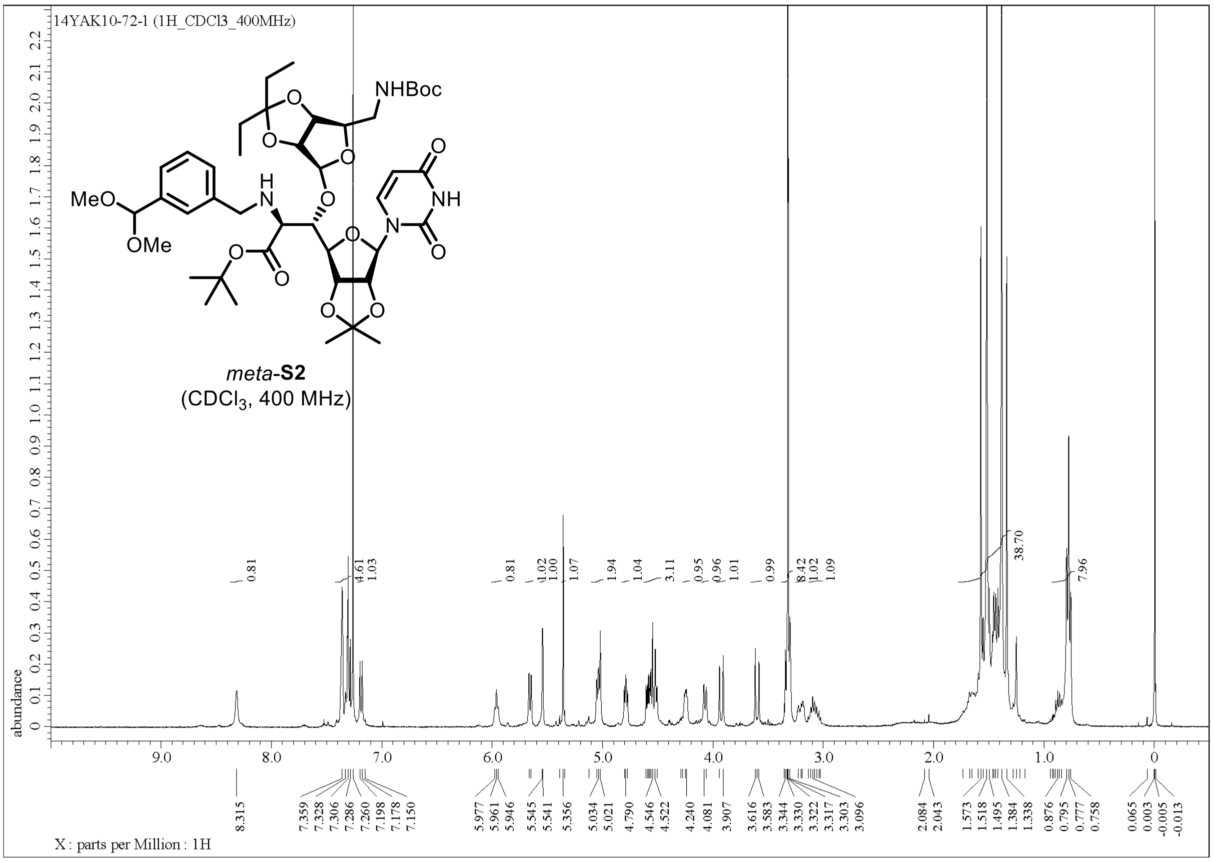

Supplementary Fig. 25. ^{1}H NMR spectrum of compound *meta*-**S2**.

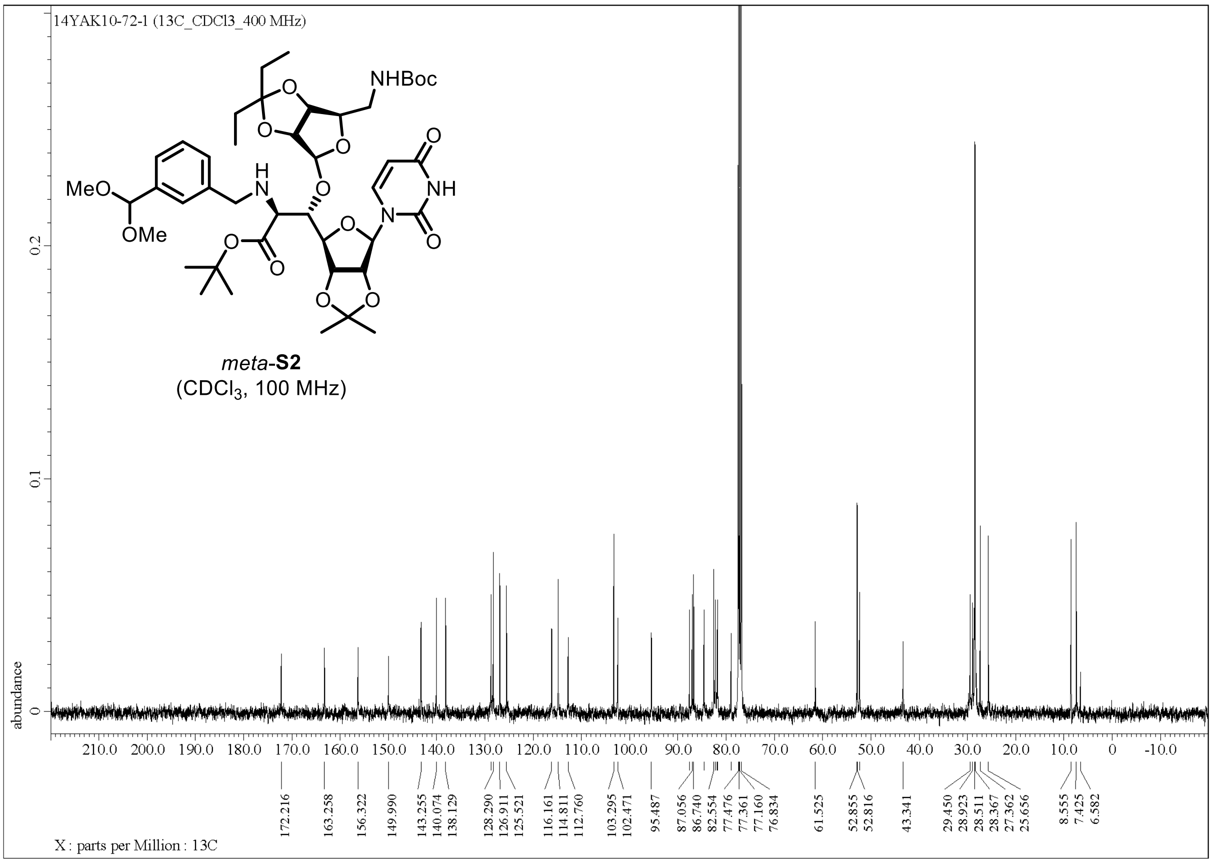

Supplementary Fig. 26. ^{13}C NMR spectrum of compound *meta*-**S2**.

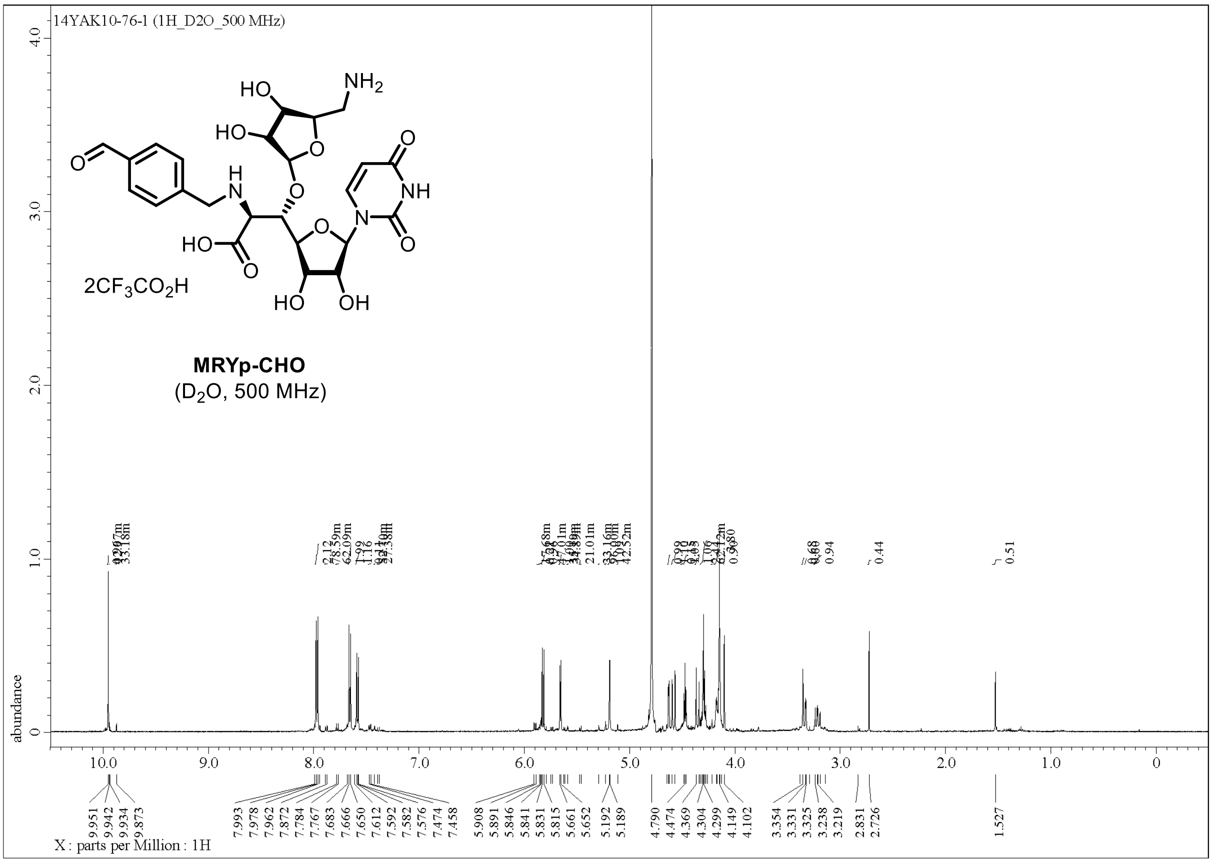

Supplementary Fig. 27. ^{1}H NMR spectrum of compound **MRYp-CHO**.

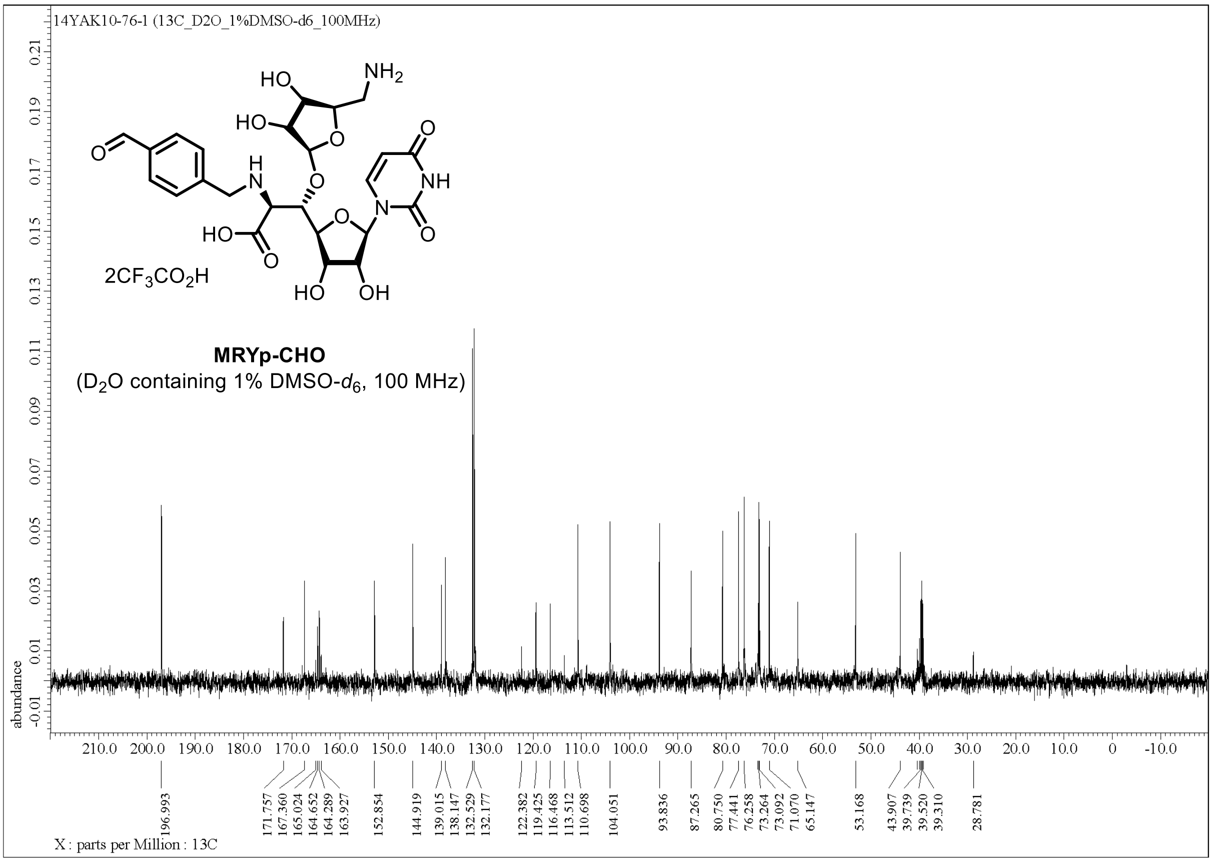

Supplementary Fig. 28. ^{13}C NMR spectrum of compound **MRYp-CHO**.

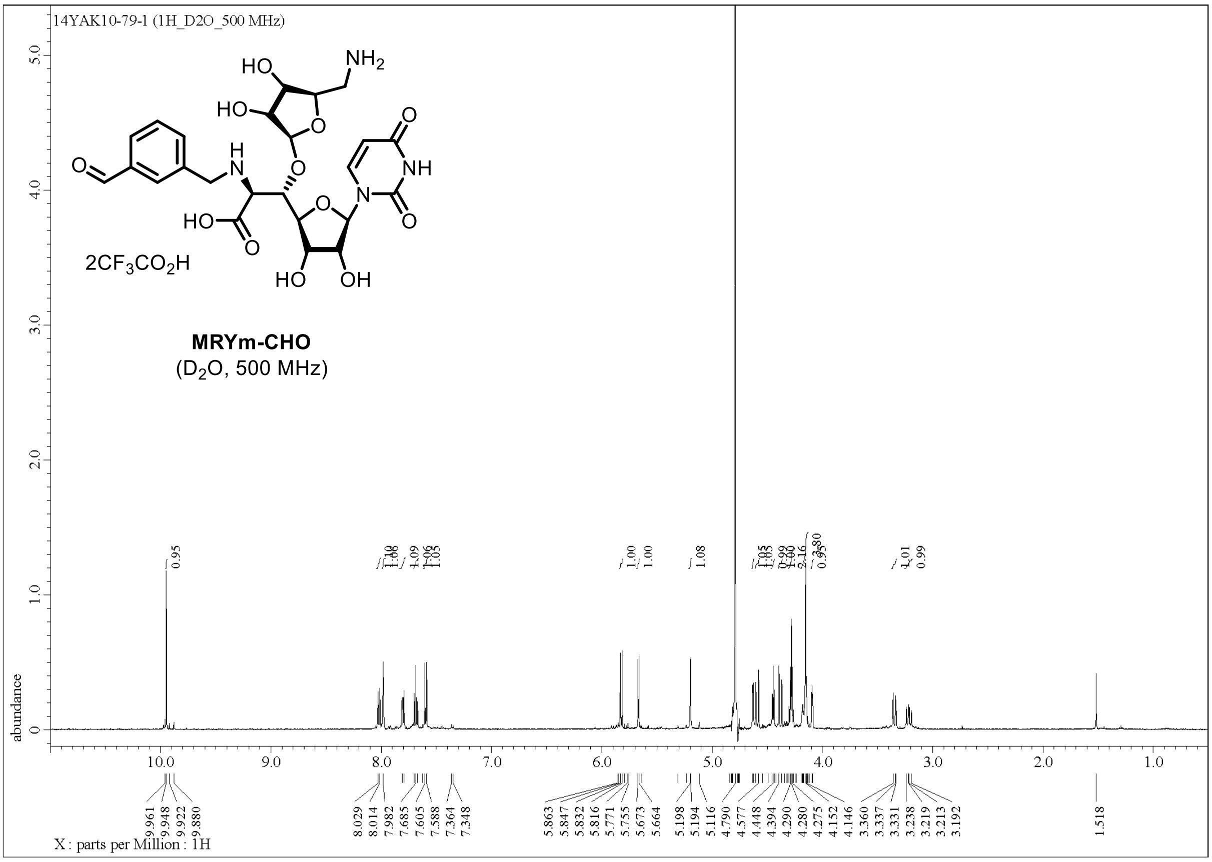

Supplementary Fig. 29. ^{1}H NMR spectrum of compound **MRYm-CHO**.

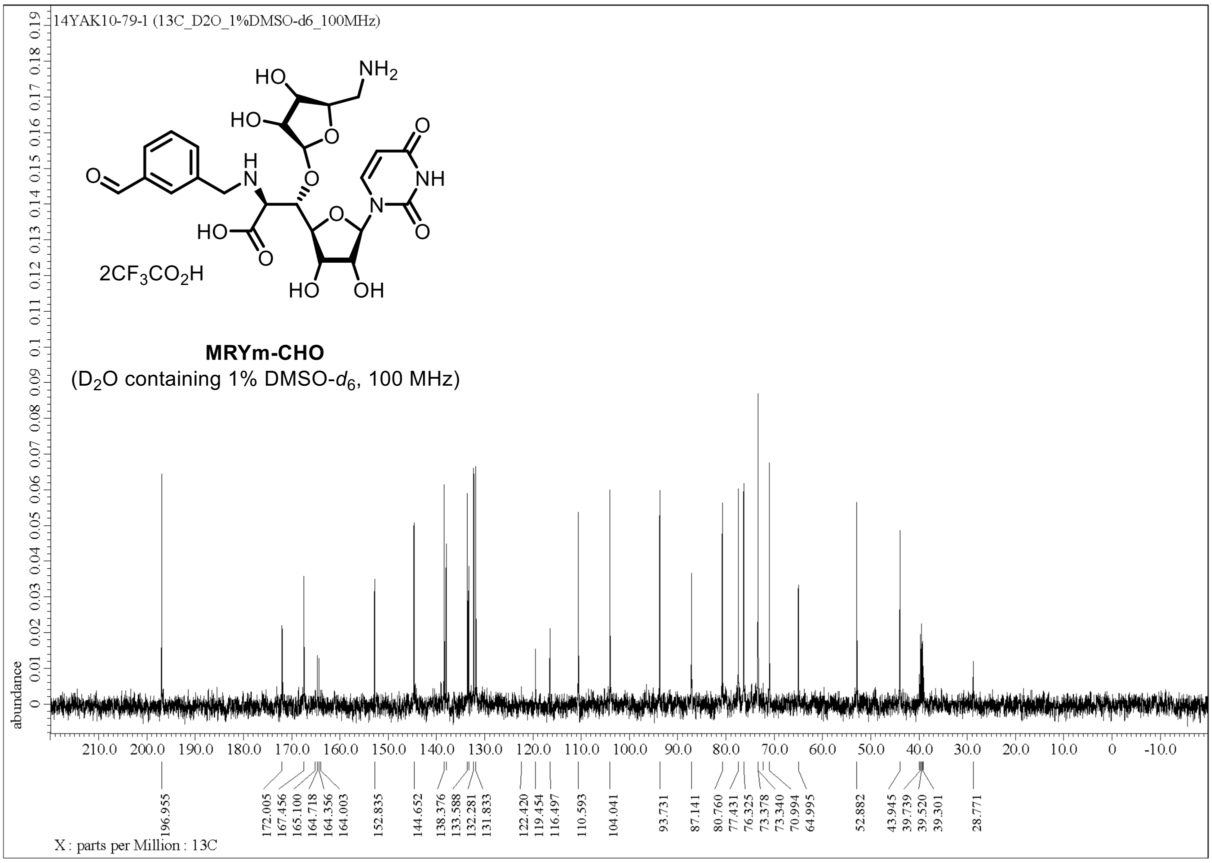

Supplementary Fig. 30. ^{13}C NMR spectrum of compound **MRYm-CHO**.

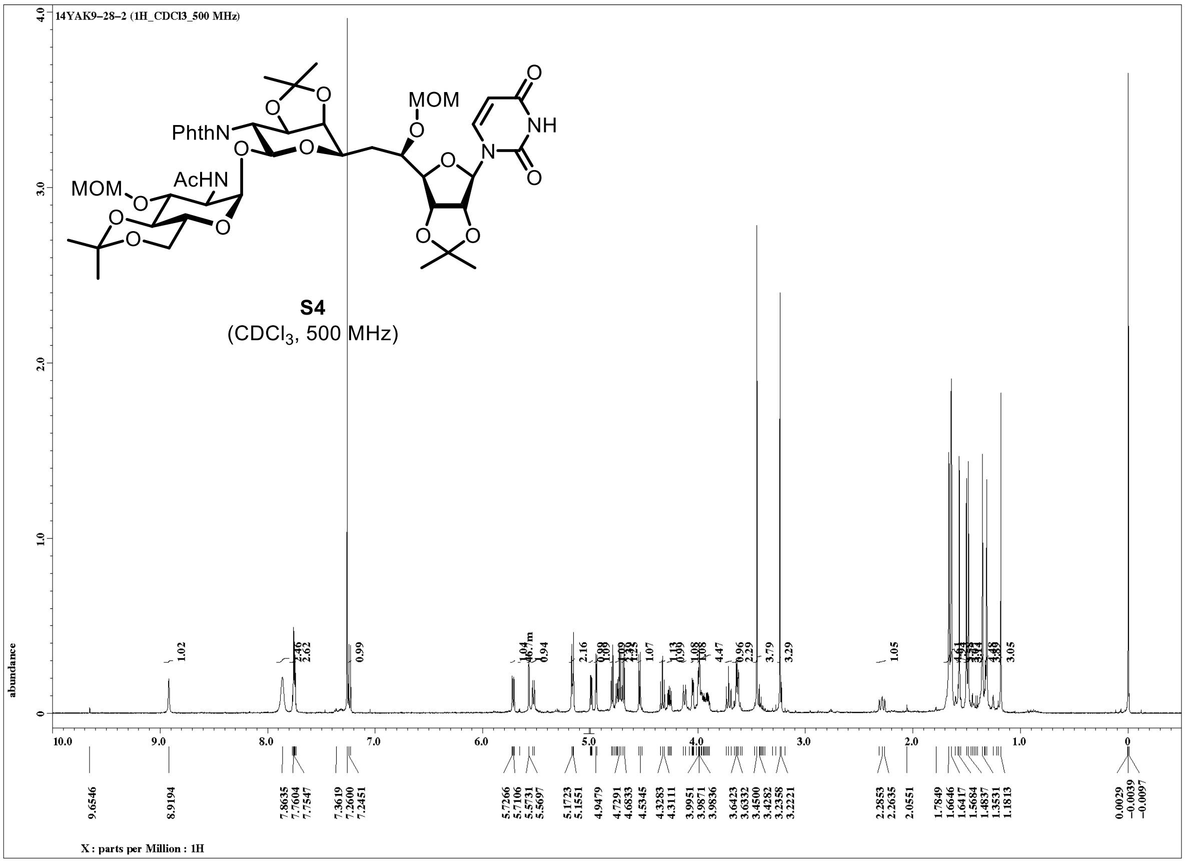

Supplementary Fig. 31. ^{1}H NMR spectrum of compound **S4**.

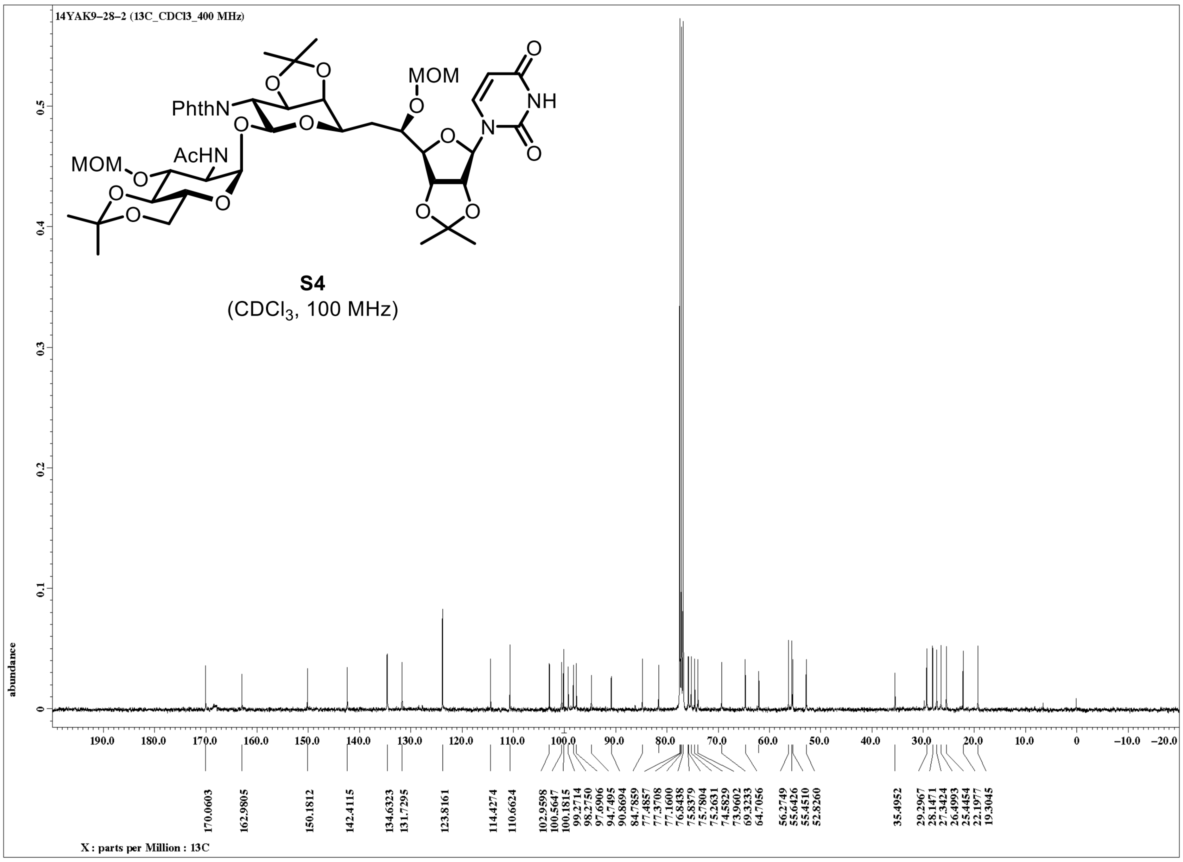

Supplementary Fig. 32. ^{13}C NMR spectrum of compound **S4**.

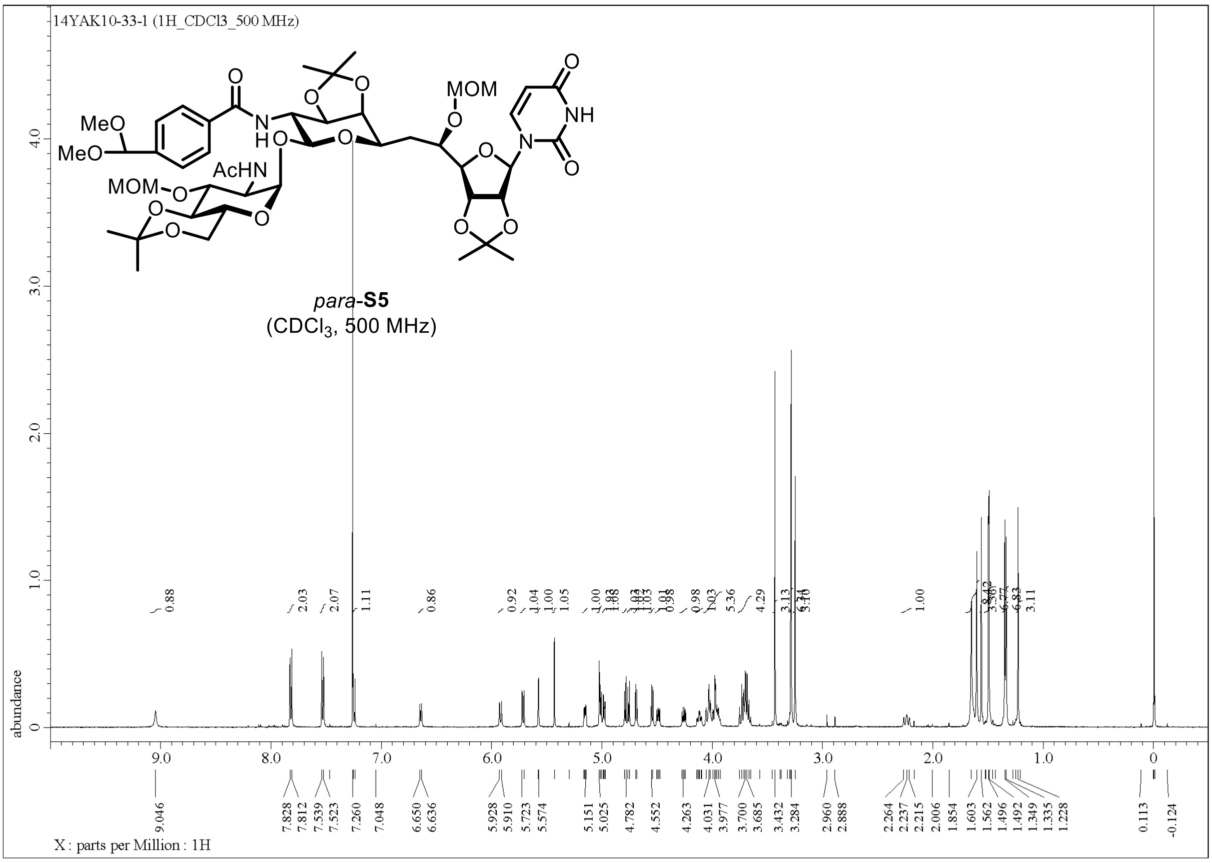

Supplementary Fig. 33. ^{1}H NMR spectrum of compound *para*-**S5**.

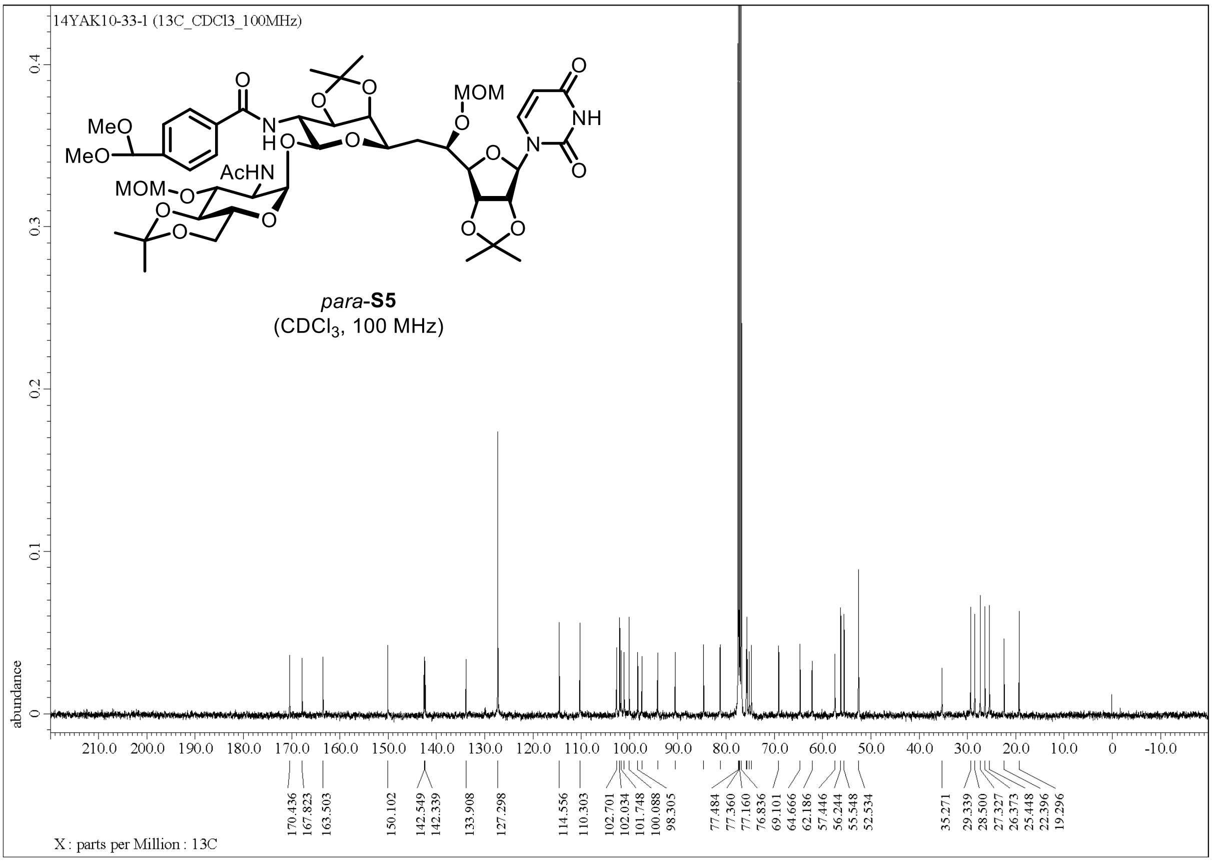

Supplementary Fig. 34. ^{13}C NMR spectrum of compound *para*-**S5**.

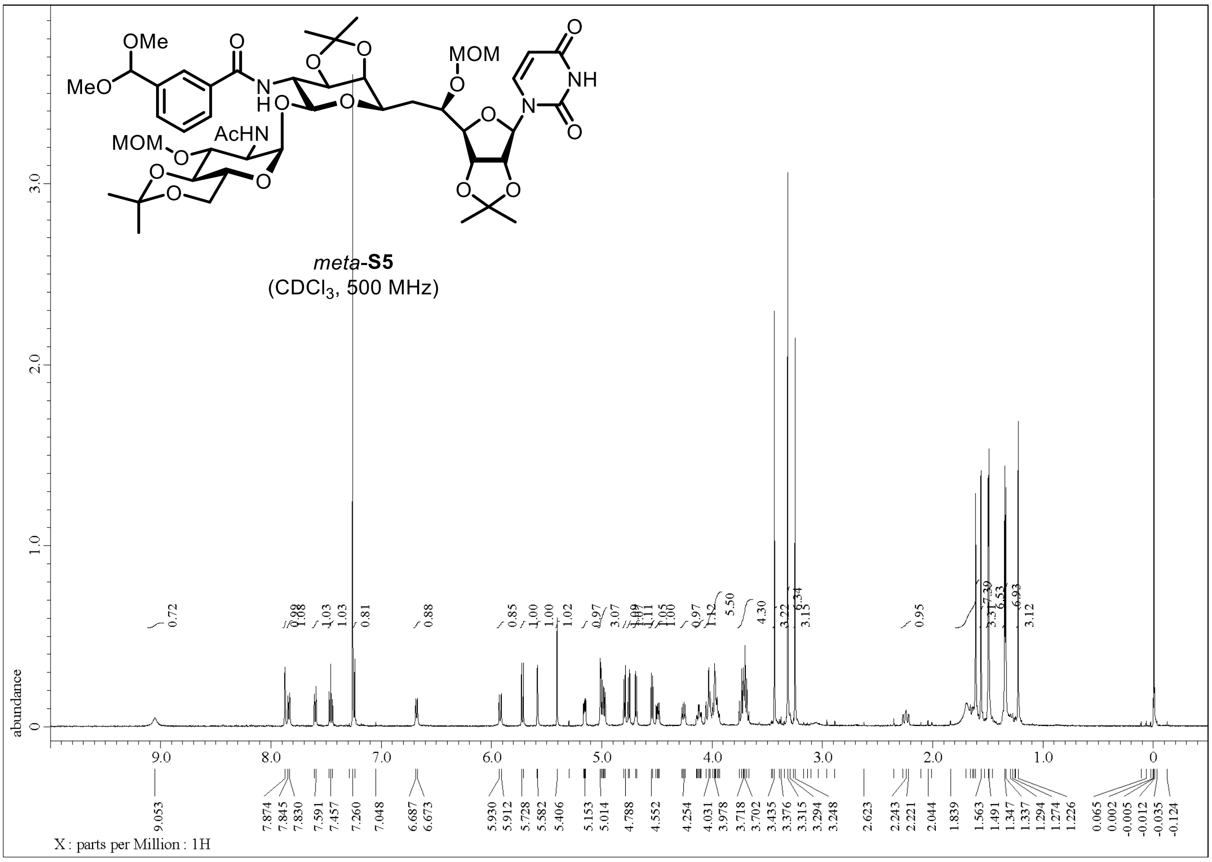

Supplementary Fig. 35. ^{1}H NMR spectrum of compound *meta*-**S5**.

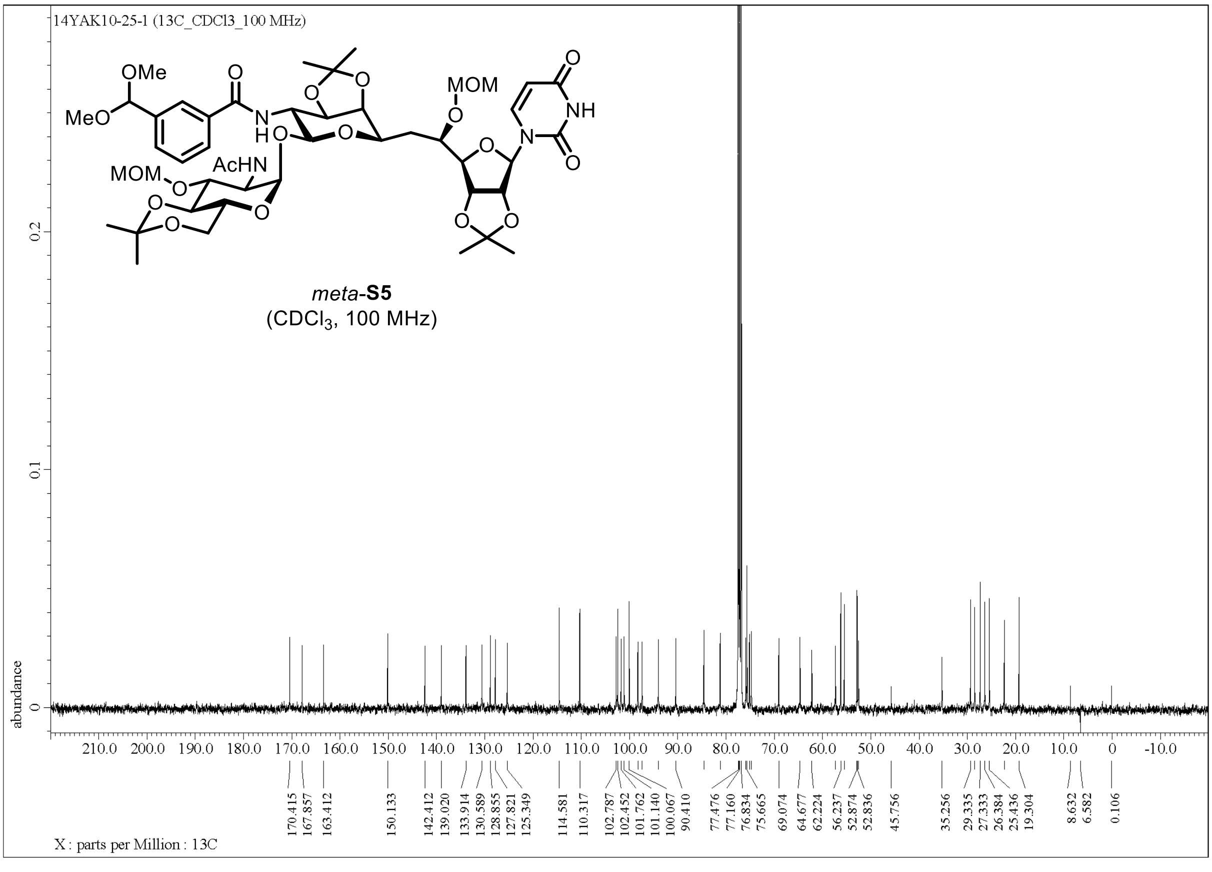

Supplementary Fig. 36. ^{13}C NMR spectrum of compound *meta*-**S5**.

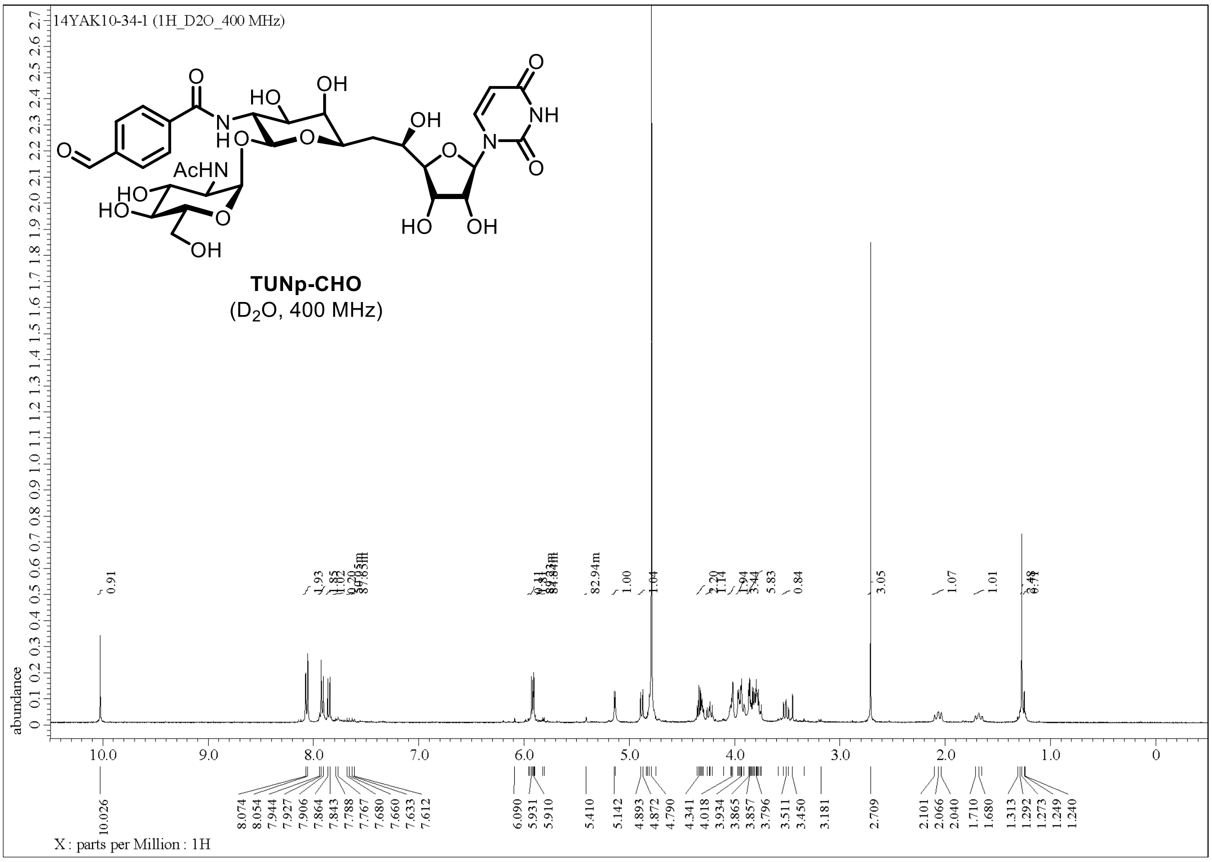

Supplementary Fig. 37. ^{1}H NMR spectrum of compound **TUNp-CHO**.

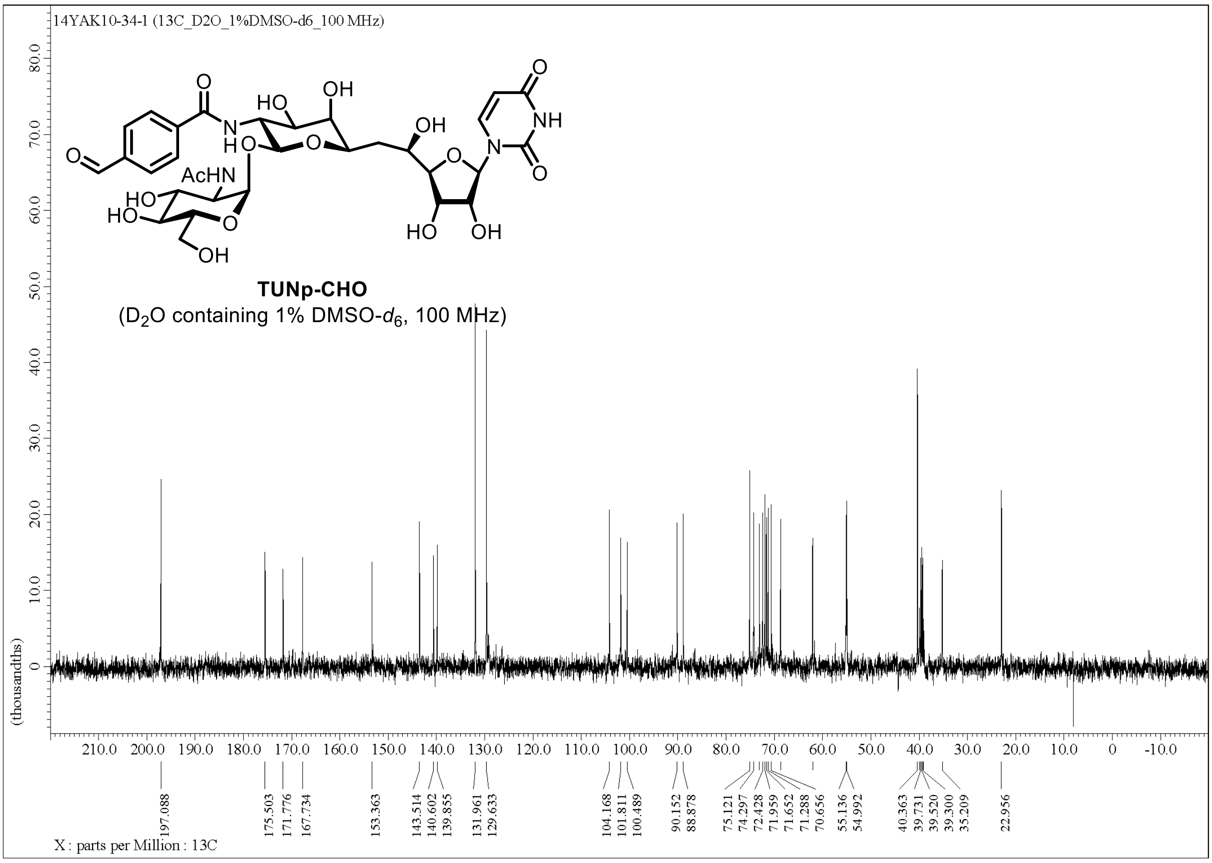

Supplementary Fig. 38. ^{13}C NMR spectrum of compound **TUNp-CHO**.

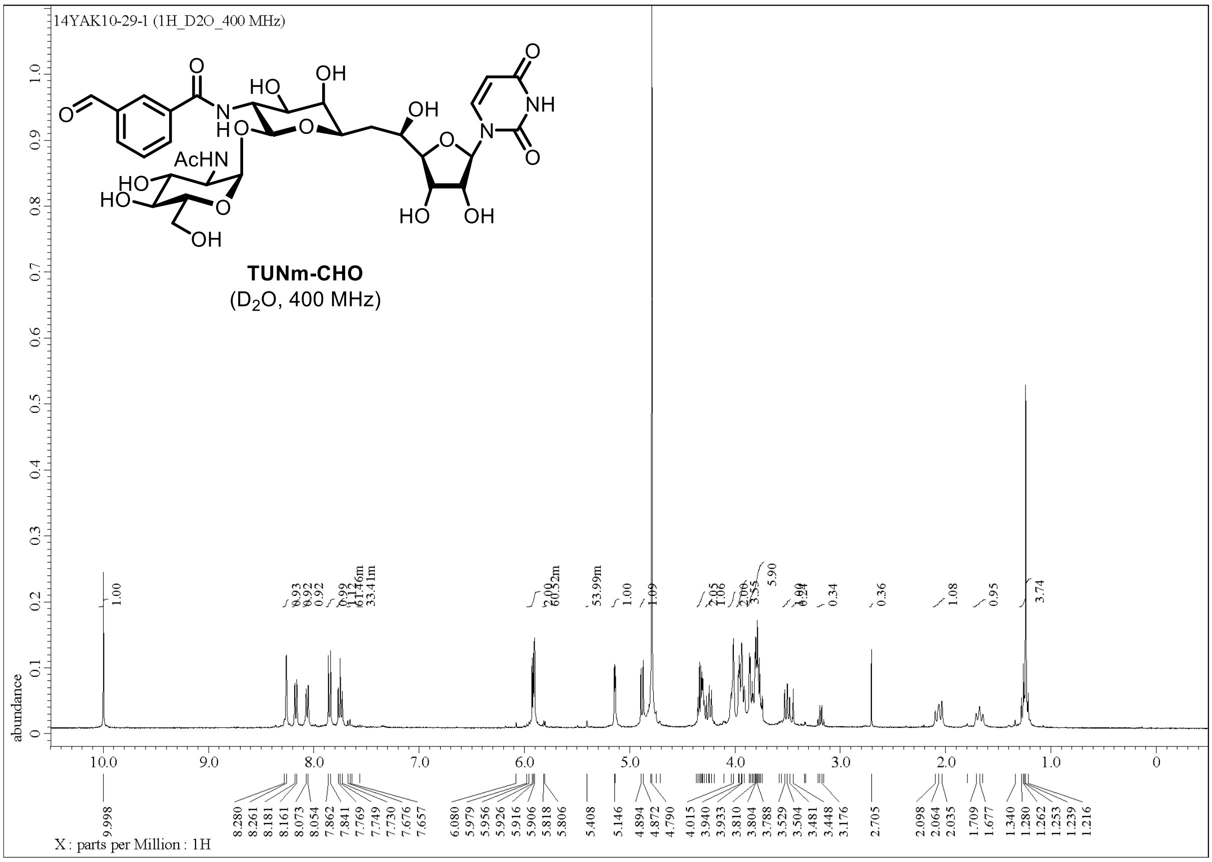

Supplementary Fig. 39. ^{1}H NMR spectrum of compound **TUNm-CHO**.

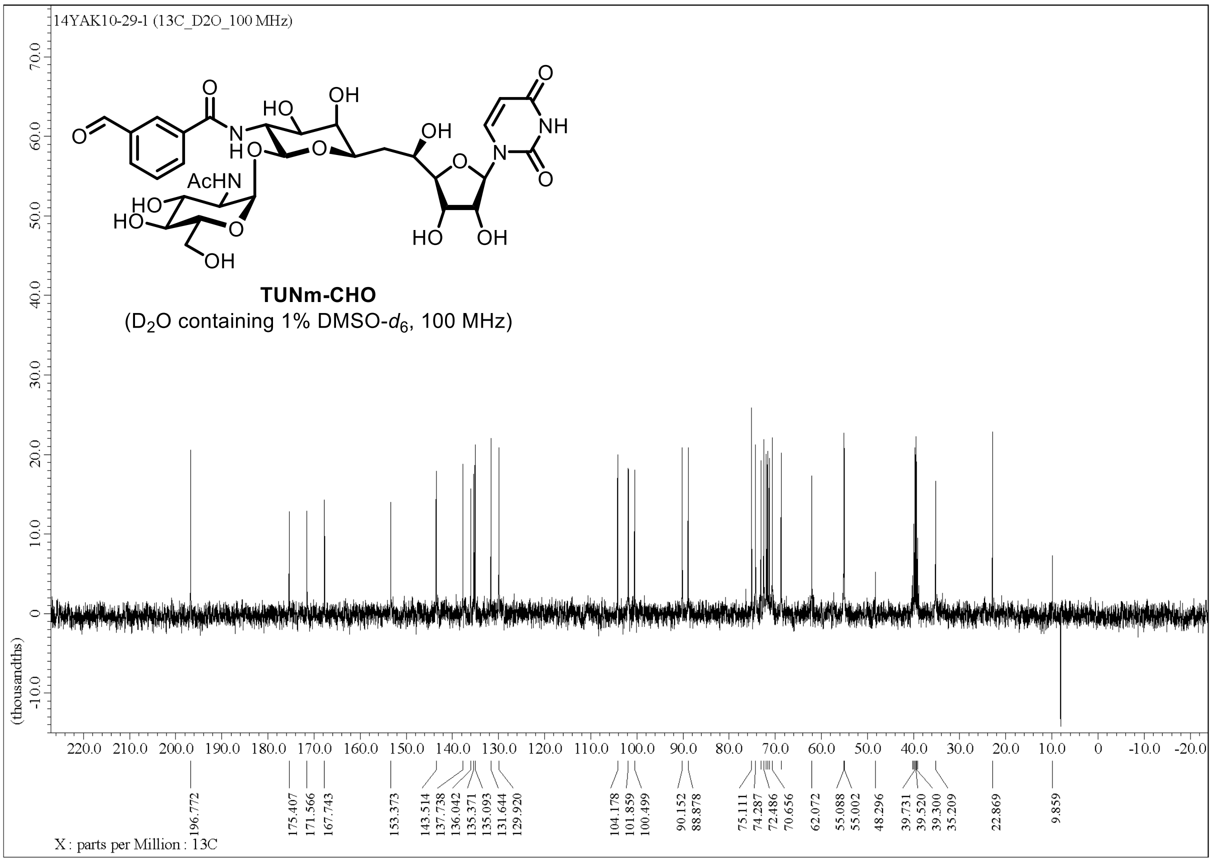

Supplementary Fig. 40. ^{13}C NMR spectrum of compound **TUNm-CHO**.

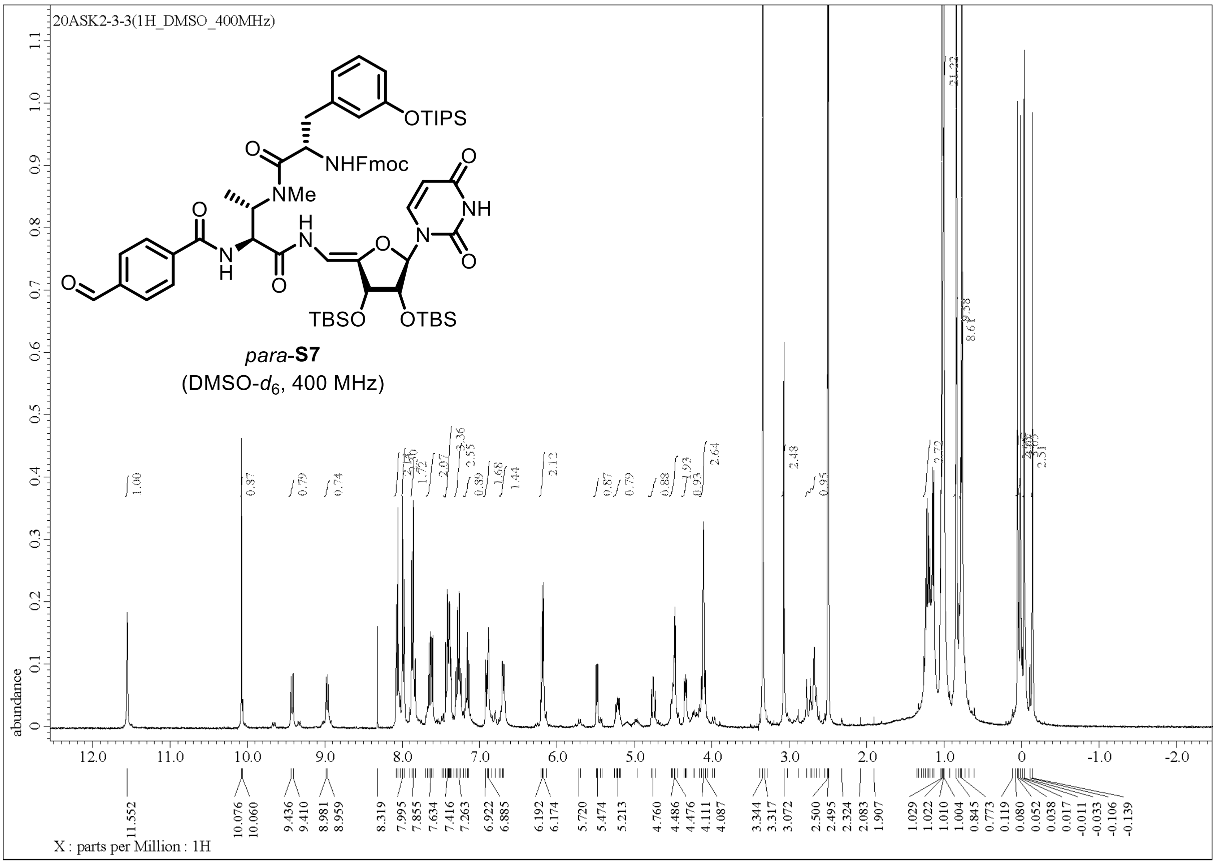

Supplementary Fig. 41. ^{1}H NMR spectrum of compound *para*-**S7**.

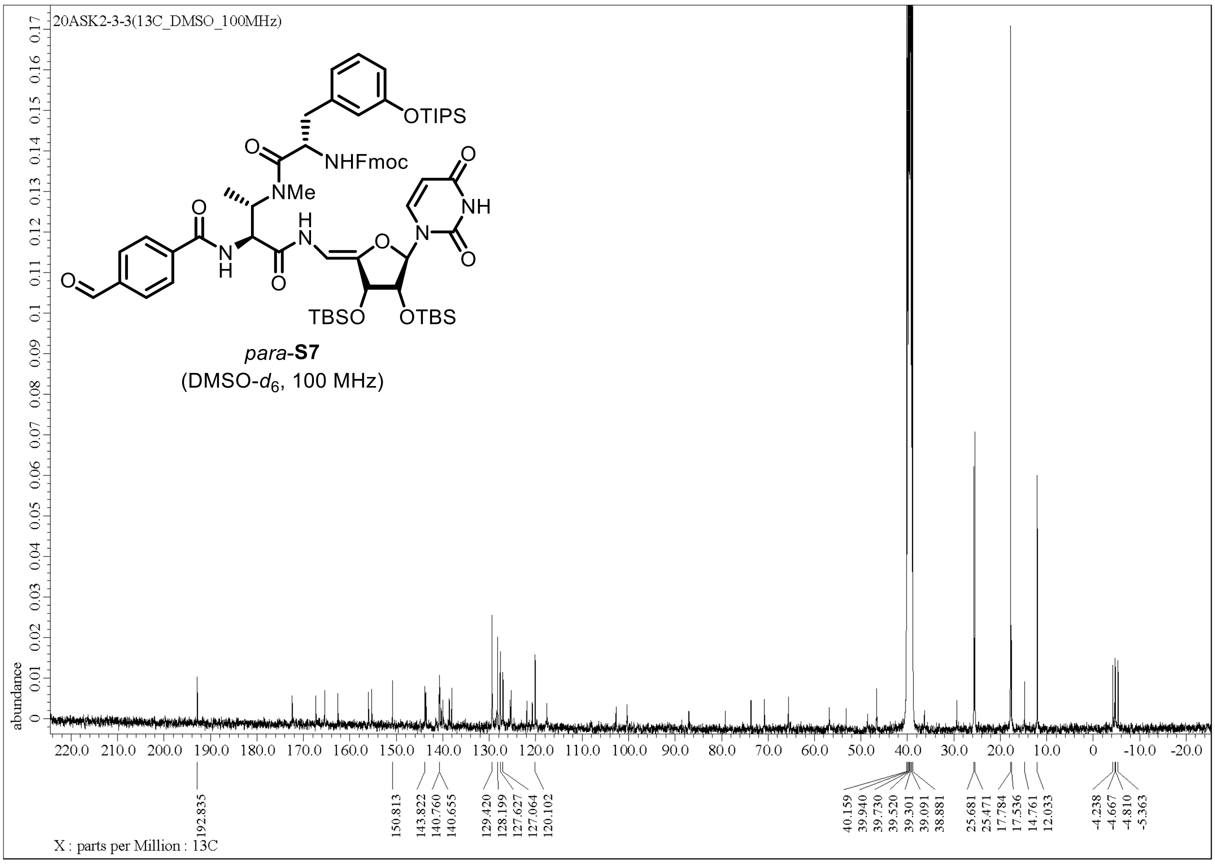

Supplementary Fig. 42. ^{13}C NMR spectrum of compound *para*-**S7**.

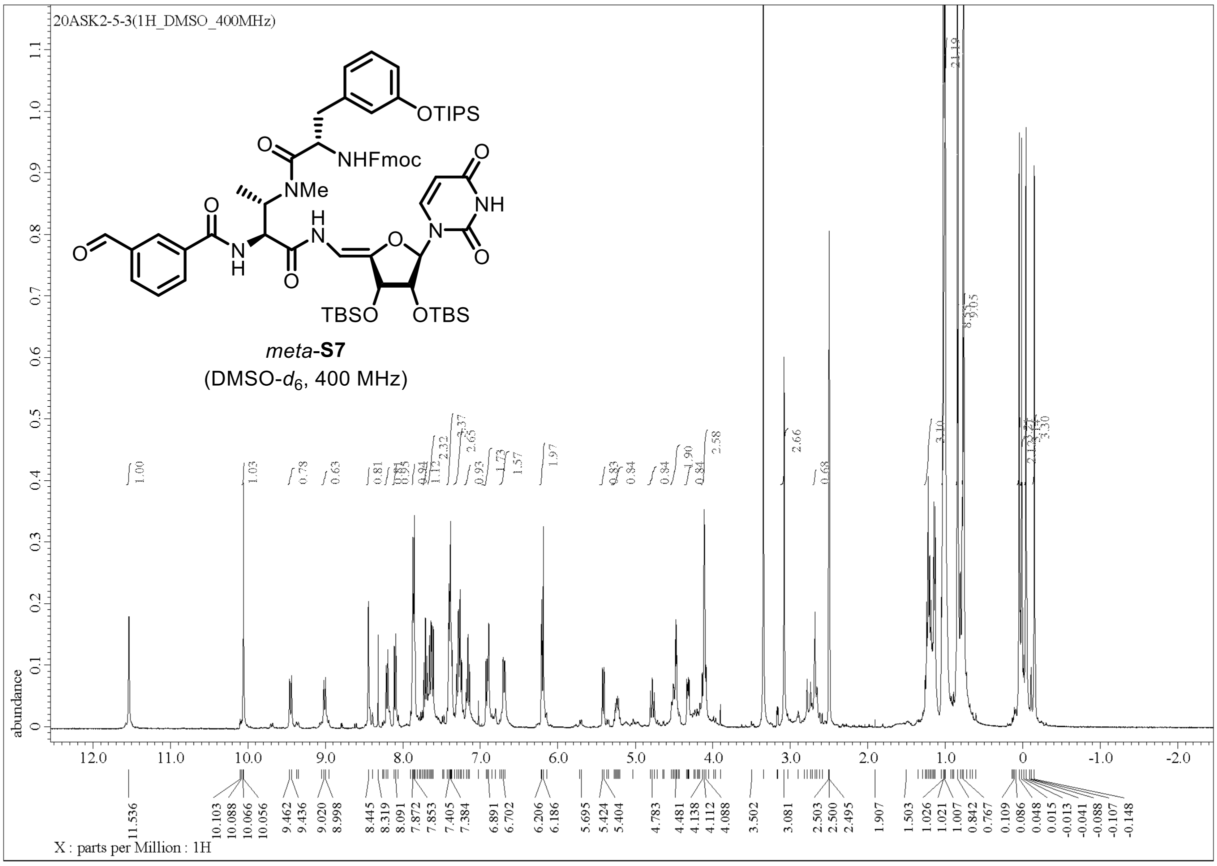

Supplementary Fig. 43. ^{1}H NMR spectrum of compound *meta*-**S7**.

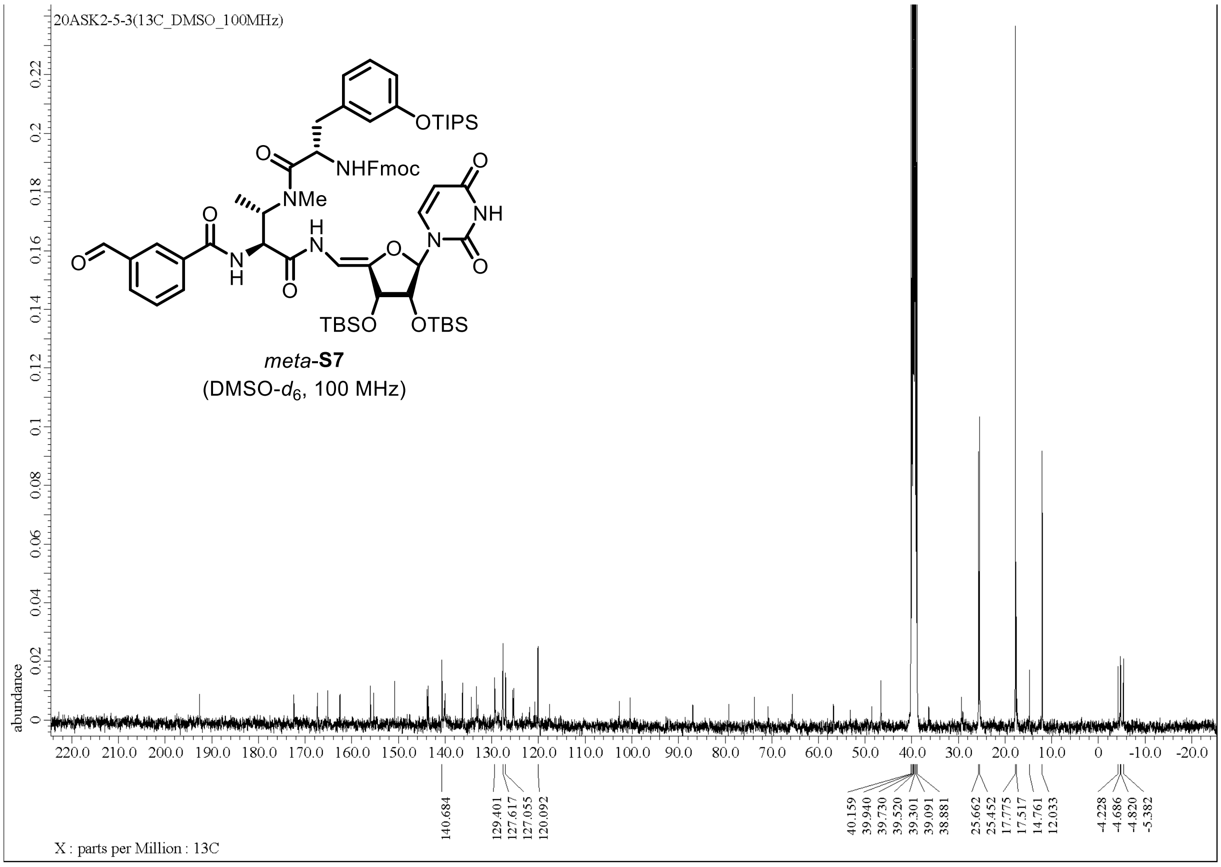

Supplementary Fig. 44. ^{13}C NMR spectrum of compound *meta*-**S7**.

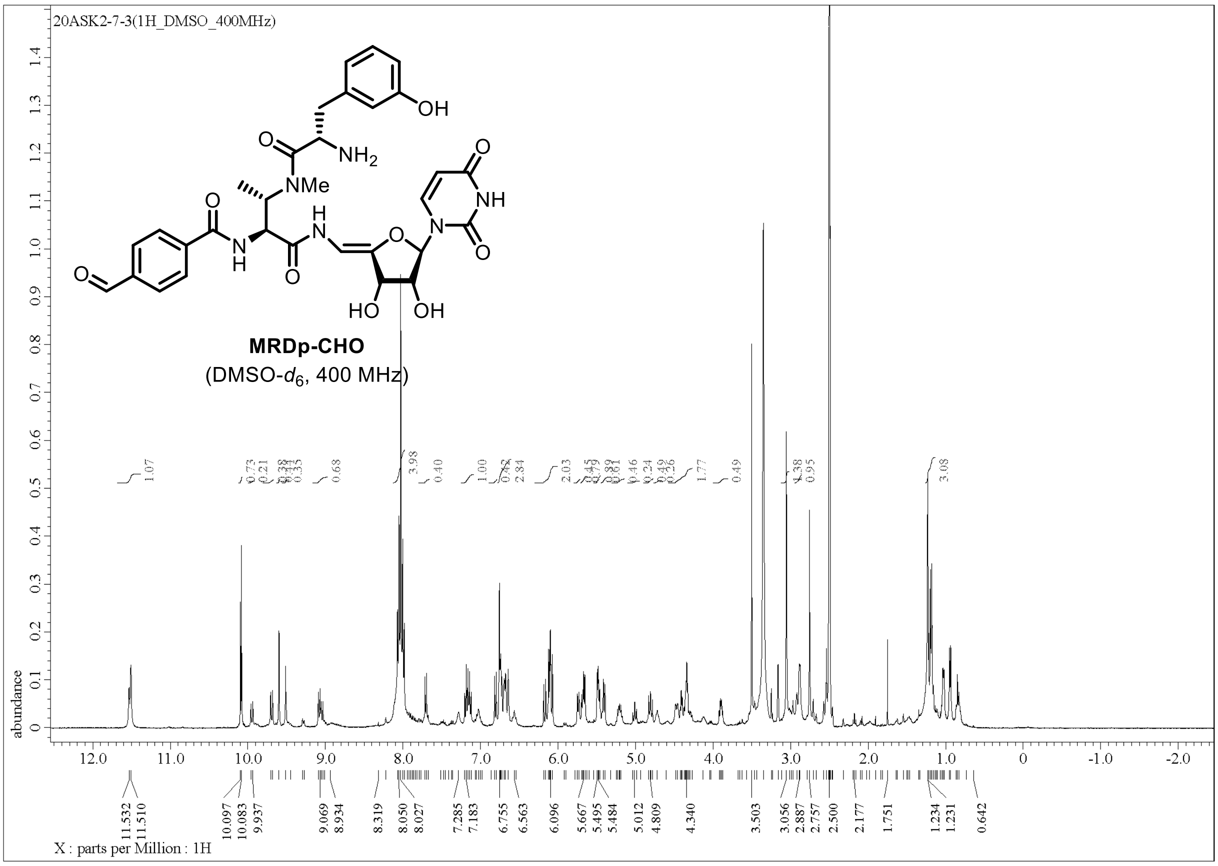

Supplementary Fig. 45. ^{1}H NMR spectrum of compound **MRDp-CHO**.

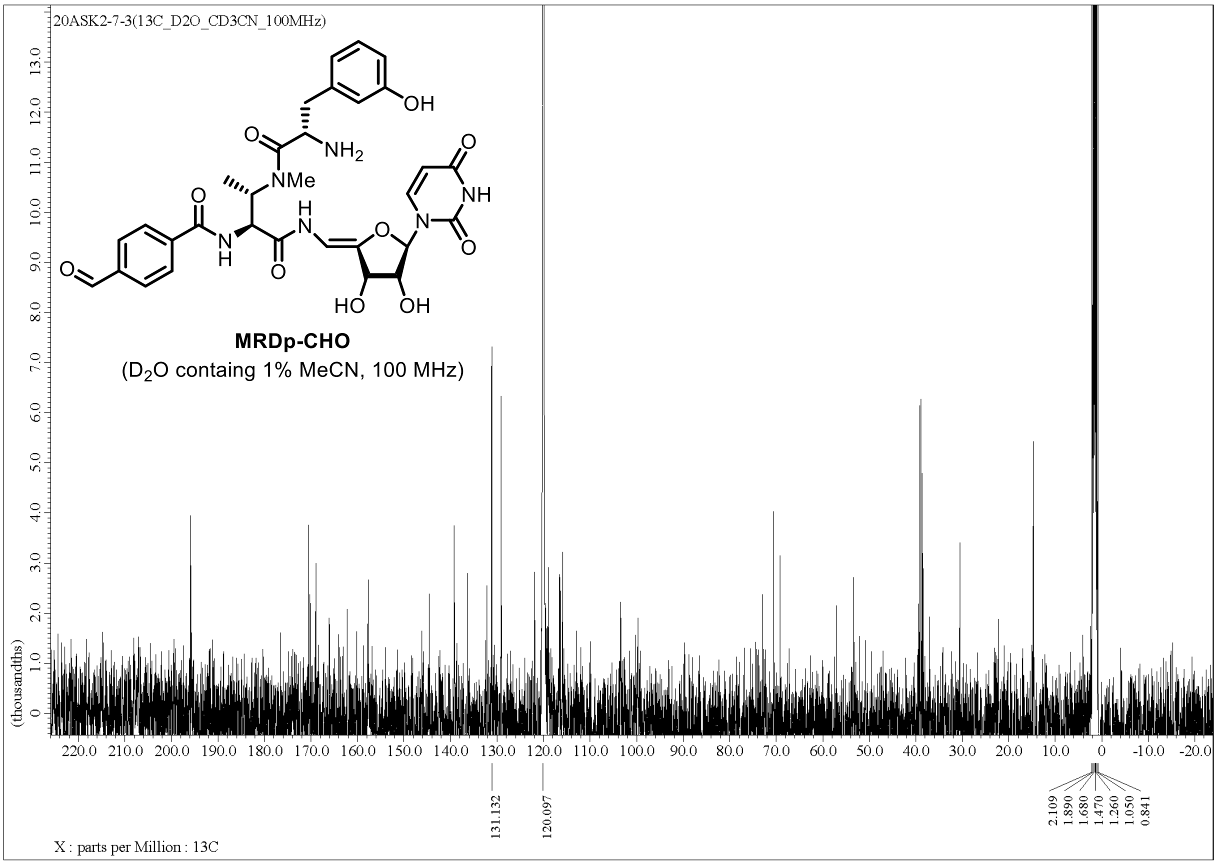

Supplementary Fig. 46. ^{13}C NMR spectrum of compound **MRDp-CHO**.

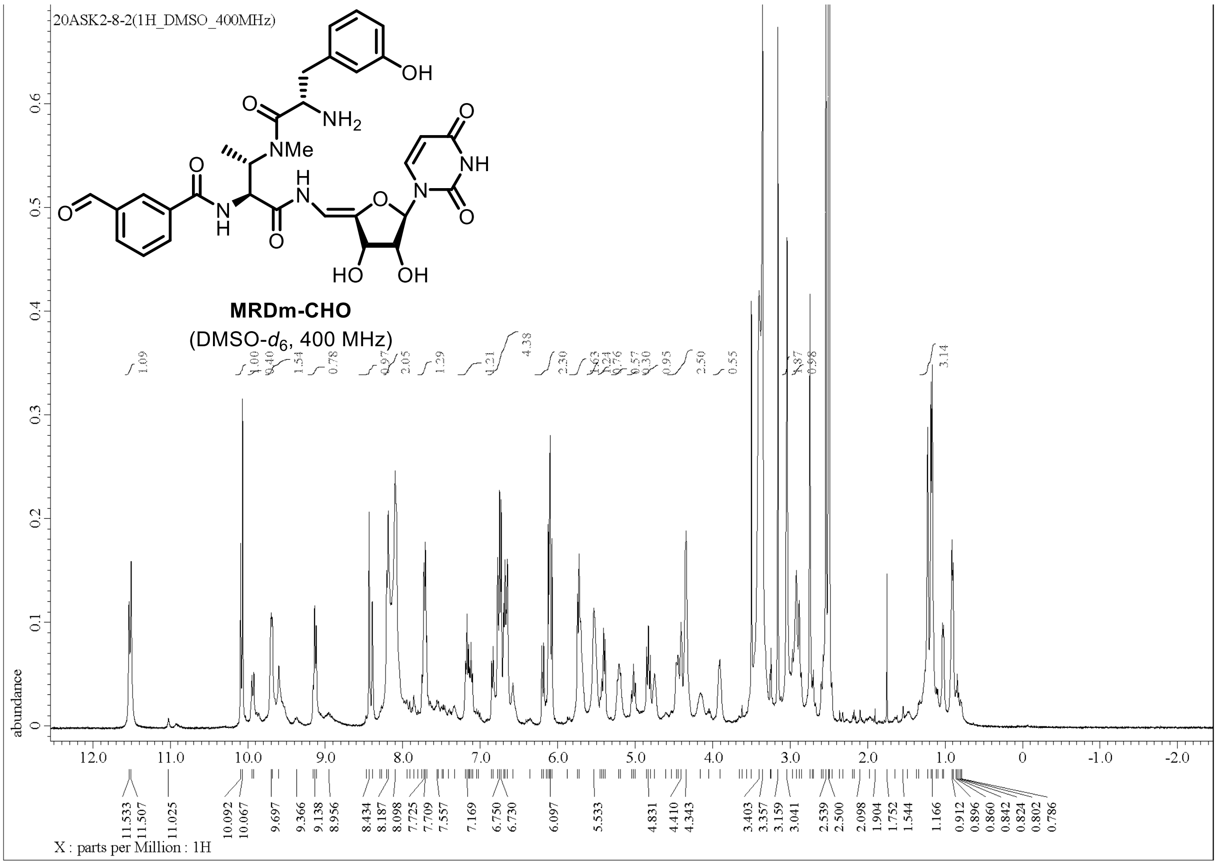

Supplementary Fig. 47. ^{1}H NMR spectrum of compound **MRDm-CHO**.

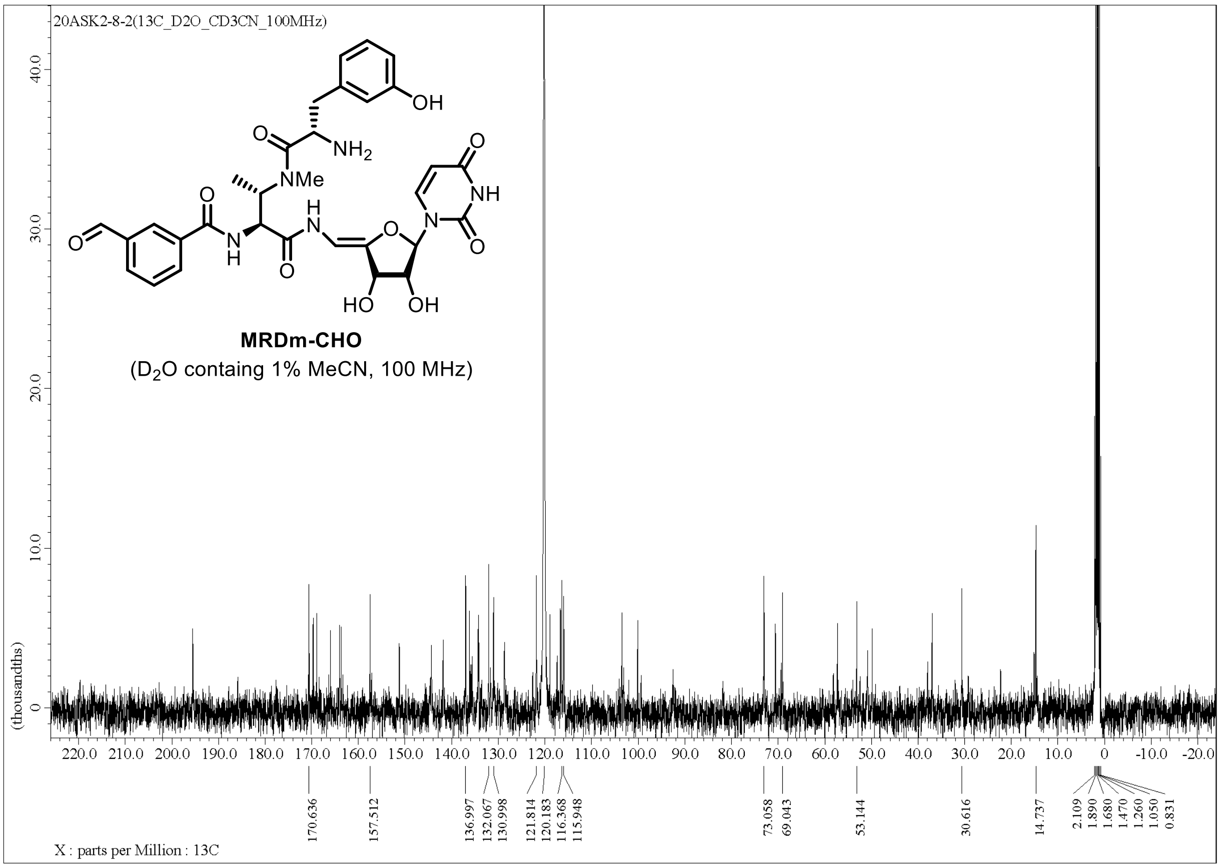

Supplementary Fig. 48. ^{13}C NMR spectrum of compound **MRDm-CHO**.

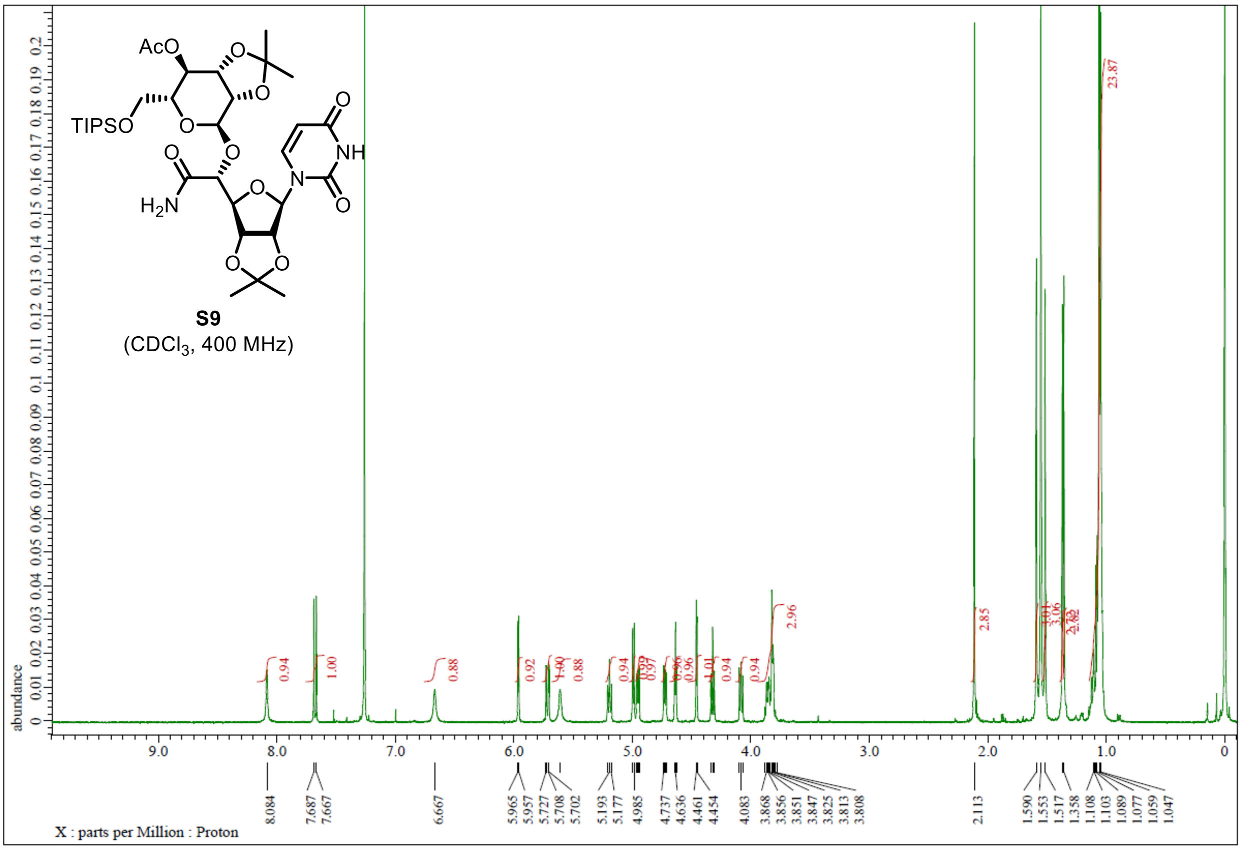

Supplementary Fig. 49. ^{1}H NMR spectrum of compound **S9**.

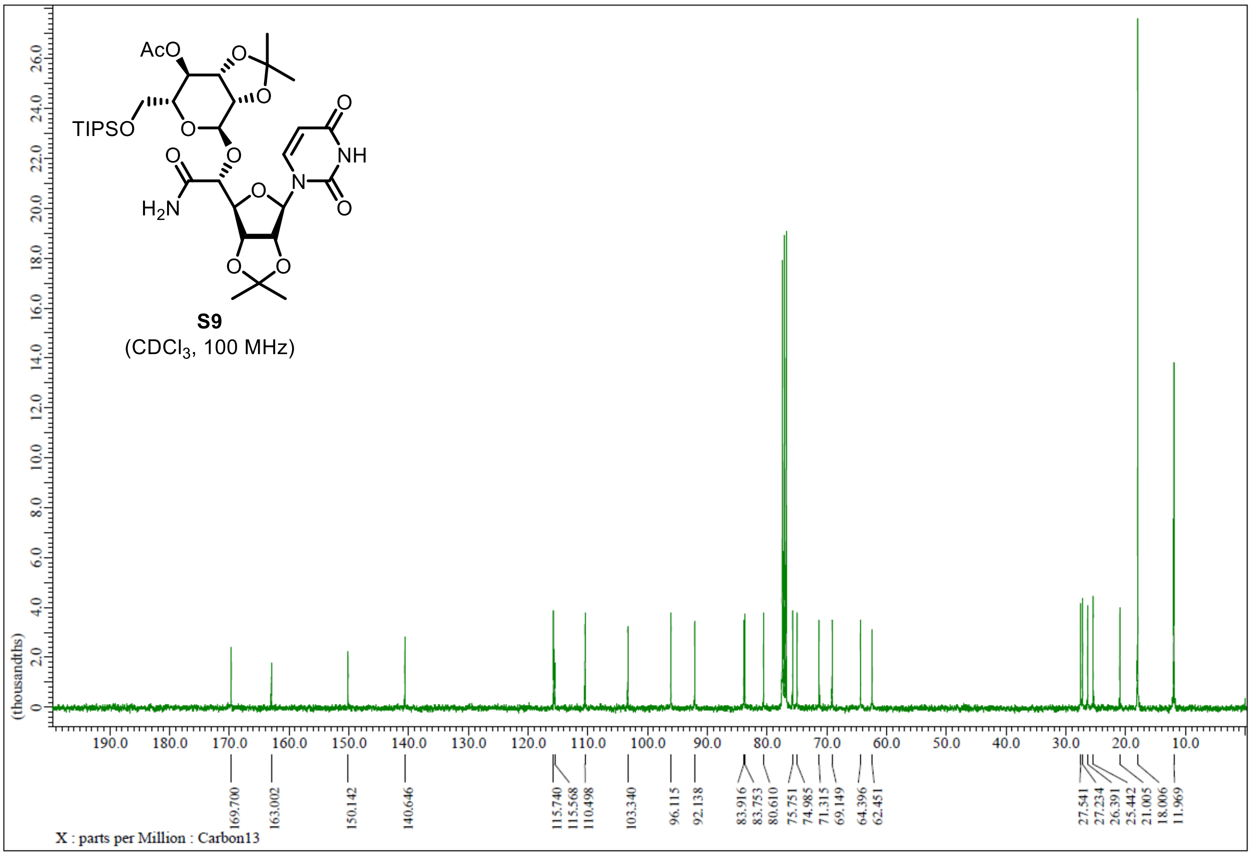

Supplementary Fig. 50. ^{13}C NMR spectrum of compound **S9**.

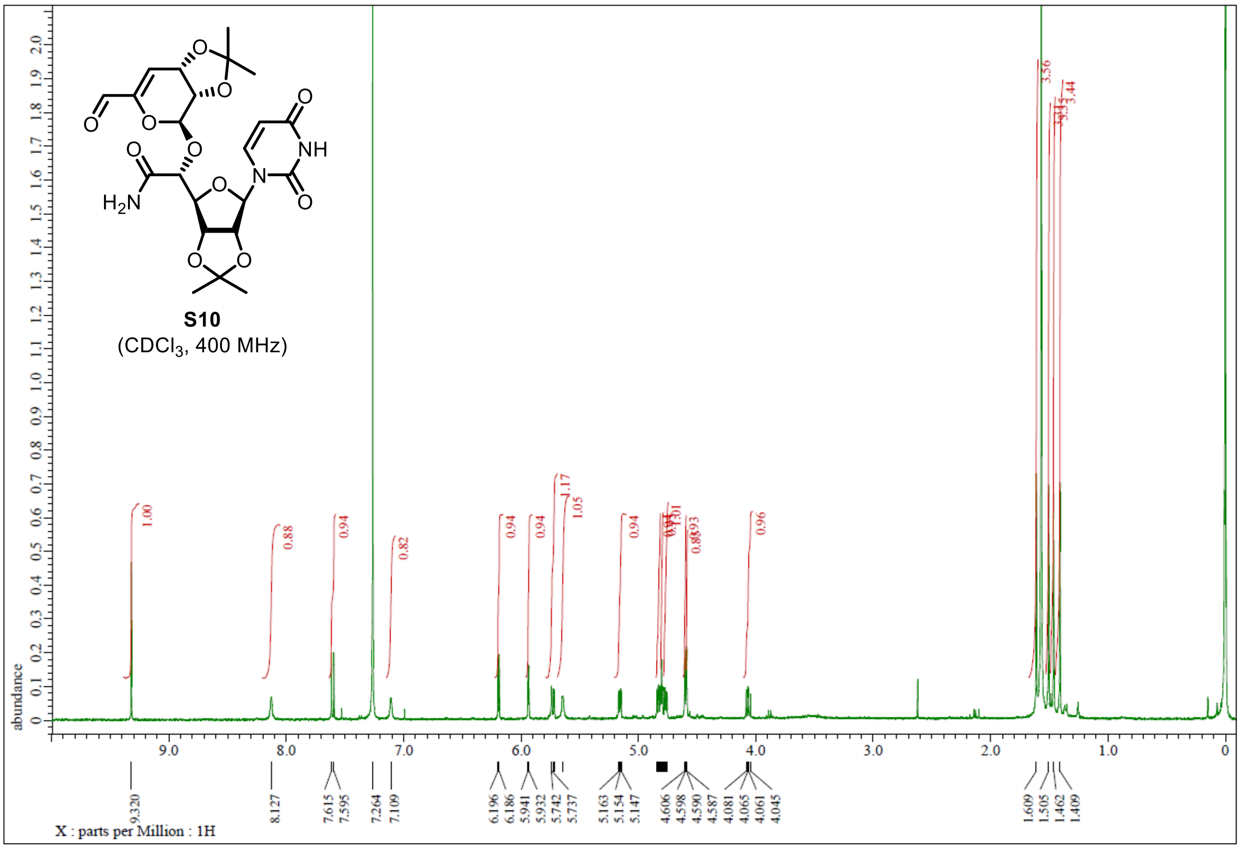

Supplementary Fig. 51. ^{1}H NMR spectrum of compound **S10**.

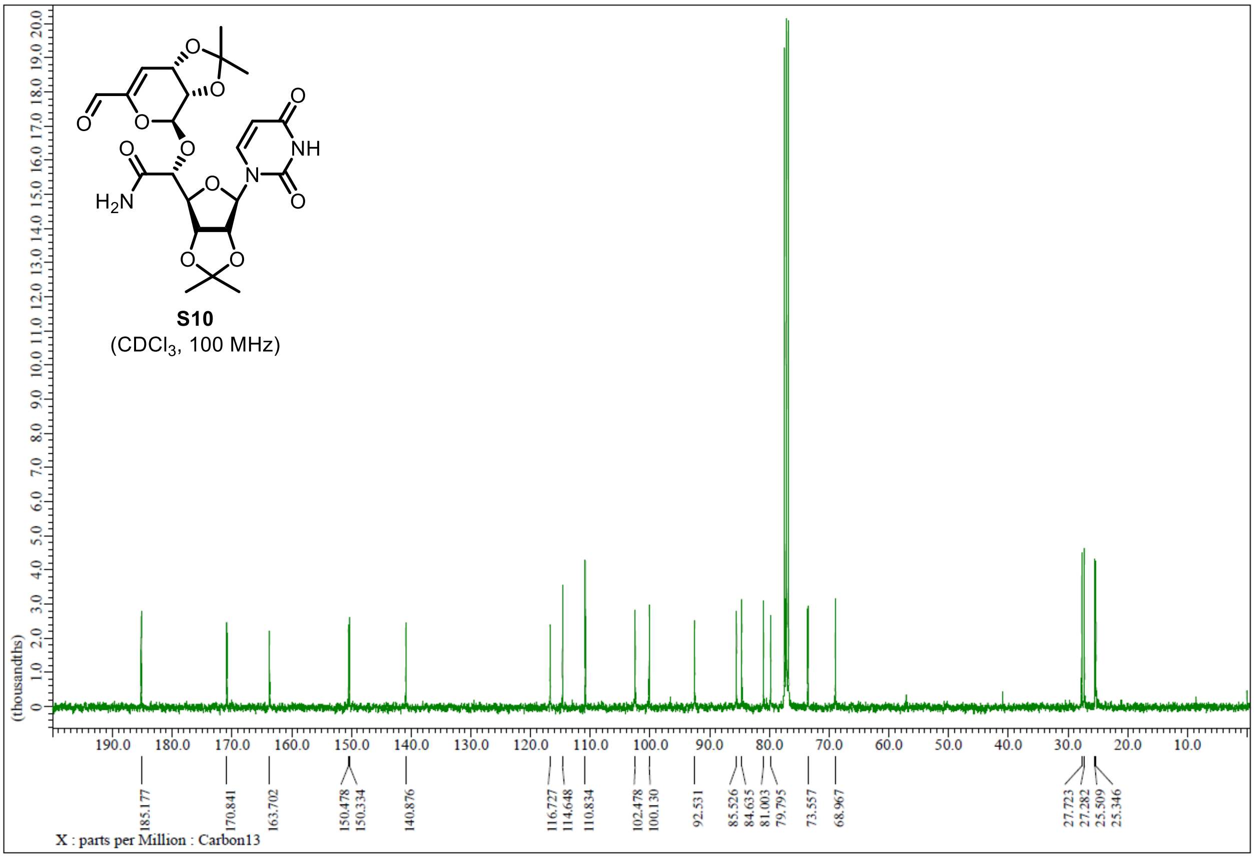

Supplementary Fig. 52. ^{13}C NMR spectrum of compound **S10**.

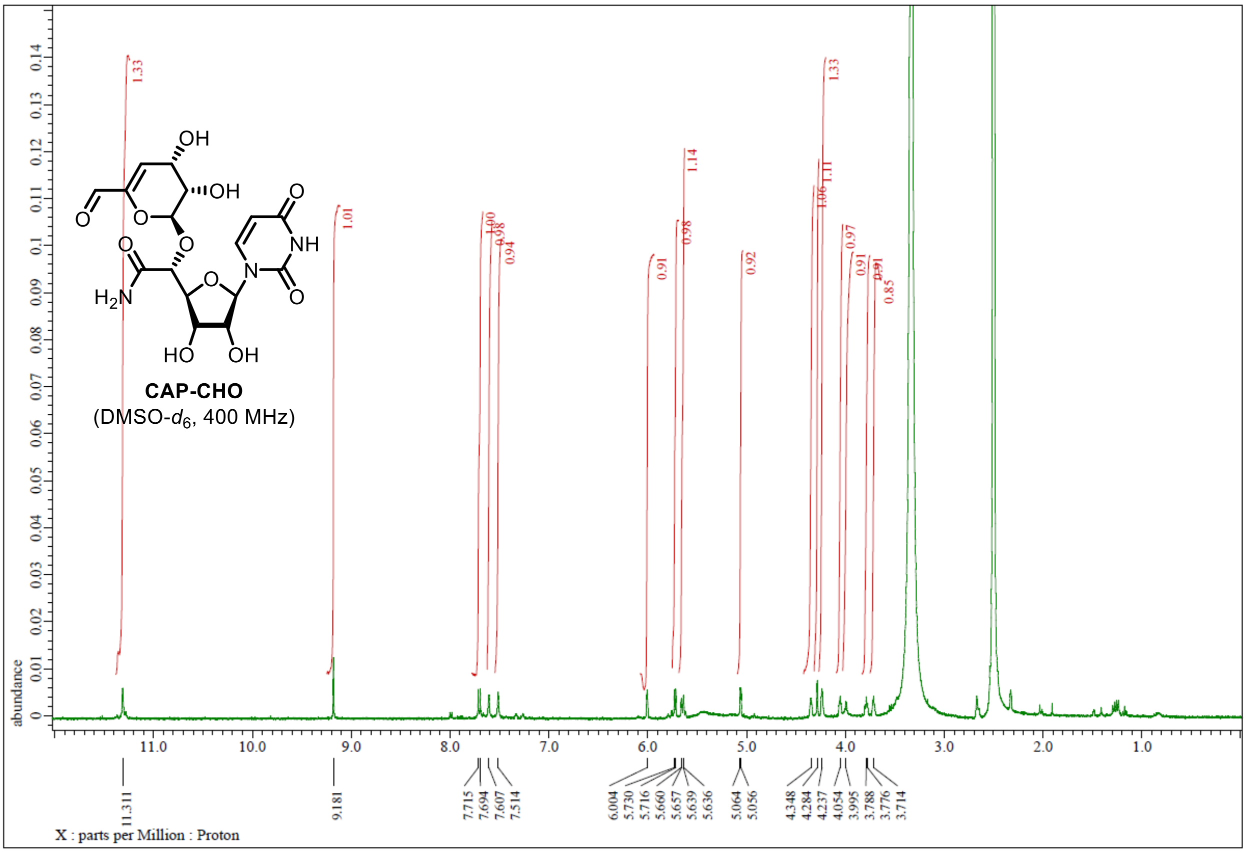

Supplementary Fig. 53. ^{1}H NMR spectrum of compound **CAP-CHO**.

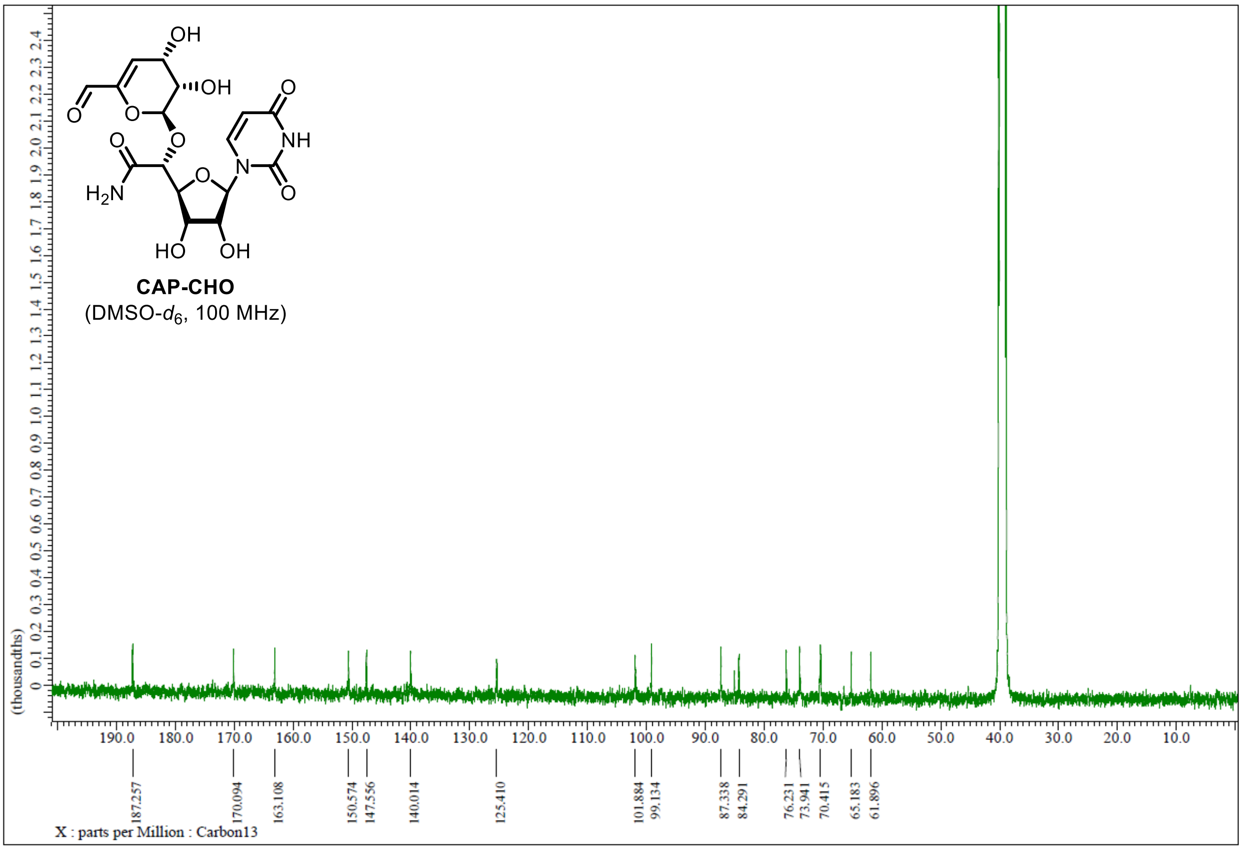

Supplementary Fig. 54. ^{13}C NMR spectrum of compound **CAP-CHO**.

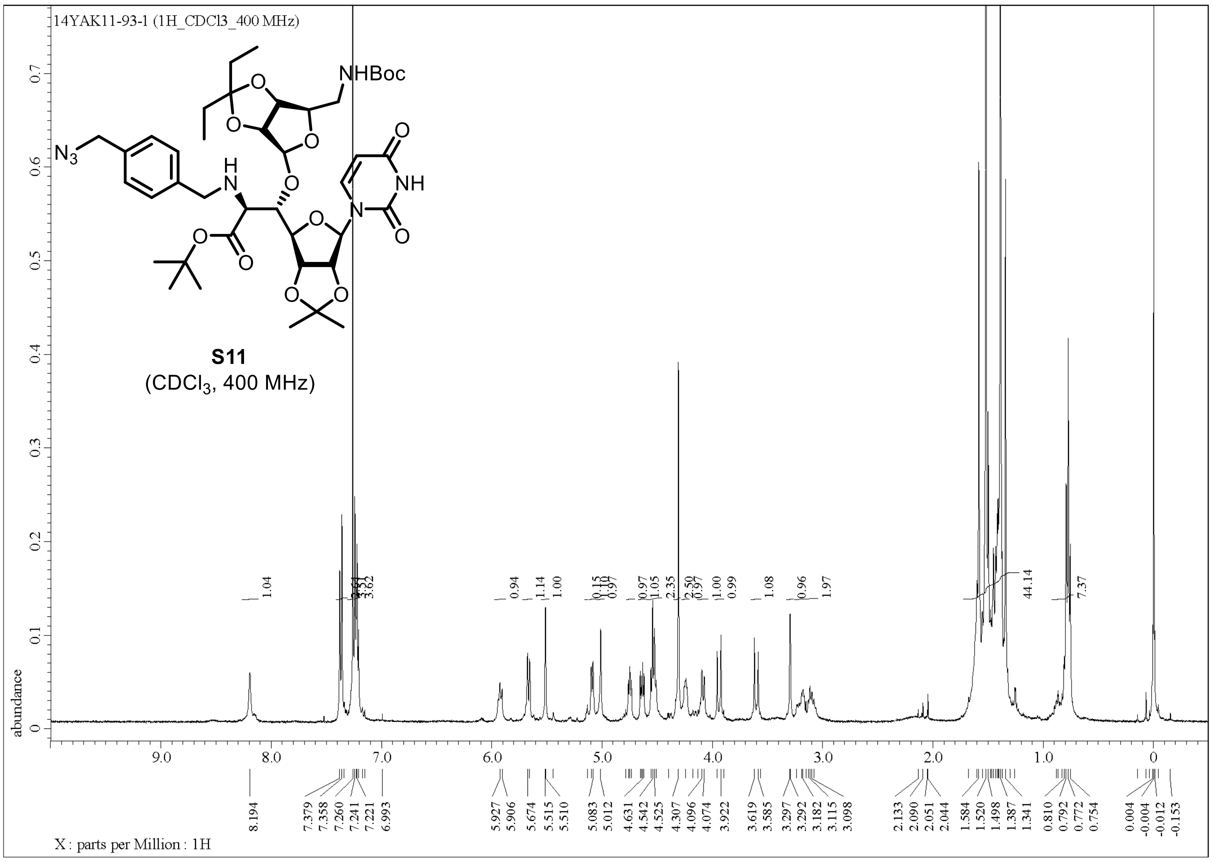

Supplementary Fig. 55. ^{1}H NMR spectrum of compound **S11**.

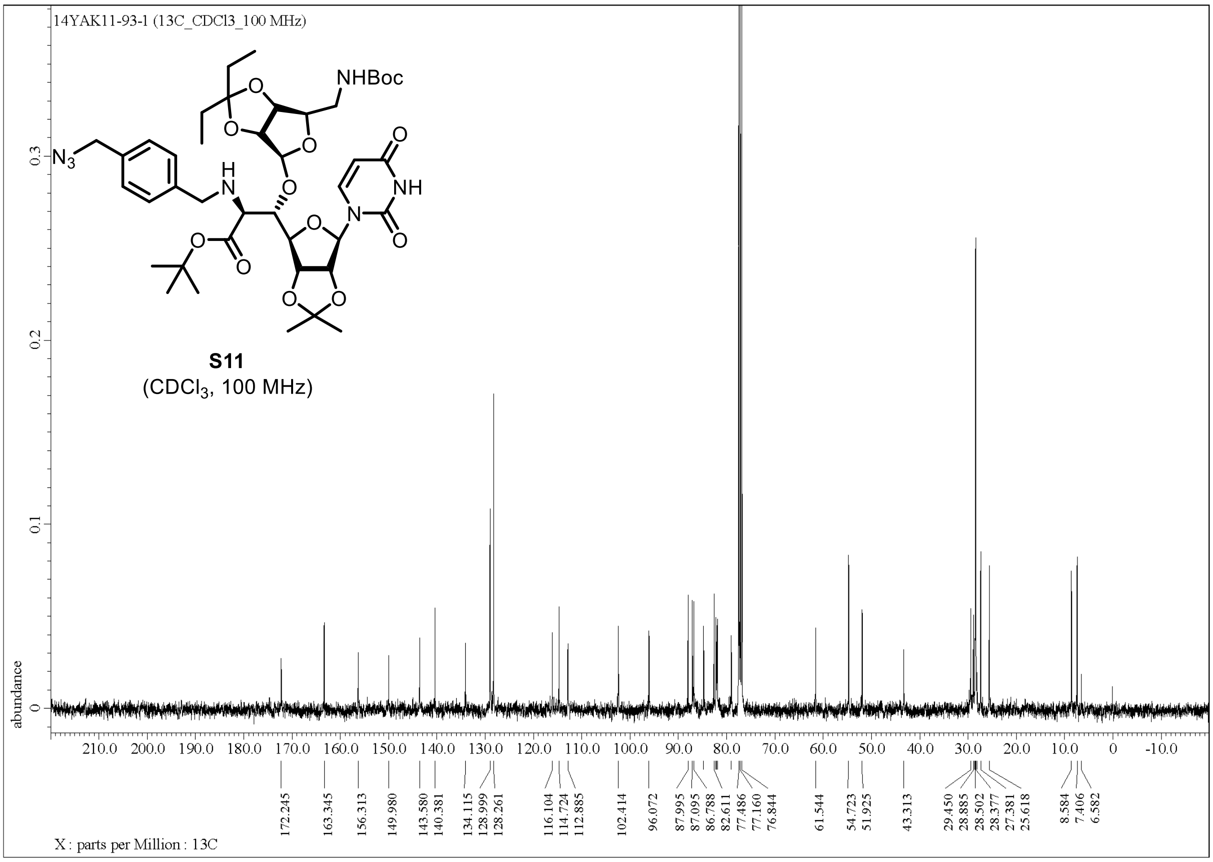

Supplementary Fig. 56. ^{13}C NMR spectrum of compound **S11**.

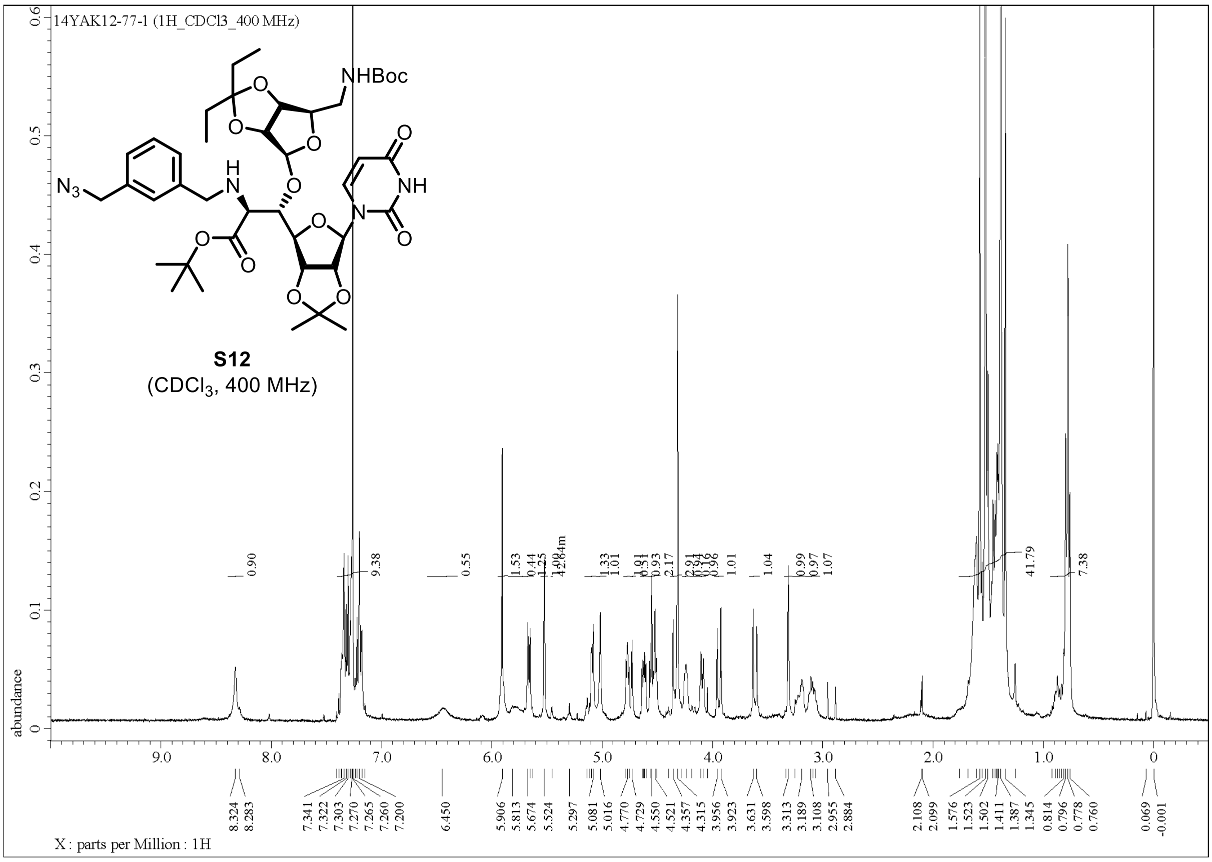

Supplementary Fig. 57. ^{1}H NMR spectrum of compound **S12**.

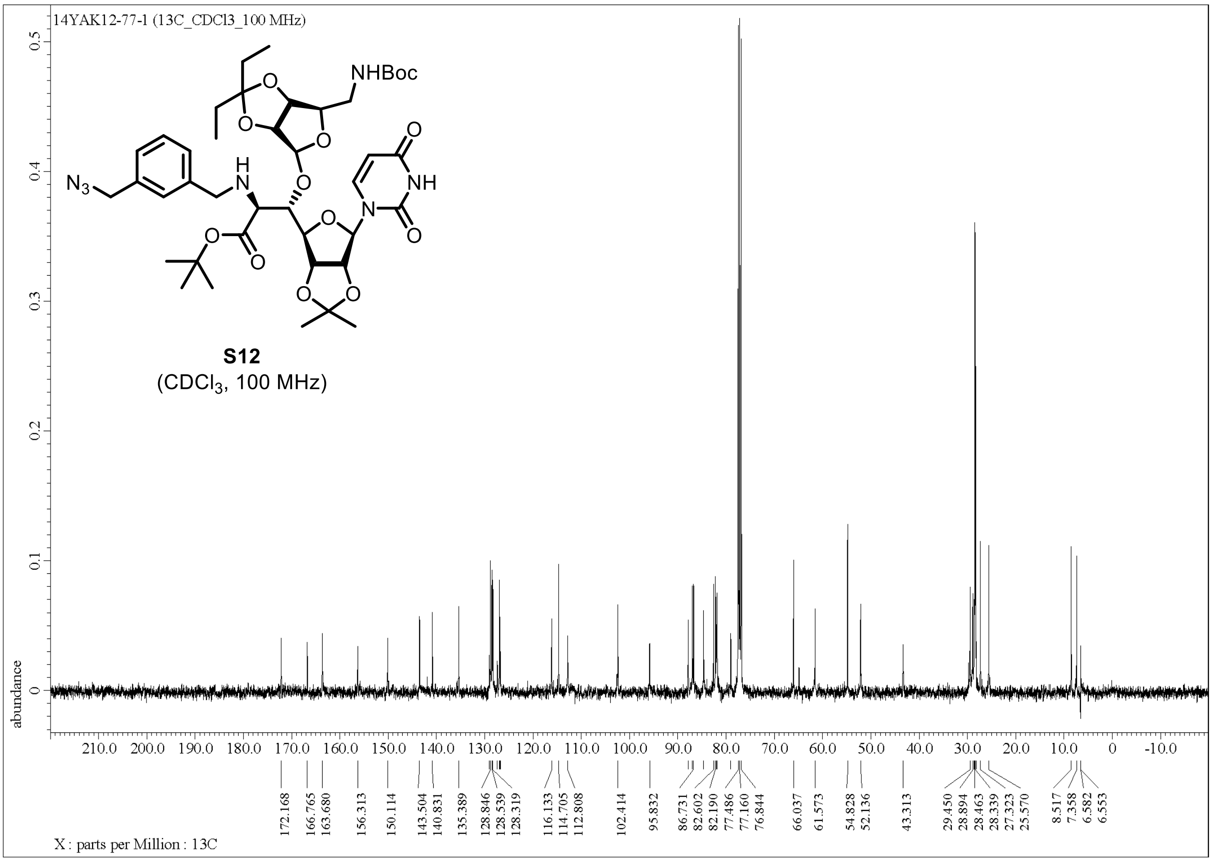

Supplementary Fig. 58. ^{13}C NMR spectrum of compound **S12**.

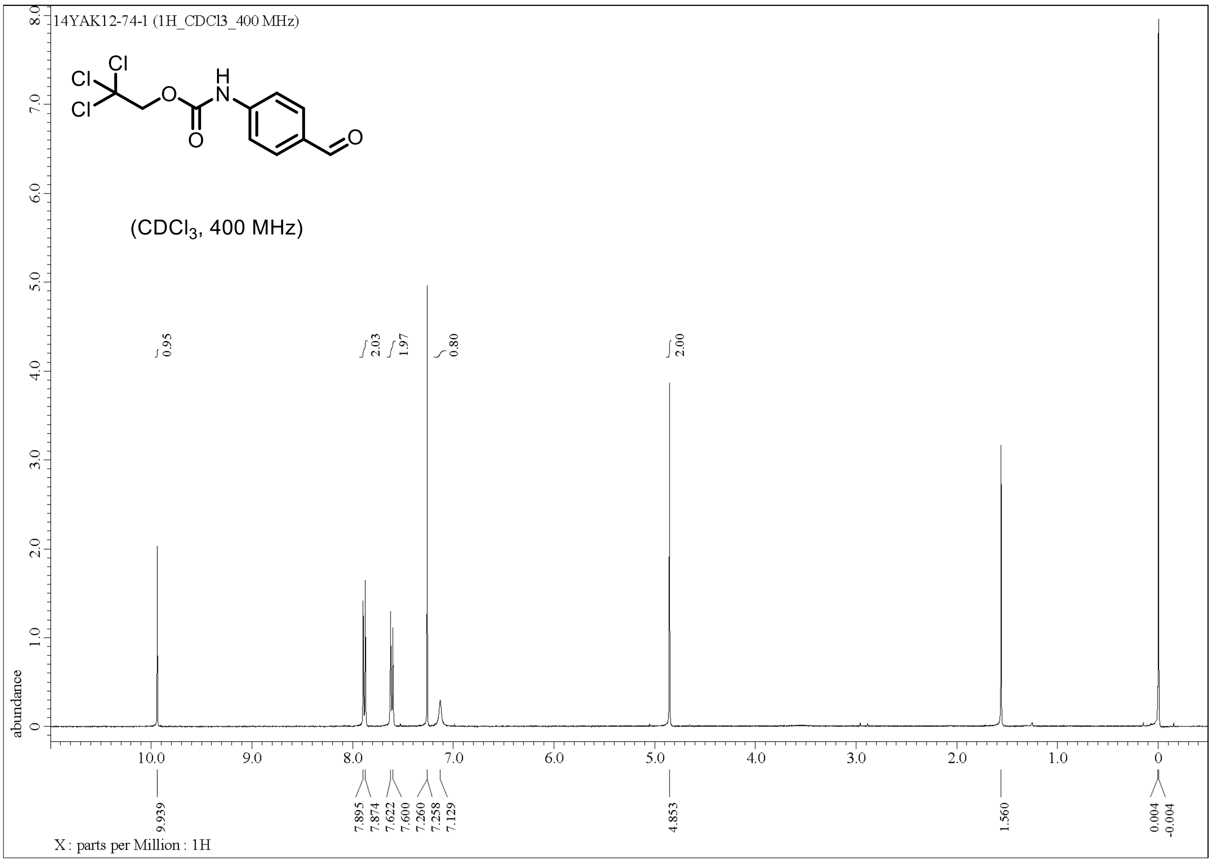

Supplementary Fig. 59. ^{1}H NMR spectrum of 2,2,2-trichloroethyl *N*-(4-formylphenyl)carbamate.

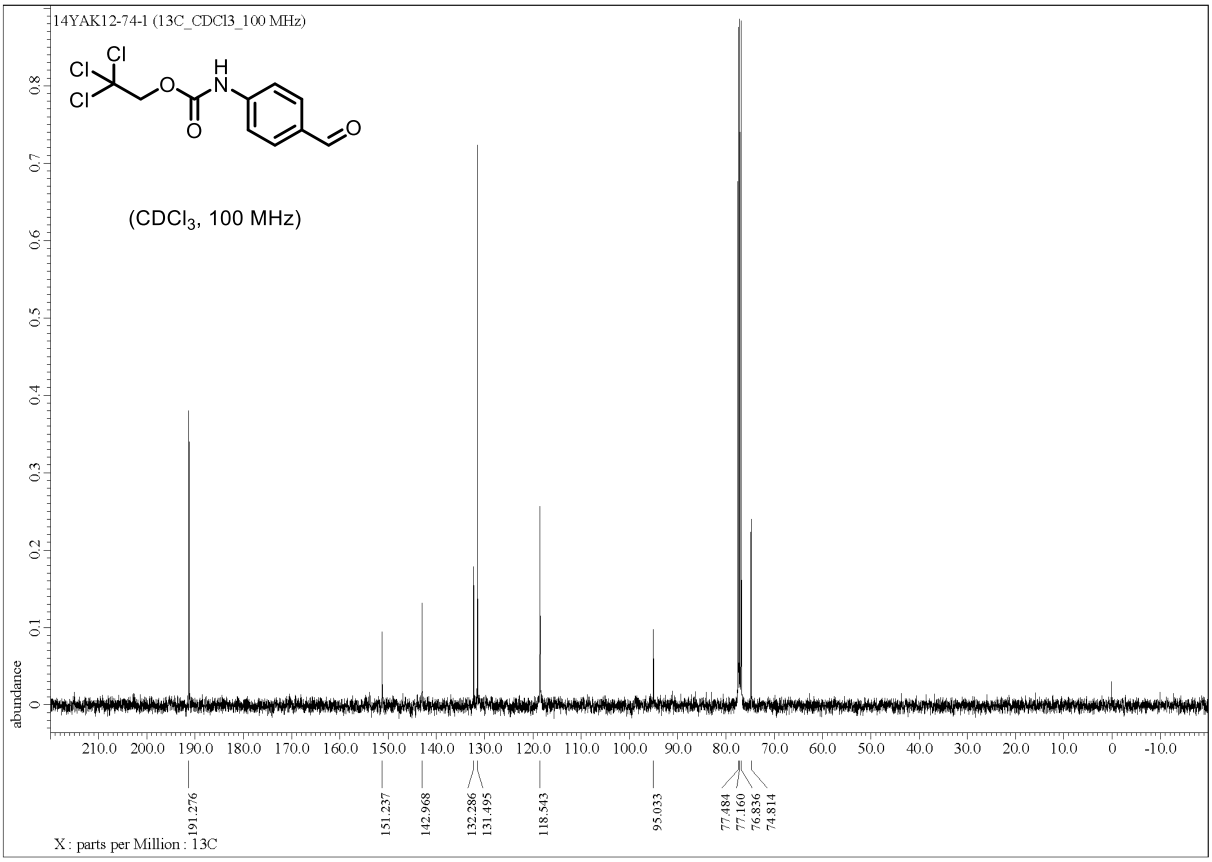

Supplementary Fig. 60. ^{13}C NMR spectrum of 2,2,2-trichloroethyl *N*-(4-formylphenyl)carbamate.

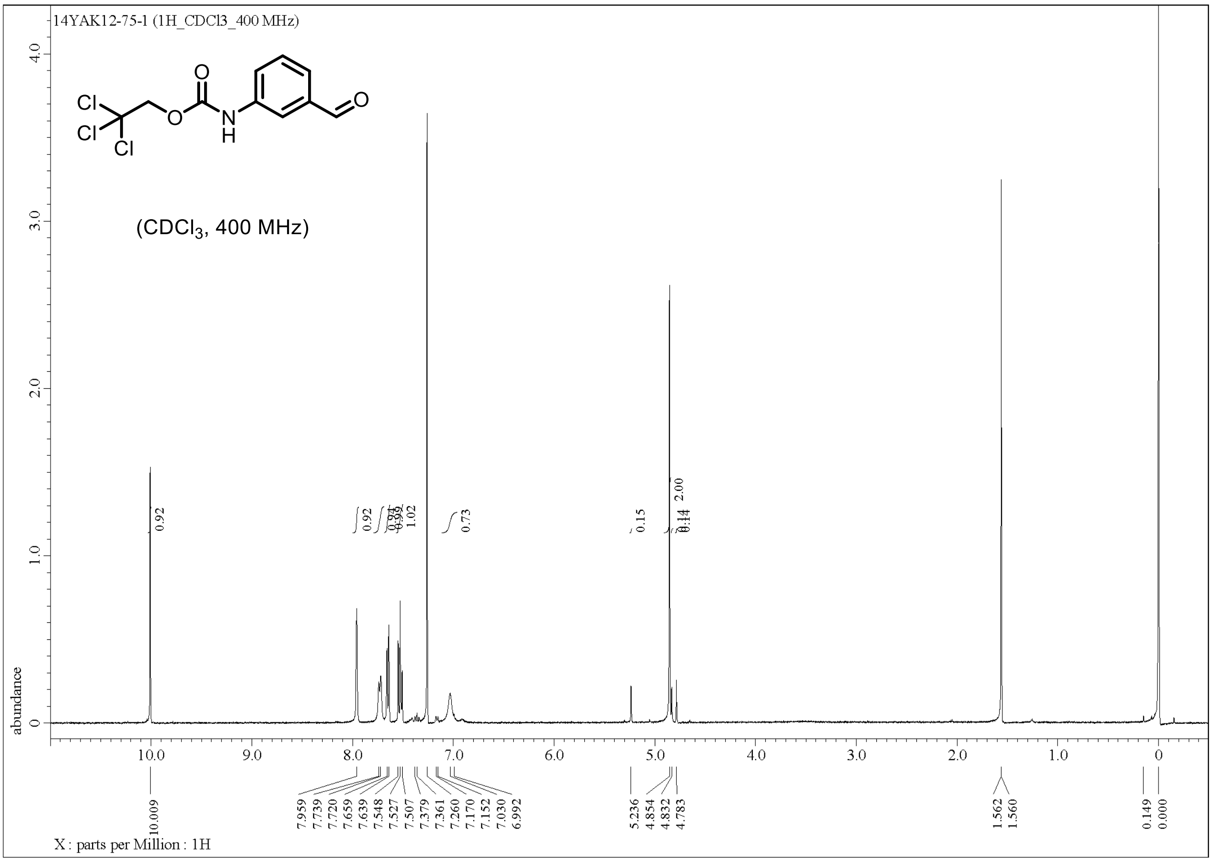

Supplementary Fig. 61. ^{1}H NMR spectrum of 2,2,2-trichloroethyl *N*-(3-formylphenyl)carbamate.

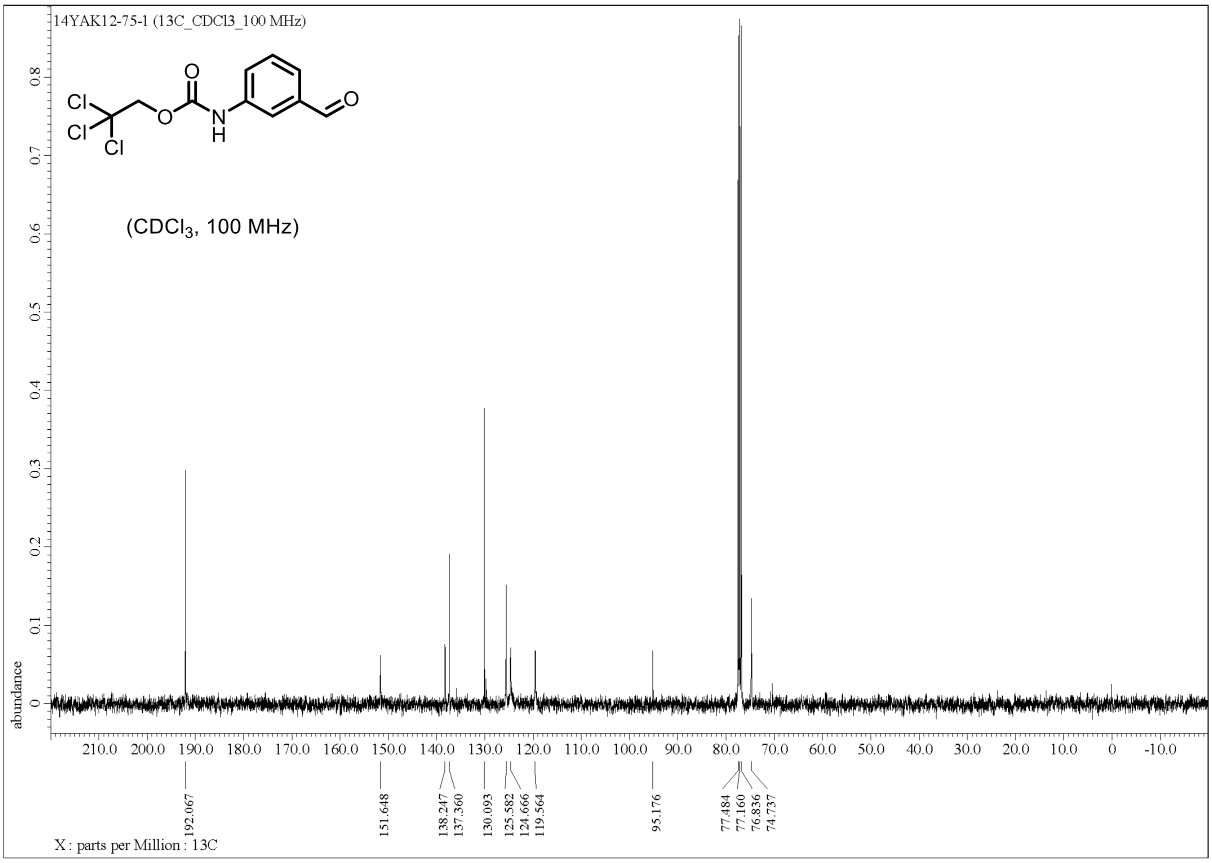

Supplementary Fig. 62. ^{13}C NMR spectrum of 2,2,2-trichloroethyl *N*-(3-formylphenyl)carbamate.

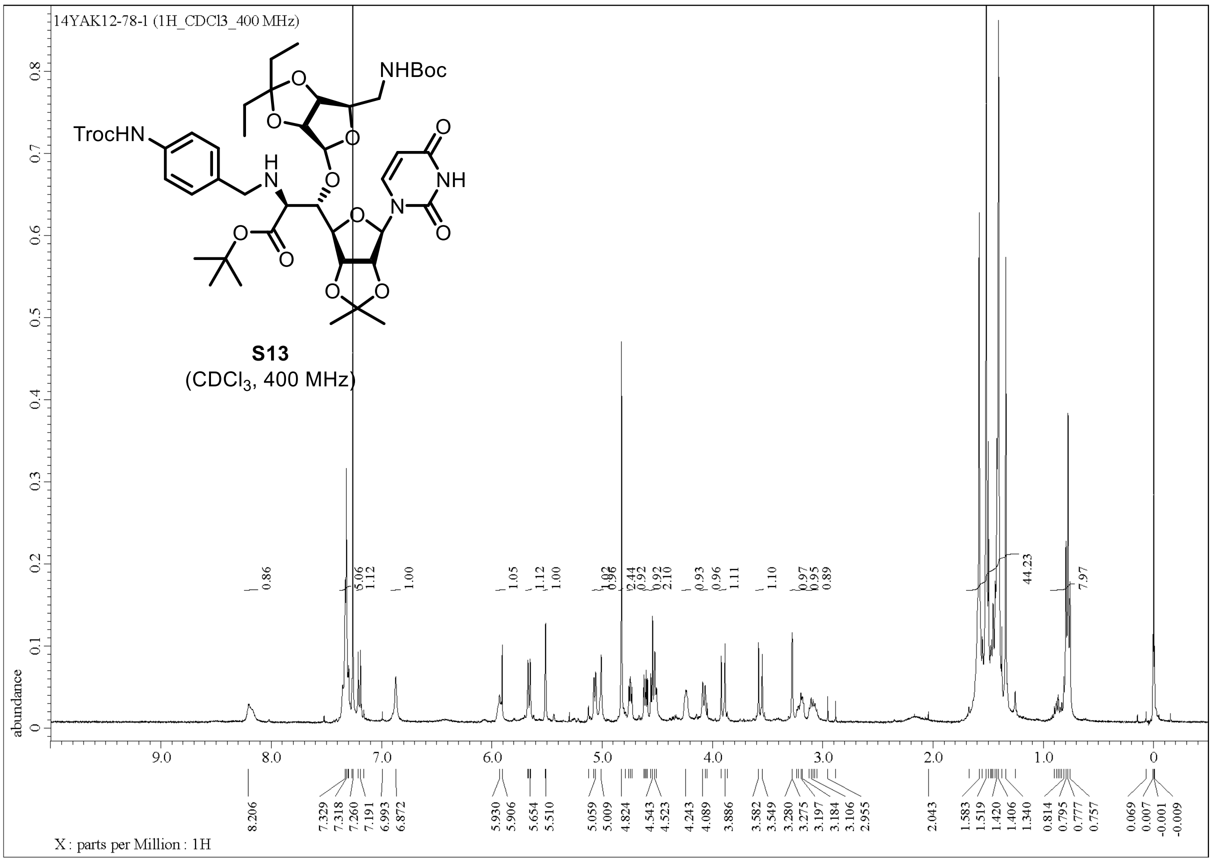

Supplementary Fig. 63. ^{1}H NMR spectrum of compound **S13**.

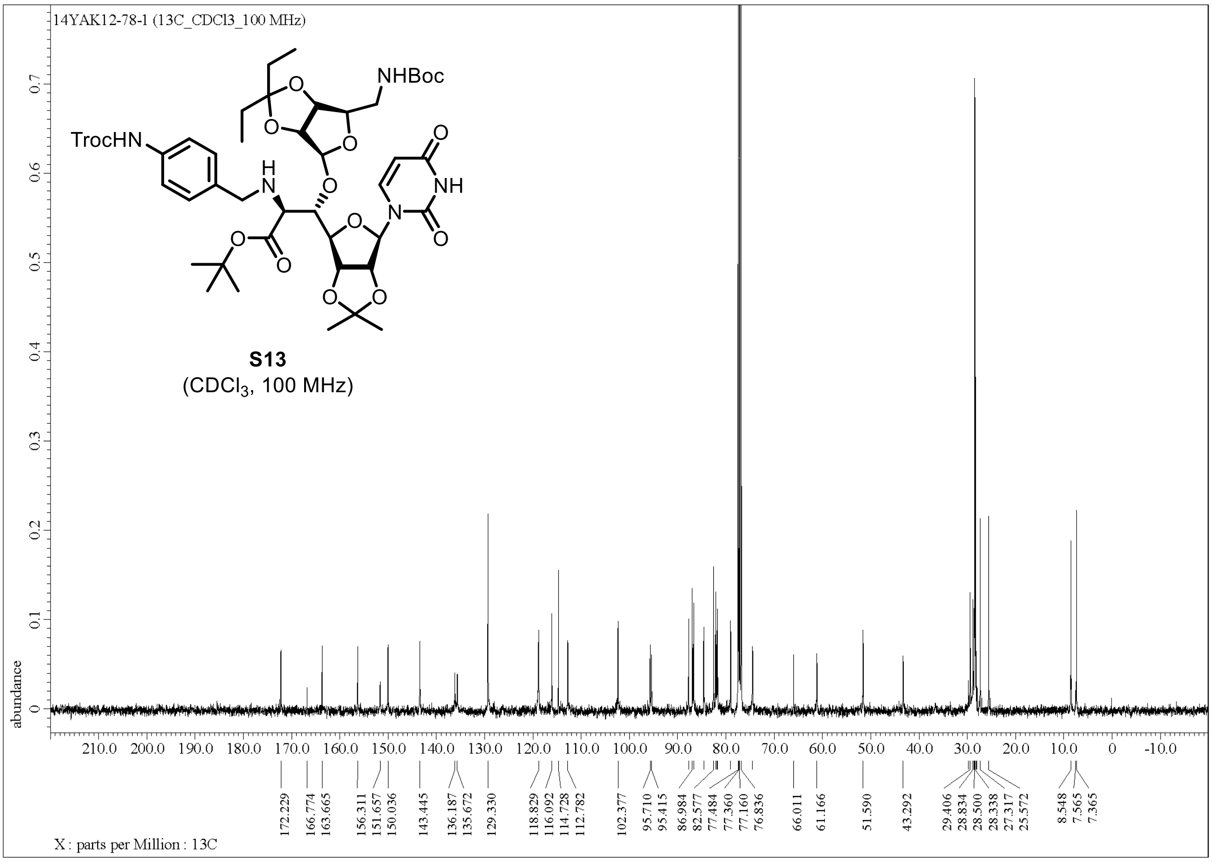

Supplementary Fig. 64. ^{13}C NMR spectrum of compound **S13**.

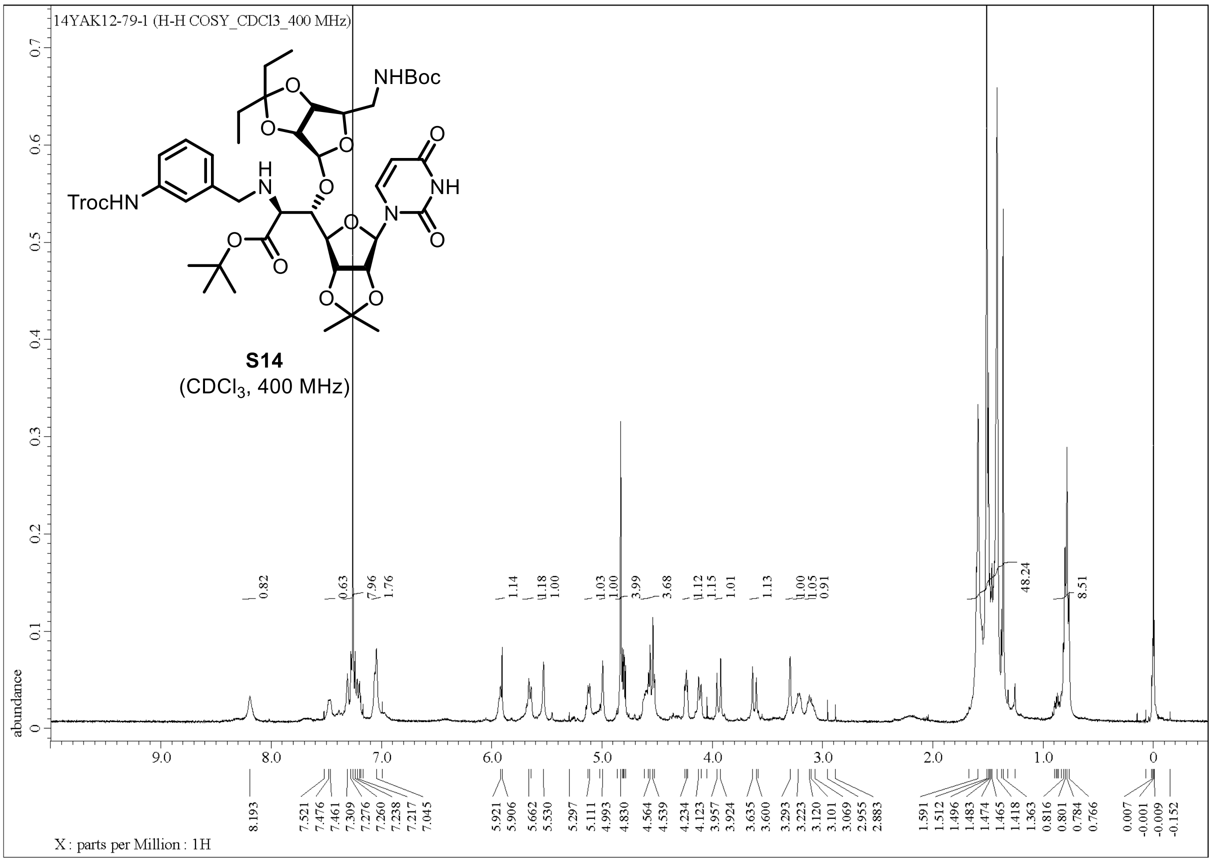

Supplementary Fig. 65. ^{1}H NMR spectrum of compound **S14**.

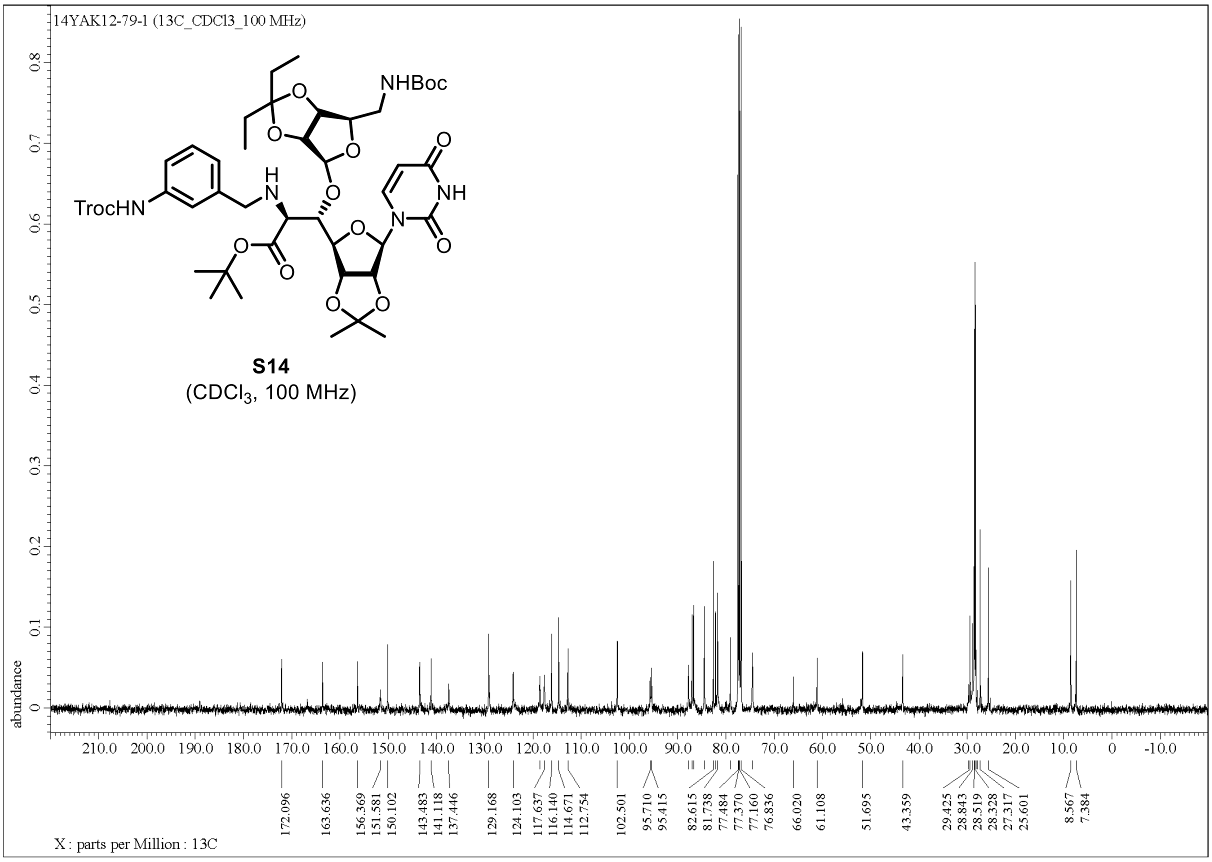

Supplementary Fig. 66. ^{13}C NMR spectrum of compound **S14**.

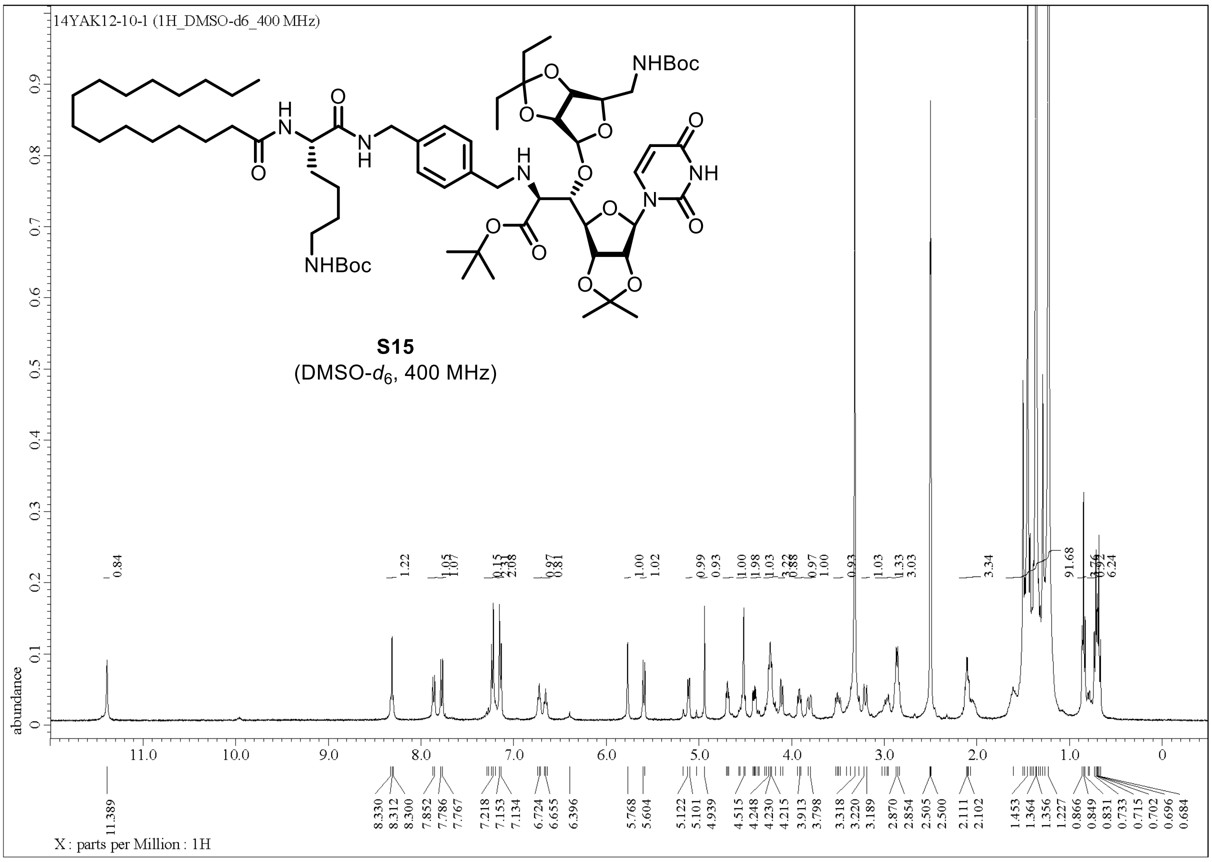

Supplementary Fig. 67. ^{1}H NMR spectrum of compound **S15**.

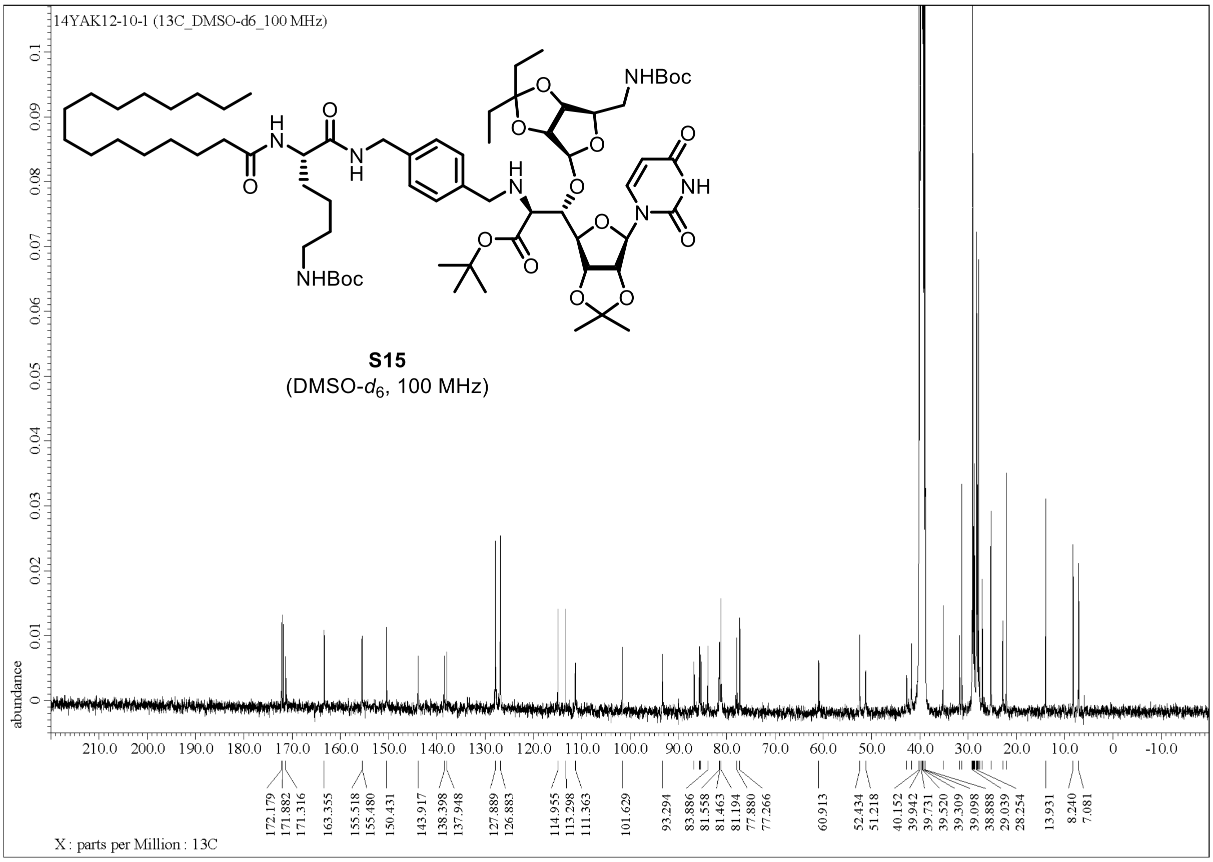

Supplementary Fig. 68. ^{13}C NMR spectrum of compound **S15**.

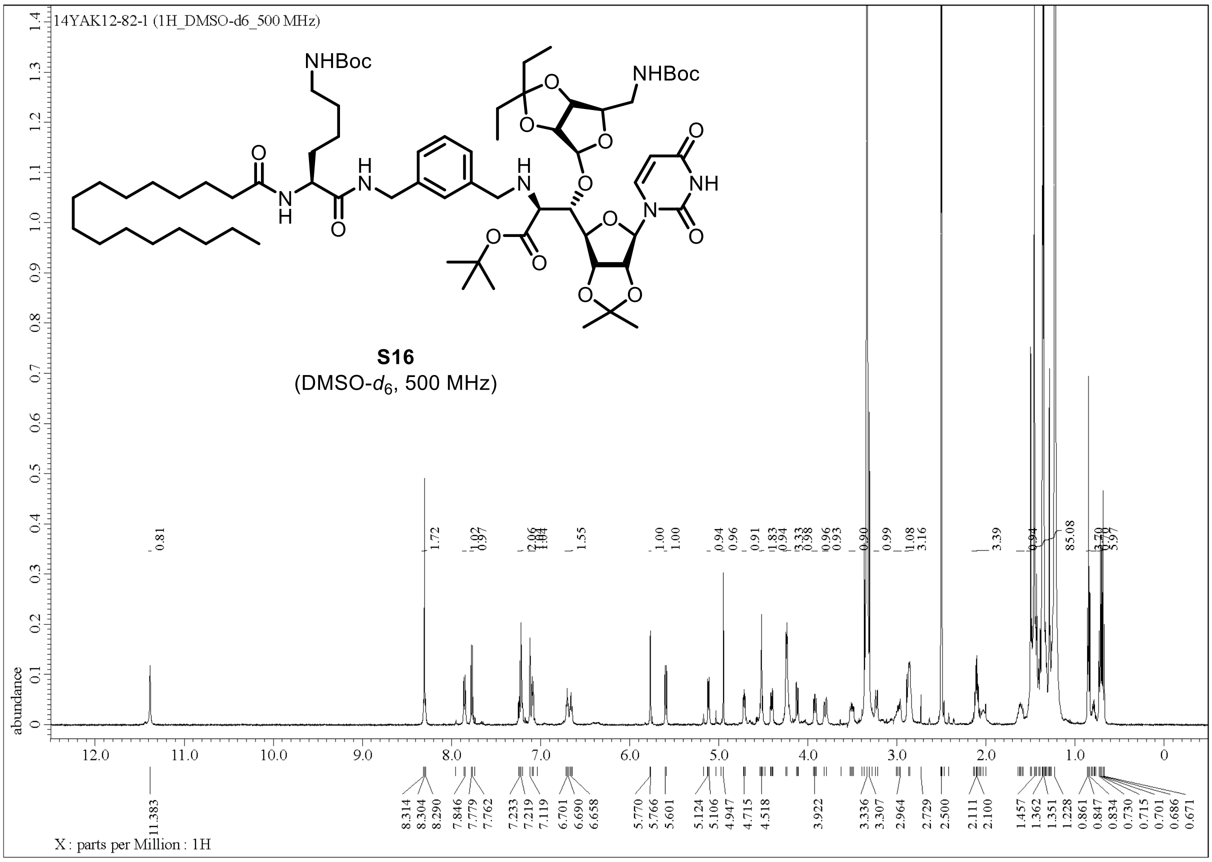

Supplementary Fig. 69. ^{1}H NMR spectrum of compound **S16**.

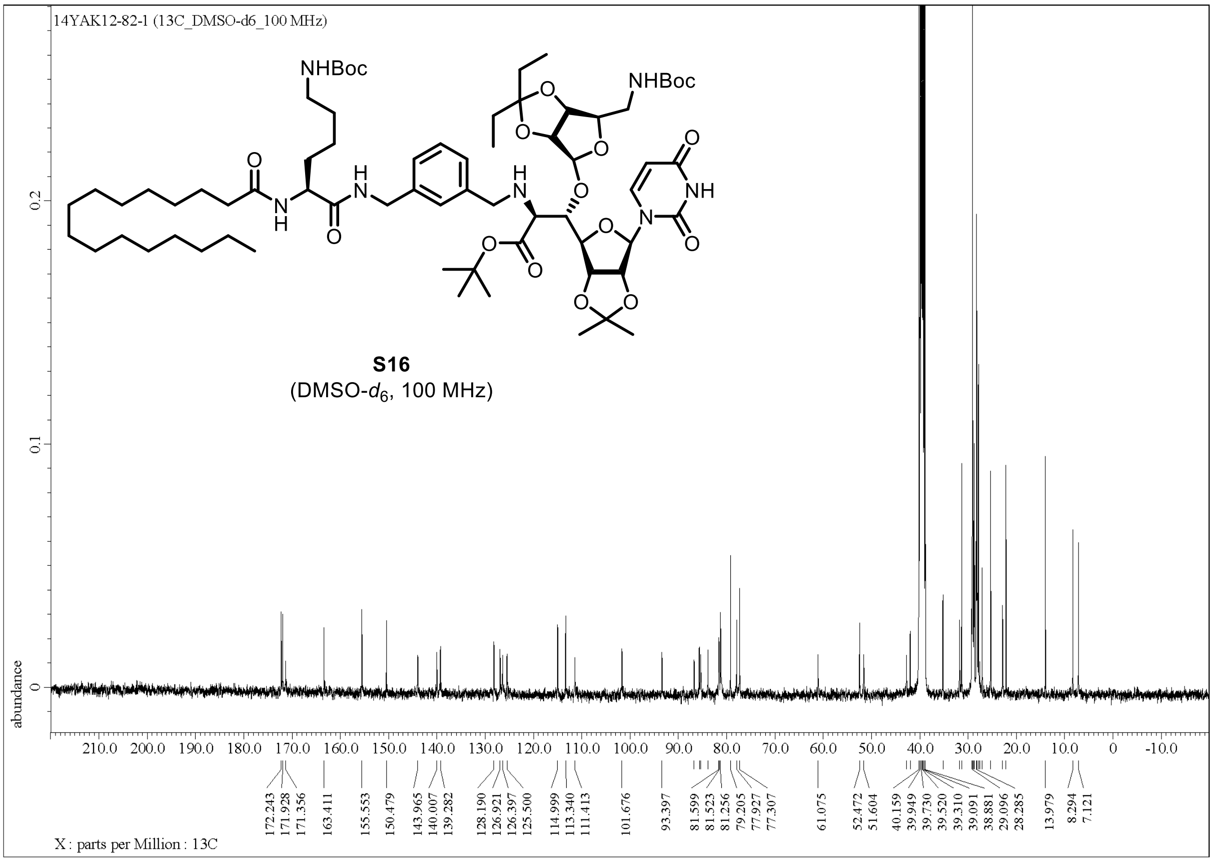

Supplementary Fig. 70. ^{13}C NMR spectrum of compound **S16**.

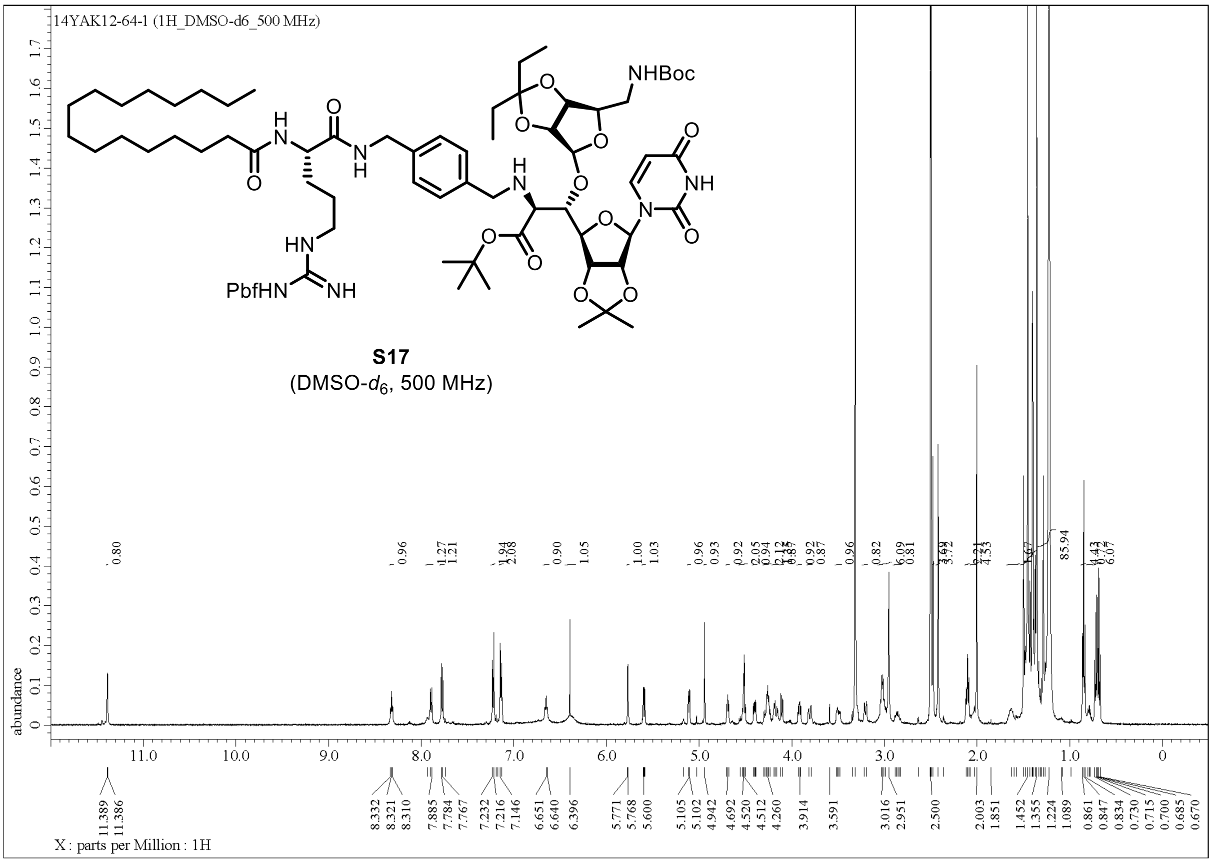

Supplementary Fig. 71. ^{1}H NMR spectrum of compound **S17**.

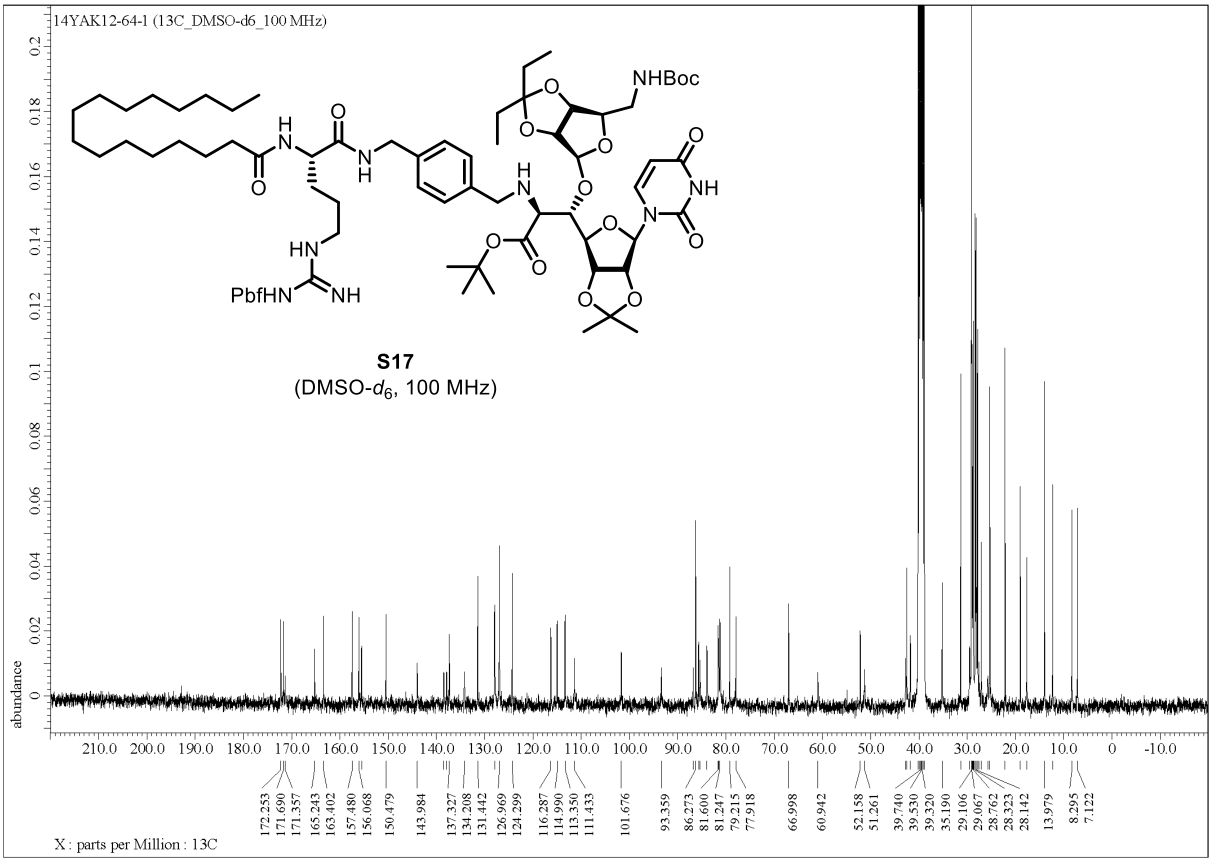

Supplementary Fig. 72. ^{13}C NMR spectrum of compound **S17**.

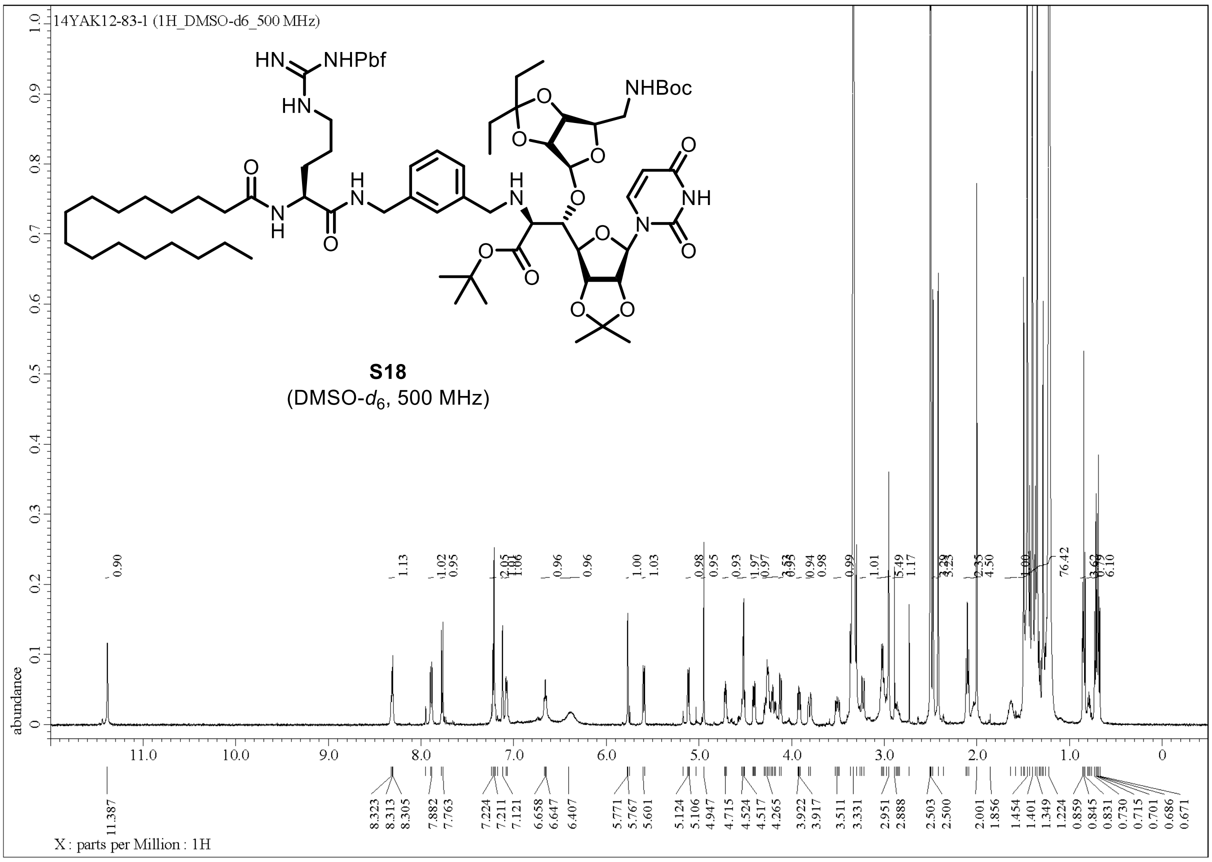

Supplementary Fig. 73. ^{1}H NMR spectrum of compound **S18**.

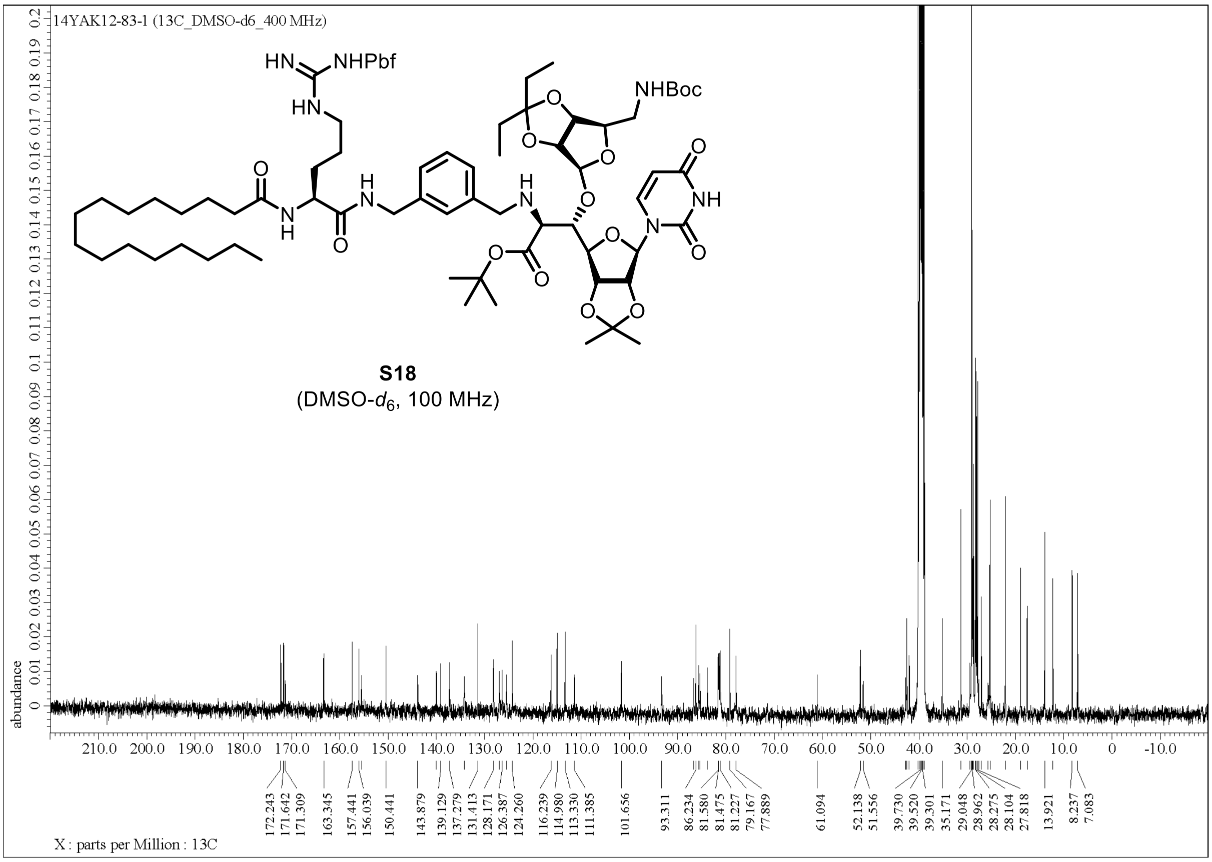

Supplementary Fig. 74. ^{13}C NMR spectrum of compound **S18**.

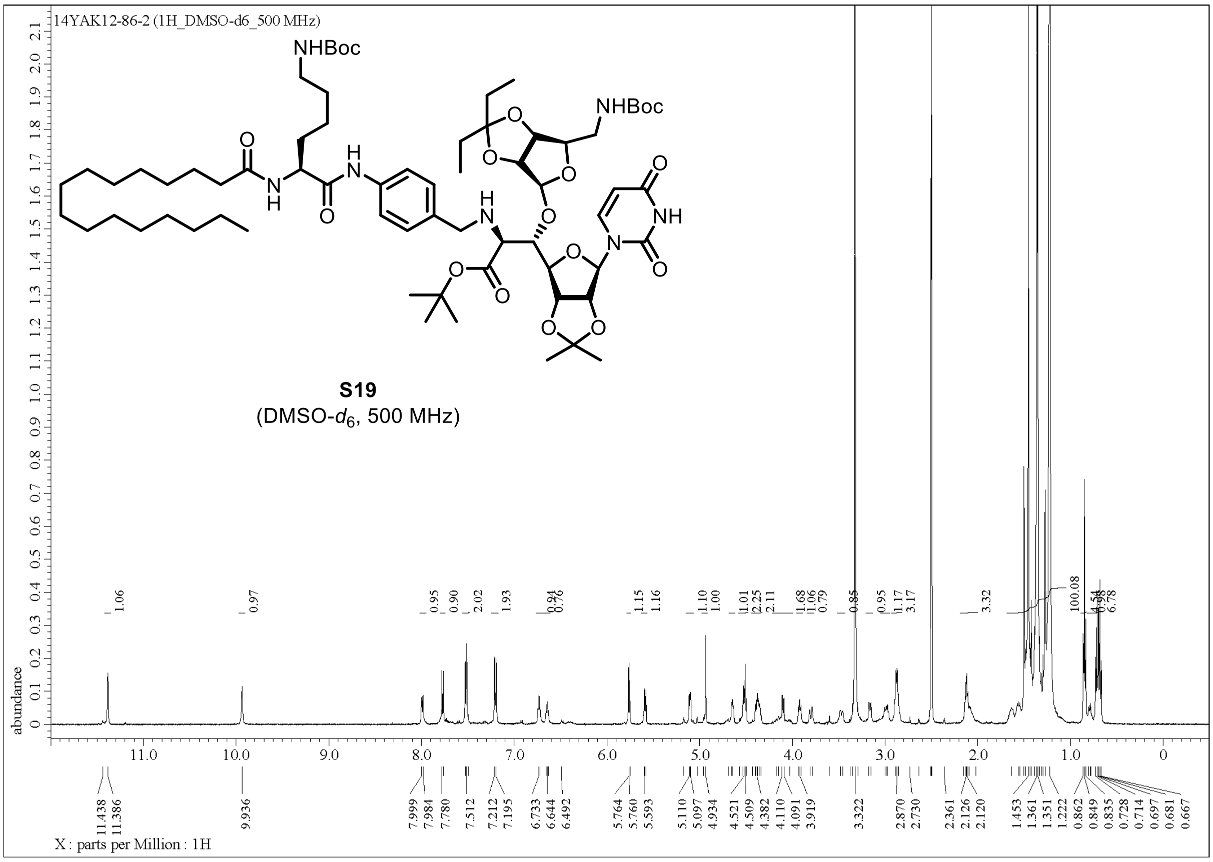

Supplementary Fig. 75. ^{1}H NMR spectrum of compound **S19**.

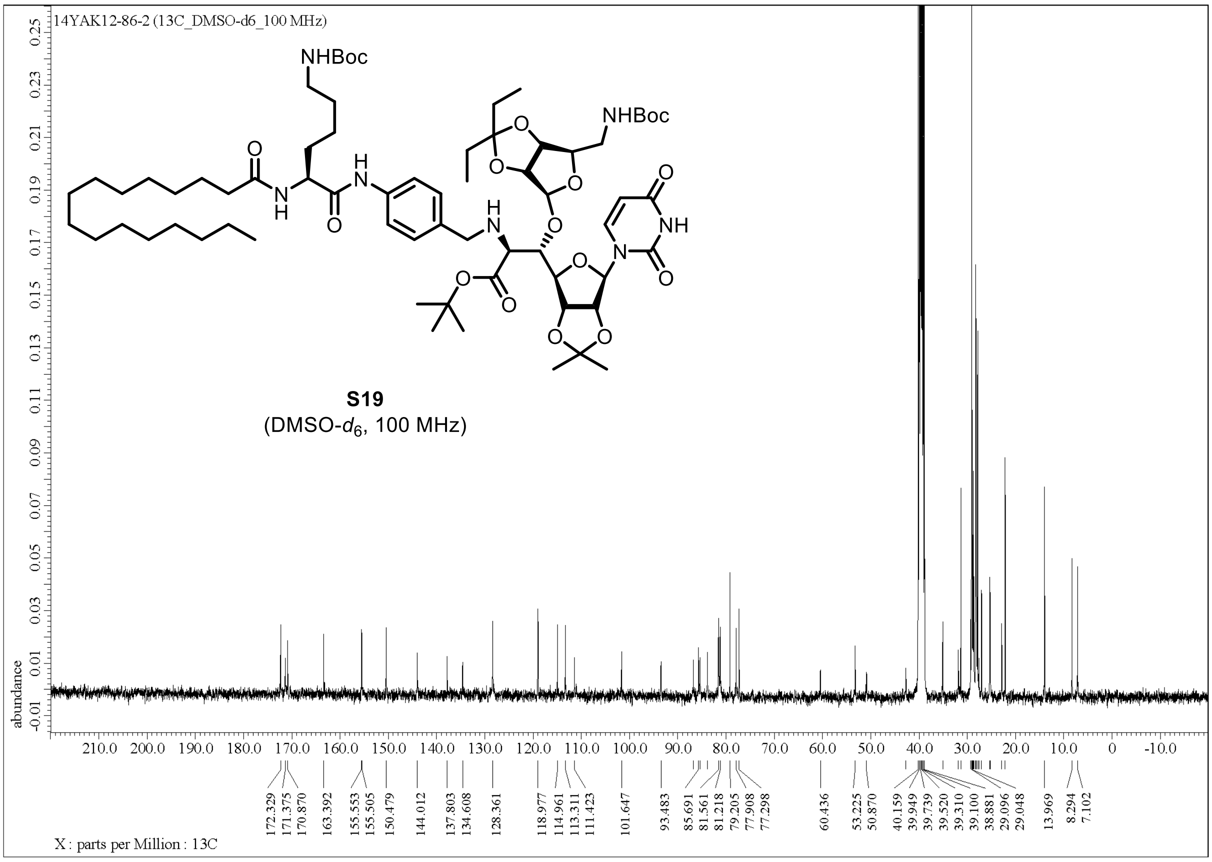

Supplementary Fig. 76. ^{13}C NMR spectrum of compound **S19**.

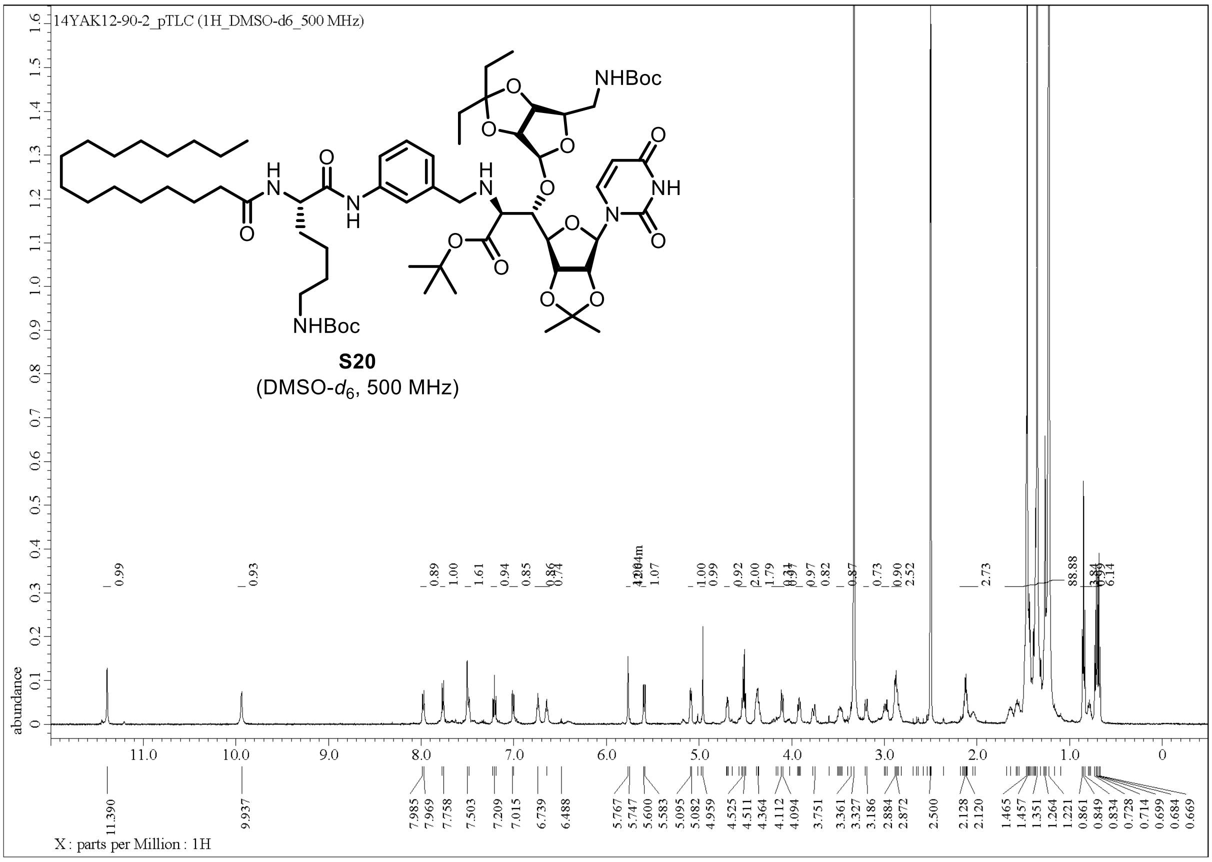

Supplementary Fig. 77. ^{1}H NMR spectrum of compound **S20**.

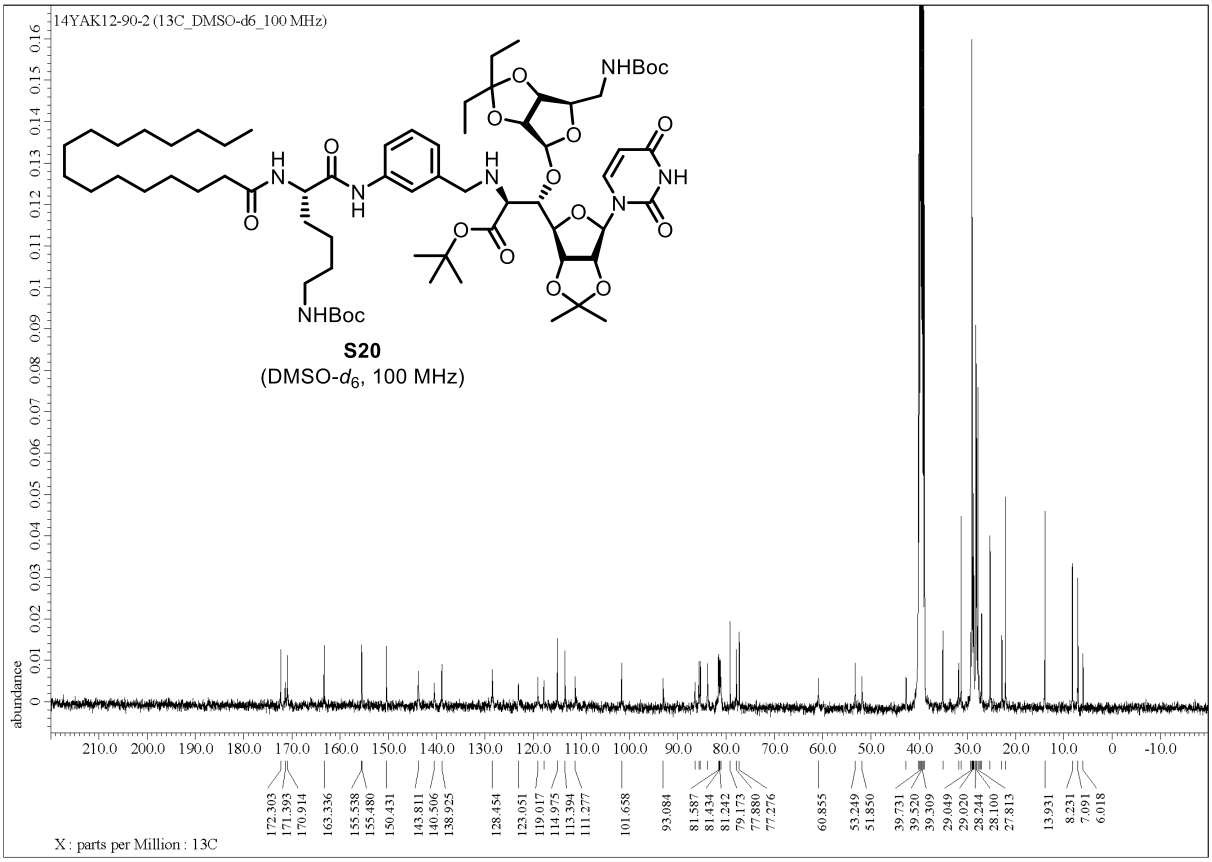

Supplementary Fig. 78. ^{13}C NMR spectrum of compound **S20**.

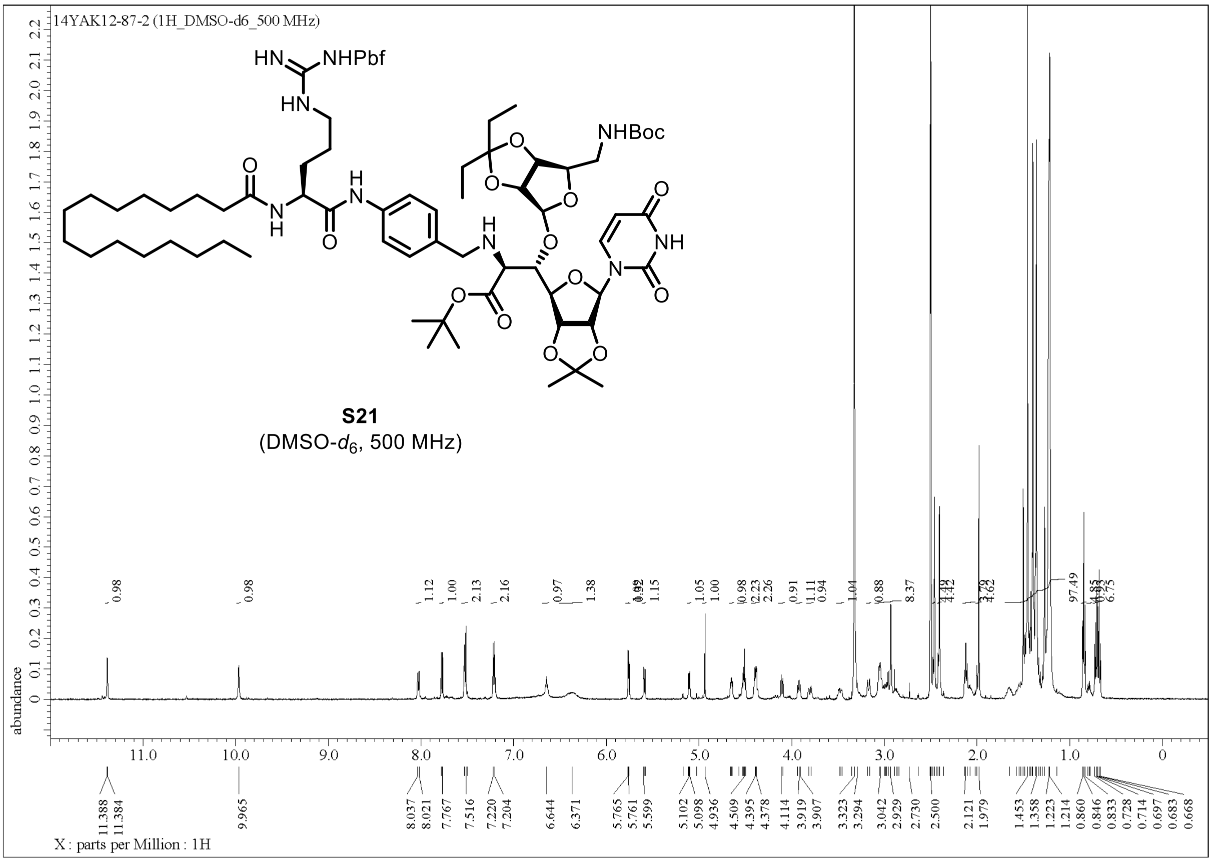

Supplementary Fig. 79. ^{1}H NMR spectrum of compound **S21**.

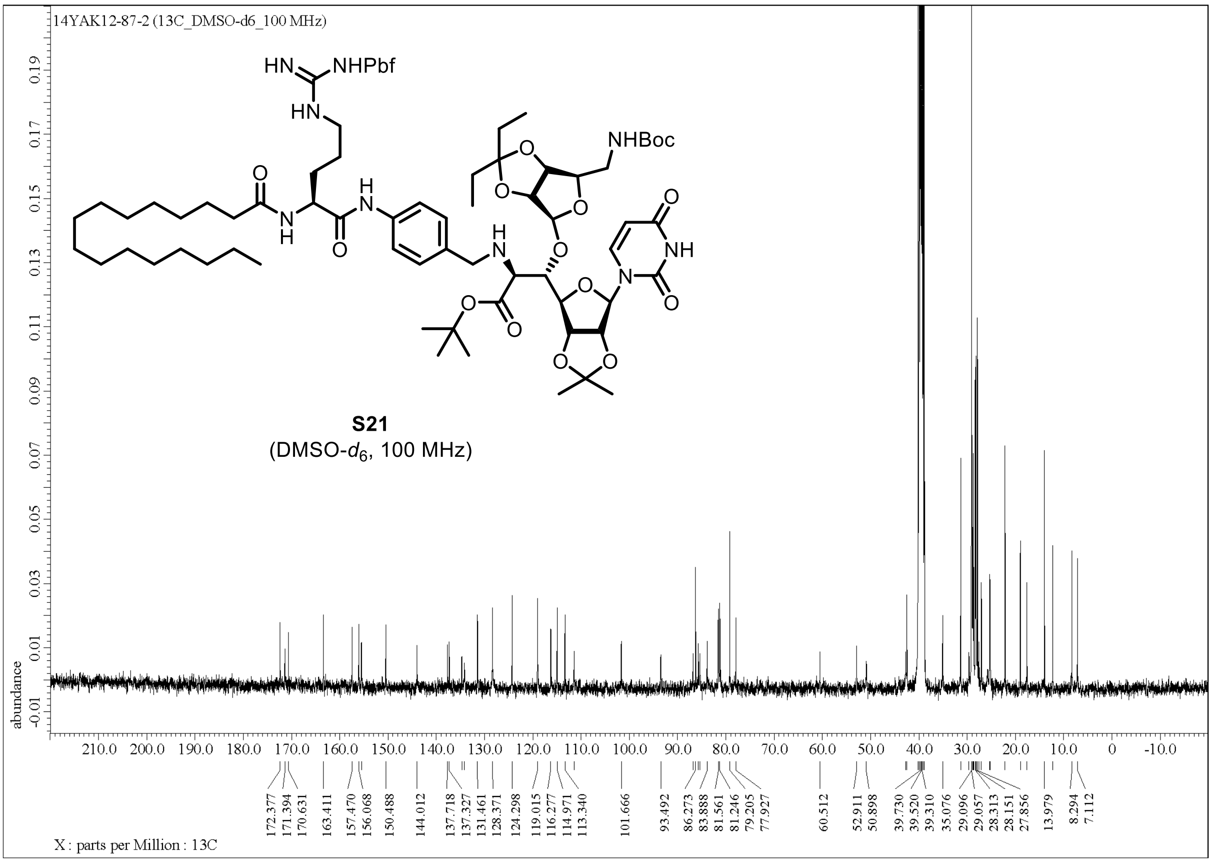

Supplementary Fig. 80. ^{13}C NMR spectrum of compound **S21**.

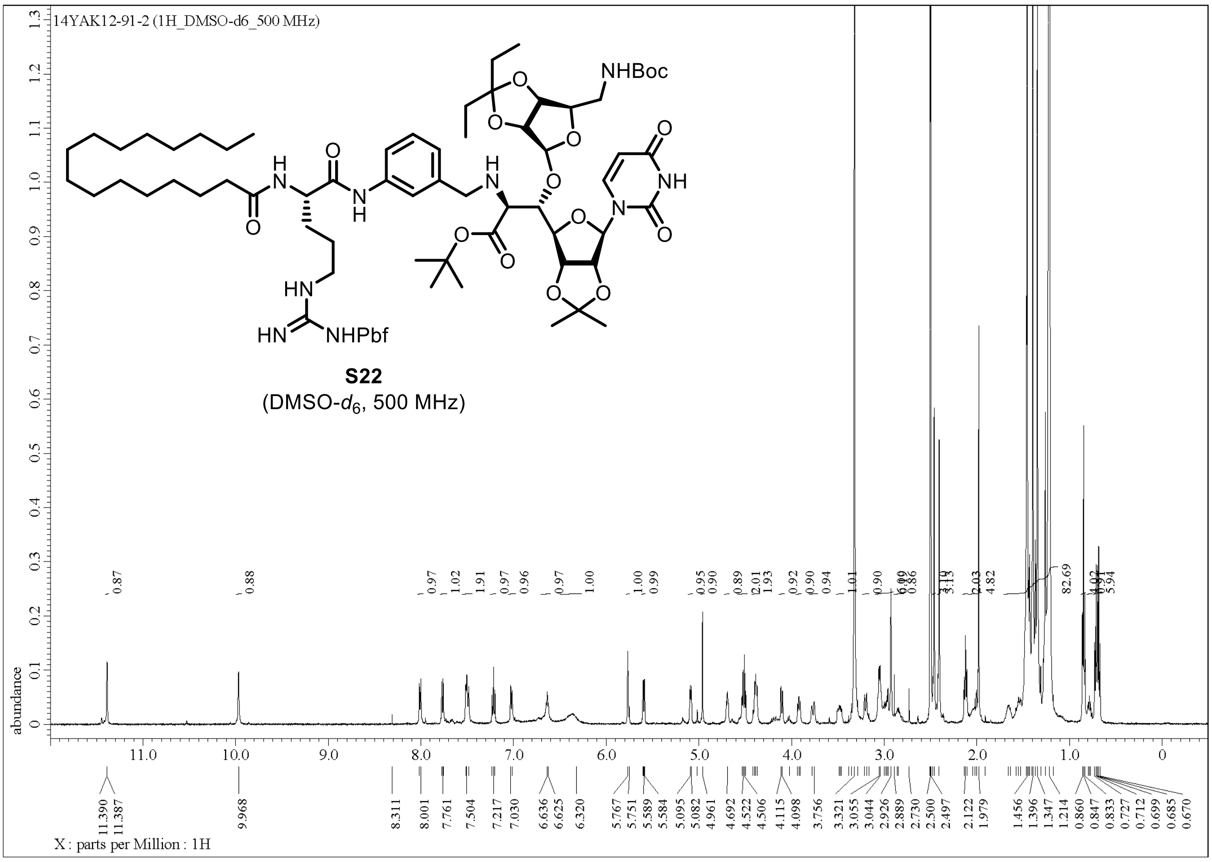

Supplementary Fig. 81. ^{1}H NMR spectrum of compound **S22**.

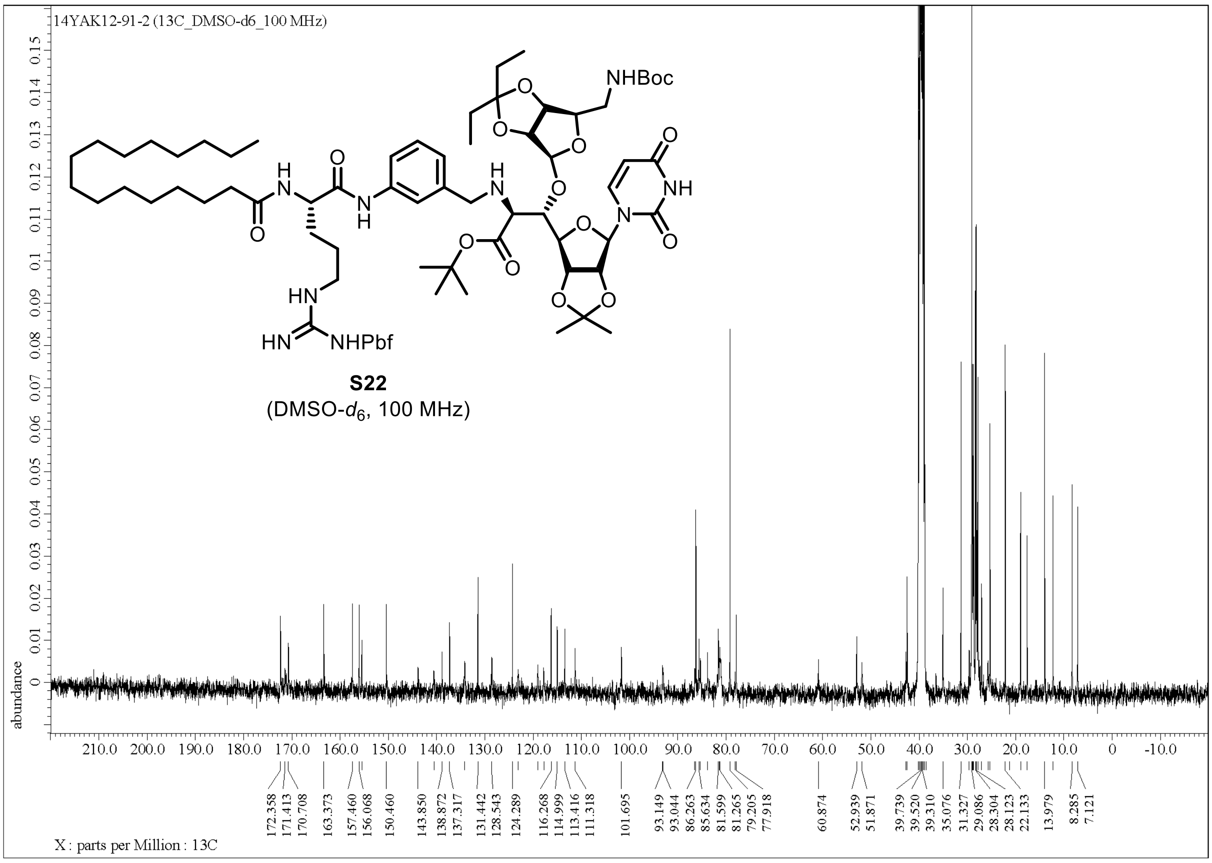

Supplementary Fig. 82. ^{13}C NMR spectrum of compound **S22**.

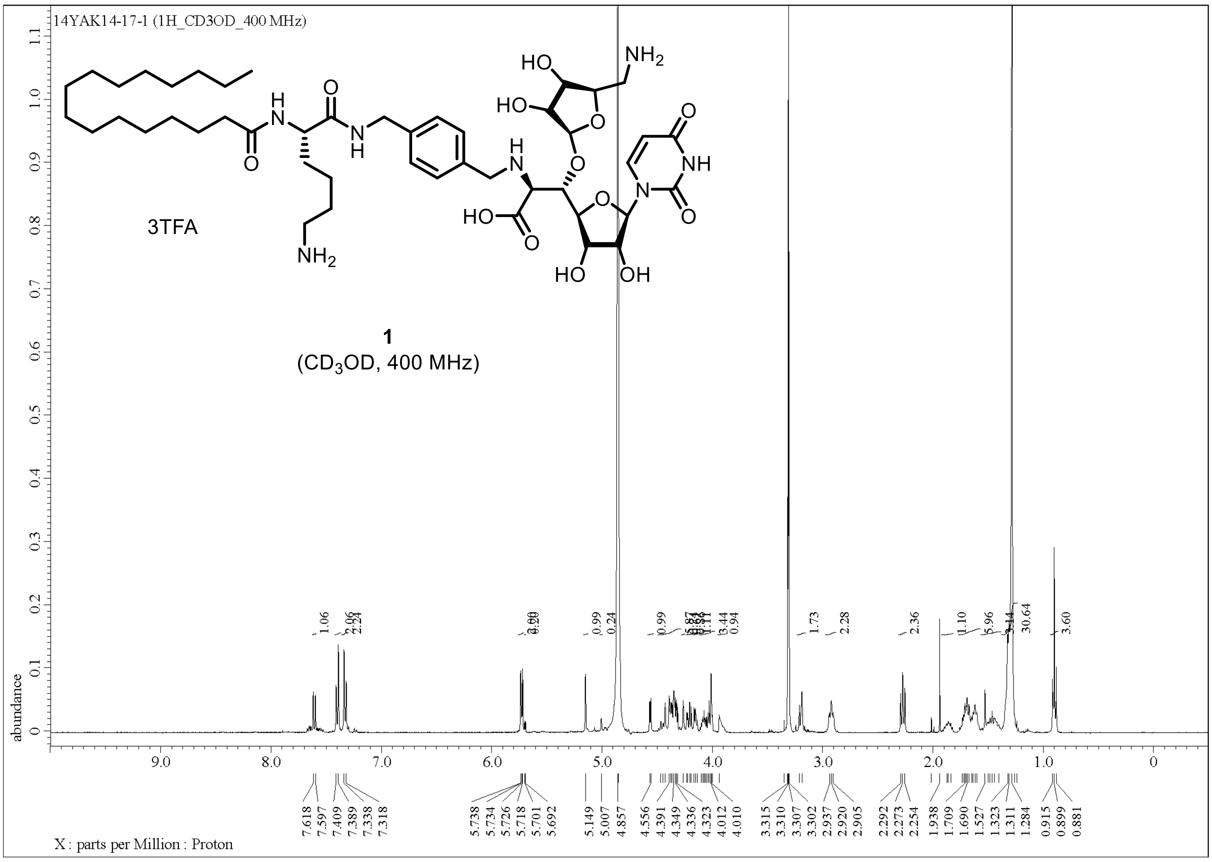

Supplementary Fig. 83. ^{1}H NMR spectrum of compound **1**.

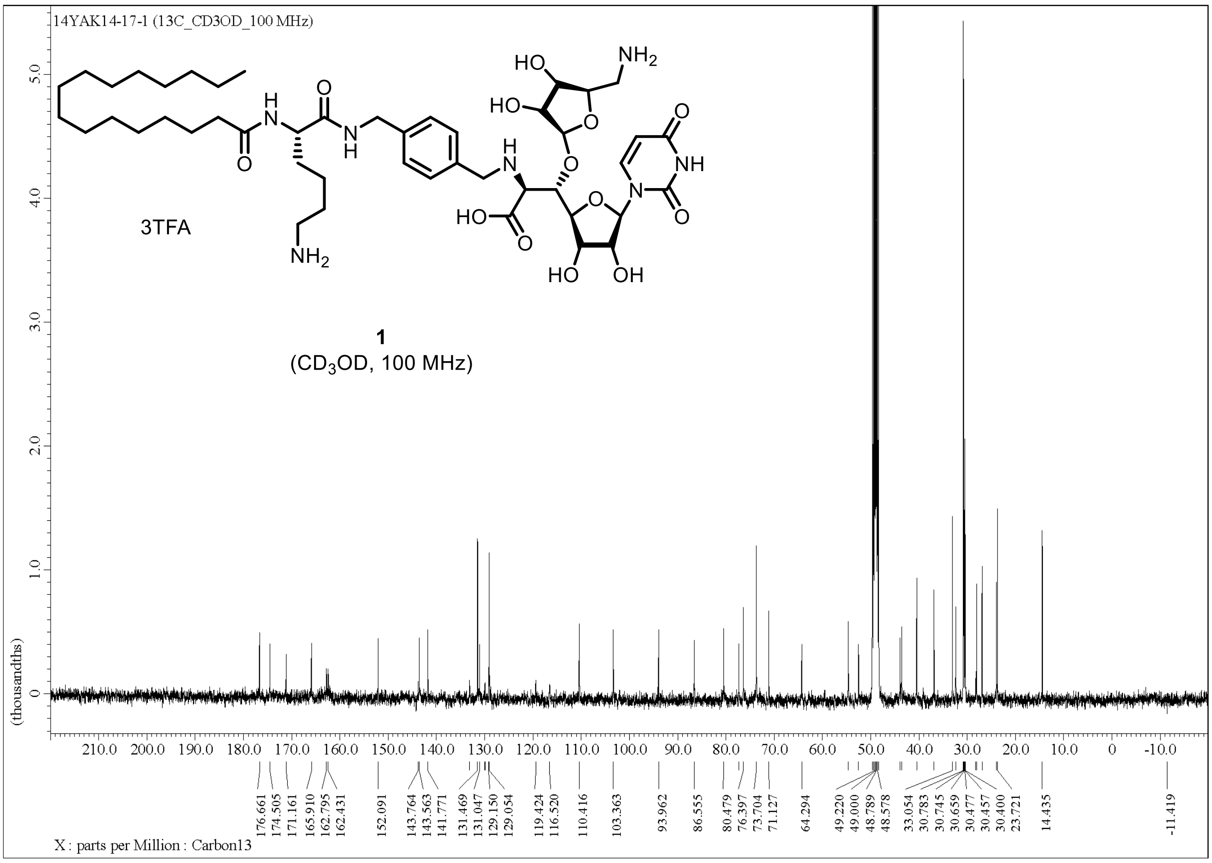

Supplementary Fig. 84. ^{13}C NMR spectrum of compound **1**.

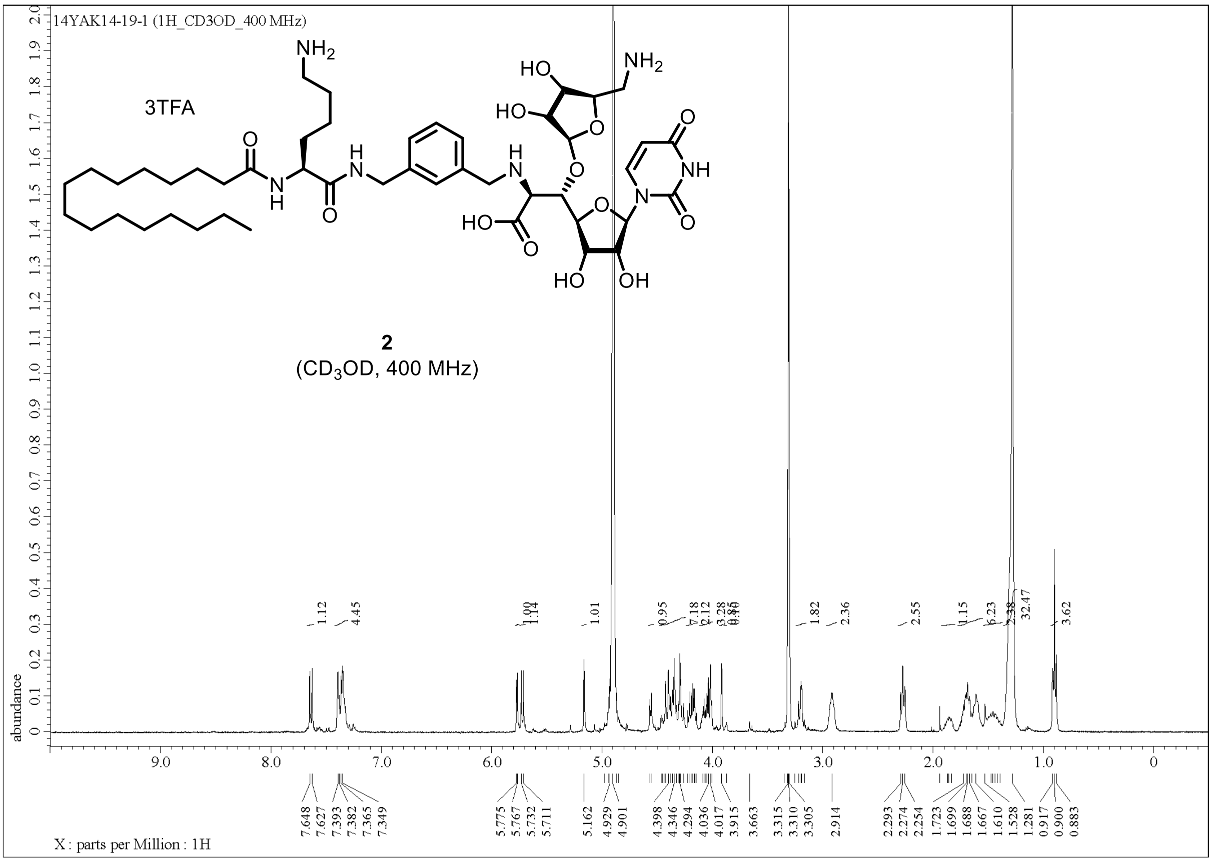

Supplementary Fig. 85. ^{1}H NMR spectrum of compound **2**.

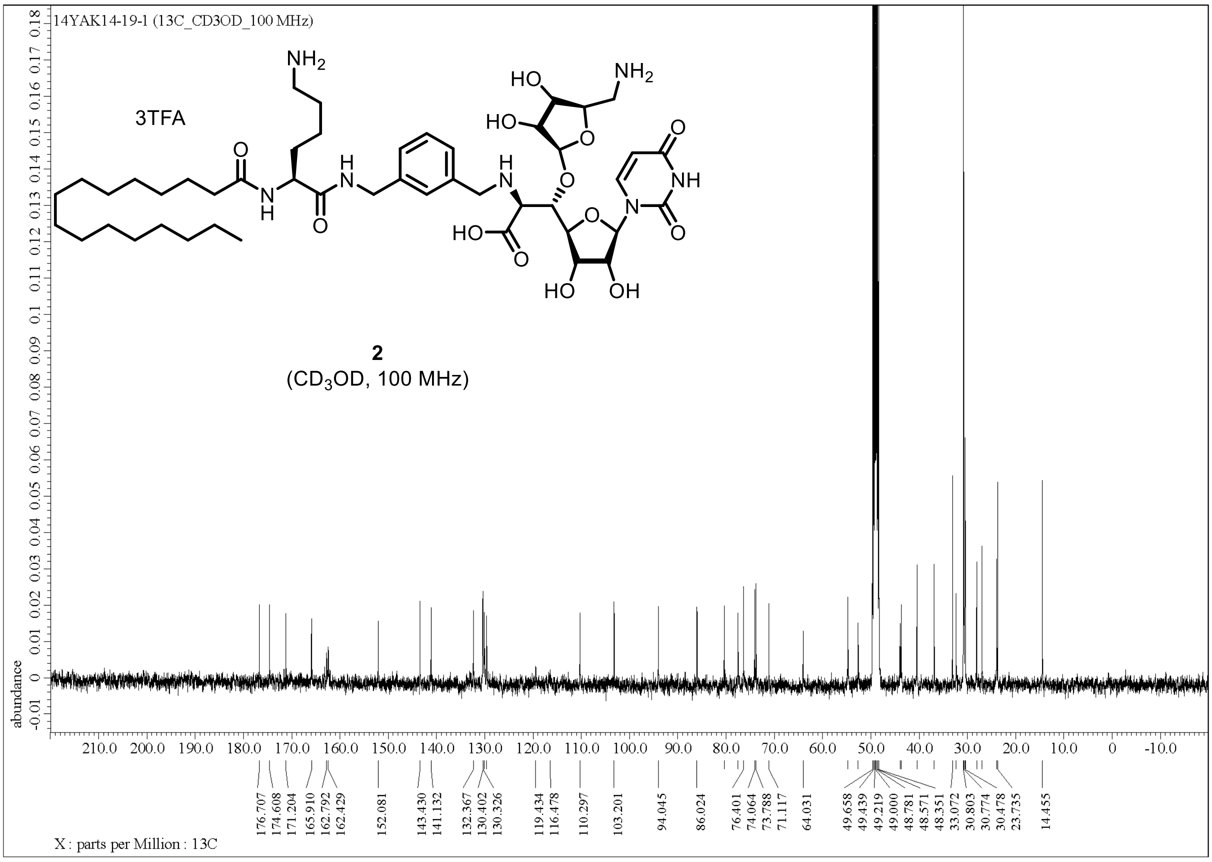

Supplementary Fig. 86. ^{13}C NMR spectrum of compound **2**.

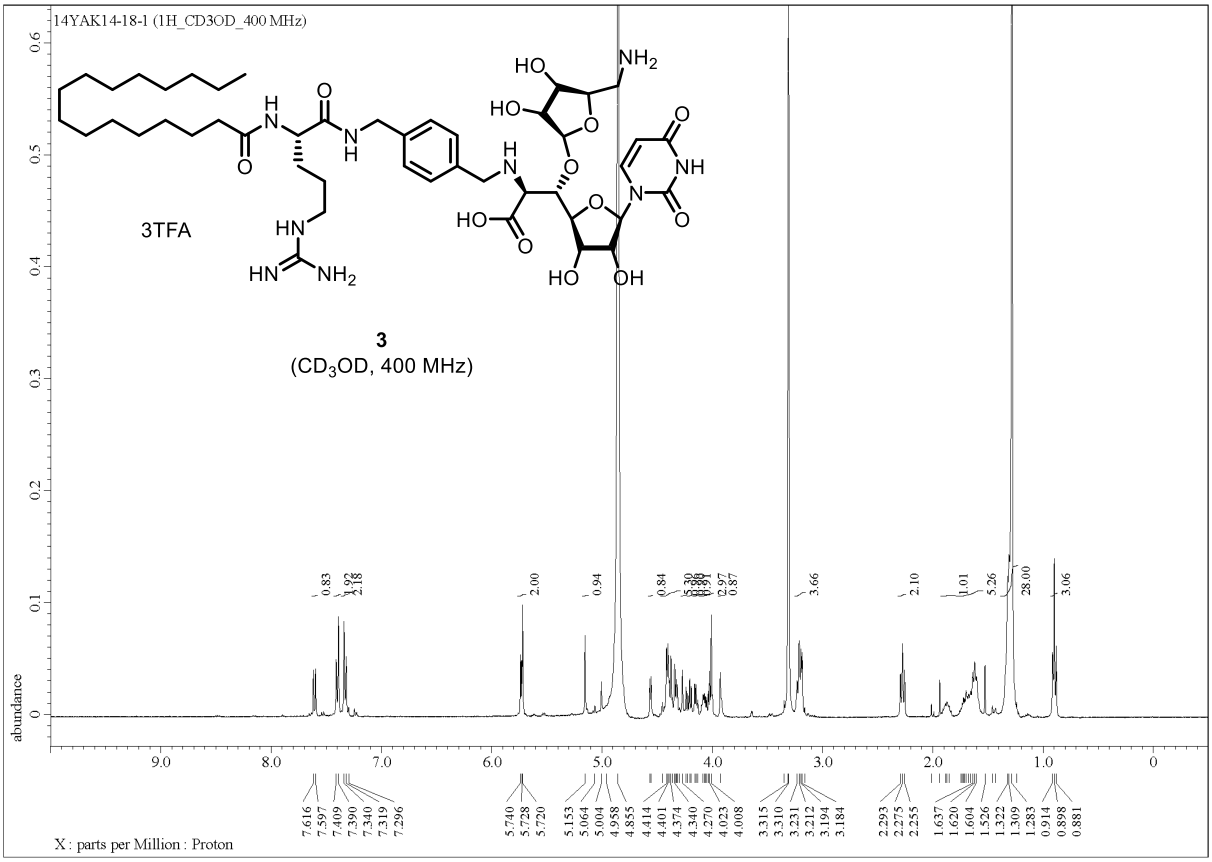

Supplementary Fig. 87. ^{1}H NMR spectrum of compound **3**.

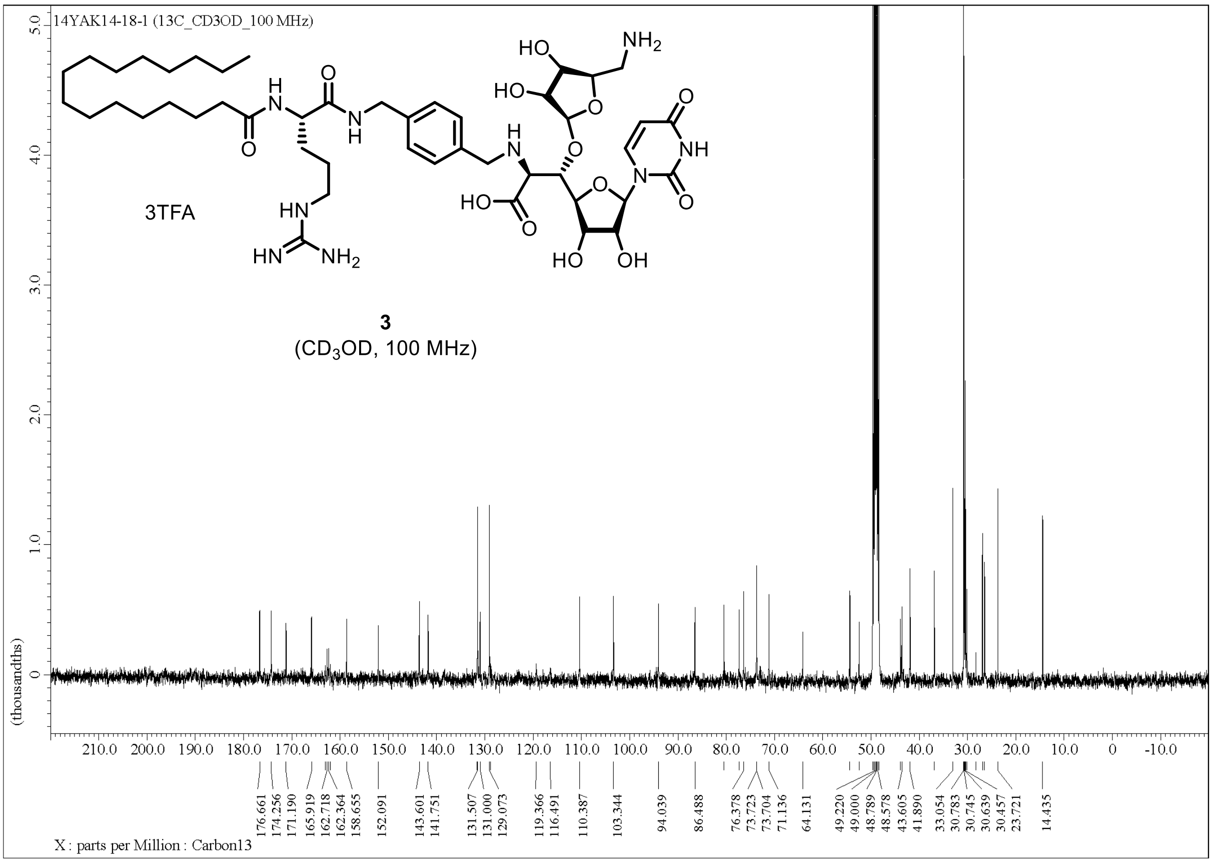

Supplementary Fig. 88. ^{13}C NMR spectrum of compound **3**.

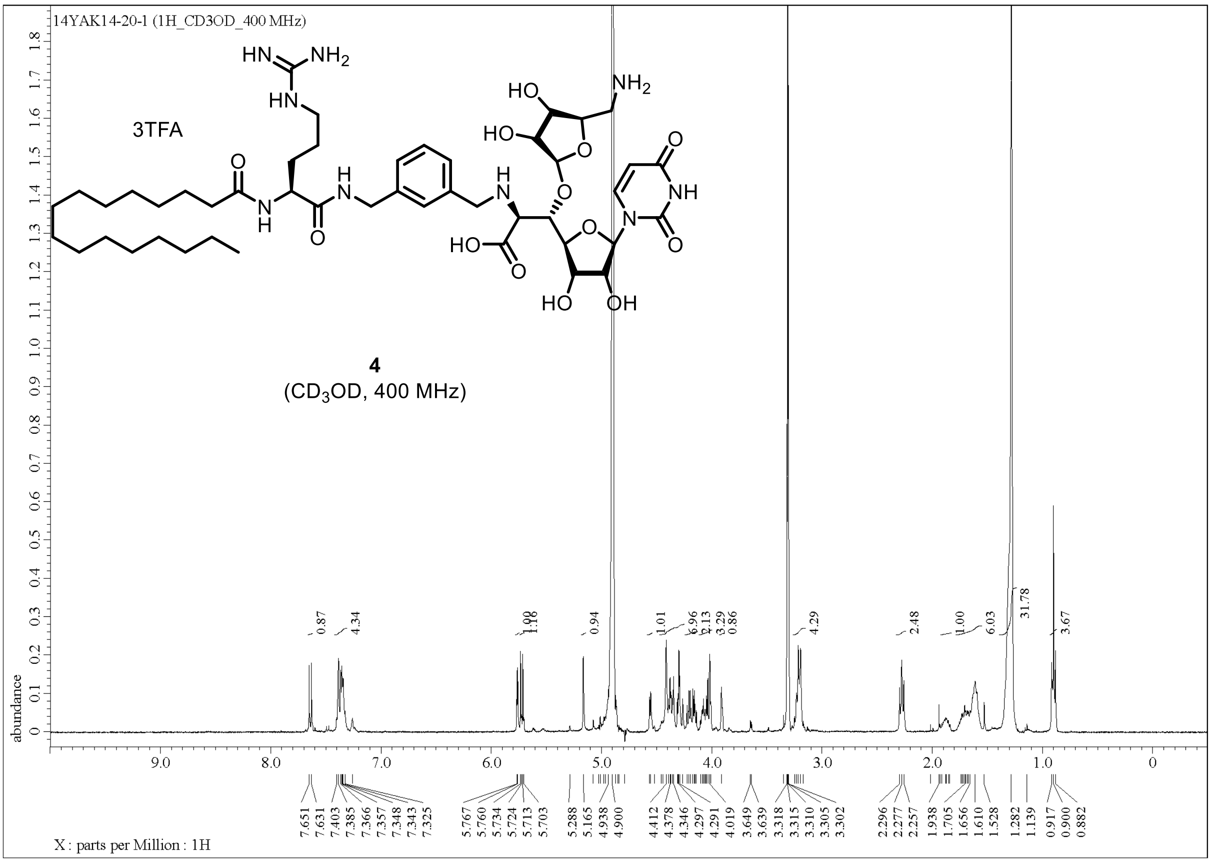

Supplementary Fig. 89. ^{1}H NMR spectrum of compound **4**.

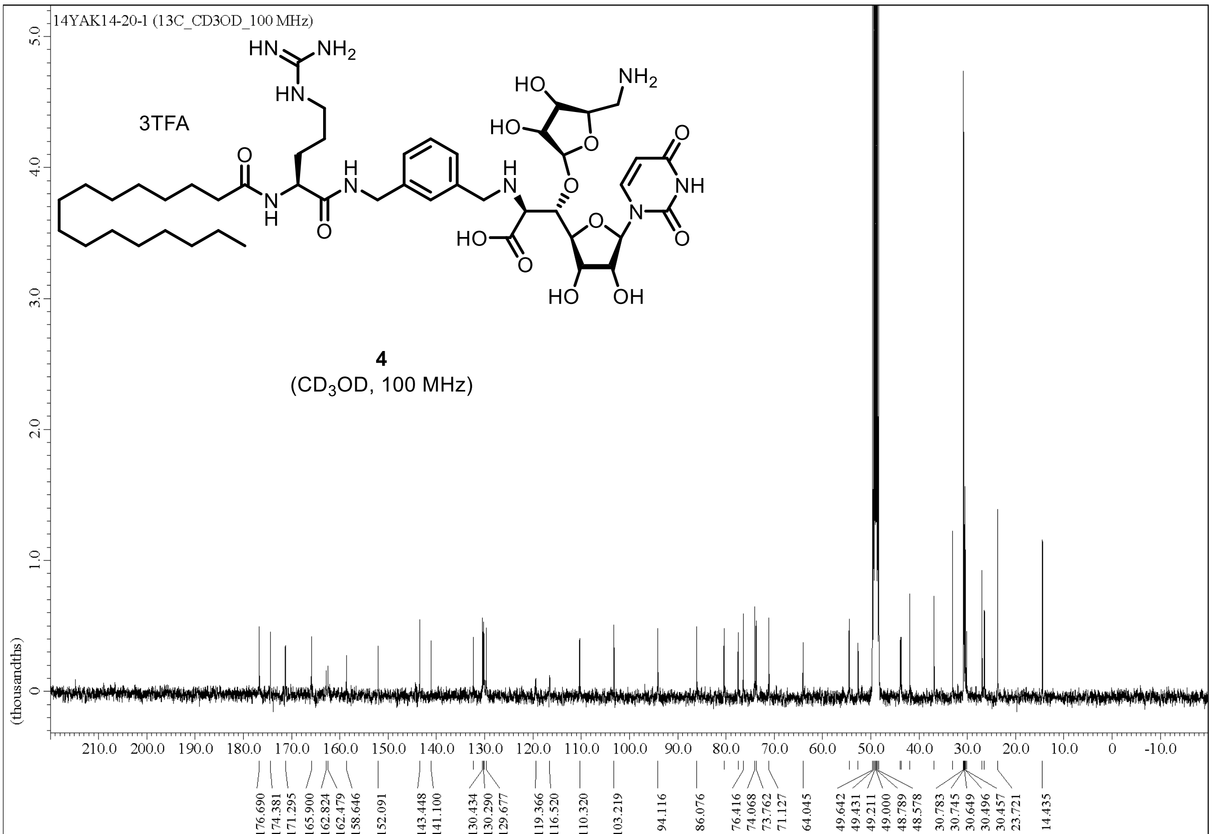

Supplementary Fig. 90. ^{13}C NMR spectrum of compound **4**.

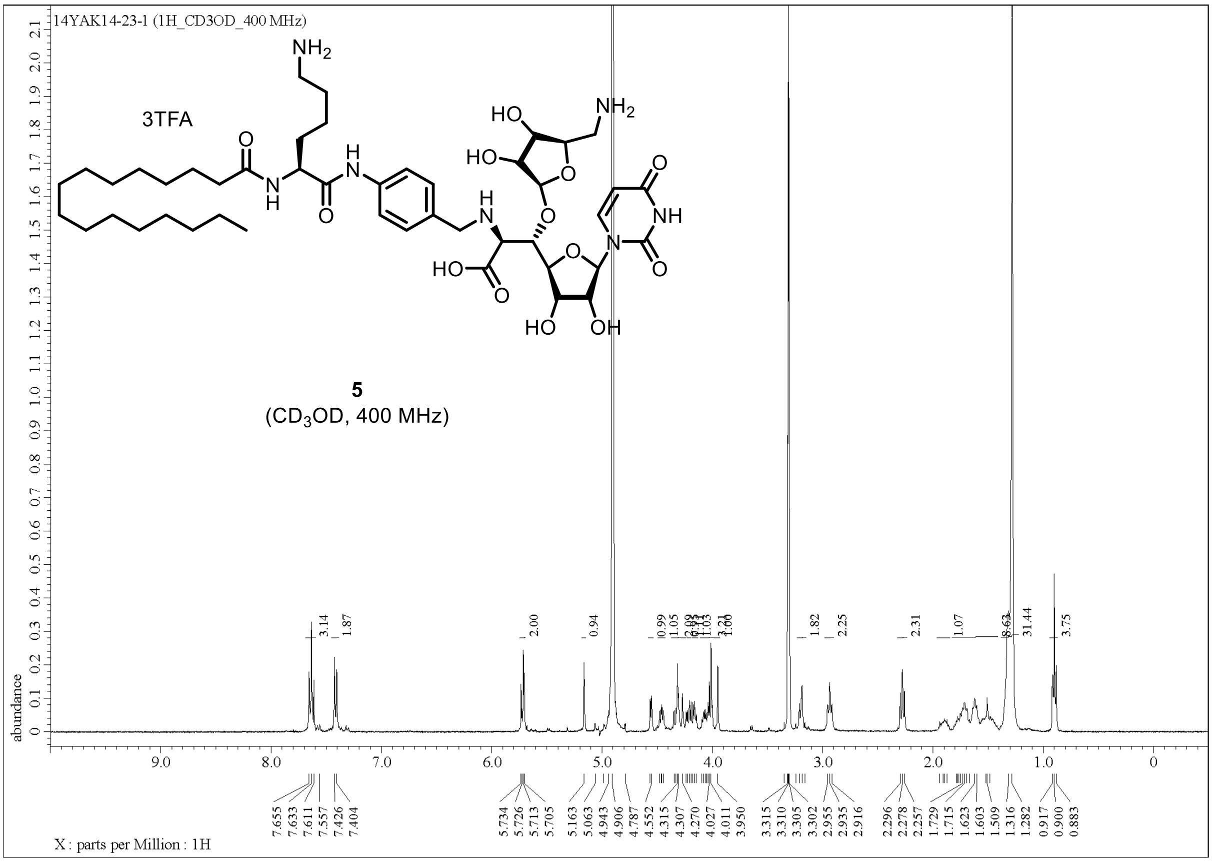

Supplementary Fig. 91. ^{1}H NMR spectrum of compound **5**.

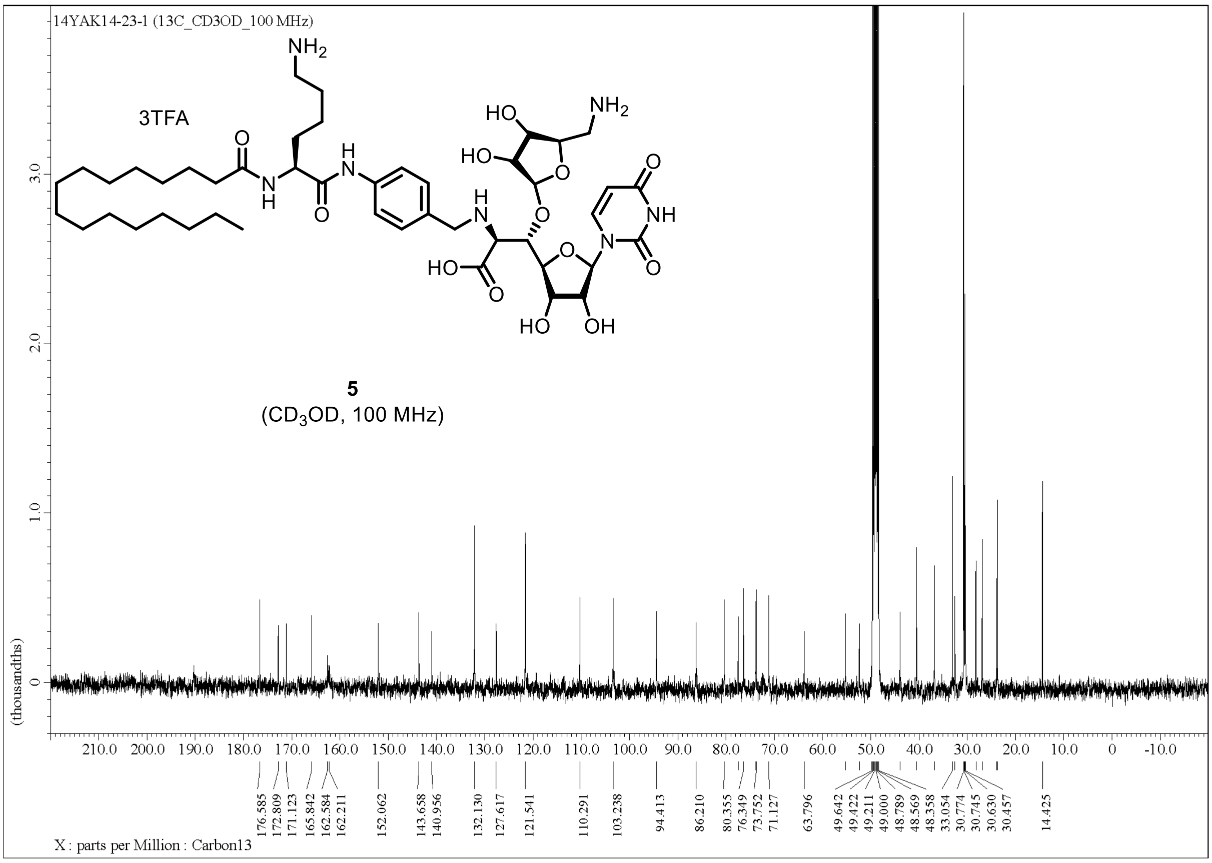

Supplementary Fig. 92. ^{13}C NMR spectrum of compound **5**.

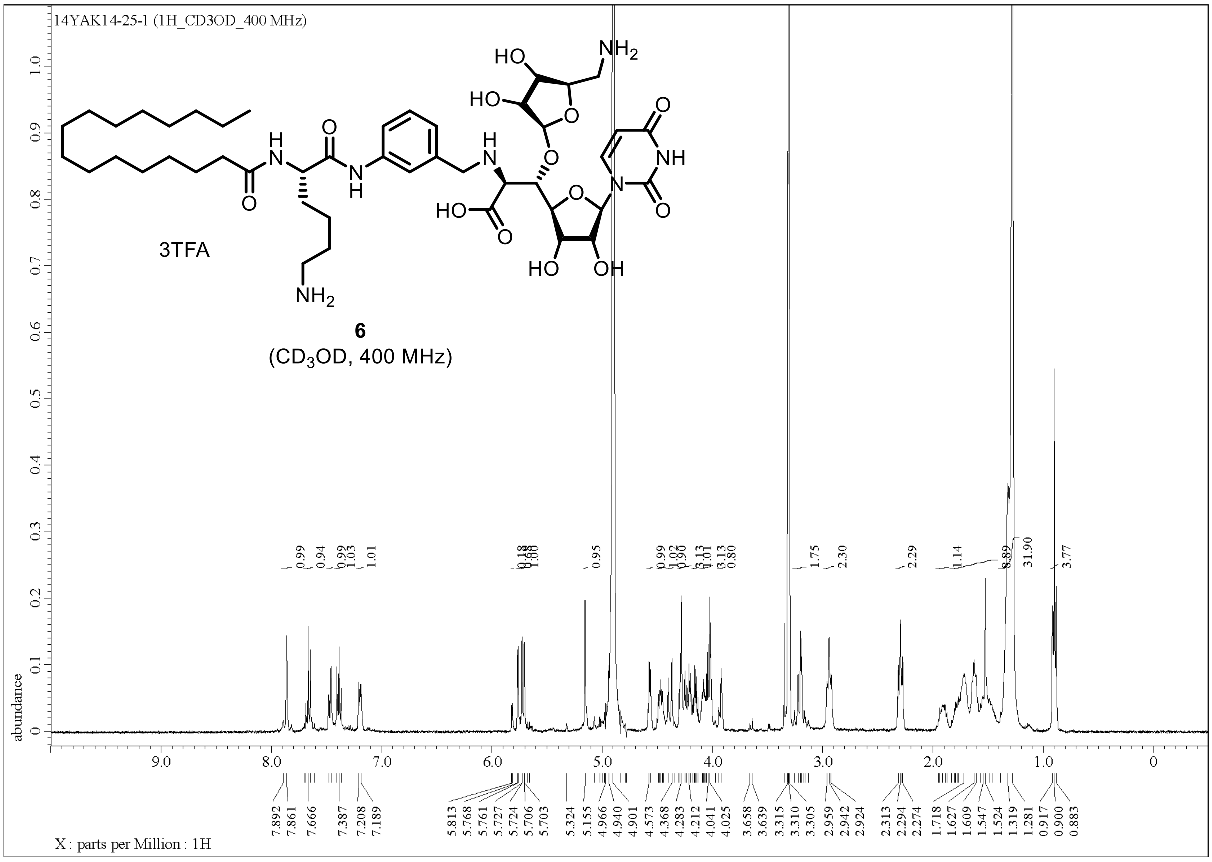

Supplementary Fig. 93. ^{1}H NMR spectrum of compound **6**.

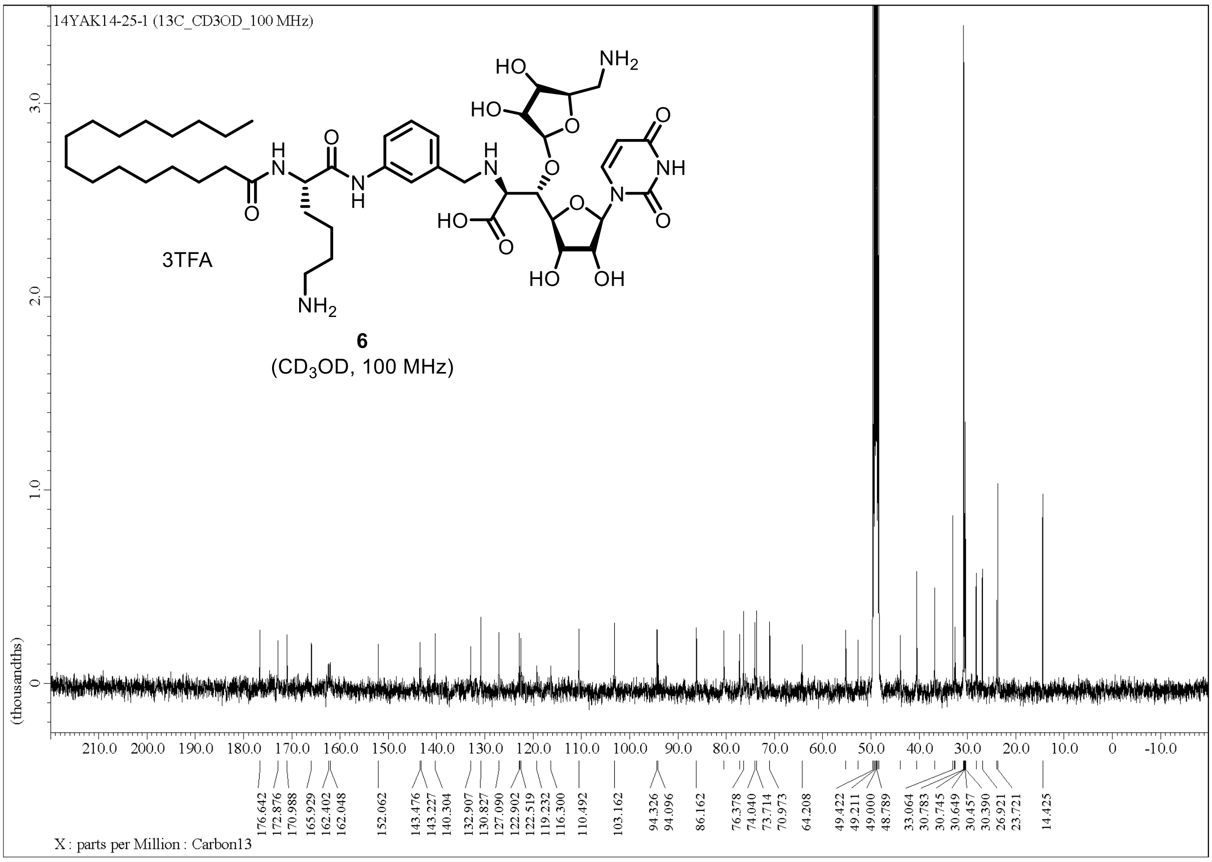

Supplementary Fig. 94. ^{13}C NMR spectrum of compound **6**.

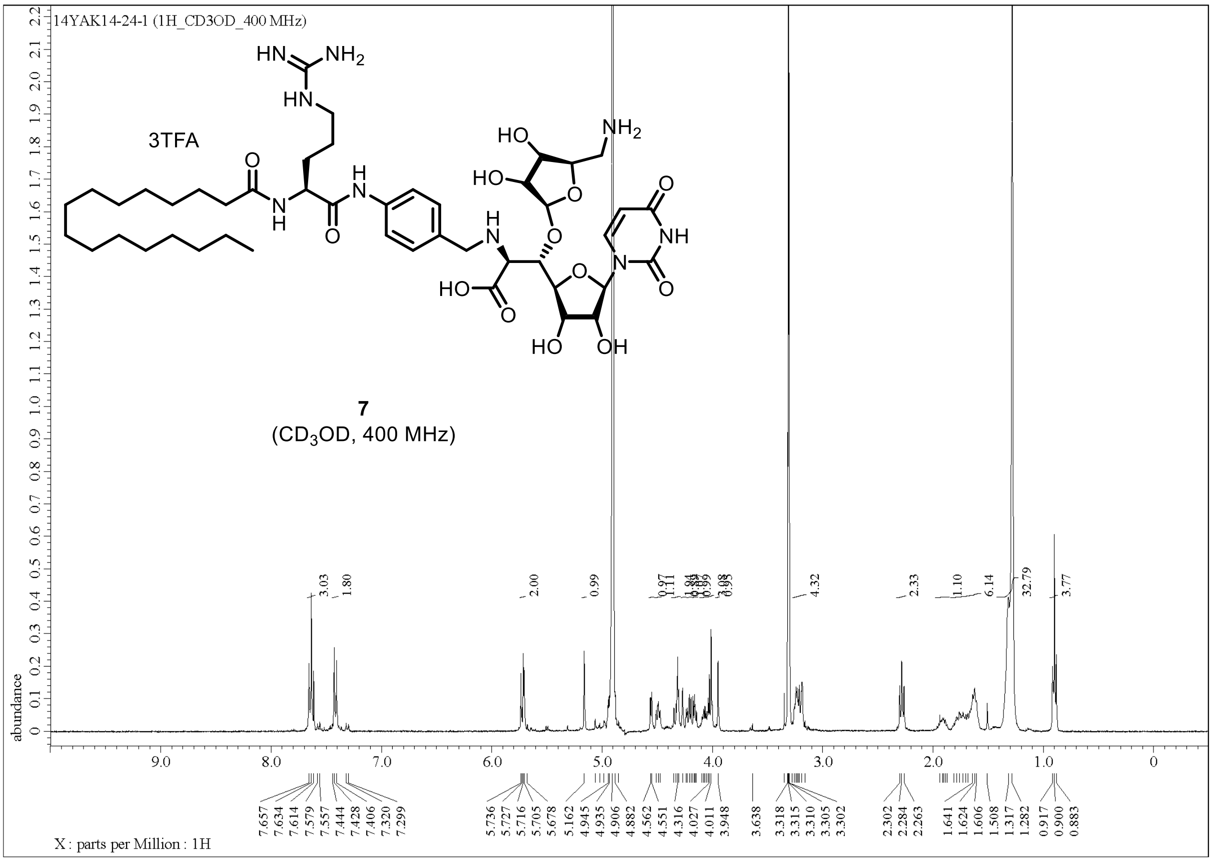

Supplementary Fig. 95. ^{1}H NMR spectrum of compound **7**.

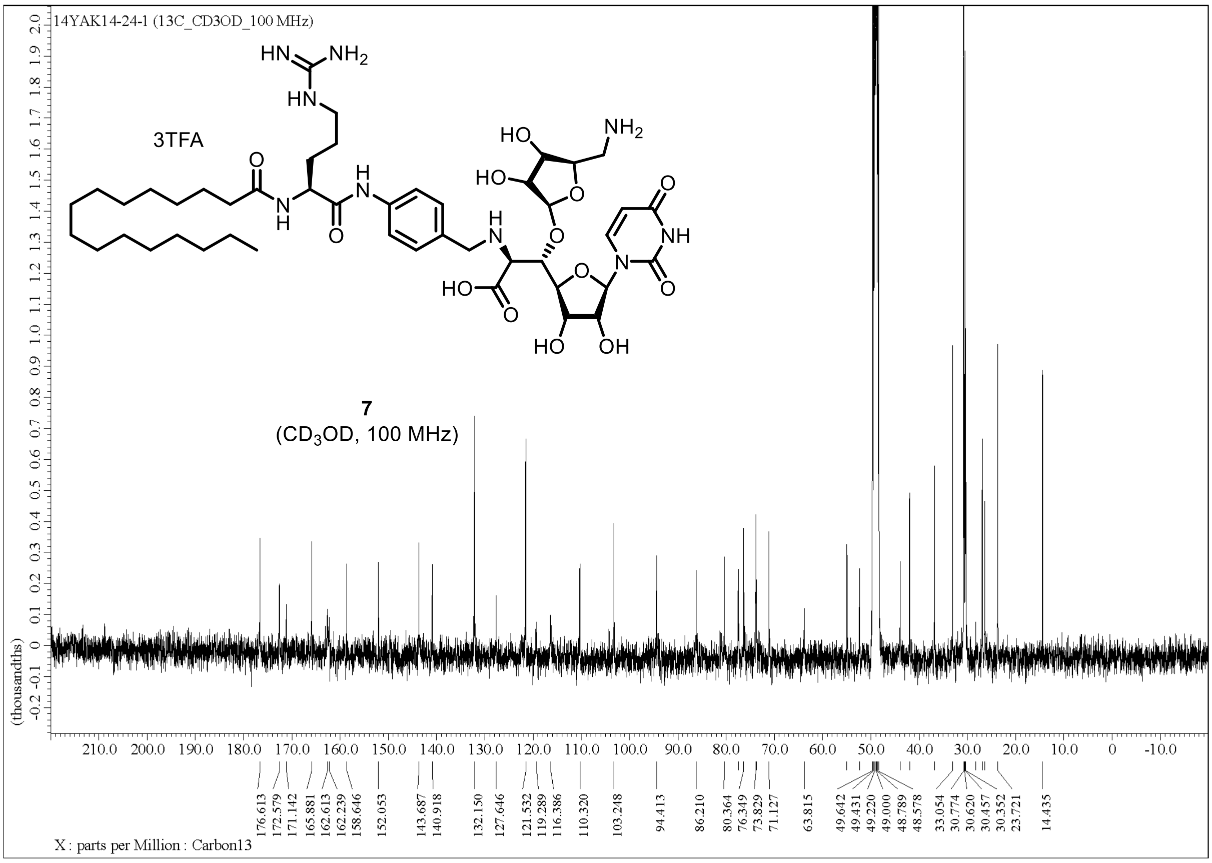

Supplementary Fig. 96. ^{13}C NMR spectrum of compound **7**.

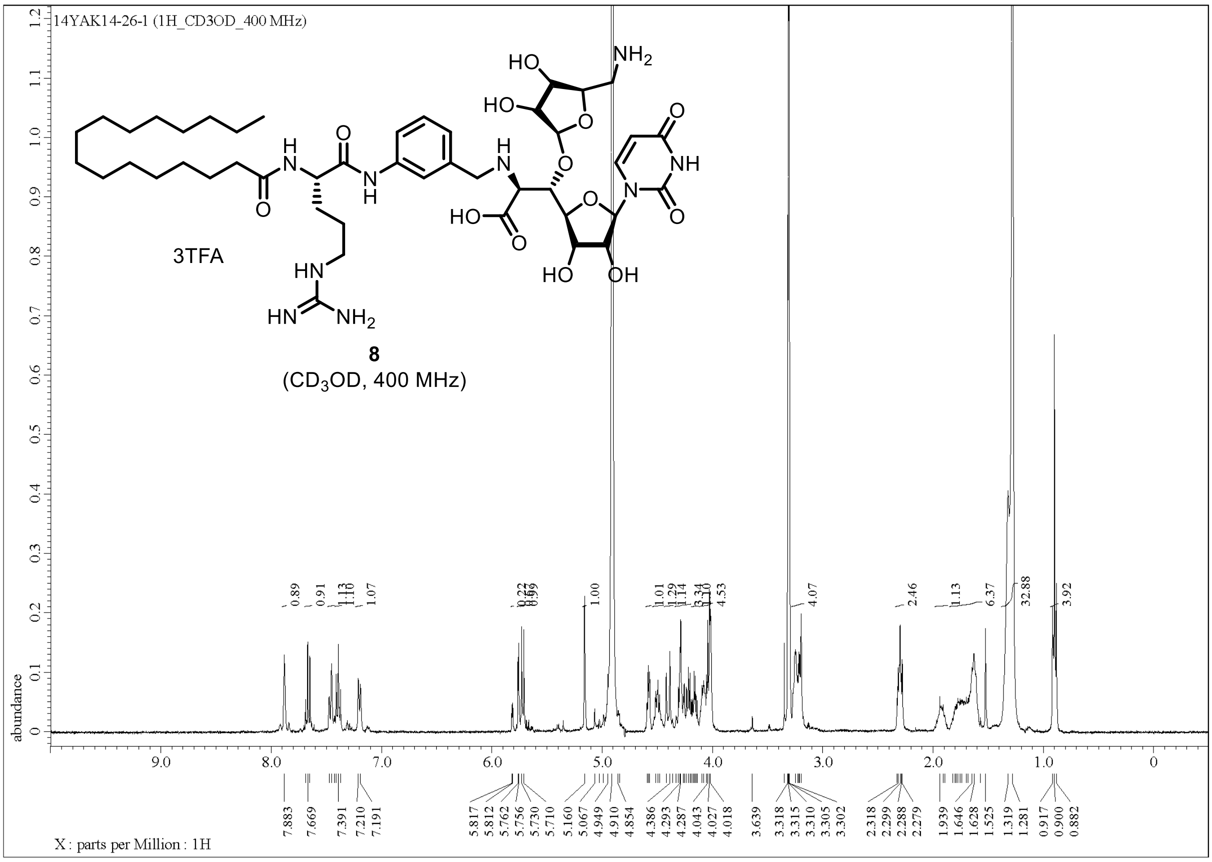

Supplementary Fig. 97. ^{1}H NMR spectrum of compound **8**.

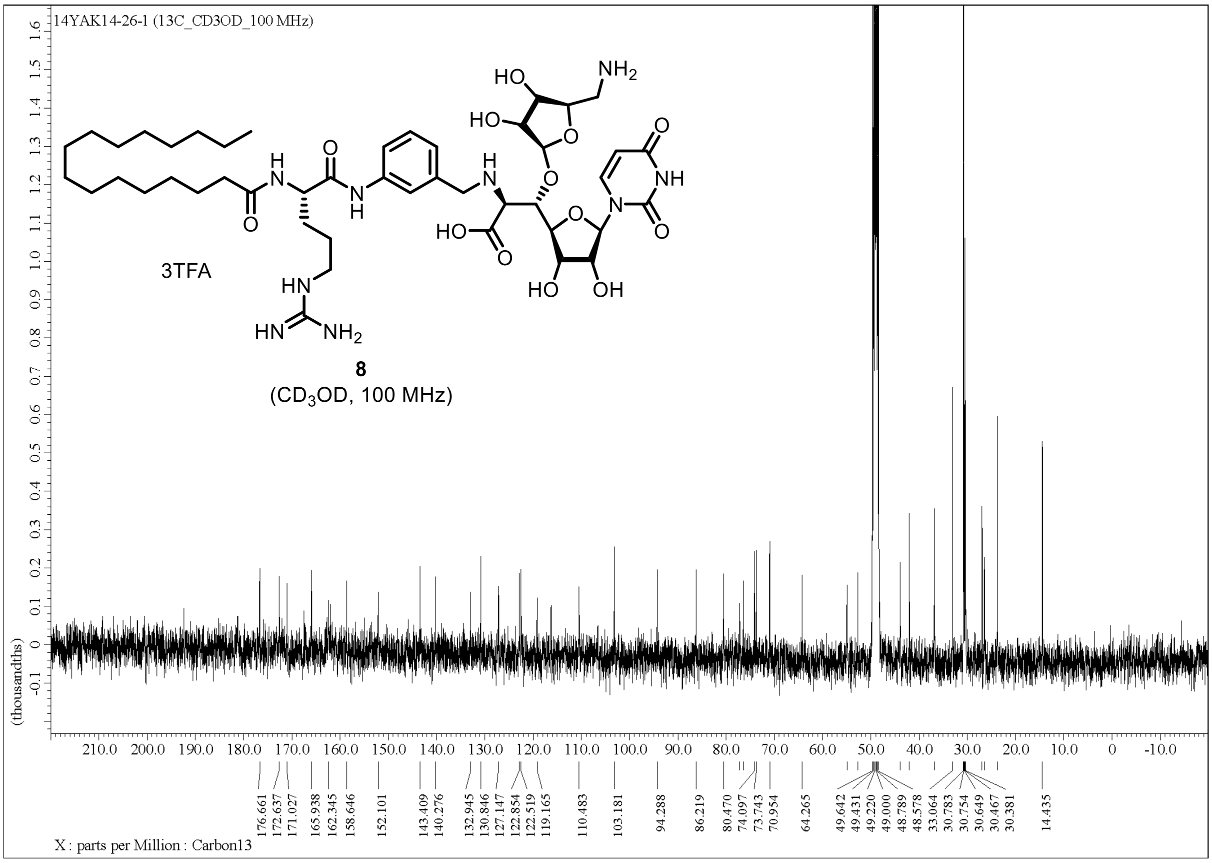

Supplementary Fig. 98. ^{13}C NMR spectrum of compound **8**.

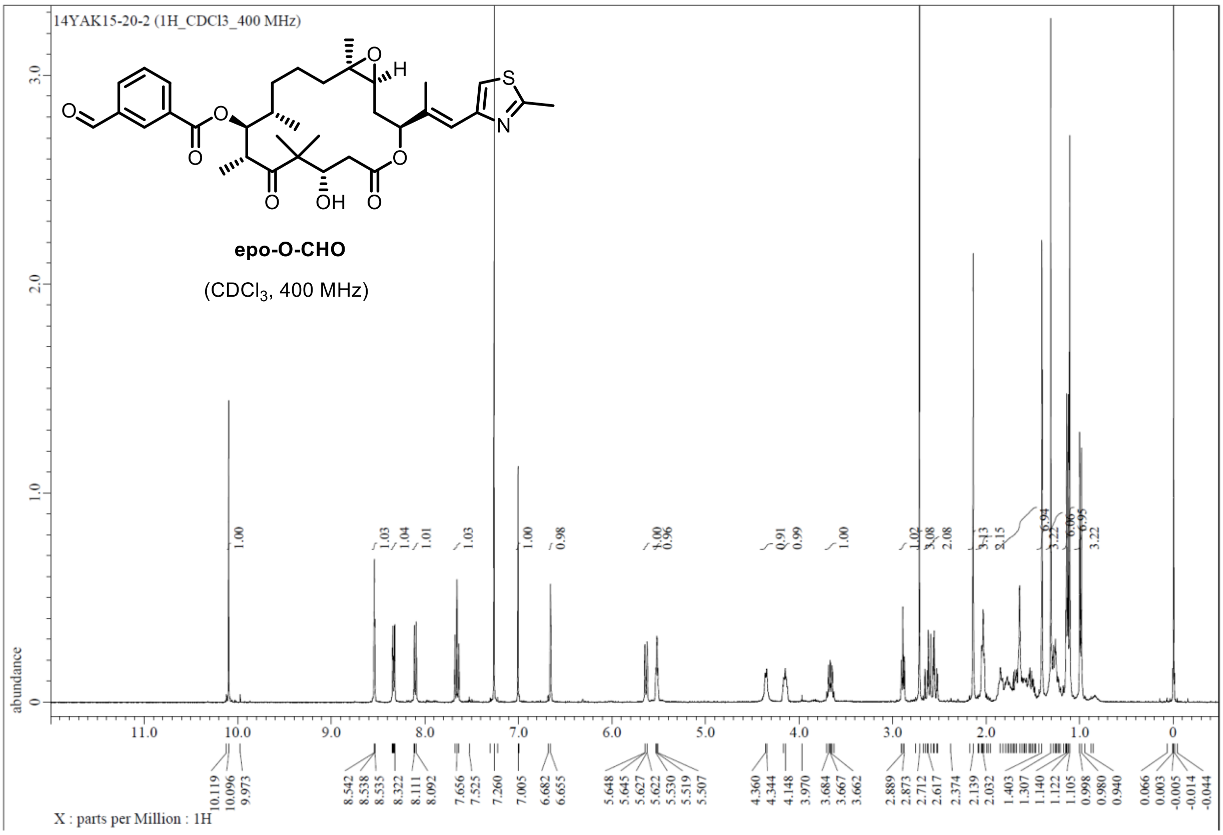

Supplementary Fig. 99. ^{1}H NMR spectrum of compound **epo-O-CHO**.

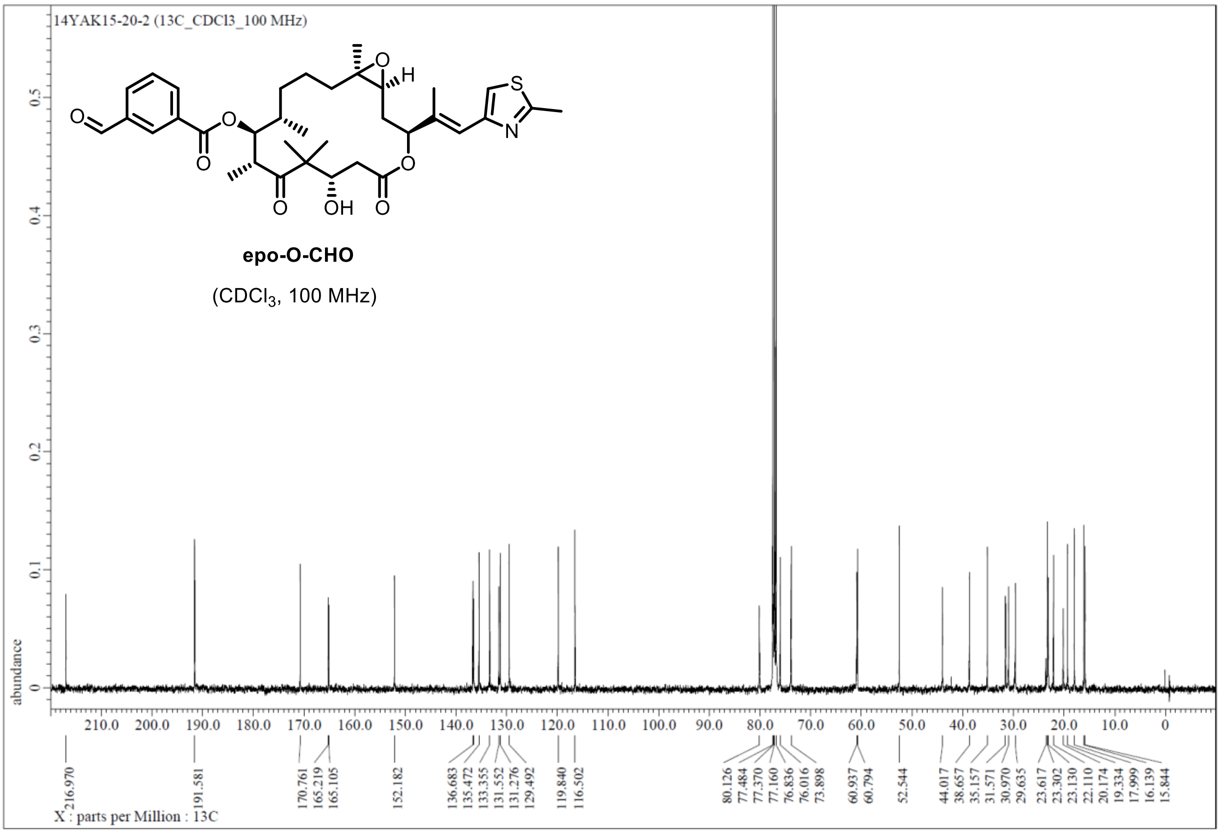

Supplementary Fig. 100. ^{13}C NMR spectrum of compound **epo-O-CHO**.

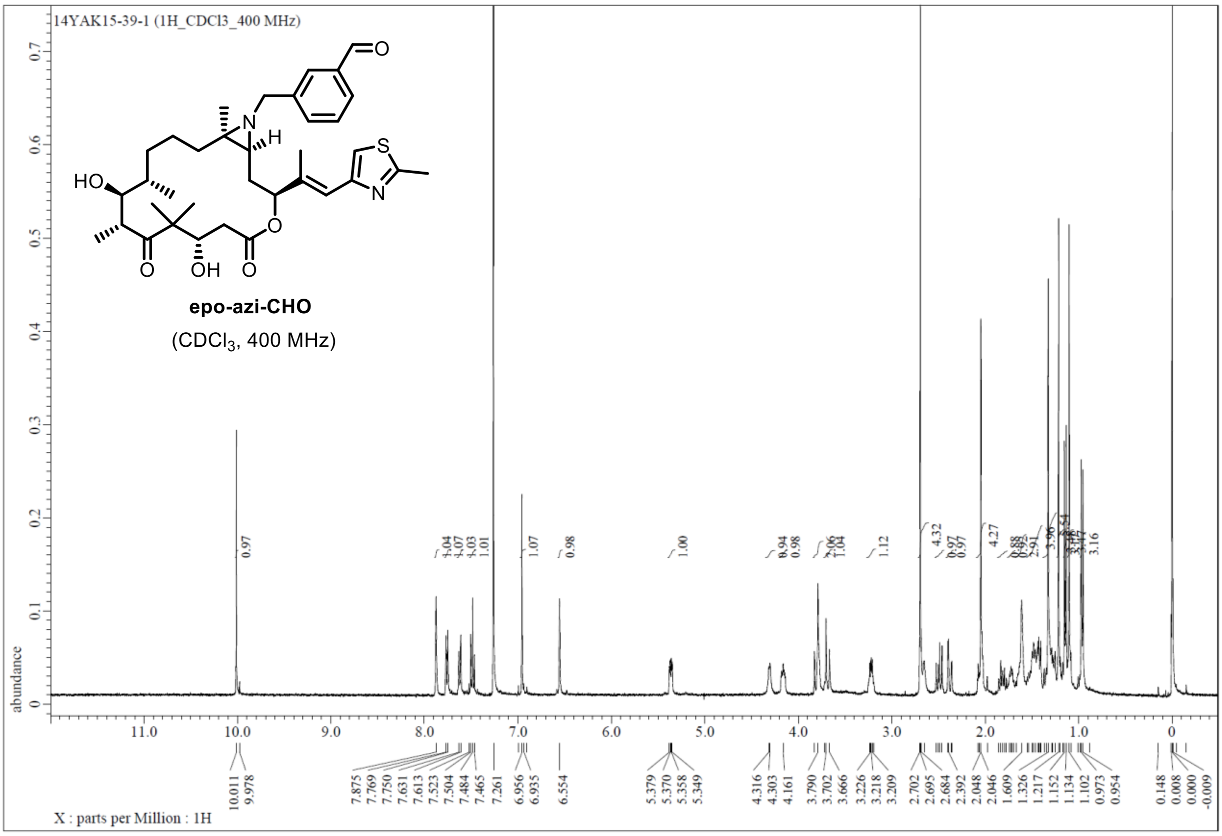

Supplementary Fig. 101. ^{1}H NMR spectrum of compound **epo-azi-CHO**.

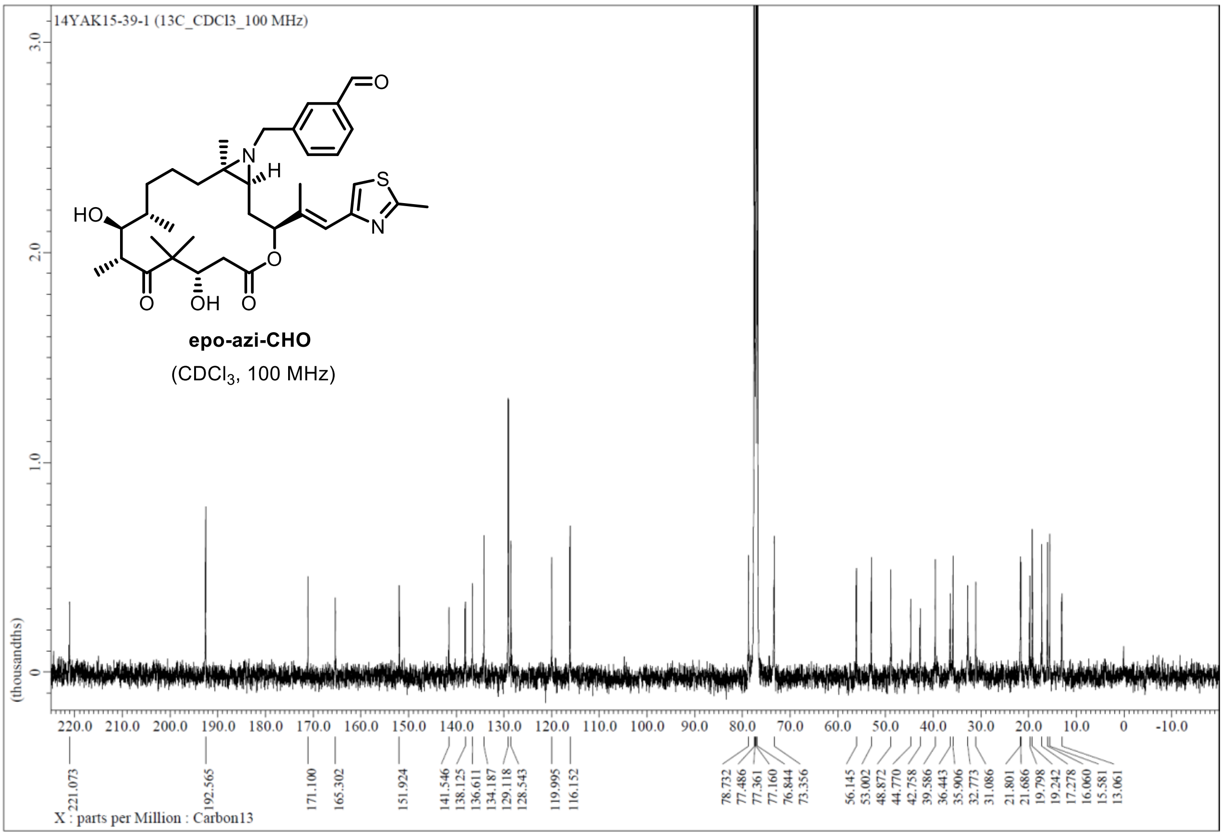

Supplementary Fig. 102. ^{13}C NMR spectrum of compound **epo-azi-CHO**.

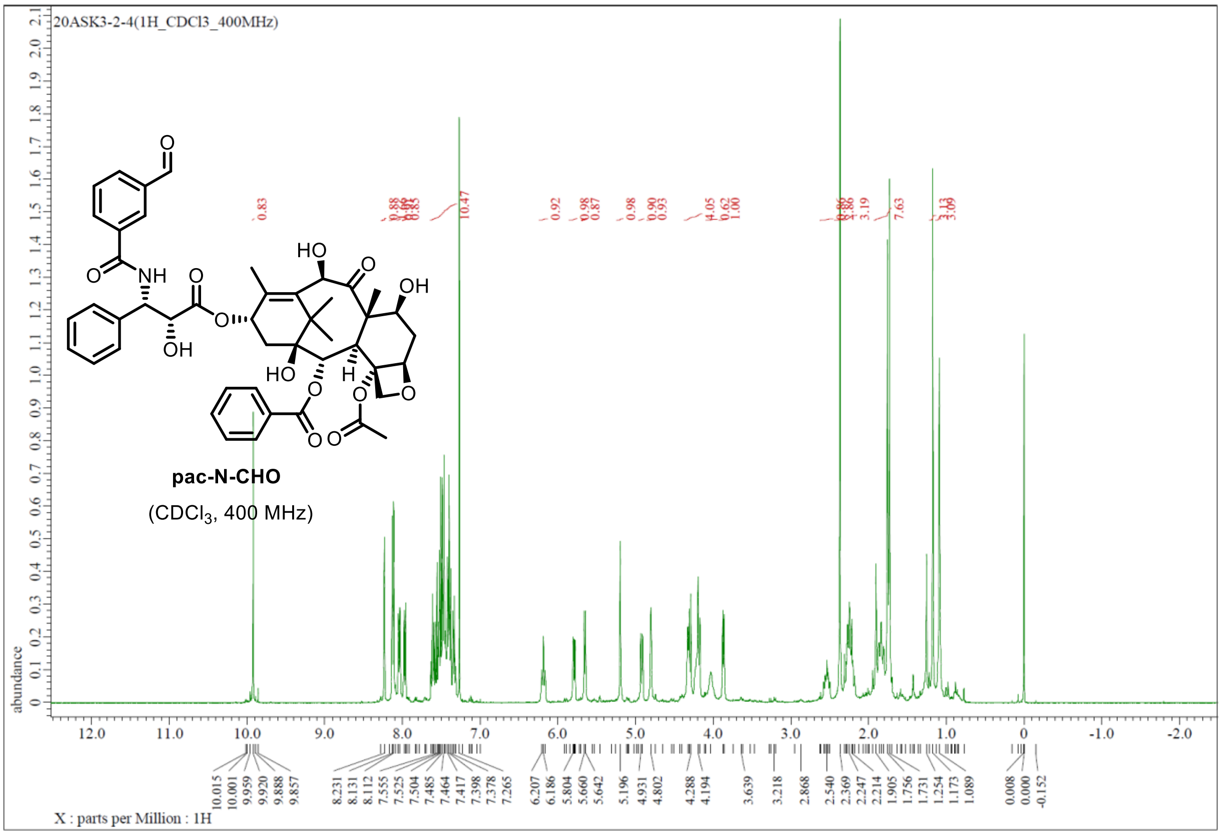

Supplementary Fig. 103. ^{1}H NMR spectrum of compound **pac-N-CHO**.

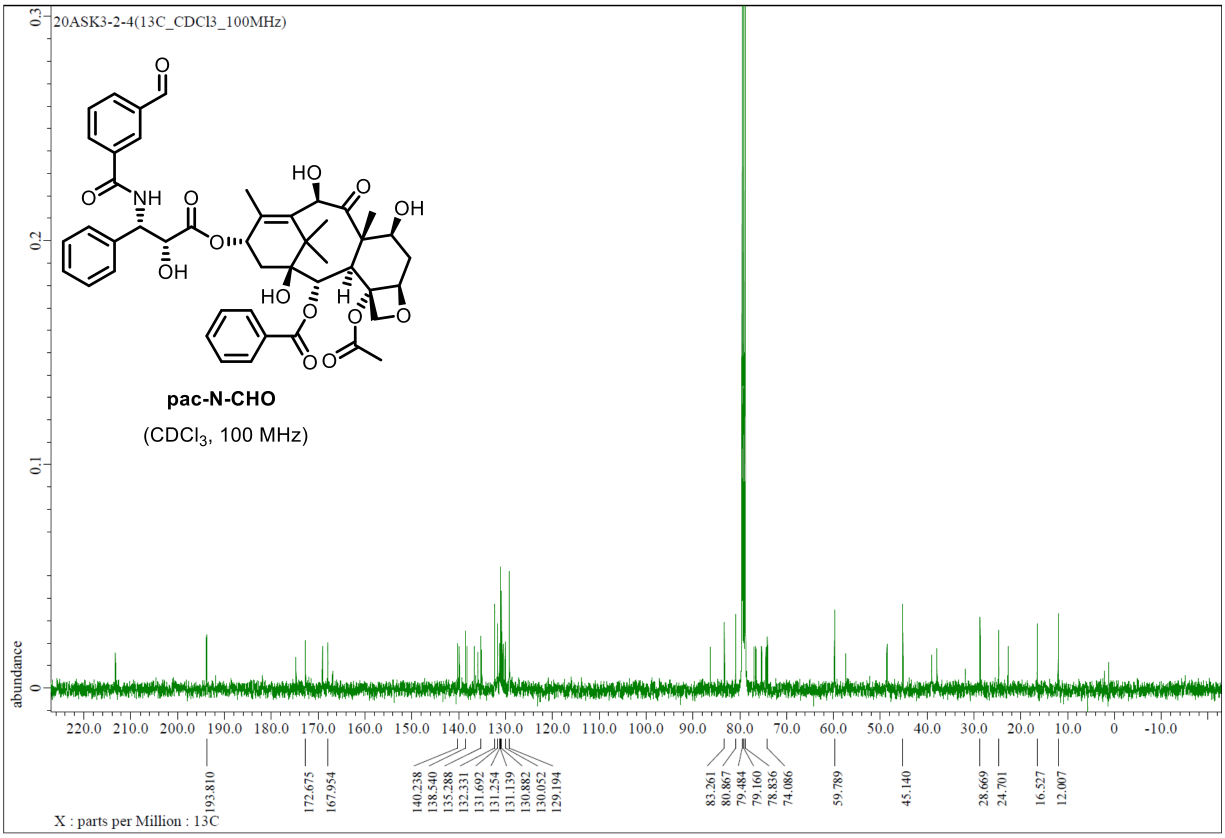

Supplementary Fig. 104. ^{13}C NMR spectrum of compound **pac-N-CHO**.

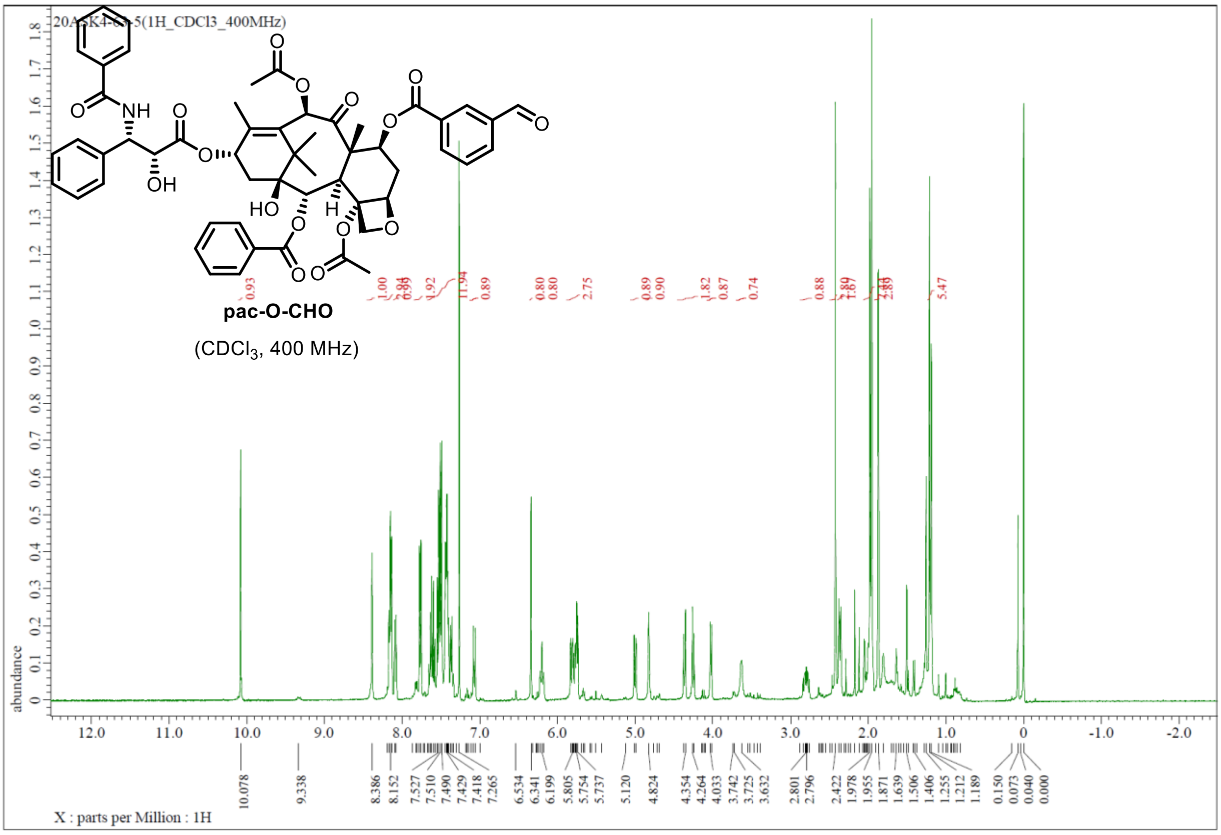

Supplementary Fig.105. ^{1}H NMR spectrum of compound **pac-O-CHO**.

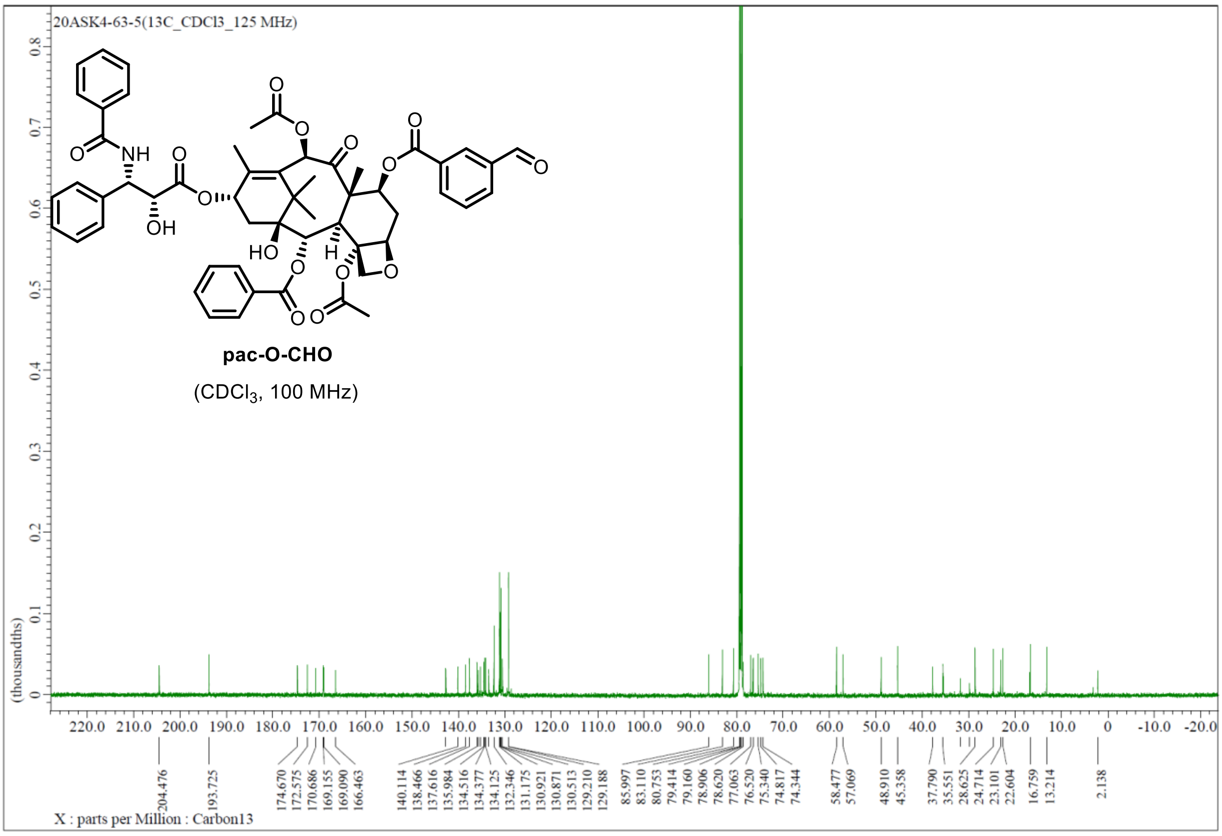

Supplementary Fig. 106. ^{13}C NMR spectrum of compound **pac-O-CHO**.

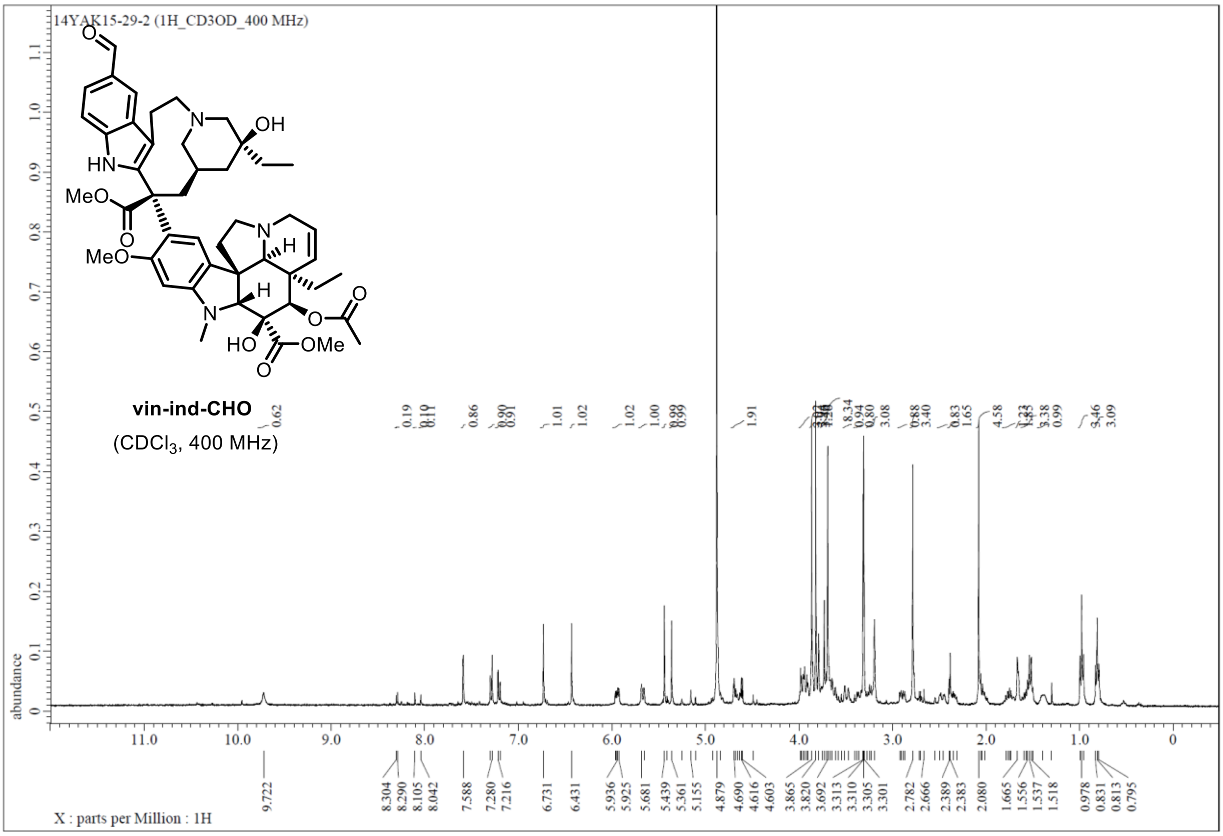

Supplementary Fig. 107. ^{1}H NMR spectrum of compound **vin-ind-CHO**.

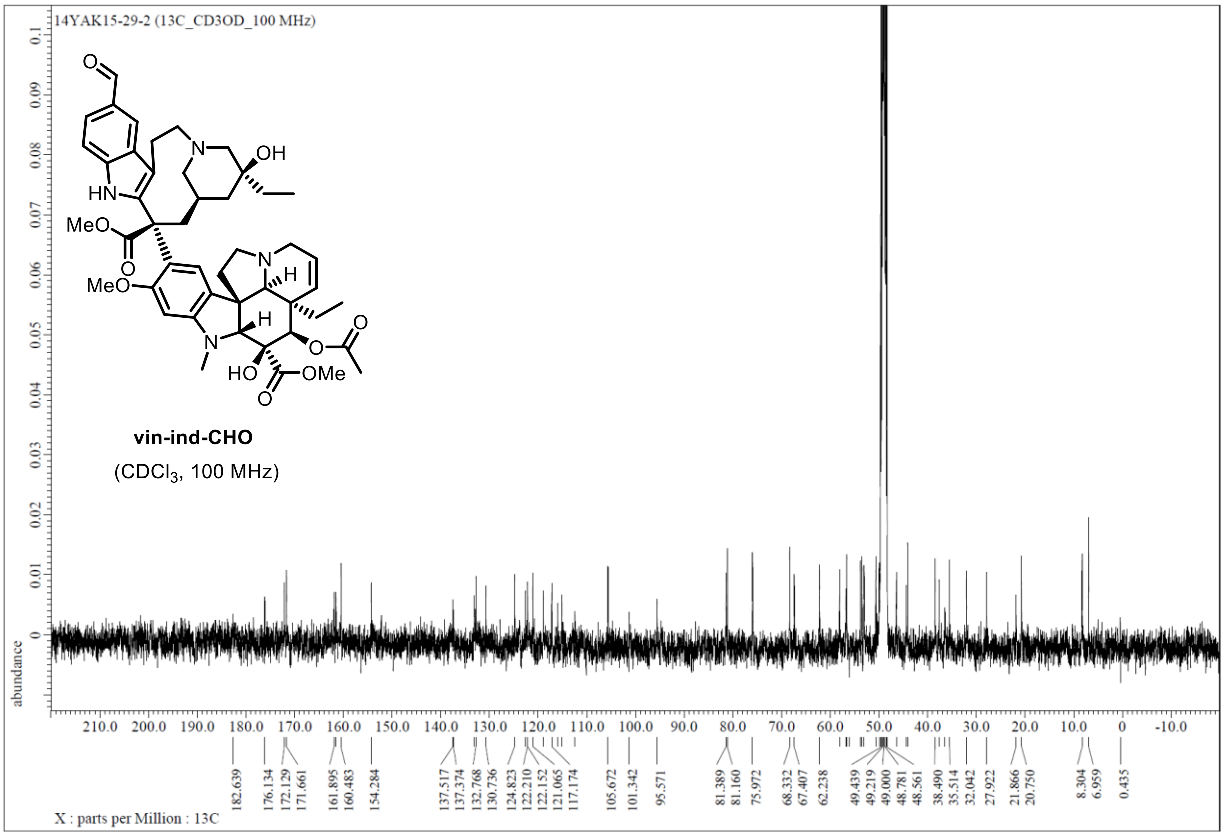

Supplementary Fig. 108. ^{13}C NMR spectrum of compound **vin-ind-CHO**.

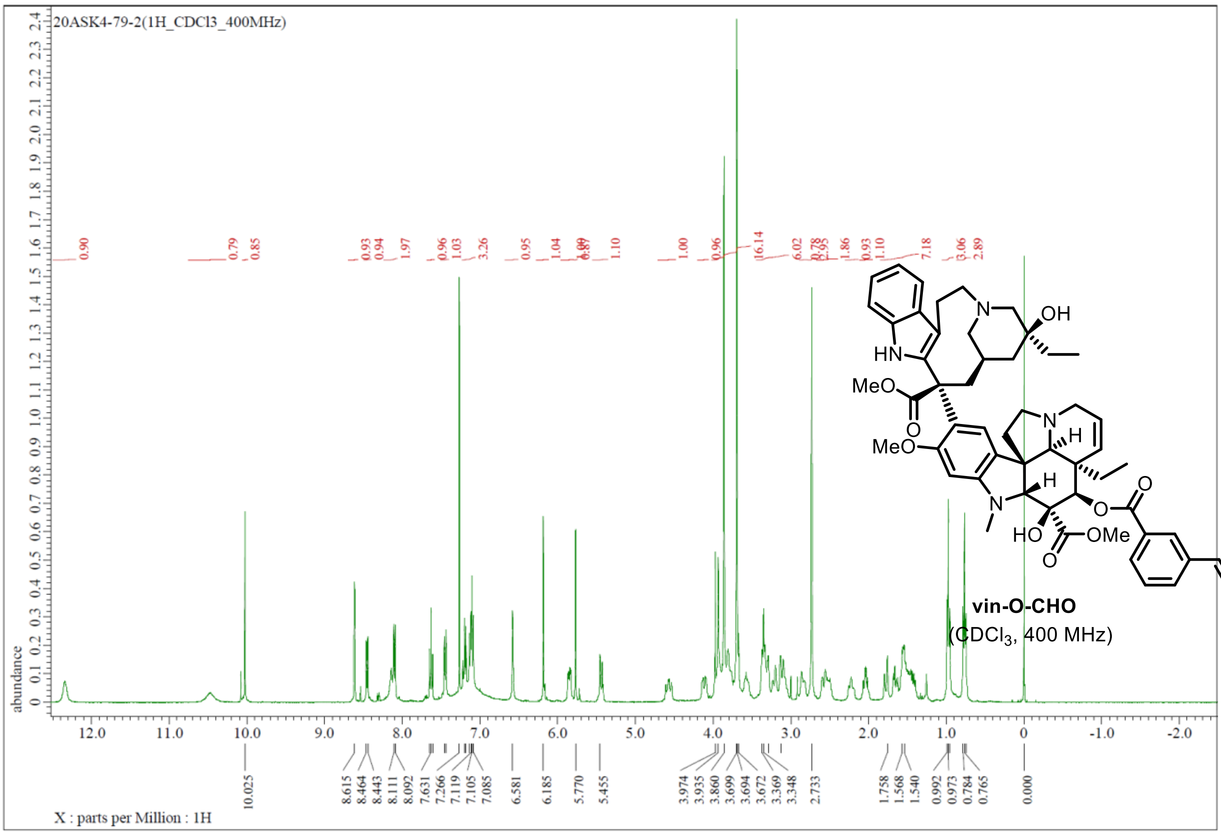

Supplementary Fig. 109. ^{1}H NMR spectrum of compound **vin-O-CHO**.

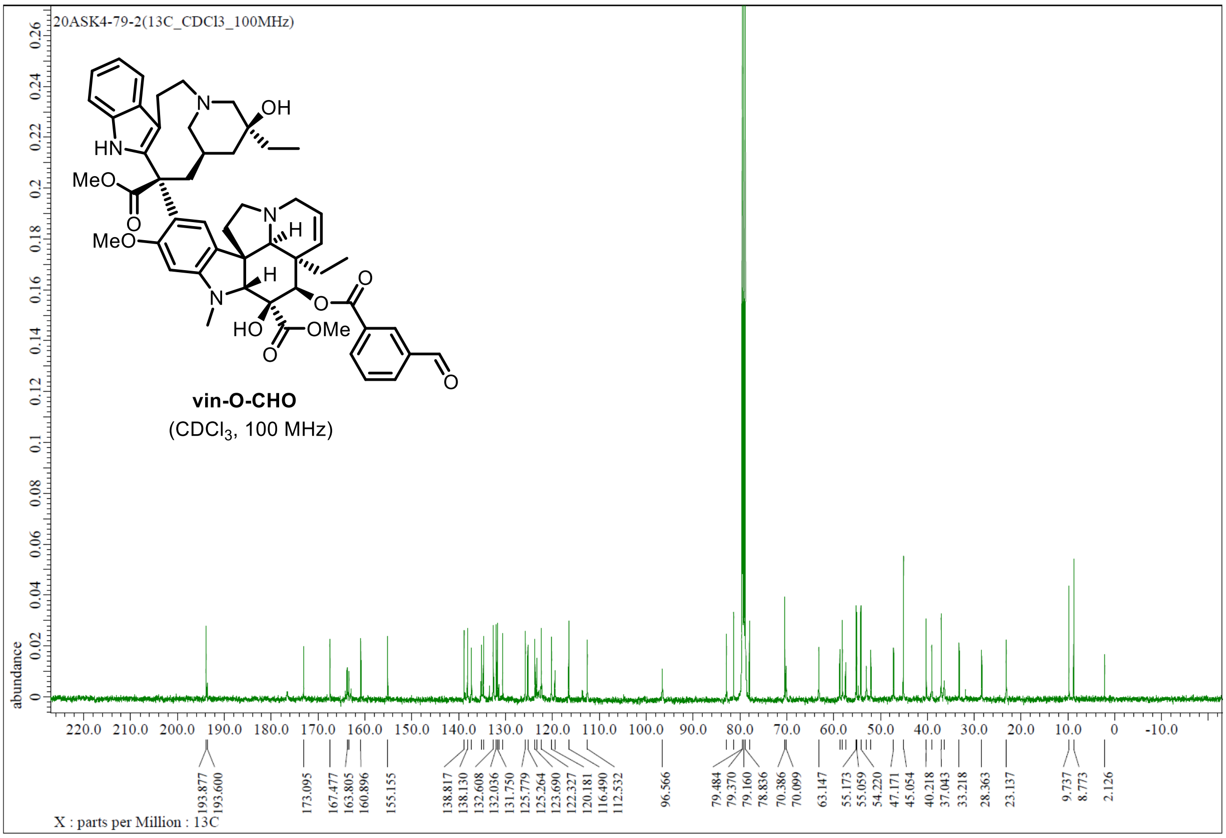

Supplementary Fig. 110. ^{13}C NMR spectrum of compound **vin-O-CHO**.

4. Supplementary References

1) Jurrus, E.; Engel, D.; Star, K.; Monson, K.; Brandi, J.; Felberg, L. E.; Brookes, D. H.; Wilson, L.; Chen, J.; Liles, K.; Chun, M.; Li, P.; Gohara, D. W.; Dolinsky, T.; Konecny, R.; Koes, D. R.; Nielsen, J. E.; Head-Gordon, T.; Geng, W.; Krasny, R.; Wei, G.-W.; Holst, M. J.; McCammon, J. A.; Baker, N. A. Improvements to the APBS biomolecular solvation software suite. *Protein Sci.* **2018**, *27*, 112-128.
2) Brandish, P. E.; Burnham, M. K.; Lonsdale, J. T.; Southgate, R.; Inukai, M.; Bugg, T. D. H. Slow binding inhibition of phospho-*N*-acetylmuramyl-pentapeptide-translocase (*Escherichia coli*) by mureidomycin A. *J. Biol. Chem.* **1996**, *271*, 7609-7614.
3) Ichikawa, S.; Yamaguchi, M.; Hsuan, L. S.; Kato, Y.; Matsuda, A. Carbacaprazamycins: Chemically stable analogueues of the caprazamycin nucleoside antibiotics. *ACS Infect. Dis.* **2015**, *1*, 151-156.
4) Wohnig, S.; Spork, A. P.; Koppermann, S.; Mieskes, G.; Gisch, N.; Jahn, R.; Ducho, C. Total synthesis of dansylated Park’s nucleotide for high-throughput MraY assays. *Chem. Eur. J.* **2016**, *22*, 17813-17819.
5) Katsuyama, A.; Sato, K.; Yakushiji, F.; Matsumaru, T.; Ichikawa, S. Solid-phase modular synthesis of Park nucleoside and lipid I and II analogues. *Chem. Pharm. Bull.* **2018**, *66*, 84-95.
6) Yamamoto, K.; Katsuyama, A.; Ichikawa, S. Structural requirement of tunicamycin V for MraY inhibition. *Bioorg. Med. Chem.* **2019**, *27*, 1714-1719.
7) Tanino, T.; Ichikawa, S.; Shiro, M.; Matsuda, A. Total synthesis of (−)-muraymycin D2 and its epimer. *J. Org. Chem.* **2010**, *75*, 1366-1377.
8) Tanino, T.; Al-Dabbagh, B.; Mengin-Lecreulx, D.; Bouhss, A.; Oyama, H.; Ichikawa, S.; Matsuda, A. Mechanistic analysis of muraymycin analogue: a guide to the design of MraY inhibitors. *J. Med. Chem.* **2011**, *54*, 8421-8439.
9) Terasawa, Y.; Sataka, C.; Sato, T.; Yamamoto, K.; Fukushima, Y.; Nakajima, C.; Suzuki, Y.; Katsuyama, A.; Matsumaru, T.; Yakushiji, F.; Yokota, S.-I.; Ichikawa, S. Elucidating the structural requirement of uridylpeptide antibiotics for antibacterial activity. *J. Med. Chem.* **2020**, *63*, 9803-9827.
10) Xia, X.; Yang, X.; Huang, P.; Yan, D. ROS-responsive nanoparticles formed from RGD-epothilone B conjugate for targeted cancer therapy. *ACS Appl. Mater. Interfaces* **2020**, *12*, 18301-18308.
11) Nicolaou, K. C.; Rhoades, D.; Wang, Y.; Bai, R.; Hamel, E.; Aujay, M.; Sandoval, J.; Gavrilyuk, J. 12, 13-Aziridinyl epothilones. Stereoselective synthesis of trisubstituted olefinic bonds from methyl ketones and heteroaromatic phosphonates and design, synthesis, and biological evaluation of potent antitumor agents. *J. Am. Chem. Soc.* **2017**, *139*, 7318-7334.
12) Nicolaou, K. C.; Shelke, Y. G.; Dherange, B. D.; Kempema, A.; Lin, B.; Gu, C.; Sandoval, J.; Hammond, M.; Aujay, M.; Gavrilyuk, J. Design, synthesis, and biological investigation of epothilone B analogues featuring lactone, lactam, and carbocyclic macrocycles, epoxide, aziridine, and 1,1-difluorocyclopropane and other fluorine residues. *J. Org. Chem.* **2020**, *85*, 2865-2917.
13) Kingston, D. G. I.; Chaudhary, A. G.; Chordia, M. D.; Gharpure, M.; Gunatilaka, A. A. L.; Higgs, P. I.; Rimoldi, J. M.; Samala, L.; Jagtap, P. G. Synthesis and biological evaluation of 2-acyl analogues of paclitaxel (taxol). *J. Med. Chem.* **1998**, *41*, 3715-3726.
14) Meng, G.; Guo, T.; Ma, T.; Zhang, J.; Shen, Y.; Sharpless, K. B.; Dong, J. Modular click chemistry libraries for

functional screens using a diazotizing reagent. *Nature* **2019**, *574*, 86-89.

15) Scott, I. L.; Ralph, J. M.; Voss, M. E. Vinca derivatives, WO 2005/055939.

16) Brady, S. F.; Pawluczyk, J. M.; Lumma, P. K.; Feng, D.-M.; Wai, J. M.; Jones, R.; DeFeo-Jones, D.; Wong, B. K.; Miller-Stein, C.; Lin, J. H.; Oliff, A.; Freidinger, R. M.; Garsky, V. M. Design and synthesis of a pro-drug of vinblastine targeted at treatment of prostate cancer with enhanced efficacy and reduced systemic toxicity. *J. Med. Chem.* **2002**, *45*, 4706-4715.

17) Yamamoto, K.; Yakushiji, F.; Matsumaru, T.; Ichikawa, S. Total synthesis of tunicamycin V. *Org. Lett.* **2018**, *20*, 256-259.

18) Kusaka, S.; Yamamoto, K.; Shinohara M.; Minato, Y.; Ichikawa, S. Synthesis of capuramycin and its analogues via a Ferrier-type I reaction and their biological evaluation. *Bioorg. Med. Chem.* **2022**, *73*, 117011.

19) Dumue, F.; Contal, E.; Wantz, G.; Phan, T. N. T.; Bertin, D.; Gigmes, D. Immobilization of styrene-substituted 1,3,4-oxadiazoles into thermoreversible luminescent organogels and their unexpected photocatalyzed rearrangement. *Chem. Eur. J.* **2013**, *19*, 1373-1384.

20) Zhu, Y.; Wu, J.; Li, C.; Zhu, J.; Hou, J.; Li, C.; Jiang, Li, Z. F···H-N and MeO···H-N hydrogen-bonding in the solid states of aromatic amides and hydrazides: a comparison study. *Cryst. Growth Des.* **2007**, *7*, 1490-1496.

21) Hwang, J. Y.; Choi, H.; Lee, D.; Gong, Y. Solid-phase synthesis of 1,3,4-oxadiazole and 1,3,4-thiadiazole derivatives via selective, reagent-based cyclization of acyldithiocarbazate resins. *J. Comb. Chem.* **2005**, *7*, 816.819.

22) Husain, A.; Ahmad, A.; Alam, M. M.; Ajmal, M.; Ahuja, P. Fenbufen based 3-[5-(substituted aryl)-1,3,4-oxadiazol-2-yl]-1-(biphenyl-4-yl)propan-1-ones as safer anti-inflammatory and analgesic agents. *Eur. J. Med. Chem.* **2009**, *44* 3798-3804.

23) Ashiq, U.; Jamal, R. A.; Mahroof-Tahir, M.; Maqsood, Z. T.; Khan, K. M.; Omer, I. Choudhary, M. I. Enzyme inhibition, radical scavenging, and spectroscopic studies of vanadium(IV)-hydrazide complexes. *J. Enzyme Inhib. Med. Chem.* **2009**, *24*, 1336-1343.

24) Ono, M.; Kobayashi, T.; Yamazaki, R.; Haibara, H.; Nishiyama, Y.; Hohashi, A.; Nishiyama, H.; Kurita, A.; Matsuzaki, T.; Kono, K.; Izumi, H. Preparation of heteroaryl compounds as ZNF143 inhibitors. *Jpn. Kokai Tokkyo Koho* **2016**, JP2016124812 B2 20160711.

25) Zender, M.; Klein, T.; Henn, C.; Kirsch, B.; Maurer, C. K.; Kail, D.; Ritter, C.; Dolezal, O.; Steinbach, A.; Hartmann, R. W. Discovery and biophysical characterization of 2-amino-oxadiazoles as novel antagonists of PqsR, an important regulator of Pseudomonas aeruginosa virulence. *J. Med. Chem.* **2013**, *56*, 6761-6774.

26) Barrett, D. G.; Deaton, D. N.; McFadyen, R. B.; Miller, A. B.; Ray, J. A.; Tavares, F. X.; Zhou, H. Preparation of α-ketoamide derivatives as cathepsin K inhibitors useful against bone disorders such as osteoporosis. *PCT Int. Appl*. **2003**, WO03013518 A1 20030220.

27) Hou, J.; Shao, X.; Chen, G.; Zhou, Y.; Jiang, X.; Li, Z. Hydrogen bonded oligo-hydrazide foldamers and their recognition for saccharides. *J. Am. Chem. Soc.* **2004**, *126*, 12386-12394.

28) Ashiq, U.; Ara, R.; Mahroof-Tahir, M.; Maqsood, Z. T.; Khan, K. M.; Khan, S. N.; Siddiqui, H.; Choudhary, M. I. Synthesis, spectroscopy, and biological properties of vanadium(IV)-hydrazide complexes. *Chem. Biodivers.* **2008**, *5*, 82-92.

29) Yu, D. D.; Forman, B. M. Identification of an agonist ligand for estrogen-related receptors ERRβ/γ. *Bioorg. Med. Chem. Lett.* **2005**, *15*, 1311-1313.

30) Khan, K. M.; Shujaat, S.; Rahat, S.; Hayat, S.; Atta-ur-Rahman; Choudhary, M. I. β-*N*-Cyanoethyl acyl hydrazide derivatives: a new class of β- glucuronidase inhibitors. *Chem. Pharm. Bull.* **2002**, *50*, 1443-1446.

31) Xue, S.; Ma, X. Preparation of neonicotinoid clothianidin analogs as insecticides. *Faming Zhuanli Shenqing* **2011**, CN102070629 A 20110525,

32) Ji, Z.; Wu, Y.; Wu, F. A ratiometric fluorescence sensor for zinc in neutral solution based on thiourea receptor. *Chem. Lett.* **2006**, *35*, 950-951.

33) Kim, Y.; Koh, M.; Kim, D.; Choi, H.; Park, S. B. Efficient discovery of selective small molecule agonists of estrogen-related receptor γ using combinatorial approach. *J. Comb. Chem.* **2009**, *11*, 928-937.

34) Yelamaggad, C. V.; Achalkumar, A. S.; Rao, D. S. Shankar; Prasad, S. K. Luminescent, liquid crystalline tris(N-salcylideneaniline)s: synthesis and characterization. *J. Org. Chem.* **2009**, *74*, 3168-3171.

35) Lu, T.; Lu, S.; Kong, K.; Liu, H.; Chen, Y.; Gao, Y.; Zhang, L.; Sun, S. Aromatic diacylhydrazines as PLK1 inhibitors and their preparation, pharmaceutical compositions and use in the treatment of cancers. *Faming Zhuanli Shenqing* **2011**, CN 102174035 A 20110907.

36) Klingele, M. H.; Brooker, S. From N-substituted thioamides to symmetrical and unsymmetrical 3,4,5-trisubstituted 4H-1,2,4-triazoles: synthesis and characterisation of new chelating ligands. *Eur. J. Org. Chem.* **2004**, *16*, 3422-3434.

37) Zamani, K.; Faghihi, K.; Tofighi, T.; Shariatzadeh, M. R. Synthesis and antimicrobial activity of some pyridyl and naphthyl substituted 1,2,4-triazole and 1,3, 4-thiadiazole derivatives. *Turk. J. Chem.* **2004**, *28*, 95-100.

38) Bhutani, H.; Singh, S.; Vir, S.; Bhutani, K. K.; Kumar, R.; Chakraborti, A. K.; Jindal, K. C. LC and LC-MS study of stress decomposition behaviour of isoniazid and establishment of validated stability-indicating assay method. *J. Pharm. Biomed. Anal.* **2007**, *43*, 1213-1220.

39) Mugherli, L.; Burchak, O. N.; Balakireva, L. A.; Thomas, A.; Chatelain, F.; Balakirev, M. Y. In situ assembly and screening of enzyme inhibitors with surfacetension microarrays. *Angew. Chem. Int. Ed.* **2009**, *48*, 7639-7644.

40) Xie, J.; Zhu, X.; Huang, M.; Meng, F.; Chen, W.; Wan, Y. Pyrrole-2-carbohydrazides as ligands for Cu-catalyzed amination of aryl halides with amines in pure water. *Eur. J. Org. Chem.* **2010**, *17*, 3219-3223.

41) Quesnel, J. S.; Kayser, L. V.; Fabrikant, A.; Arndtsen, B. A. Acid chloride synthesis by the palladium-catalyzed chlorocarbonylation of aryl bromides. *Chem. Eur. J.* **2015**, *21*, 9550-9555.

42) Brown, S. D.; Coburn, C. A.; Kharenko, O. Preparation of substituted imidazolyl triazoles as BET protein inhibitors via binding to bromodomains useful in therapy. *PCT Int. Appl.* **2016**, WO 2016092375 A1 20160616.

43) Ameryckx, A.; Thabault, L.; Pochet, L.; Leimanis, S.; Poupaert, J. H.; Wouters, J.; Joris, B.; Bambeke, F. V.; Frédérick, R. 1-(2-Hydroxybenzoyl)-thiosemicarbazides are promising antimicrobial agents targeting D-alanine-D-alanine ligase in bacterio. *Eur. J. Med. Chem.* **2018**, *159*, 324-338.

44) Kubo, Y.; Aotake, T.; Yakushiji, H.; Yamamoto, T. Dibenzo pyrromethene boron chelate compound, the near infrared light absorbing dye, a photoelectric converter, a near-infrared light sensor and an image sensor. *Jpn. Kokai Tokkyo Koho* **2018**, JP 2018123093 A 20180809.

45) Taha, M.; Sultan, S.; Imran, S.; Rahim, F.; Zaman, K.; Wadood, A.; Rehman, A.U.; Uddin, A.; Khan, K. M. Synthesis of quinoline derivatives as diabetic II inhibitors and molecular docking studies. *Bioorg. Med. Chem.* **2019**, *27*, 4081-4088.

46) Hajimahdi, Z.; Zabihollahi, R.; Aghasadeghi, M. R.; Zarghi, A. Design, synthesis and docking studies of new 4-hydroxyquinoline-3-carbohydrazide derivatives as anti-HIV-1 agents. *Drug Res.* **2013**, *63*, 192-197.

47) Dulla, B.; Wan, N.; Franzblau, S. G.; Kapavarapu, R.; Reiser, O.; Iqbal, J.; Pal, M. Construction and functionalization of fused pyridine ring leading to noval compounds as potential antitubercular agents. *Bioorg. Med. Chem. Lett.* **2012**, *22*, 4629-4635.

48) Kumar, D.; Kumar N. M.; Noel, B.; Shah, K. A series of 2-arylamino-5-(indolyl)-1,3,4-thiadiazoles as potent cytotoxic agents. *Eur. J. Med. Chem.* **2012**, *55*, 432-438.

49) Suo, Y.; Ye, Y.; Han, L. Preparation of hydrzinecarbonylindole compounds as antimicrobial agents. *Northwest Inst. Plateau Bio.* **2015**, CN20151038346 A 20150126.

50) Bombrun, A.; Gerber, P.; Church, D. A. Preparation of oxindole hydrazide modulators of protein tyrosine phosphatases (PTPs). *PCT Int. Appl.* **2003**, WO03037328 A1 20030508.

51) Thompson, S. K.; Halbert, S. M.; DesJarlais, R. L.; Tomaszek, T. A.; Levy, M. A.; Tew, D. G.; Ijames, C. F.; Veber, D. F. Structure-based design of non-peptide, carbohydrazide-based cathepsin K inhibitors. *Bioorg. Med. Chem.* **1999**, *7*, 599-605.

52) Monga, V.; Nayyar, A.; Vaitilingam, B.; Palde, P. B.; Jhamb, S. S.; Kaur, S.; Singh, P. P.; Jain, R. Ring-substituted quinolines. Part 2: synthesis and antimycobacterial activities of ring-substituted quinolinecarbohydrazide and ring-substituted quinolinecarboxamide analogs. *Bioorg. Med. Chem.* **2004**, *12*, 6465-6472.

53) Shang, Y.; Hao, Q.; Jiang, K.; He, M.; Wang, J. Discovery of heterocyclic carbohydrazide derivatives as novel selective fatty acid amide hydrolase inhibitors: design, synthesis and anti-neuroinflammatory evaluation. *Bioorg. Med. Chem. Lett.* **2020**, *30*, 127118.

54) Lancelot, J. C.; Maume, D.; Robba, M. Pyrazolo[1,5-*d*]triazines-1,2,4. I. Dérivés pyrazoliques. *J. Heterocycl. Chem.* **1981**, *18*, 1319-1324.

55) Zarghi, A.; Tabatabai, S. A.; Faizi, M.; Ahadian, A.; Navabi, P.; Zanganeh, V.; Shafiee, A. Synthesis and anticonvulsant activity of new 2-substituted-5-(2-benzyloxyphenyl)-1,3,4-oxadiazoles. *Bioorg. Med. Chem. Lett.* **2005**, *15*, 1863-1865.

56) Bera, H.; Tan, B. J.; Sun, L.; Dolzhenko, A. V.; Chui, W. K.; Chiu, G. N. A structure-activity relationship study of 1,2,4-triazolo[1,5-a][1,3,5] triazin-5,7-dione and its 5-thioxo analogs on anti-thymidine phosphorylase and associated anti-angiogenic activities. *Eur. J. Med. Chem.* **2013**, *67*, 325-334.

57) Crestini, C.; Saladino, R. A new efficient and mild synthesis of 2-oxindoles by one-pot Wolff-Kishner-like reduction of isatin derivatives. *Synth. Commun.* **1994**, *24*, 2835-2841.

58) Amir, M.; Javed, S. A.; Kumar, H. Synthesis of some newer analogs of 4-hydroxyphenyl acetic acid as potent anti-inflammatory agents. *J. Chin. Chem. Soc.* **2008**, *55*, 201-208.

59) Prata, J. V.; Clemente, D. S.; Prabhakar, S.; Lobo, A. M.; Mourato, I.; Branco, P. S. Intramolecular addition of acyldiazenecarboxylates onto double bonds in the synthesis of heterocycles. *J. Chem. Soc., Perkin Trans. 1* **2002**,

4, 513-528.

60) Bakal, R. L; Gattani, S. G. Identification and development of 2,5-disubstituted oxadiazole as potential candidate for treatment of XDR and MDR tuberculosis. *Eur. J. Med. Chem.* **2012**, *47*, 278-282.

61) Tariq, Q. N.; Malik, S.; Khan, A.; Naseer, M. M.; Khan, S. U.; Ashraf, A.; Ashraf, M.; Rafiq, M.; Mahmood, K.; Tahir, M. N.; Shafiq, Z. Xanthenone based hydrazones as potent α- glucosidase inhibitors: synthesis, solid state self-assembly and in silico studies. *Bioorg. Chem.* **2019**, *84*, 372-383.

62) Khan, K. M.; Rasheed, M.; Ullah, Z.; Hayat, S.; Kaukab, F.; Choudhary, M. I.; Rahman, A.; Perveen, S. Synthesis and in vitro leishmanicidal activity of some hydrazides and their analogs. *Bioorg. Med. Chem.* **2003**, *11*, 1381-1387.

63) Karabanovich, G.; Zemanová, J.; Smutný, T.; Székely, R.; Šarkan, M.; Centárová, I.; Vocat, A.; Pávková, I.; Čonka, P.; Němeček, J.; Stolaříková, J.; Vejsová, M.; Vávrová, K.; Klimešová, V.; Hrabálek, A.; Pável, Cole, S. T.; Mikušová, K.; Roh, J. Development of 3,5-dinitrobenzylsulfanyl-1,3,4-oxadiazoles and thiadiazoles as selective antitubercular agents active against replicating and nonreplicating *Mycobacterium tuberculosis. J. Med. Chem.* **2016**, *59,* 2362-2380.
