# Supplementary material for: Development of a natural product optimization strategy for inhibitors against MraY, a promising antibacterial target
Source: Nat Commun. 2024 Jun 14;15:5085. doi: 10.1038/s41467-024-49484-7 (PMC11178787; doi:10.1038/s41467-024-49484-7)
Supplement: Supplementary file 3 — Reporting Summary [file 41467_2024_49484_MOESM3_ESM.pdf]

nature portfolio

Corresponding author(s): Kazuki Yamamoto
Satoshi Ichikawa

Last updated by author(s): May 17, 2024

Reporting Summary

Nature Portfolio wishes to improve the reproducibility of the work that we publish. This form provides structure for consistency and transparency in reporting. For further information on Nature Portfolio policies, see our Editorial Policies and the Editorial Policy Checklist.

Statistics

For all statistical analyses, confirm that the following items are present in the figure legend, table legend, main text, or Methods section.

n/a	Confirmed	
☐	☒	The exact sample size (*n*) for each experimental group/condition, given as a discrete number and unit of measurement
☐	☒	A statement on whether measurements were taken from distinct samples or whether the same sample was measured repeatedly
☒	☐	The statistical test(s) used AND whether they are one- or two-sided<br>*Only common tests should be described solely by name; describe more complex techniques in the Methods section.*
☒	☐	A description of all covariates tested
☒	☐	A description of any assumptions or corrections, such as tests of normality and adjustment for multiple comparisons
☐	☒	A full description of the statistical parameters including central tendency (e.g. means) or other basic estimates (e.g. regression coefficient) AND variation (e.g. standard deviation) or associated estimates of uncertainty (e.g. confidence intervals)
☒	☐	For null hypothesis testing, the test statistic (e.g. *F*, *t*, *r*) with confidence intervals, effect sizes, degrees of freedom and *P* value noted<br>*Give P values as exact values whenever suitable.*
☒	☐	For Bayesian analysis, information on the choice of priors and Markov chain Monte Carlo settings
☒	☐	For hierarchical and complex designs, identification of the appropriate level for tests and full reporting of outcomes
☒	☐	Estimates of effect sizes (e.g. Cohen's *d*, Pearson's *r*), indicating how they were calculated

Our web collection on statistics for biologists contains articles on many of the points above.

Software and code

Policy information about availability of computer code

Data collection	titude S 1.3
Data analysis	Graphpad Prism (version 4.0a) - commerically available, MotionCor2 1.2.6 - published and freely available, Relion 3.1/4.0 - published and freely available, cryoSPARC 3.0/3.1 - commerically available, Coot 0.8 - published and freely available, PHENIX 1.19 - published and freely available, UCSF Chimera 1.16 - published and freely available, ChimeraX-1.2 - published and freely available, MolProbity server -freely available, PyMOL 2.5.0 - open source, APBS Tools 2.1 - open source, Maestro 12.2 - commerically available

For manuscripts utilizing custom algorithms or software that are central to the research but not yet described in published literature, software must be made available to editors and reviewers. We strongly encourage code deposition in a community repository (e.g. GitHub). See the Nature Portfolio guidelines for submitting code & software for further information.

Data

Policy information about availability of data

All manuscripts must include a data availability statement. This statement should provide the following information, where applicable:

- Accession codes, unique identifiers, or web links for publicly available datasets
- A description of any restrictions on data availability
- For clinical datasets or third party data, please ensure that the statement adheres to our policy

Data supporting the findings of this manuscript are available from the corresponding author upon request. Details about characterization data of synthetic organic

compounds and experimental procedures are available in Supplementary Information. The coordinates generated in this study have been deposited in the Protein Data Base under accession code 9B70 [https://doi.org/10.2210/pdb9b70/pdb] (MraY-Analogue 2) and 9B71 [https://doi.org/10.2210/pdb9b71/pdb] (MraY-Analogue 3). The cryo-EM density maps generated in this study have been deposited in EMDB under accession code EMD-44293 [https://www.ebi.ac.uk/emdb/EMD-44293] (MraY-Analogue 2) and EMD-44294 [https://www.ebi.ac.uk/emdb/EMD-44294] (MraY-Analogue 3). Source data for Figure 2, Figure 5 and Tables 1 and Supplementary Figure 9, Figure 13, Figure 14, Figure 18-22, Table 1, and Table 2 can be found in Source Data.

Research involving human participants, their data, or biological material

Policy information about studies with human participants or human data. See also policy information about sex, gender (identity/presentation), and sexual orientation and race, ethnicity and racism.

Reporting on sex and gender	n/a
Reporting on race, ethnicity, or other socially relevant groupings	n/a
Population characteristics	n/a
Recruitment	n/a
Ethics oversight	n/a

Note that full information on the approval of the study protocol must also be provided in the manuscript.

Field-specific reporting

Please select the one below that is the best fit for your research. If you are not sure, read the appropriate sections before making your selection.

☒ Life sciences ☐ Behavioural & social sciences ☐ Ecological, evolutionary & environmental sciences

For a reference copy of the document with all sections, see nature.com/documents/nr-reporting-summary-flat.pdf

Life sciences study design

All studies must disclose on these points even when the disclosure is negative.

Sample size	Sample size was determined based on our experimental observations and experience, in which a particular sample size was found to provide reliable and reproducible results.
Data exclusions	No data were excluded.
Replication	The data in Figure 2, Figure 5c, Supplementary Table 1, Supplementary Fig. 19, 20, and 22 were performed once since they were intended for screening purposes. For compounds that appeared to be highly active, replicated experiments were performed (biological triplicates, technical singlicate). Experiments shown in Table 1, Fig. 5a ,5b, Supplementary Table 2, 3, Fig. 9, 13, 14, and 21 were performed in biological triplicates each containing technical singlicate). Experiment shown in Fig. 5d was performed with 4 or 5 mice (biologically independent). All experiments at replication were successful.
Randomization	Randomization is not relevant to this study because of the nature of the presented data, which are enzymatic inhibitory and antibacterial activity.
Blinding	Blinding was not required for this study because of the nature of the presented data, which are enzymatic inhibitory and antibacterial activity.

Reporting for specific materials, systems and methods

We require information from authors about some types of materials, experimental systems and methods used in many studies. Here, indicate whether each material, system or method listed is relevant to your study. If you are not sure if a list item applies to your research, read the appropriate section before selecting a response.

Materials & experimental systems

n/a	Involved in the study
☐	☒ Antibodies
☐	☒ Eukaryotic cell lines
☒	☐ Palaeontology and archaeology
☐	☒ Animals and other organisms
☒	☐ Clinical data
☒	☐ Dual use research of concern
☒	☐ Plants

Methods

n/a	Involved in the study
☒	☐ ChIP-seq
☒	☐ Flow cytometry
☒	☐ MRI-based neuroimaging

Antibodies

Antibodies used	Nanobodies were generated from a llama repertoire and used to aid data processing in cryo-EM.
Validation	This nanobody work was previously validated and published (PMID: 31266949). Also, it was validated in this paper via cryo-EM.

Eukaryotic cell lines

Policy information about cell lines and Sex and Gender in Research

Cell line source(s)	The cell lines used in this manuscript were purchased from ATCC (HepG2 ATCC HB-8065, THC116 ATCC CCL-247).
Authentication	No further authentication was performed for commercially available cell lines.
Mycoplasma contamination	Cell lines with negative mycoplasma tests were purchased and used.
Commonly misidentified lines (See ICLAC register)	No commonly misidentified cell lines were used in this study.

Animals and other research organisms

Policy information about studies involving animals; ARRIVE guidelines recommended for reporting animal research, and Sex and Gender in Research

Laboratory animals	Mouse (Female), Slc:ICR, 6-week-old, Dark/light cycle: 12-h light and 12-h dark cycles, Ambient temperature: 22±1 °C, Humidity: 50 ±10%
Wild animals	No wild animals were involved
Reporting on sex	Only females were used in the study according to a previous report (e.g. Antimicrob. Agents Chemother. 2017 61(7):e00281-1.)
Field-collected samples	No field-collected samples were involved
Ethics oversight	Ethical approval was obtained through the Wakunaga Pharmaceutical Company Institutional Animal Care and Use Committee (Protocol number: 0303)

Note that full information on the approval of the study protocol must also be provided in the manuscript.

Plants

Seed stocks	n/a
Novel plant genotypes	n/a
Authentication	n/a
